# Review of *Gasteruption* Latreille (Hymenoptera, Gasteruptiidae) from Iran and Turkey, with the description of 15 new species

**DOI:** 10.3897/zookeys.458.8531

**Published:** 2014-11-28

**Authors:** Cornelis van Achterberg, Ali Asghar Talebi

**Affiliations:** 1 Department of Terrestrial Zoology, Naturalis Biodiversity Center, Postbus 9517, 2300 RA Leiden, The Netherlands; 2 Department of Entomology, Faculty of Agriculture, Tarbiat Modares University, P.O. Box 14115-336, Tehran, Iran

**Keywords:** Revision, Gasteruptiidae, *Gasteruption*, key, new species, new synonyms, new records, lectotype designations, Iran, Turkey

## Abstract

The genus *Gasteruption* Latreille, 1796 (Hymenoptera: Evanioidea: Gasteruptiidae: Gasteruptiinae) from North Iran and Turkey is revised, keyed and fully illustrated for the first time. In total 36 species are treated of which 33 are recorded from Turkey and 23 from Iran. Fifteen species are new for science: *Gasteruption aciculatum* van Achterberg, **sp. n.**, *Gasteruption agrenum* van Achterberg, **sp. n.**, *Gasteruption brevibasale* van Achterberg & Saure, **sp. n.**, *Gasteruption coriacoxale* van Achterberg, **sp. n.**, *Gasteruption flavimarginatum* van Achterberg, **sp. n.**, *Gasteruption heminitidum* van Achterberg, **sp. n.**, *Gasteruption henseni* van Achterberg, **sp. n.**, *Gasteruption ischnolaimum* van Achterberg, **sp. n.**, *Gasteruption nigrapiculatum* van Achterberg, **sp. n.**, *Gasteruption paglianoi* van Achterberg & Saure, **sp. n.**, *Gasteruption pseudolaticeps* van Achterberg, **sp. n.**, *Gasteruption punctifrons* van Achterberg, **sp. n.**, *Gasteruption schmideggeri* van Achterberg & Saure, **sp. n.**, *Gasteruption scorteum* van Achterberg, **sp. n.** and *Gasteruption smitorum* van Achterberg, **sp. n.** Twenty-one species are reported new for Turkey and 16 species new for Iran. Fifteen new synonyms are proposed: *Foenus terrestris* Tournier, 1877, *Gasteruption trifossulatum* Kieffer, 1904, and *Gasteruption ignoratum* Kieffer, 1912, of *Gasteruption caucasicum* (Guérin-Méneville, 1844); *Gasteruption daisyi* Alekseev, 1993, of *Gasteruption dolichoderum* Schletterer, 1889; Gasteruption assectator var. nitidulum Schletterer, 1885, of *Gasteruption freyi* (Tournier, 1877); *Gasteruption schossmannae* Madl, 1987, of *Gasteruption hastator* (Fabricius, 1804); *Gasteryption fallaciosum* Semenov, 1892, *Gasteruption dubiosum* Semenov, 1892 and *Gasteruption obsoletum* Semenov, 1892, of *Gasteruption insidiosum* Semenov, 1892; *Gasteryption schewyrewi* Semenov, 1892, of *Gasteruption jaculator* (Linnaeus, 1758); *Gasteruption floreum* Szépligeti, 1903, of *Gasteruption lugubre* Schletterer, 1889; *Gasteruption trichotomma* Kieffer, 1904, and *Gasteruption palaestinum* Pic, 1916, of *Gasteruption merceti* Kieffer, 1904; *Gasteryption foveiceps* Semenov, 1892, of *Gasteruption nigrescens* Schletterer, 1885, and *Gasteruption libanense* Pic, 1916, of *Gasteruption syriacum* Szépligeti, 1903. *Gasteruption lugubre* Schletterer, 1889, is recognised as a valid species. Lectotypes are designated for *Ichneumon assectator* Linnaeus, 1758; *Ichneumon jaculator* Linnaeus, 1758; *Foenus terrestris* Tournier, 1877; *Foenus freyi* Tournier, 1877; *Foenus nigripes* Tournier, 1877; *Foenus goberti* Tournier, 1877; *Foenus granulithorax* Tournier, 1877; *Foenus minutus* Tournier, 1877; *Foenus borealis* Thomson, 1883; *Faenus diversipes* Abeille de Perrin, 1879; *Foenus rugulosus* Abeille de Perrin, 1879; *Faenus obliteratus* Abeille de Perrin, 1879; *Faenus undulatum* Abeille de Perrin, 1879; *Faenus variolosus* Abeille de Perrin, 1879; *Gasteruption distinguendum* Schletterer, 1885; *Gasteruption laeviceps* Schletterer, 1885; *Gasteruption thomsonii* Schletterer, 1885; *Gasteruption foveolatum* Schletterer, 1889; *Gasteruption sowae* Schletterer, 1901; *Gasteruption foveolum* Szépligeti, 1903; *Gasteruption floreum* Szépligeti, 1903; *Gasteruption caudatum* Szépligeti, 1903; *Gasteruption syriacum* Szépligeti, 1903; *Gasteruption merceti* Kieffer, 1904 and *Gasteruption ignoratum* Kieffer, 1912. A neotype is designated for *Gasteruption tournieri* Schletterer, 1885.

## Introduction

The family Gasteruptiidae is a small group of wasps comprising about 500 described species in two subfamilies, Gasteruptiinae (four genera) (Macedo 2009, 2011; Zhao et al. 2012) and Hyptiogastrinae (two genera) (Jennings and Austin 2002). Gasteruptiidae are traditionally classified in the superfamily Evanioidea, together with the Aulacidae and Evaniidae (Jennings and Austin 2000). All three families share the highly inserted metasoma and the mid-coxal articulation. However, the biology of each family is different as are the thoracic musculature, the internal skeletal structure, the antenna cleaner and the shape of the ovipositor. According to these anatomical characters the monophyly of the superfamily has been questioned (Quicke 1997), but recent molecular evidence supports their monophyly (Heraty et al. 2011; Sharkey et al. 2012). Gasteruptiidae are easily recognized from the other apocritan hymenopterans by the elongated “neck” (propleuron) the swollen hind tibiae and the highly attached slender metasoma. Adults are free-living insects feeding on nectar mainly on flowers with easily accessible nectar such are of the families Apiaceae, Asteraceae or Euphorbiaceae, but likely at least some *Gasteruption* species feed on both nectar and pollen (Jennings and Austin 2004). Gasteruptiidae are also known by their hovering flight during inspection of bee nests (van Achterberg 2013). The larvae feed on the larval food of solitary bees, after consuming the egg or larva of the bee (Malyshev 1966). They select bees of the subfamilies Apinae, Colletinae and Megachilinae nesting in stems or in wood, and less often in clay banks or other vertical soil substrates (Zhao et al. 2012; van Achterberg 2013); as far as known, bees nesting in horizontal soil substrates are far less attacked. In Australia members of the Hyptiogastrinae do attend bee nests in flat ground (Houston 1987). There is only indirect evidence that Gasteruptiinae attack wasp nests, e.g. Crabronidae, Sphecidae and solitary Vespidae (Eumeninae) (Crosskey 1951; Gauld and Hanson 1995, 2006; Jennings and Austin 1997a, 1997b, 2004). Metamorphosis takes place inside the host’s nest where the gasteruptiid pupa hibernates until the next spring or summer (Malyshev 1966; He 2004; Jennings and Austin 2004).

All known gasteruptiids from the Palaearctic Region belong to the subfamily Gasteruptiinae and to the genus *Gasteruption* Latreille, 1796. A revision of the 30 European species so far recorded (Madl 2004) is being prepared (van Achterberg and Saure, in prep.) and the rich fauna of Iran and Turkey is revised separately in this paper for the first time. The revision became possible after extensive sampling with Malaise traps in the northern part of Iran; so far only some accidentally collected specimens are recorded (Semenov 1892; Hedwig 1957; Tirgari 1975; Samin and Bagriacik 2012). The rich manually collected Asia Minor collection of the Oberösterreichisches Landesmuseum (Biologiezentrum) at Linz allowed the inclusion of the fauna of Turkey. Seven species have been reported from Iran (only two under a valid name) and 14 species from Turkey, but only eight under valid names. Of the remaining six species two are synonyms of two other reported species (Table 1). After this study 23 species are known from Iran of which eight are new to science; from Turkey 34 species are reported, of which 13 are new to science (Table 2).

**Table 1.** *Gasteruption* species known from Iran and Turkey.

Name	Reported from Turkey by	Reported from Iran by	Valid name
*Gasteruption assectator*	Yildirim et al. 2004	----	*Gasteruption assectator*
*Gasteruption diversipes*	Madl 1988	Samin and Bagriacik 2012	*Gasteruption diversipes*
*Gasteruption erythrostomum*	Madl 1987	Samin and Bagriacik 2012	*Gasteruption insidiosum*
*Gasteruption foveolum*	----	Tirgari 1975	*Gasteruption laticeps*
*Gasteruption freyi*	Yildirim et al. 2004	----	*Gasteruption freyi*
*Gasteruption jekylljaechi*	Madl 1987	----	*Gasteruption merceti*
*Gasteruption jaculator*	Yildirim et al. 2004	Tirgari 1975	*Gasteruption jaculator*
*Gasteruption nigrescens*	Schmid-Egger and Saure 2010	----	*Gasteruption nigrescens*
*Gasteruption opacum*	Yildirim et al. 2004	----	*Gasteruption opacum*
*Gasteruption pedemontanum*	Yildirim et al. 2004	Semenov 1892	*Gasteruption caucasicum*
*Gasteruption pyrenaicum*	Yildirim et al. 2004	----	*Gasteruption merceti*
*Gasteruption rubricans*	Semenov 1892	Hedwig 1957	*Gasteruption hastator*
*Gasteruption tibiale*	Yildirim et al. 2004	----	*Gasteruption hastator*
*Gasteruption tournieri*	Madl 1987	Samin and Bagriacik 2012	*Gasteruption tournieri*
*Gasteruption undulatum*	Madl 1988	----	*Gasteruption undulatum*

**Table 2.** Revised list of *Gasteruption* species from Iran and Turkey.

Name	First record for Turkey	First record for Iran
*Gasteruption aciculatum* van Achterberg, sp. n.	this paper	----
*Gasteruption agrenum* van Achterberg, sp. n.	this paper	this paper
*Gasteruption assectator* (Linnaeus, 1758)	Yildirim et al. 2004	this paper
*Gasteruption brevibasale* van Achterberg & Saure, sp. n.	this paper	----
*Gasteruption caucasicum* (Guérin-Méneville, 1844)	Yildirim et al. 2004	Semenov 1892
*Gasteruption coriacoxale* van Achterberg, sp. n.	this paper	this paper
*Gasteruption diversipes* (Abeille de Perrin, 1879)	Madl 1988	Samin and Bagriacik 2012
*Gasteruption dolichoderum* Schletterer, 1889	this paper	this paper
*Gasteruption flavimarginatum* van Achterberg, sp. n.	this paper	----
*Gasteruption freyi* (Tournier, 1877)	Yildirim et al. 2004	----
*Gasteruption goberti* (Tournier, 1877)	this paper	----
*Gasteruption hastator* (Fabricius, 1804)	Semenov 1892	Hedwig 1957
*Gasteruption heminitidum* van Achterberg, sp. n.	----	this paper
*Gasteruption henseni* van Achterberg, sp. n.	this paper	----
*Gasteruption insidiosum* Semenov, 1892	Madl 1987	Samin and Bagriacik 2012
*Gasteruption ischnolaimum* van Achterberg, sp. n.	this paper	this paper
*Gasteruption jaculator* (Linnaeus, 1758	Yildirim et al. 2004	Tirgari 1975
*Gasteruption laticeps* (Tournier, 1877)	this paper	Tirgari 1975
*Gasteruption lugubre* Schletterer, 1889, stat. rev.	this paper	----
*Gasteruption merceti* Kieffer, 1904	Madl 1987	this paper
*Gasteruption minutum* (Tournier, 1877)	this paper	this paper
*Gasteruption nigrapiculatum* van Achterberg, sp. n.	----	this paper
*Gasteruption nigrescens* Schletterer, 1885	Schmid-Egger and Saure 2010	this paper
*Gasteruption opacum* (Tournier, 1877)	Yildirim et al. 2004	this paper
*Gasteruption paglianoi* van Achterberg & Saure, sp. n.	this paper	----
*Gasteruption phragmiticola* Saure, 2006	this paper	this paper
*Gasteruption pseudolaticeps* van Achterberg, sp. n.	this paper	this paper
*Gasteruption punctifrons* van Achterberg, sp. n.	this paper	this paper
*Gasteruption schlettereri* Magretti, 1890	this paper	this paper
*Gasteruption schmideggeri* van Achterberg & Saure, sp. n.	this paper	this paper
*Gasteruption scorteum* van Achterberg, sp. n.	this paper	----
*Gasteruption smitorum* van Achterberg, sp. n.	this paper	----
*Gasteruption syriacum* Szépligeti, 1903	this paper	----
*Gasteruption tournieri* Schletterer, 1885	Madl 1987	Samin and Bagriacik 2012
*Gasteruption undulatum* (Abeille de Perrin, 1879)	Madl 1988	----
*Gasteruption variolosum* (Abeille de Perrin, 1879)	this paper	this paper

## Material and methods

**Figure 1.**
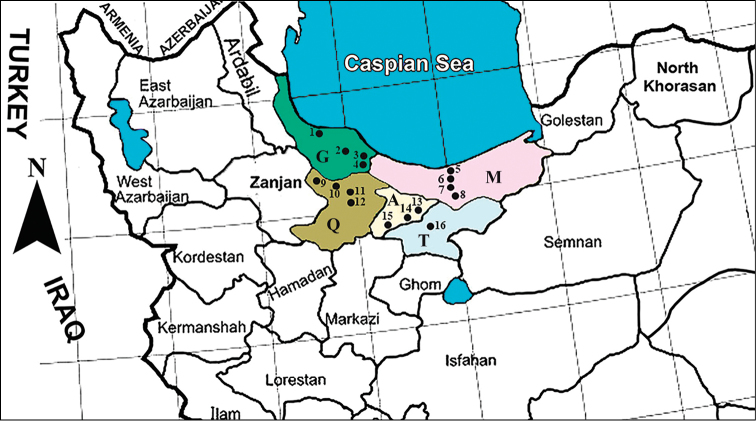
Sampling map of *Gasteruption* species in North Central Iran; **G**= Gilan, **M**= Mazandaran, **Q**= Qazvin, **A**= Alborz and **T**= Tehran provinces.

The specimens were collected by hand net or sweep net (Turkey) or in Malaise traps and with sweep net (Iran). The material collected during 2011–2012 is stored in 70% ethanol, prepared using the AXA method (van Achterberg 2009; van Achterberg et al. 2010) and glued on card points; older specimens are collected dry, mounted on card points or pinned. In Iran the present study was carried out in 16 localities in Gilan and Tehran provinces in northern Iran (Fig. 1). Alborz province is the recently renamed western part of the former Tehran province.

The Alborz Mountains separate the subtropical Caspian Sea area (Gilan and Mazandaran) from Tehran province. Gilan (or Guilan) province with an area of 14.042 km² extends along the Caspian Sea and in the northern slopes of the Alborz Mountains. Situated between the high mountains of Alborz and the Caspian Sea, Gilan has a humid subtropical climate with heavy annual rainfall of about 1500 mm, moderate temperature and high relative humidity leading to diverse vegetation. The main part of the precipitation is in autumn and winter and October is the rainiest month of the year. The relative humidity is about 80%, which decreases with altitude. The minimum temperature at sea level is +3°C in January. From March on it rises and reaches its maximum of around 30°C in July-August. The Alborz Mountains provide many unique types of vegetation at various altitudes in addition to the Caspian coast flora.

The well-known natural biome of this region is the Caspian Hyrcanian mixed forest but the coastal plains have been nearly entirely converted to urban sites and wet rice fields. As the elevation increases, the flora gradually differentiates and diversifies from humid forests below 700 m a.s.l. to pure Oriental beech or mixed forests at middle altitude (700–1500 m a.s.l.). Shrub lands and steppes occur in the upper mountains and the highest elevations are covered with alpine tundra and meadows (Marvie Mohajer 2006). Tehran province covers an area of 18,909 km² and is located on the southern slopes of the Alborz Mountains with various vegetation types and climates. This area receives an average annual rainfall of about 240 mm, which usually starts from October. The maximum precipitation occurs in March with 47 mm and April with 34 mm. The southern part of the province has a semi-arid steppe climate and the northern one a more alpine character. The climate in the mountain regions of northern Tehran is cold and semi-humid and in higher elevations is cold with a long winter. The coldest months of the year are December- February with minus 1–2°C. The spring begins in March and the temperature gradually rises to 30–35°C from mid July to mid September. The province is the most densely populated region in Iran with many different valleys and rivers which makes it very heterogeneous. The specimens were collected during March to November at four locations per province (Tehran (including Alborz) and Gilan). Two Malaise traps were placed in each location. The geographical and main floristic characteristics of each location are presented in Table 3. Sampling procedures were similar at the different locations. Malaise traps were placed in different habitats such as forest, range land or orchards. The specimens were extracted from the Malaise traps and sorted weekly and stored in 70% ethanol.

**Figure 2.**
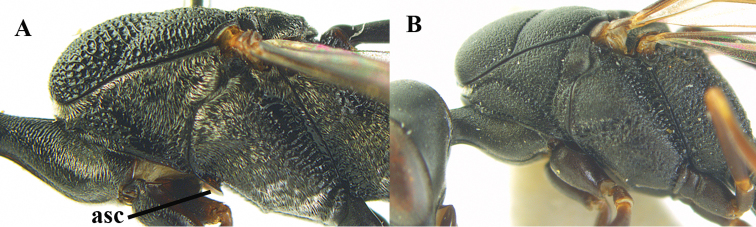
**A** Antesternal carina (“asc”) present **B** antesternal carina absent.

**Figure 3.**
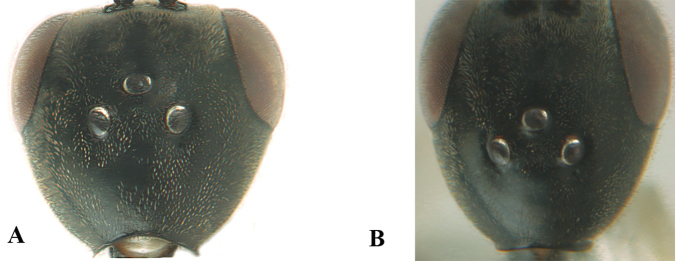
Head of same female specimen of *Gasteruption dolichoderum* Schletterer showing effect of the middle of the vertex (**A**) or the stemmaticum (= ocellar triangle; **B**) in plane of the objective of the binocular microscope.

**Figure 4.**
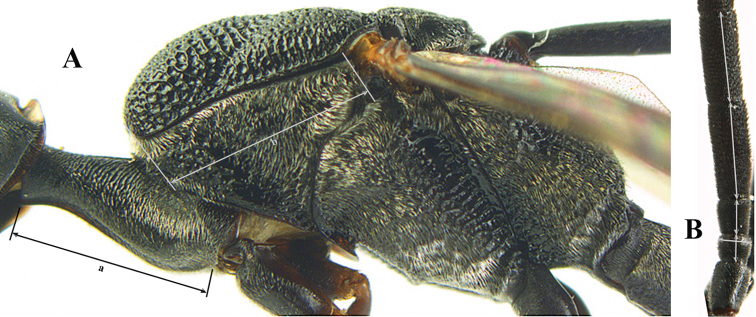
Measurements (**A**) of the relative length of the propleuron (a) and length of the mesoscutum in front of the tegulum (b) and (**B**) the length and maximum width of the basal antennal segments.

The antesternal carina (van Achterberg in Zhao et al. 2012; van Achterberg 2013) is the lamelliform upcurved anterior ridge of the mesopleuron (directly behind the base of the fore coxa; “asc”, in Fig. 2A); in many species the anterior ridge is not or only slightly lamelliform and straight (Fig. 2B). The middle of the vertex should be in plane of objective of binocular microscope (Fig. 3A). For the other terminology, see Zhao et al. (2012). Measurements are performed as indicated in Fig. 4 and in van Achterberg (1988). Additional non-exclusive characters in the key are between square brackets. The association of males with the females is based on similarity; in the few cases no males are available distinctive and probably non-sexual characters of the female are tentatively used for the inclusion in the key as far as possible. A new record for the country is indicated by an asterisk. The following abbreviations are used for the depositories: BZL = Oberösterreichisches Landesmuseum, Biologiezentrum, Linz; CSC = personal collection of C. Saure, Berlin; CSEC = personal collection of C. Schmid-Egger, Berlin; ETHZ = Eidgenossische Technische Hochschule, Zürich; MCG = Museo Civico di Storia Naturale, Genoa; MHNG = Museum of Natural History of the City of Geneva, Genève; MNHN = Museum National d’Histoire Naturelle, Paris; MNCN = Museo Nacional de Ciencias Naturales, Madrid; MTMA
= Magyar Termeszettudományi Múzeum Allatára, Budapest; MZL = Museum Zoologie de Lausanne, Lausanne; NHRS = Naturhistoriska Riksmuseet, Stockholm; NMW = Naturhistorische Museum, Wien; RMNH = Naturalis Biodiversity Center, Leiden; TMUT = Tarbiat Modares University, Tehran; ZIL = Museum of Zoology, Biological Museums of Lund University, Lund; ZISP = Zoological Institute, Academia NAUK, St. Petersburg; ZMH = Zoological Museum, University of Helsinki, Helsinki.

Both authors of the names of the three species described in this paper by Dr C. Saure and CvA (*Gasteruption brevibasale* van Achterberg & Saure, sp. n., *Gasteruption paglianoi* van Achterberg & Saure, sp. n., and *Gasteruption schmideggeri* van Achterberg & Saure, sp. n.) are responsible for making the name available under the International Code of Zoological Nomenclature (Article 50.1.2, etc.) i.e. both are responsible for coining the name and for satisfying all other criteria for availability.

**Table 3.** Positions of Malaise traps in northern Iran with *Gasteruption* specimens.

Trap no.	Province	Locality	Geographical coordinates	Altitude (m)	Habitat
1	Gilan	Eshman-komachal	N37°22'03", E49°57'57"	-1	humid forest
2	Gilan	Ghazichak	N36°45'52", E50°00'01"	1787	Hazelnut/pasture
3	Gilan	Orkom	N36°45'44 E50°18'11"	1225	Deciduous forests/ hazelnut
4	Gilan	Ziaz	N36°52'27", E50°13'24"	490	Hazelnut
5	Mazandaran	Joorband	N36°26'17", E52°07'16"	272	Garden/rice field
6	Mazandaran	Tangehvaz	N36°21'55", E52°06'10"	702	Deciduous forests
7	Mazandaran	Noor	N36°34'52", E52°02'45"	-14	Forests
8	Mazandaran	Gaznasara	N36°16'56", E52°10'58"	2032	Pasture
9	Qazvin	Loshan	N36°40'14", E49°25'38"	292	Olive
10	Qazvin	Koohin	N36°22'14", E49°40'02"	1514	Rosaceus orchard
11	Qazvin	Zereshk Road, Barajin	N36°21'39", E50°03'55"	1541	Rosaceus orchard
12	Qazvin	Zereshk	N36°25'23", E50°06'37"	1926	Rosaceus orchard
13	Alborz	Arangeh & Sarziarat	N35°55'07", E51°05'09"	1891–1980	Rosaceus orchard
14	Alborz	Shahrestanak	N35°57'34", E51°22'19"	2305	Rosaceus orchard / pasture
15	Alborz	Karaj	N35°46'20", E50°56'05"	1278	Rosaceus orchard
16	Tehran	Shahriar	N35°40'08", E50°56'56"	1168	Rosaceus orchard

## *Gasteruption* Latreille, 1796

### Key to species of the genus *Gasteruption* Latreille from Iran and Turkey

**Table:** 

1	Ovipositor present (a); antenna with 14 segments (b; females)	**2**
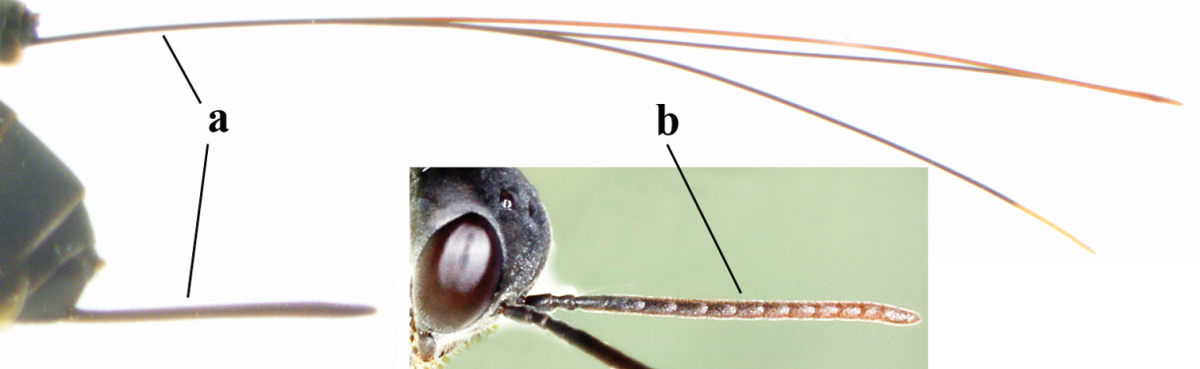		
–	Ovipositor absent (aa); antenna with 13 segments (bb; males); [if males are unknown the species is provisionally inserted]	**39**
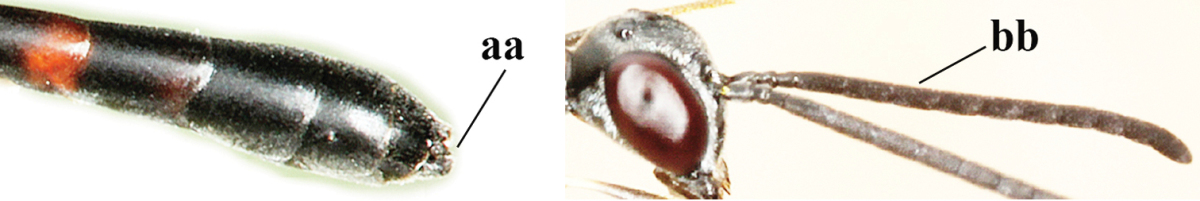		
2	Apex of ovipositor sheath blackish or dark brown, if narrowly pale apically then white or ivory part at most 0.8 times as long as hind basitarsus (a)	**3**
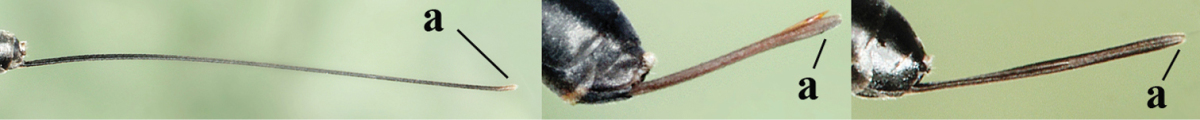		
–	Apex of ovipositor sheath distinctly white or ivory, pale part at least about as long as hind basitarsus (aa), rarely shorter	**23**
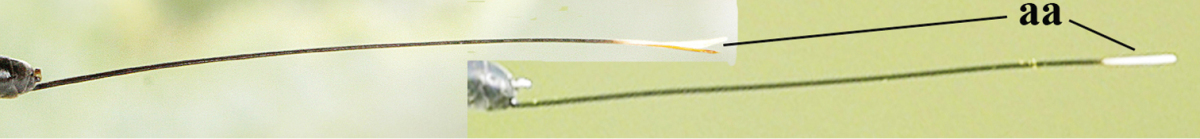		
3	Ovipositor sheath 0.6–1.1 times as long as hind tibia and 0.6–0.7 times as long as hind tibia and tarsus combined (a); incision of hypopygium shallow V-shaped and up to apical 0.2 (b); occipital carina obsolescent to narrowly lamelliform medio-dorsally (c)	**4**
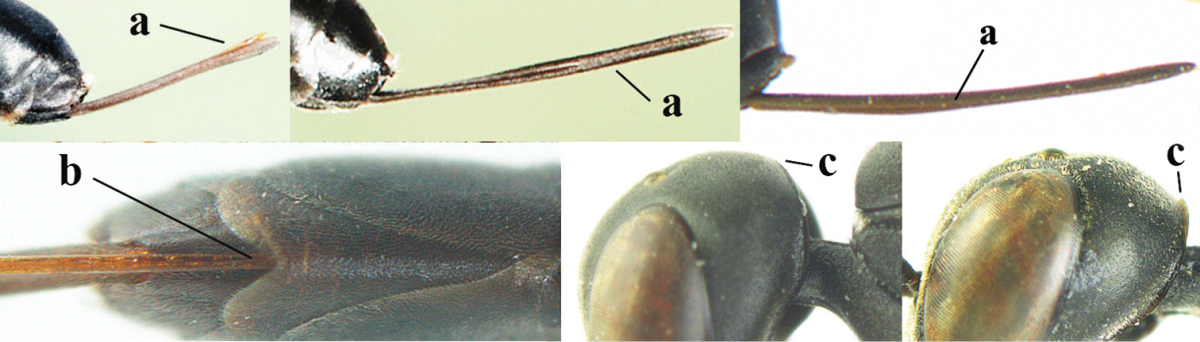		
–	Ovipositor sheath 1.8–6.3 times as long as hind tibia and 1.1–3.8 times as long as hind tibia and tarsus combined (aa); incision of hypopygium variable, either slit-like and up to apical 0.4–0.5 (bb) or shallower and up to apical 0.2–0.3 in *G. variolosum, dolichoderum* and *merceti*; occipital carina obsolescent (c) or distinctly lamelliform medio-dorsally (cc)	**11**
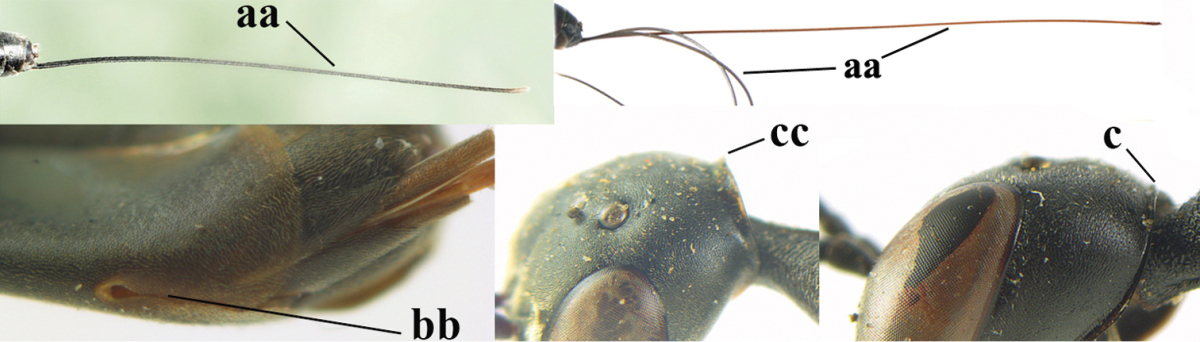		
4	Clypeus with rather large shallow depression (a); mesoscutum densely reticulate-rugulose or -rugose (b); hind basitarsus stout (c); head and mesosoma laterally mainly reddish-brown (d), but sometimes black; first discal cell of fore wing glabrous, rarely with a few setae (e)	***Gasteruption hastator* (Fabricius, 1804)**
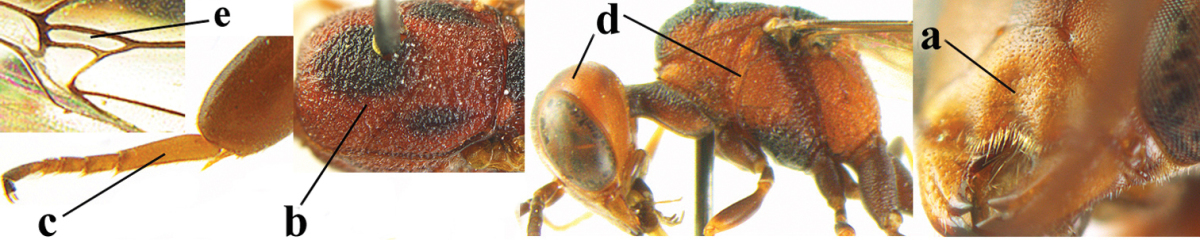		
–	Clypeus with small depression or depression obsolescent (aa); mesoscutum densely coriaceous, rugose or rugulose (bb); hind basitarsus slenderer (cc), rarely similarly stout; head and usually mesosoma laterally black (bb); first discal cell of fore wing usually with some setae (ee)	**5**
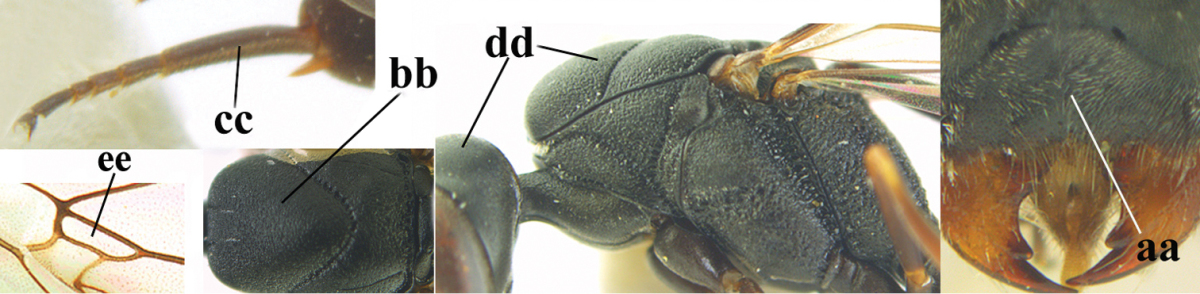		
5	Occipital carina narrow lamelliform (a); hind tibial spurs blackish or dark brown (b); hind tibia slightly less swollen (c); hind tibia dark brown subbasally (d); [ovipositor sheath 1.2–1.4 times as long as hind tibia]	***Gasteruption freyi* (Tournier, 1877)**
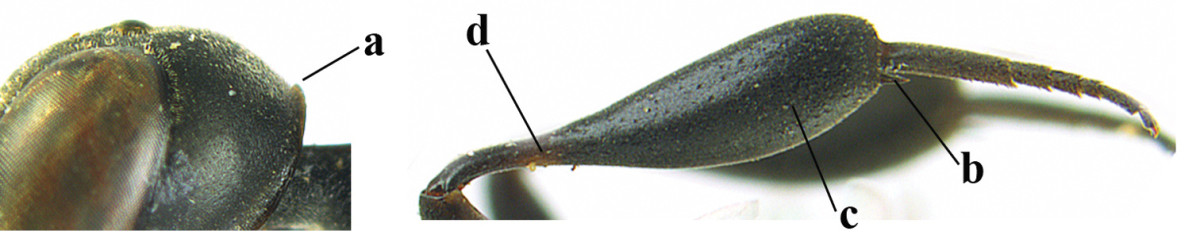		
–	Occipital carina obsolescent, non-lamelliform (aa); hind tibial spurs yellowish-brown, brown (bb) or dark brown; hind tibia more swollen, resulting in a distinctly convex ventral border (cc); hind tibia with ivory subbasal patch (dd)	**6**
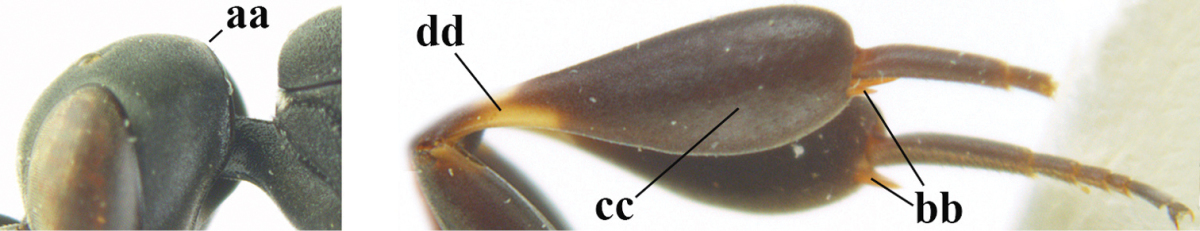		
6	Mesoscutum coarsely rugose, different from very finely aciculate vertex (a) **and** antero-lateral tooth of pronotum present, protruding anteriorly and rather wide basally (b); hind basitarsus more or less widened basally in dorsal view (c); hind tarsus (except telotarsus) dorsally brownish-yellow to yellowish-brown (d), rarely infuscate; ovipositor sheath 1.2–1.4 times as long as hind tibia (e); hind tibial spurs pale brown (f); [mandible dark brown basally]	***Gasteruption undulatum* (Abeille de Perrin, 1879)**
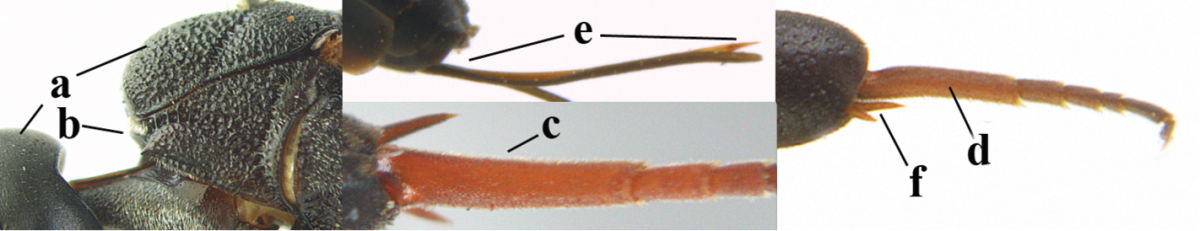		
–	Mesoscutum and head similarly coriaceous, at most mesoscutum moderately rugulose (aa); antero-lateral tooth of pronotum absent or obsolescent (bb), if present then protruding laterally and narrower basally; hind basitarsus usually parallel-sided basally in dorsal view (cc), but sometimes widened (ccc); hind tarsus dorsally brown, dark brown, ivory or blackish (dd); ovipositor sheath 0.4–1.3 times as long as hind tibia (ee); hind tibial spurs dark brown or brown (ff)	**7**
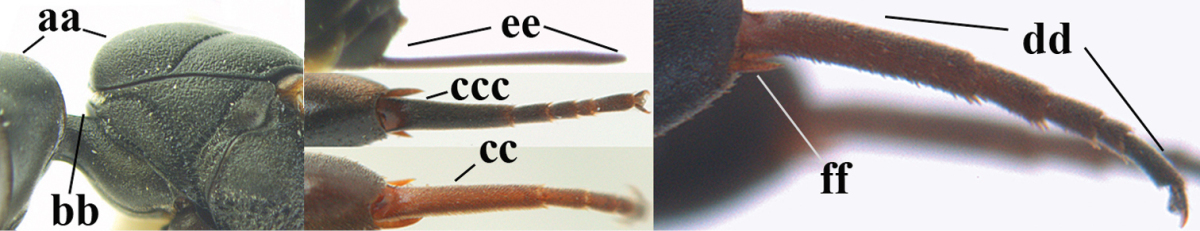		
7	Basal petiolate part of hind tibia shorter and wider in dorsal view (a); hind femur shallowly depressed ventrally, at least subbasally (b)	***Gasteruption brevibasale* sp. n.**
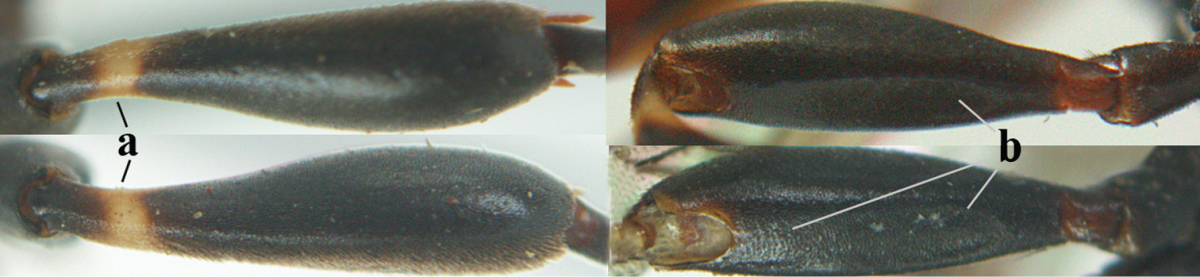		
–	Basal petiolate part of hind tibia longer and narrower in dorsal view (aa); hind femur slightly convex or flattened ventrally (bb)	**8**
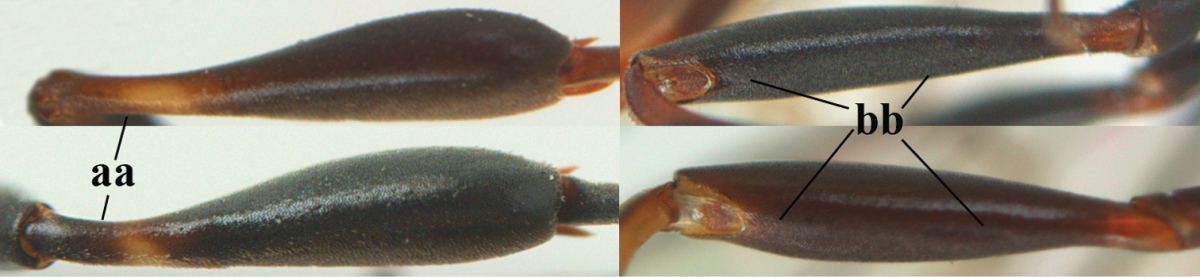		
8	Head in anterior view protruding below lower level of eyes 0.5–0.6 times length of second antennal segment and 0.4–0.6 times basal width of mandible and mandibular condylus distinctly below lower level of eyes (a); in lateral view condylar incision of malar space remains far removed from eye (b); ovipositor sheath 0.4–0.9 times as long as hind tibia (c); first discal cell of fore wing usually directly narrowed (d)	***Gasteruption minutum* (Tournier, 1877)**
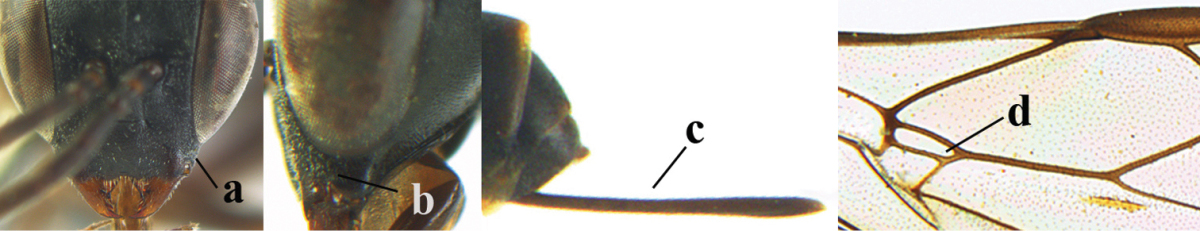		
–	Head in anterior view slightly protruding below lower level of eyes by less than basal width of mandible and mandibular condylus near lower level of eyes (aa); in lateral view condylar incision of malar space close to eye (bb); ovipositor sheath 0.8–1.3 times as long as hind tibia (cc); first discal cell of fore wing usually gradually narrowed (dd)	**9**
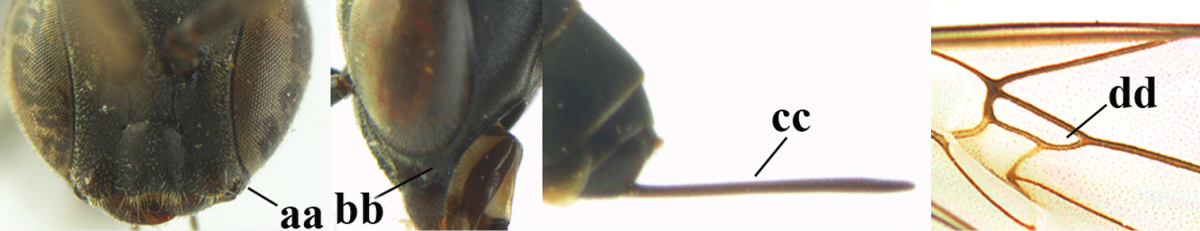		
9	Mandible dark brown or reddish brown basally (a), rarely brownish yellow; basal depression of mandible rather large and deep (b); fifth (= pre-apical) sternite dark brown or blackish or narrowly pale medio-apically (c); [mandible slightly less convex; mesosoma black; hind basitarsus usually rather slender]	***Gasteruption assectator* (Linnaeus, 1758)**
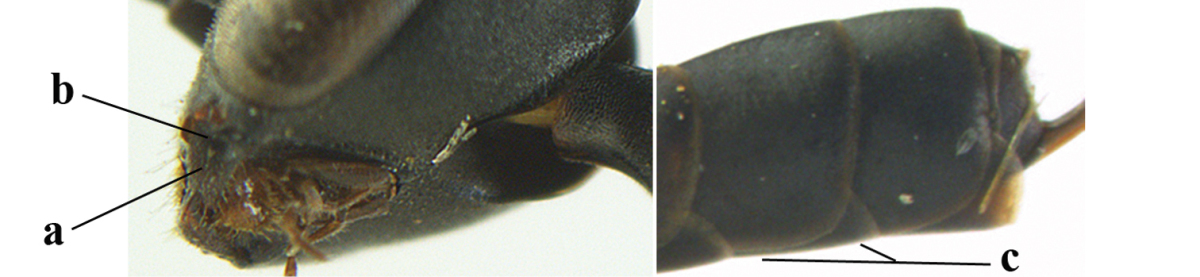		
–	Mandible pale yellow basally (aa); basal depression of mandible smaller and shallower (bb); fifth sternite yellowish brown medio-apically (cc)	**10**
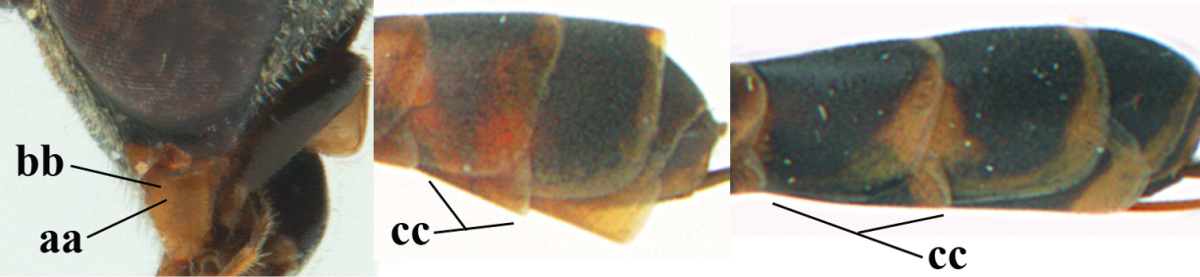		
10	Antenna (except dark brown basal third) yellowish brown (a); head subparallel-sided behind eyes (b); hypopygium entirely pale yellowish brown (c); hind femur distinctly inflated (d); hind basitarsus entirely brown (e); mesosoma 1.8–1.9 times as long as high	***Gasteruption paglianoi* sp. n.**
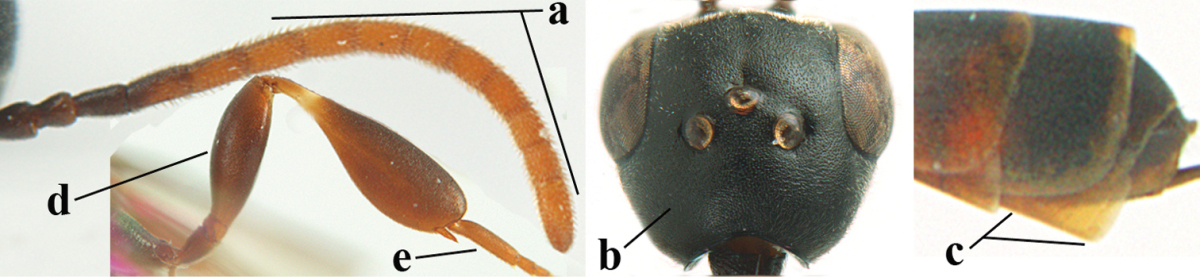		
–	Antenna dark brown (aa); head directly narrowed behind eyes (bb); at least basal half of hypopygium dark brown (cc); hind femur slightly inflated (dd); hind basitarsus ivory medially (ee); mesosoma 1.5–1.6 times as long as high	***Gasteruption flavimarginatum* sp. n.**
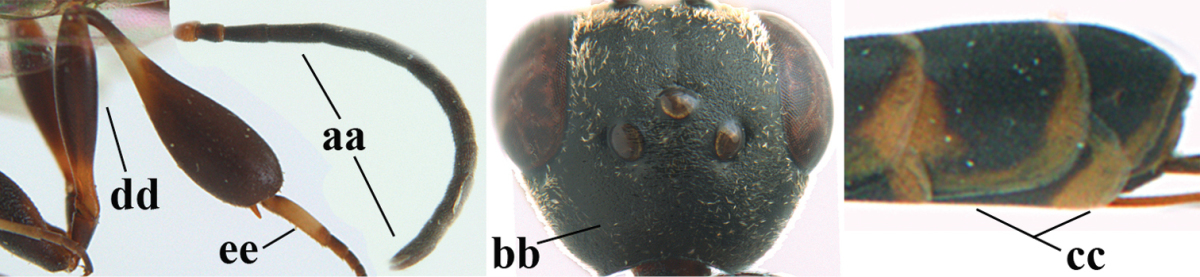		
11	Vertex distinctly bulging near occipital carina (a); ovipositor sheath 1.8–2.0 times as long as hind tibia (b); hind basitarsus stout (c)	***Gasteruption variolosum* (Abeille de Perrin, 1879)**
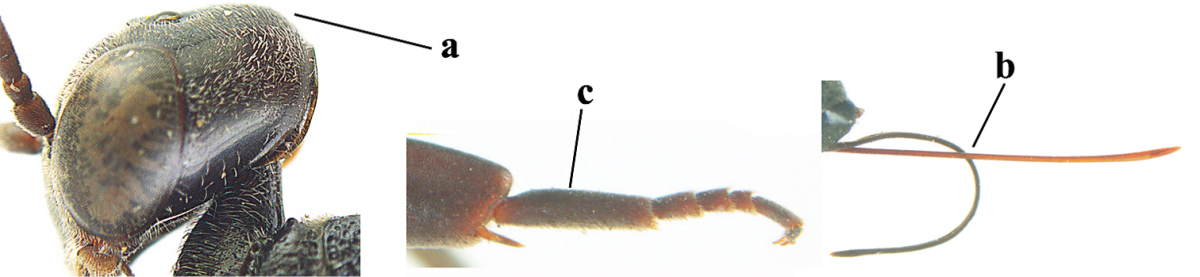		
–	Vertex weakly convex or flat in front of occipital carina (aa); ovipositor sheath 2.5–5.6 times as long as hind tibia (bb); hind basitarsus slender (cc)	**12**
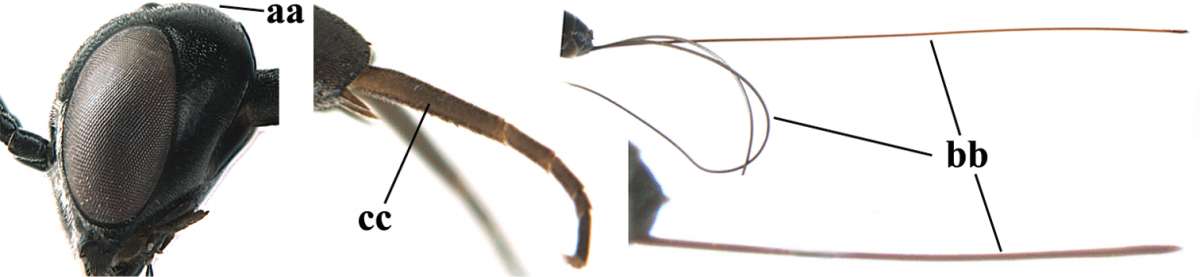		
12	Head in anterior view elongate (“fez-shaped”; a); vertex flattened (b); propleuron 1.0–1.3 times distance from tegulae to anterior border of mesoscutum (c); incision of hypopygium up to apical 0.2 (d)	***Gasteruption dolichoderum* Schletterer, 1889**
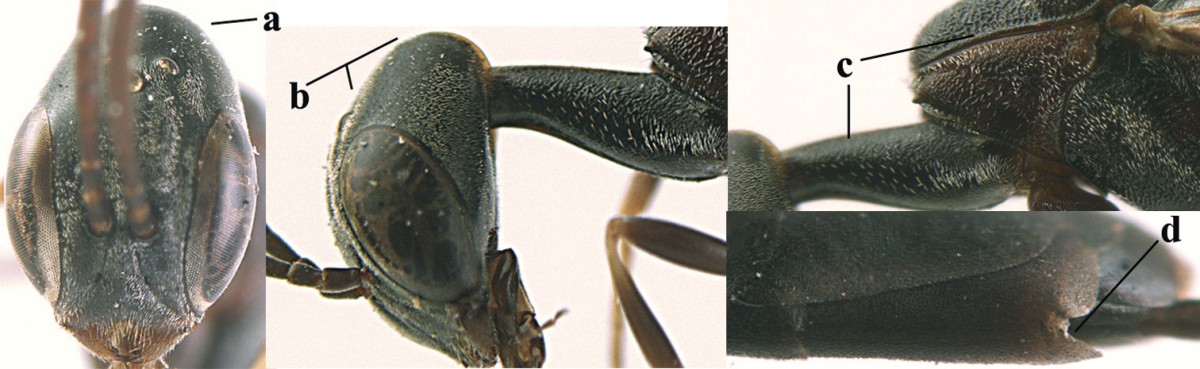		
–	Head in anterior view normal (aa); vertex convex (bb); propleuron usually 0.7–1.0 times distance from tegulae to anterior border of mesoscutum (cc); incision of hypopygium up to apical 0.3–0.5 (dd)	**13**
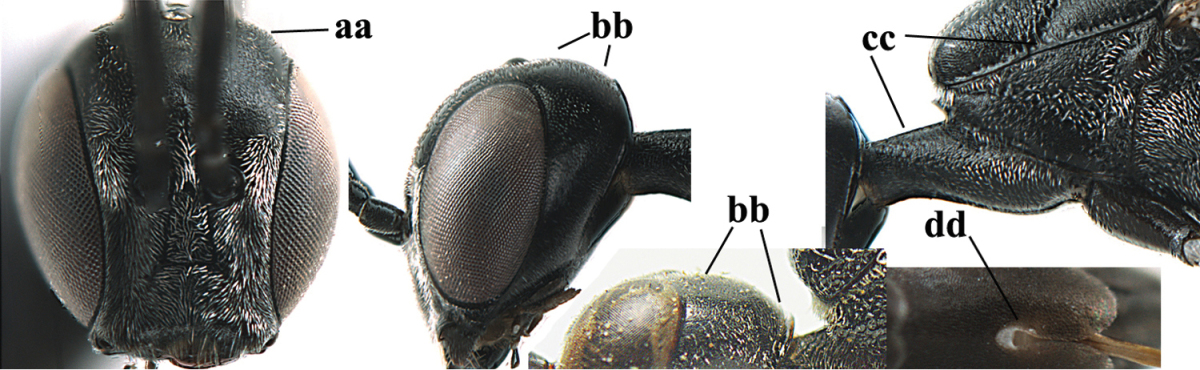		
13	Occipital carina non-lamelliform medio-dorsally (a) **and** pronotal side matt and ventrally coriaceous (b) or largely so and with some rugulae; mandible black or dark brown basally (c); apex of ovipositor sheath distinctly ivory (d), rarely pale part reduced; [head more or less cylindrical behind eyes or nearly so; stemmaticum sculptured medially and without distinct punctures; ovipositor sheath 4.8–6.5 times as long as hind tibia and 1.4–1.7 times as long as metasoma]	**14**
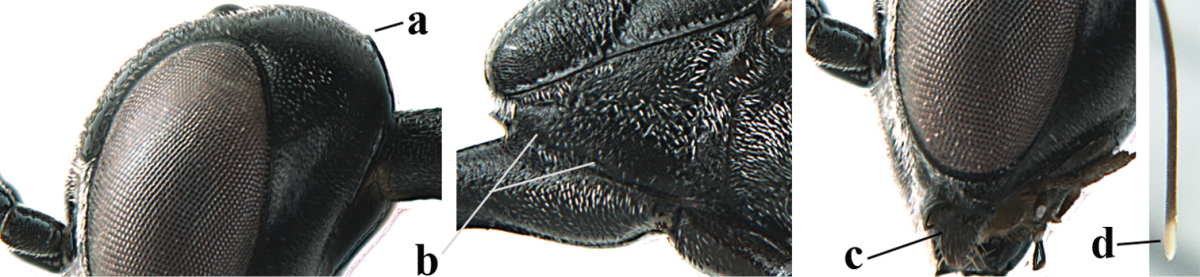		
–	Occipital carina at least narrowly lamelliform medio-dorsally (aa) **and/or** pronotal side more or less shiny and ventrally partly rugulose or rugose (bb); mandible often yellowish brown basally (cc); apex of ovipositor sheath often black (dd); [vertex often somewhat depressed medio-posteriorly]	**15**
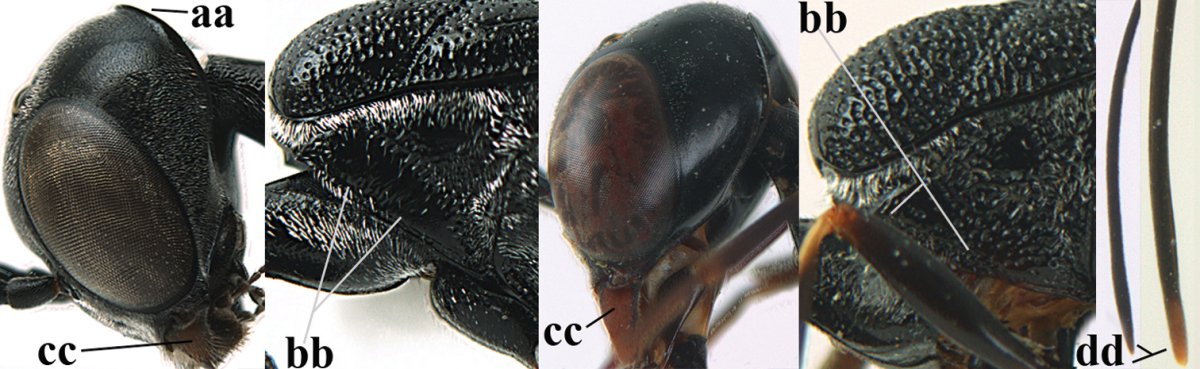		
14	Hind coxa coriaceous or finely rugulose dorsally (a); face slightly narrower (b); propleuron in ventral view slenderer (c); part of punctures of middle lobe of mesoscutum separated from rugulae or punctures obsolescent (d); head in dorsal view usually rather narrowed posteriorly (e), but sometimes subparallel-sided	***Gasteruption coriacoxale* sp. n.**
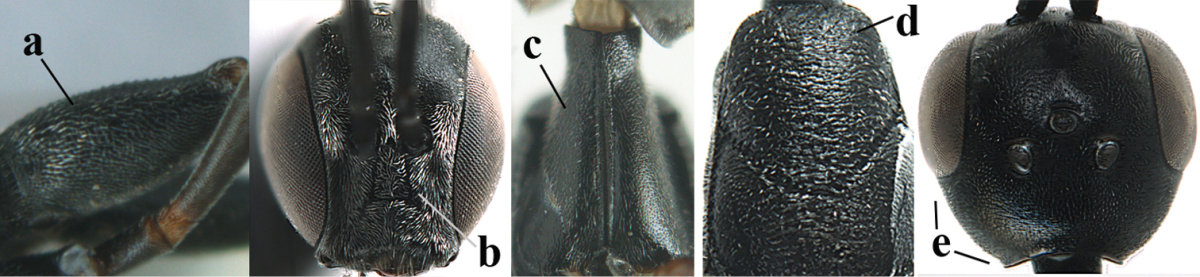		
–	Hind coxa distinctly rugose or rugulose (aa); face slightly wider (bb); propleuron in ventral view less slender (cc), punctures of middle lobe of mesoscutum as far as differentiated connected to rugae (dd); head in dorsal view often subparallel-sided (ee)	***Gasteruption phragmiticola* Saure, 2006**
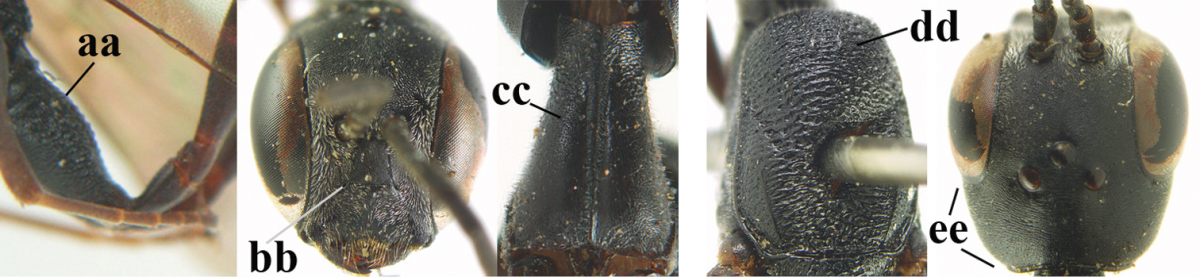		
15	Occipital carina wide to medium-sized medio-dorsally (a), basally thick, more or less aciculate or crenulate (b) and black or dark brown (c); if intermediate than head distinctly concave medio-posteriorly (d); head rather elongate trapezoid in dorsal view (e); [face wide]	**16**
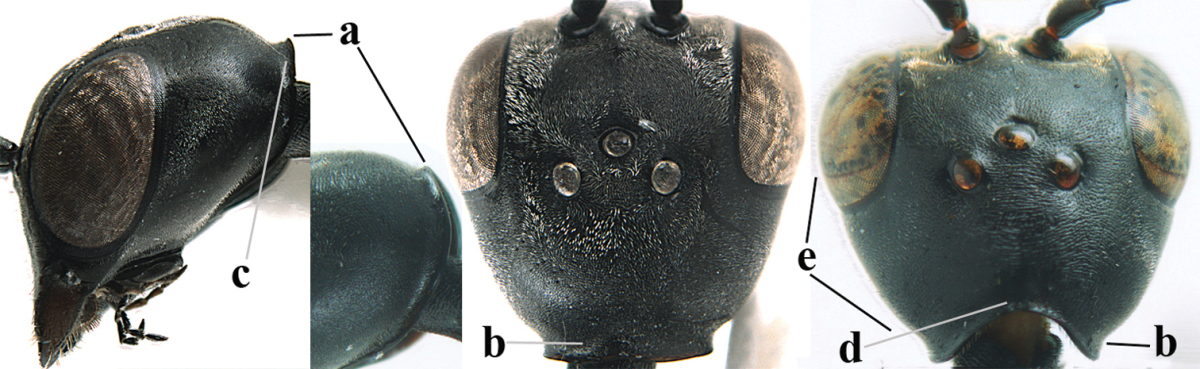		
–	Occipital carina usually narrower medio-dorsally (aa), **if** wide then basally thin, basally smooth (bb) and brown (cc); head at most slightly concave medio-posteriorly (dd) and shorter trapezoid or subglobular in dorsal view (ee)	**17**
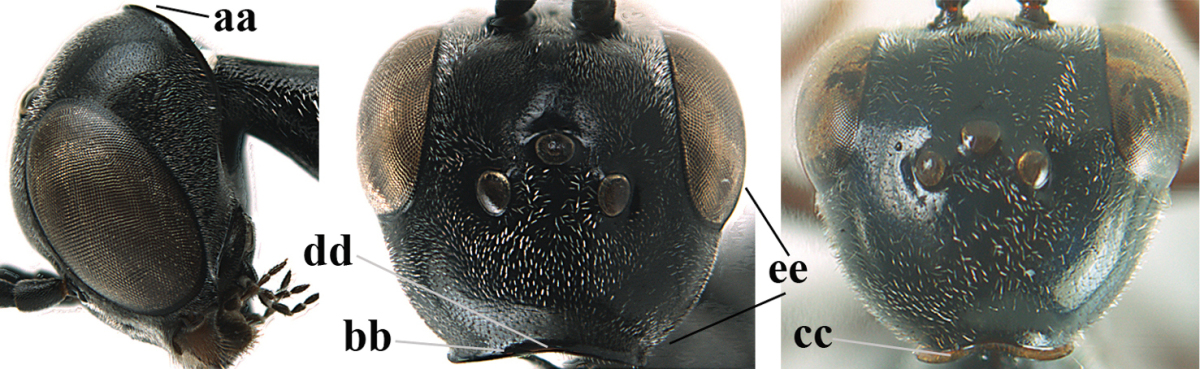		
16	Occipital carina widely collar-shaped (a), often partly brown and area in front of carina more or less aciculate or crenulate (b); length of ovipositor sheath 2.5–3.1 (but rarely up to 4.3) times as long as hind tibia and 0.8–0.9 (rarely up to 1.2) times as long as metasoma (c); apical half of hind tibia with pale yellowish setae and more or less reddish or yellowish brown (d); [stout species; lateral lobe of mesoscutum largely coarsely punctate to reticulate; mandible often yellowish or orange brown basally, but sometimes dark brown; fifth and sixth sternites yellowish brown or dark brown]	***Gasteruption insidiosum* Semenov, 1892**
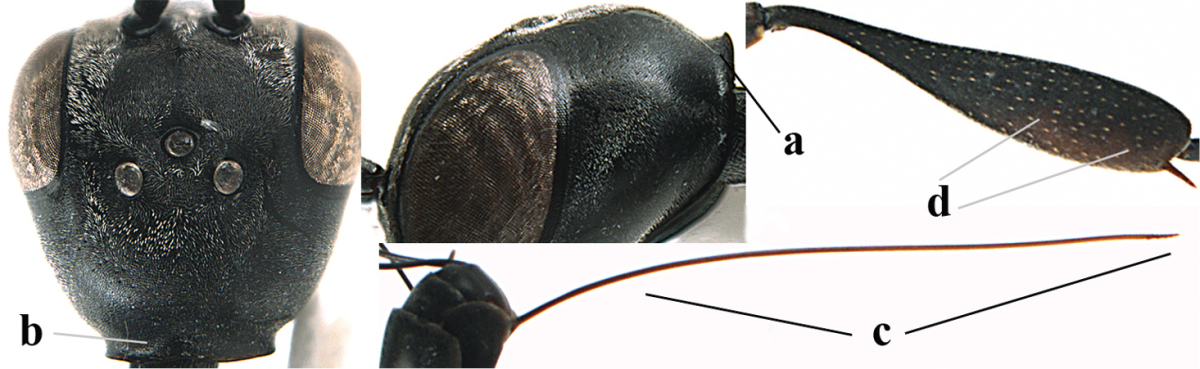		
–	Occipital carina at most medium-sized, entirely black and not collar-shaped (aa) and area in front of carina usually smooth or nearly so (bb); length of ovipositor sheath 3.4–4.7 times as long as hind tibia and 1.1–1.6 times as long as metasoma (cc); apical half of hind tibia with brown setae (rarely yellowish) and outer side black or dark brown (dd); [rather slender species]	***Gasteruption nigrescens* Schletterer, 1885**
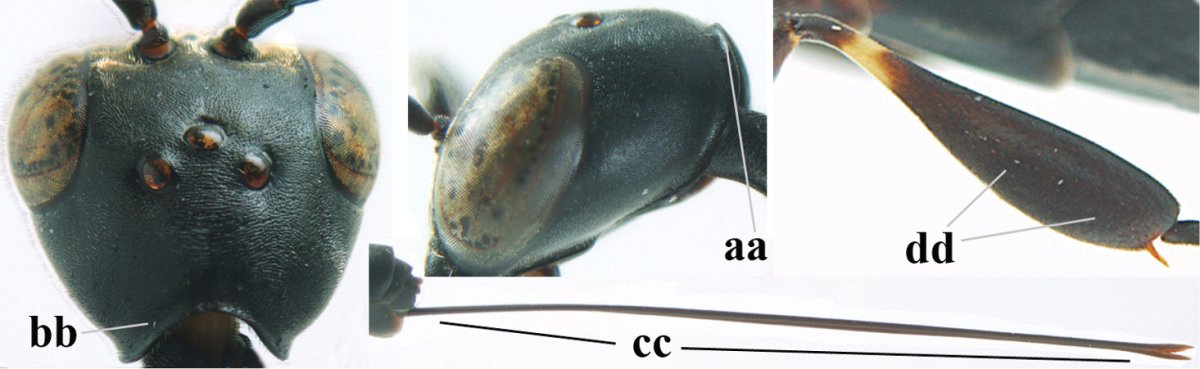		
17	Mesoscutum largely very finely coriaceous and at most with superficial punctures (a)	**18**
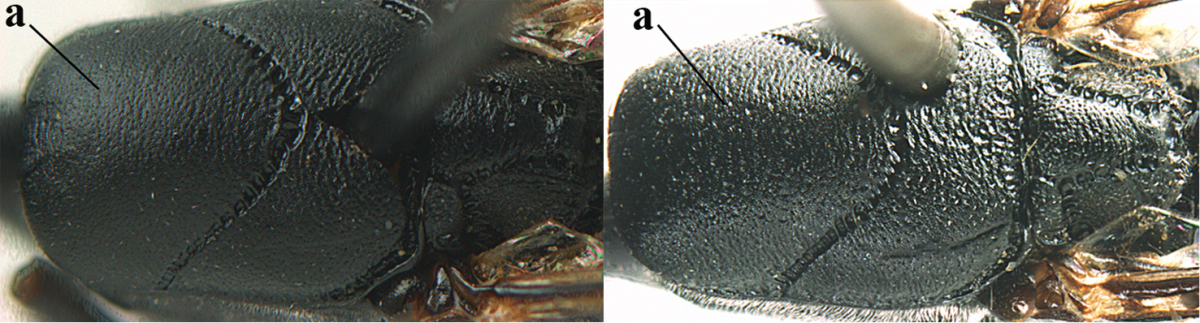		
–	Mesoscutum reticulate or with distinct punctures, rugulae or rugae (aa)	**19**
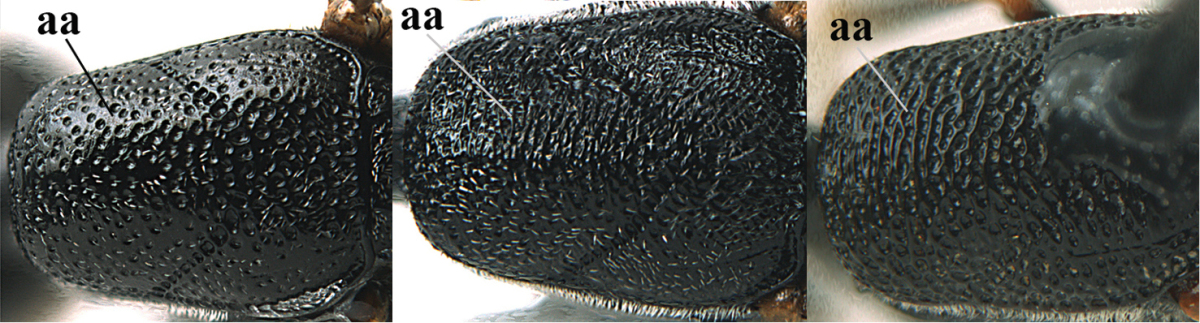		
18	Occipital carina wide medio-dorsally (a); apex of ovipositor sheath dark brown (b); mesoscutum only coriaceous (c); [ovipositor sheath 4.5–5.0 times as long as hind tibia and about as long as body; subbasally hind tibia dark brown dorsally]	***Gasteruption scorteum* sp. n.**
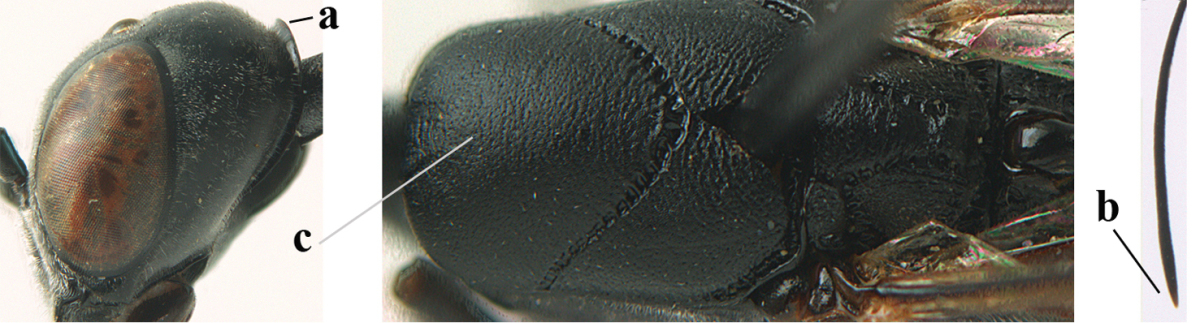		
–	Occipital carina rather narrow medio-dorsally (aa); apex of ovipositor sheath distinctly white or ivory (bb); mesoscutum with some fine punctures (cc); [ovipositor sheath 3.6–3.8 times as long as hind tibia and 0.7 times as long as body; lateral lobe of mesoscutum and scutellum finely and densely rugulose or rugose; vertex slightly depressed medio-posteriorly; mandible black or dark brown basally]	***Gasteruption ischnolaimum* sp. n.**
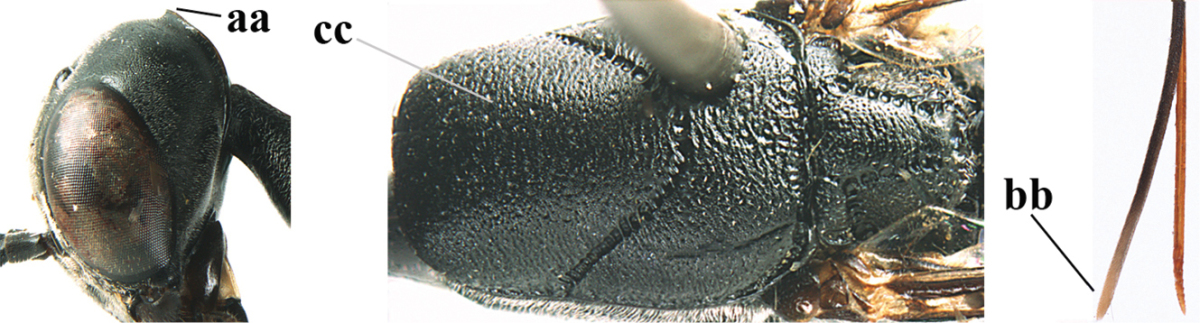		
19	Mesoscutum coarsely reticulate and shiny (a); pronotal side shiny and partly or entirely coarsely punctate or reticulate antero-ventrally (b); mandible dark yellowish or reddish brown (c)	**20**
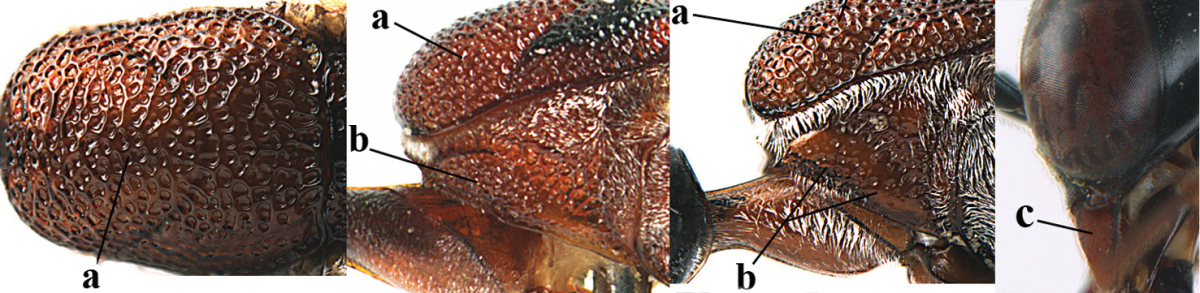		
–	Mesoscutum punctate, rugose, punctulate or coriaceous and matt or with satin sheen (aa); pronotal side with satin sheen and rugulose or coriaceous antero-ventrally (bb); colour of mandible variable, blackish (cc) to yellowish brown	**21**
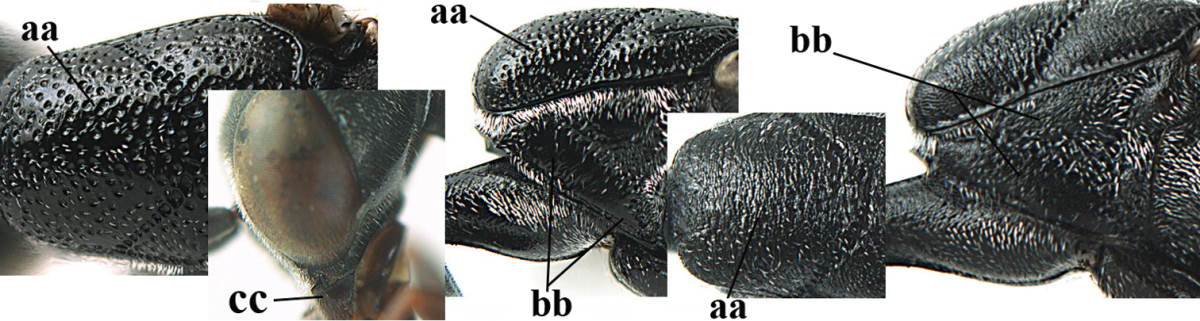		
20	Apex of ovipositor sheath pale brown or ivory (a; 0.2–0.5 times as long as hind basitarsus); ovipositor sheath 5.0–5.7 times as long as hind tibia (b); antesternal carina narrow medio-ventrally (c); incision of hypopygium deeper, up to apical 0.4–0.5 (d); occipital carina narrow medio-dorsally (e); [fifth sternite of female orange brown; antenna 1.3–1.4 times as long as hind tibia; mesosoma entirely black or up to anterior half largely orange-brown]	***Gasteruption agrenum* sp. n.**
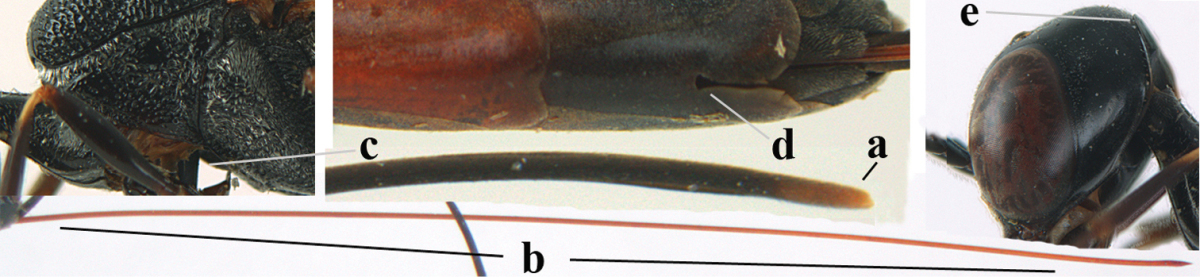		
–	Apex of ovipositor sheath black or dark brown (aa); ovipositor sheath 2.5–3.7 times as long as hind tibia (bb); antesternal carina medium-sized medio-ventrally (dd); incision of hypopygium shallower, up to apical 0.3–0.4 (ee); occipital carina medium-sized medio-dorsally (ff); [fifth sternite black or dark brown (cc)]	***Gasteruption merceti* Kieffer, 1904**
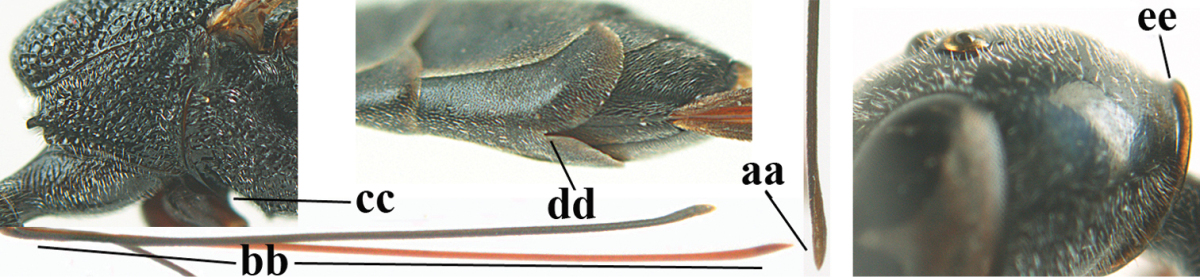		
21	Mesoscutum laterally with smooth and shiny interspaces between punctures (a); apex of ovipositor sheath black (b); [pronotal side smooth ventrally; three basal hind tarsal segments black dorsally but sometimes partly ivory]	***Gasteruption heminitidum* sp. n.**
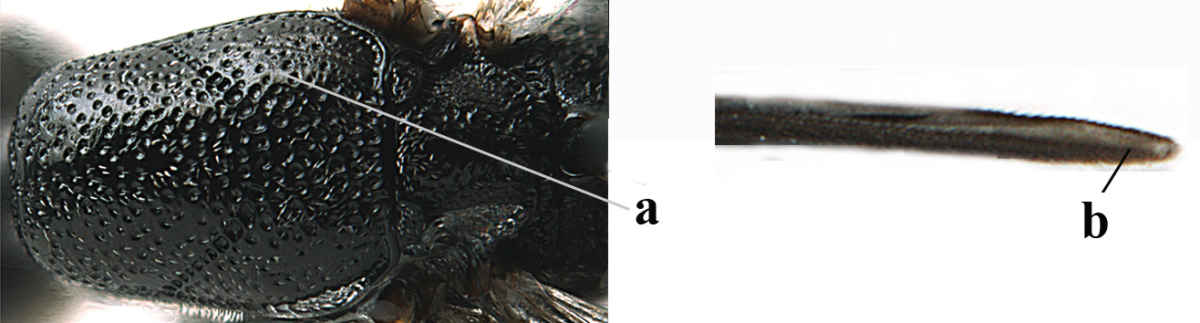		
–	Mesoscutum densely sculptured, without separate punctures or with distinct interspaces and with satin sheen (aa); apex of ovipositor sheath dark brown, pale brown or black, rarely ivory (bb)	**22**
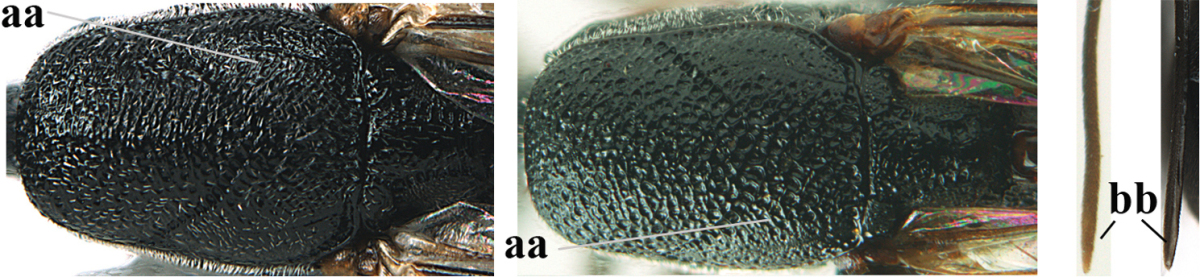		
22	Vertex anteriorly punctulate and without additional small punctures (a); scutellum shiny medially (b); lateral lobe of mesoscutum with oblique rugae anteriorly (c); mesoscutum regularly transversely rugose (d); hind tibial spurs much paler than dark brown hind basitarsus (e)	***Gasteruption nigrapiculatum* sp. n.**
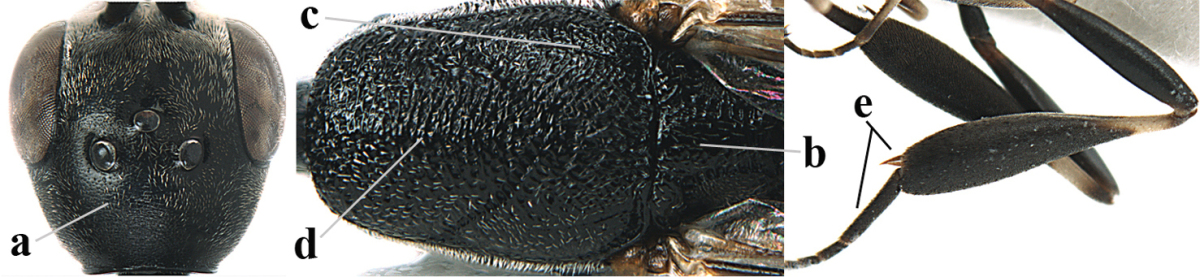		
–	Vertex anteriorly punctulate or punctulate-aciculate and with some small punctures (aa); scutellum at most with satin sheen medially (bb); lateral lobe of mesoscutum more or less irregularly punctate-rugose, without distinct oblique rugae anteriorly (dd); mesoscutum more or less irregularly rugose or crater-like punctate (ee); colour of hind tibial spurs variable, often about as dark as dark base of hind basitarsus (ff)	***Gasteruption schmideggeri* sp. n.**
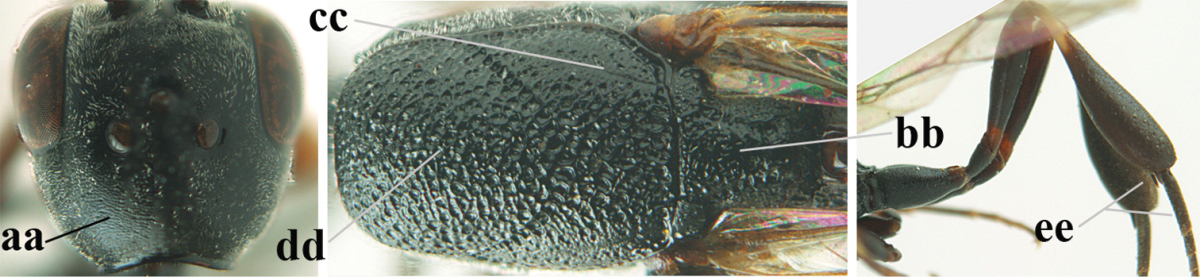		
23	Vertex with a deep medial depression in front of occipital carina (a) **and** in lateral view angulate in front of depression (b); mandible black or dark brown basally (c)	**24**
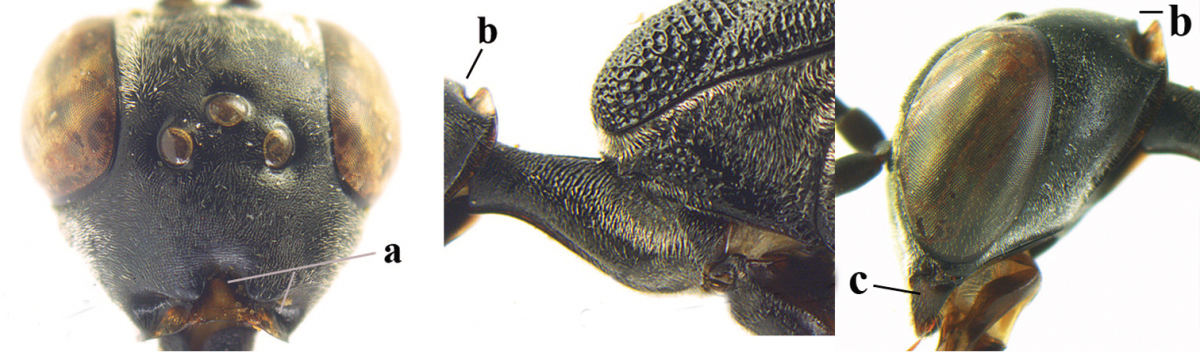		
–	Vertex without depression (aa) or with shallow depression, **if** with deep round medial depression in front of occipital carina (aaa; *Gasteruption pseudolaticeps* and *laticeps*), then in lateral view its border obtuse dorsally (bb) and mandible usually pale yellowish brown basally (cc)	**25**
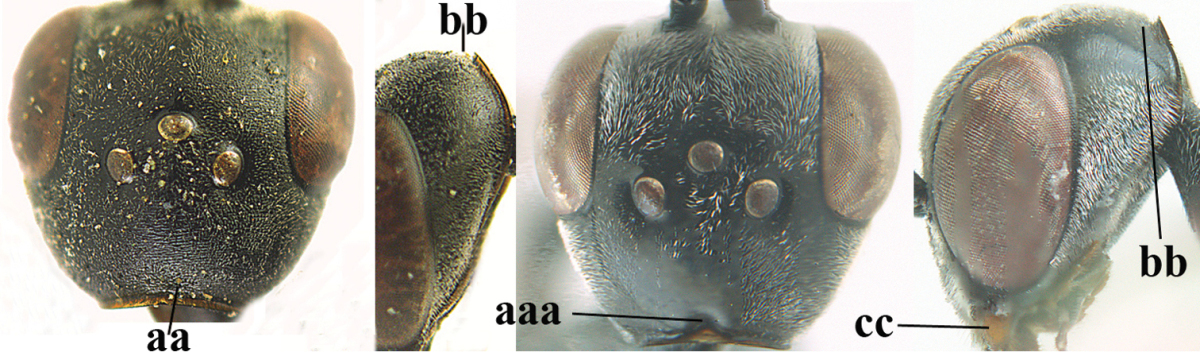		
24	Antesternal carina distinctly lamelliform and wide (a); head in dorsal view linearly narrowed behind eyes (b) and densely sculptured, with satin sheen (c); hind basitarsus partly white (d); [scutellum with oblique rugae antero-laterally]	***Gasteruption caucasicum* (Guérin-Méneville, 1844)**
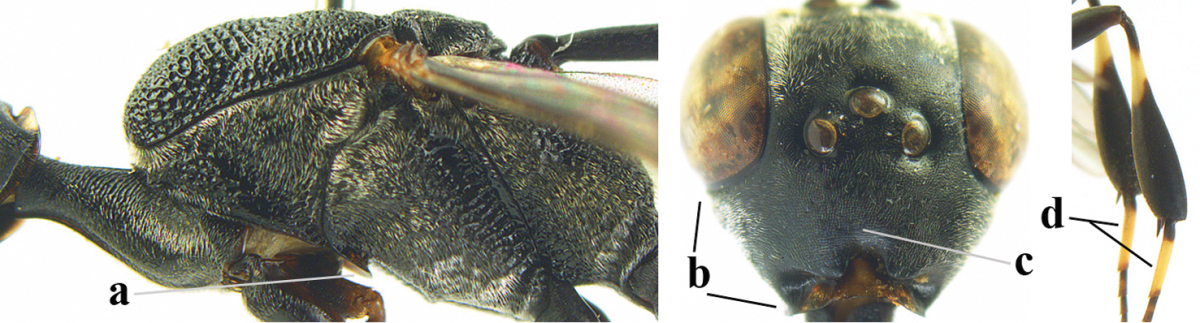		
–	Antesternal carina non-lamelliform and narrow, not elevated above mesosternum (aa); head in dorsal view gradually narrowed (bb) and sparsely sculptured, distinctly shiny (cc); hind basitarsus dark brown (dd); [length of body 15–22 mm]	***Gasteruption goberti* (Tournier, 1877)**
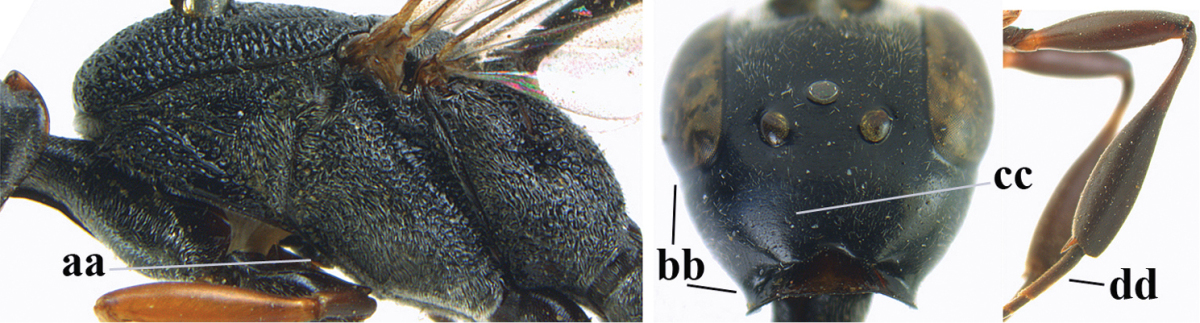		
25	Antesternal carina wide to medium-sized (distinctly wider than prepectal carina) and lamelliform, curved up with fore coxa distinctly removed from mesosternum (a; intermediate in *Gasteruption schlettereri*, but included in both alternatives); medio-posteriorly mesoscutum coarsely punctate-rugose or reticulate (b)	**26**
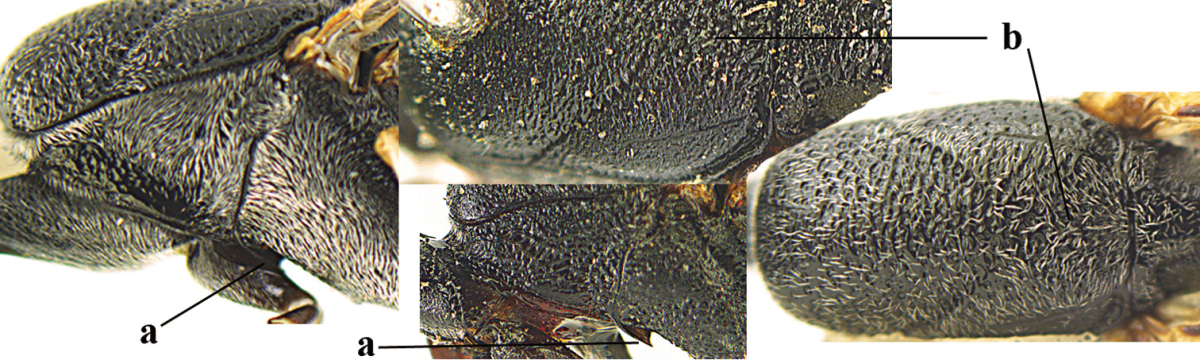		
–	Antesternal carina non-lamelliform, narrow (similar to prepectal carina) and fore coxa close to mesosternum (aa); medio-posteriorly mesoscutum with separate punctures or transversely rugose (bb)	**30**
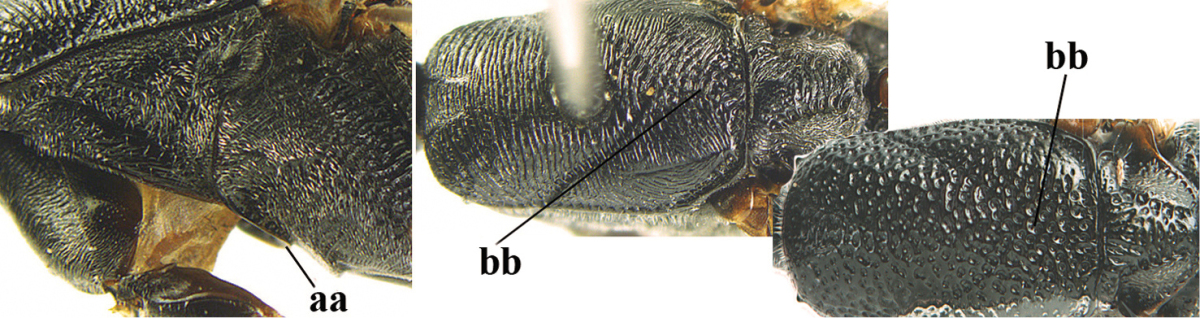		
26	Vertex with distinct depression medio-posteriorly (a), rarely superficially impressed; mesoscutum and mesosoma laterally often conspicuously setose, partly obscuring sculpture (b)	**27**
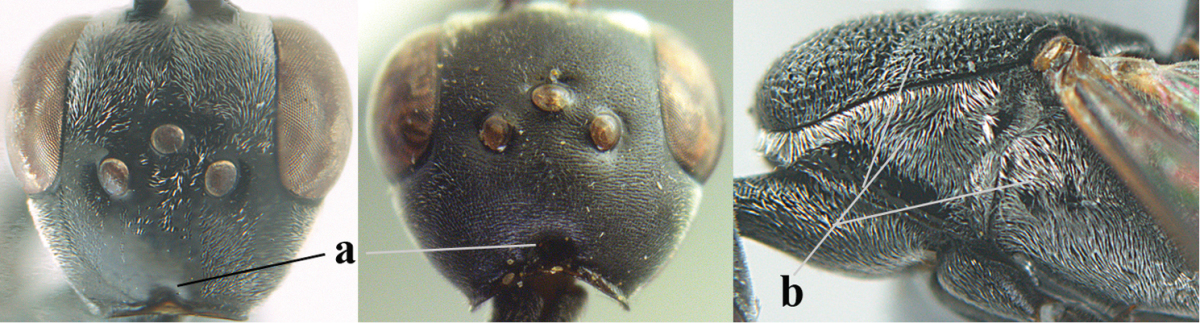		
–	Vertex without depression medio-posteriorly (aa), but sometimes slightly impressed in *Gasteruption diversipes*; mesoscutum and mesosoma laterally less conspicuously setose, less obscuring sculpture (bb); [head dorsally distinctly sculptured and with satin sheen]	**28**
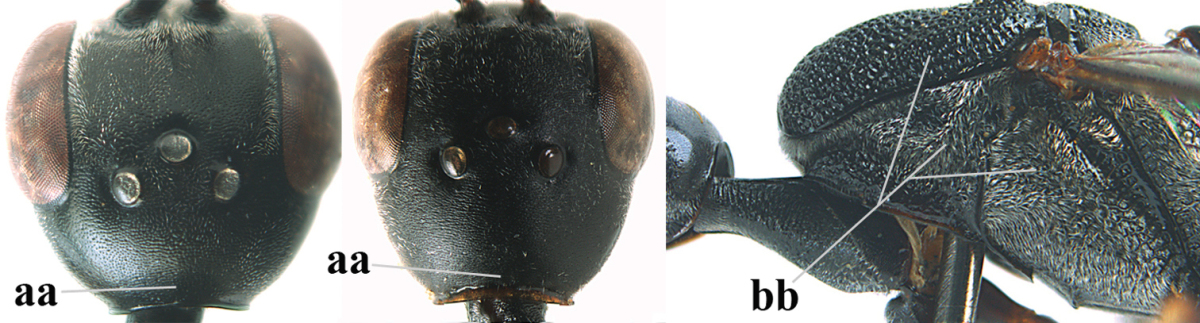		
27	Head dorsally only superficially sculptured and shiny (a); antesternal carina moderately curved up (b); mesoscutum and mesosoma laterally conspicuously setose, partly obscuring sculpture (c; but sometimes secondarily reduced); pronotal side usually more elongate (d); [occipital carina moderately wide and distinctly lamelliform medio-dorsally; medio-posterior depression of vertex rather deep, rarely superficially impressed]	***Gasteruption pseudolaticeps* sp. n.**
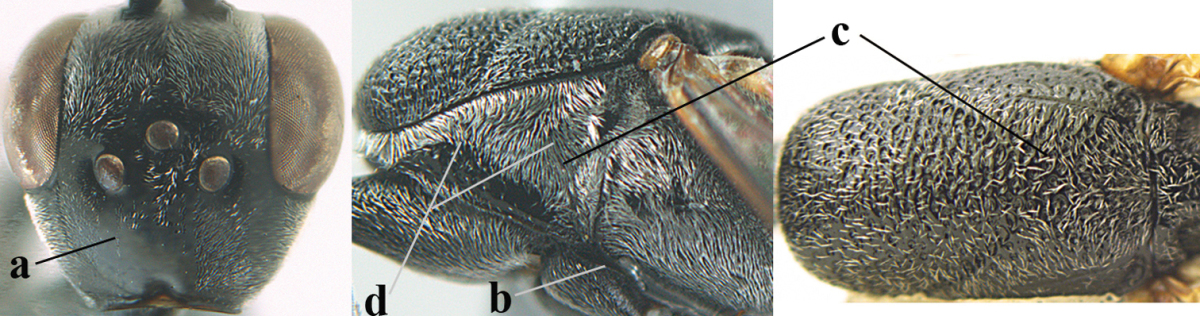		
–	Head dorsally densely micro-sculptured and with satin sheen (aa); antesternal carina strongly curved up (bb); mesoscutum and mesosoma laterally less setose, less or not obscuring sculpture (cc); pronotal side less elongate (dd)	***Gasteruption laticeps* (Tournier, 1877)**
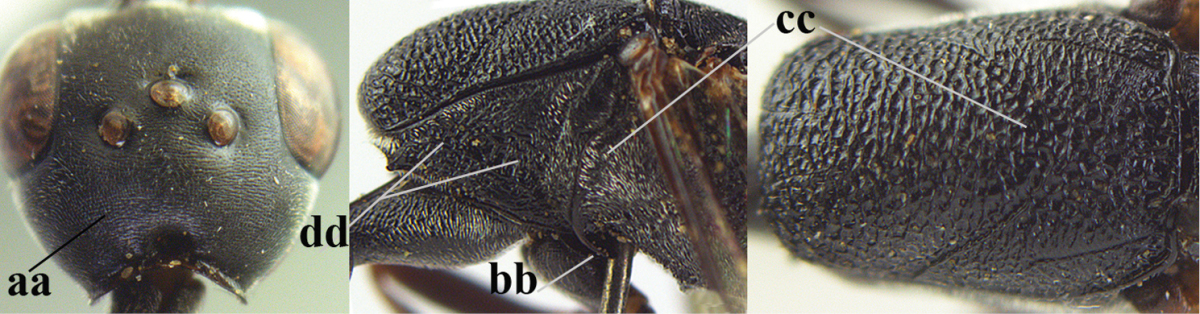		
28	Propleuron narrower and 0.9–1.1 times as long as distance between tegulae and anterior border of mesoscutum (a); mesoscutum antero-dorsally with more or less separate punctures; (b); hind basitarsus usually largely or completely dark brown or brown (c), but sometimes with distinct ivory band or dorsal patch; pronotal side more elongate (d)	***Gasteruption opacum* (Tournier, 1877)**
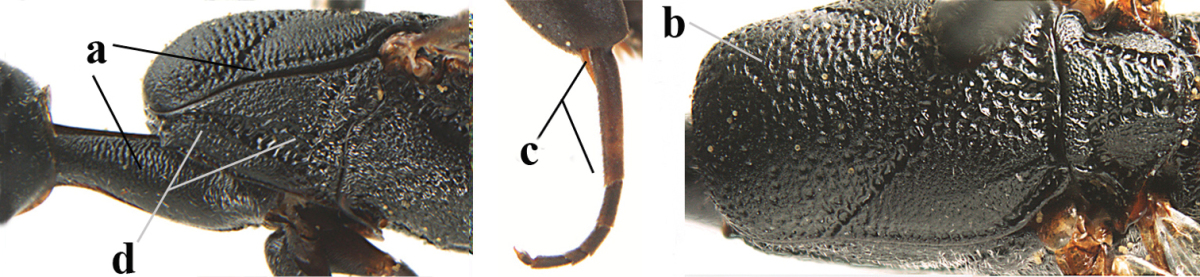		
–	Propleuron wider and 0.8–0.9 times distance between tegulae and anterior border of mesoscutum (aa); mesoscutum antero-dorsally rugose, without separate punctures (bb); hind basitarsus partly ivory or yellow (cc); pronotal side less elongate (dd)	**29**
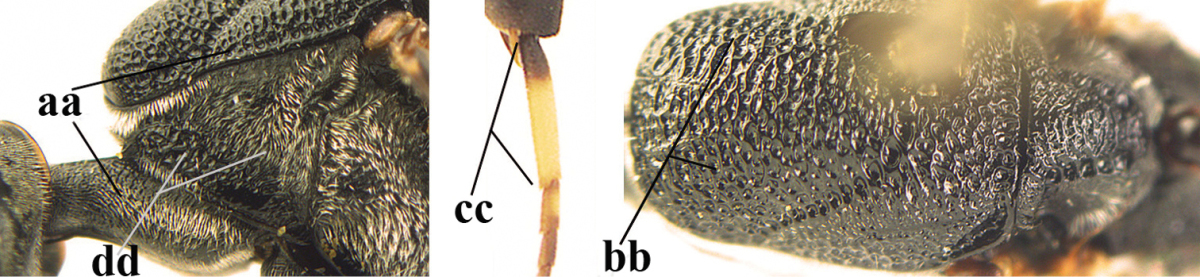		
29	Antesternal carina distinctly protruding (a); lateral lobe of mesoscutum reticulate-rugose (b); mandible more or less dark brown or infuscate basally (c)	***Gasteruption diversipes* (Abeille de Perrin, 1879)**
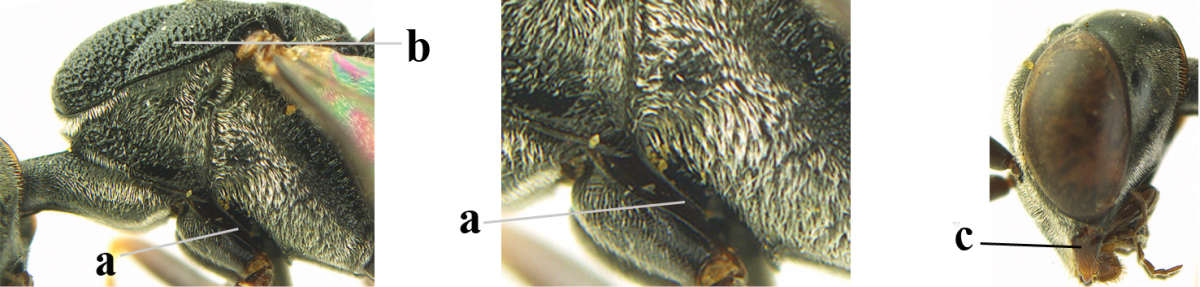		
–	Antesternal carina less protruding (aa); lateral lobe of mesoscutum with smooth and very shiny interspaces (bb); mandible yellow basally (cc; variable in male)	***Gasteruption schlettereri* Magretti, 1890**
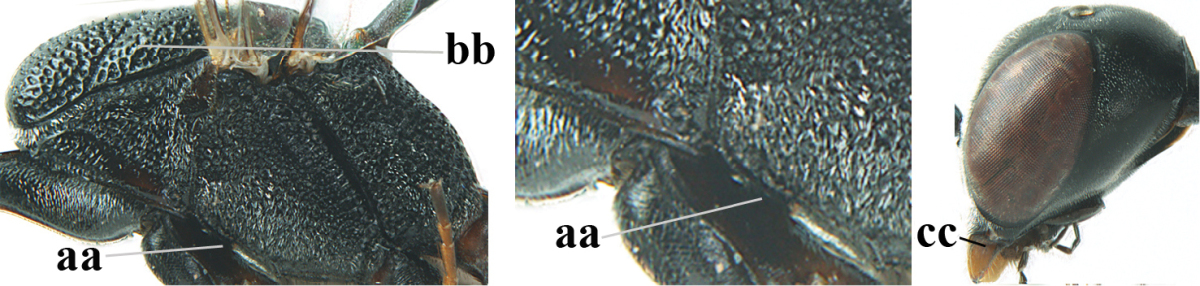		
30	Propleuron elongate and slender, about as long as length of mesoscutum in front of tegulae (a); pronotal side antero-ventrally mainly coriaceous (b); middle lobe of mesoscutum distinctly punctate and interspaces coriaceous (c)	***Gasteruption syriacum* Szépligeti, 1903**
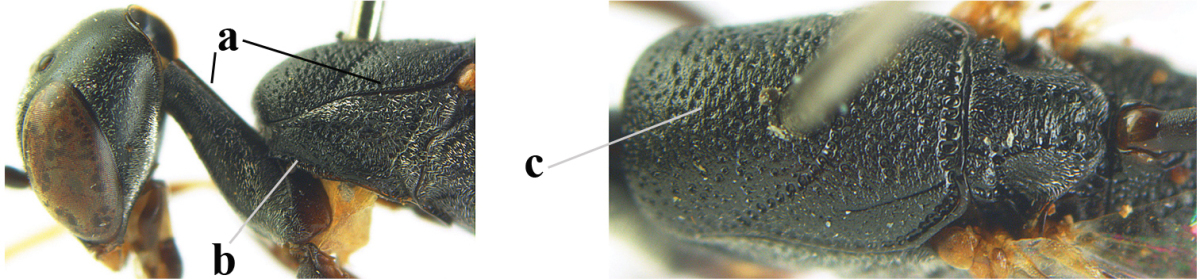		
–	Propleuron shorter and stout, shorter than length of mesoscutum in front of tegulae (aa); pronotal side antero-ventrally reticulate or punctate (bb); middle lobe of mesoscutum variable (cc)	**31**
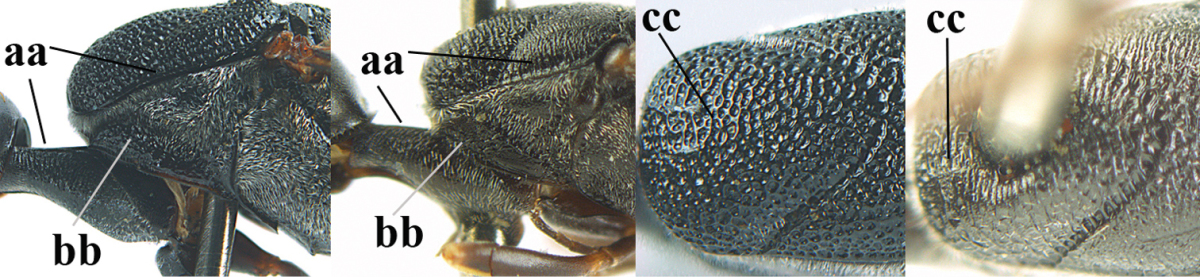		
31	Occipital carina wide and lamelliform and its medio-dorsal width 0.5–0.6 times diameter of posterior ocellus (a); mesoscutum largely finely transversely rugulose (b); ovipositor sheath 1.0–1.2 times as long as body and 1.4–1.7 times as long as metasoma (c)	**32**
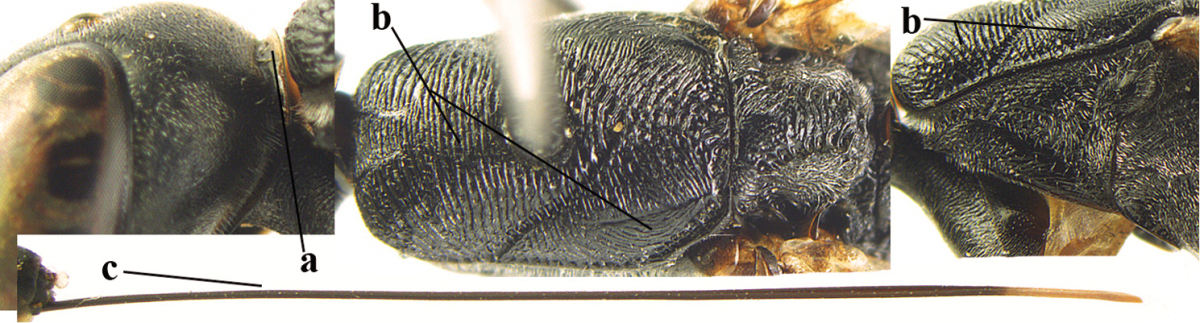		
–	Occipital carina narrow and non-lamelliform (aa) or medium-sized lamelliform and 0.2–0.3 times as wide as diameter of posterior ocellus; mesoscutum coarsely rugose-punctate or punctate-coriaceous (bb), punctate or rugulose; ovipositor sheath 0.8–1.0 times as long as body and 1.1–1.6 times as long as metasoma (cc)	**33**
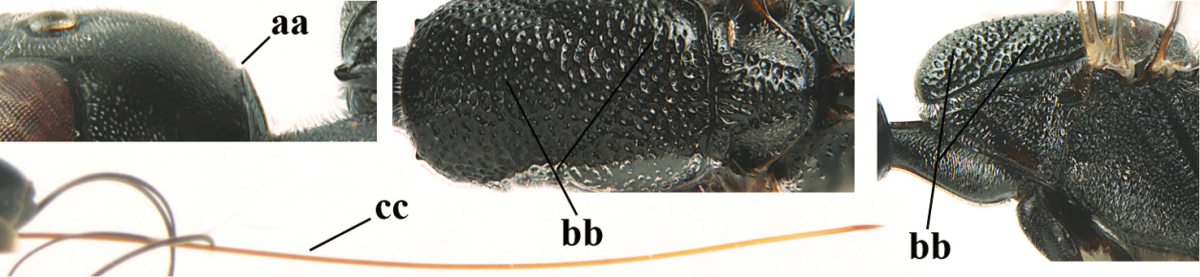		
32	Shallow medial depression in front of occipital carina absent (a); temples more gradually narrowed behind eyes (b); frons densely sculptured (c); mesoscutum largely regularly transversely rugose or rugulose (d); vertex evenly curved dorso-laterally in front of occipital carina (e)	***Gasteruption jaculator* (Linnaeus, 1758)**
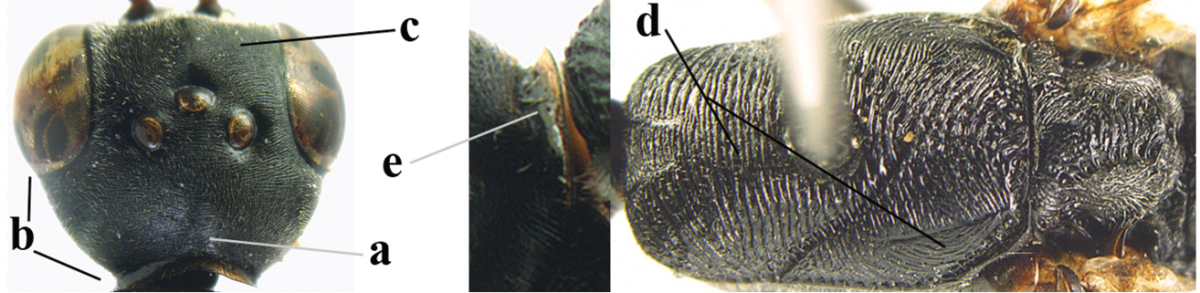		
–	Shallow medial depression in front of occipital carina present (aa), rarely obsolescent; temples linearly narrowed behind eyes (bb); frons sparsely sculptured (cc); mesoscutum irregularly transversely rugose or rugulose (dd); vertex with weak ridge dorso-laterally in front of occipital carina and more or less depressed below it (ee)	***Gasteruption tournieri* Schletterer, 1885**
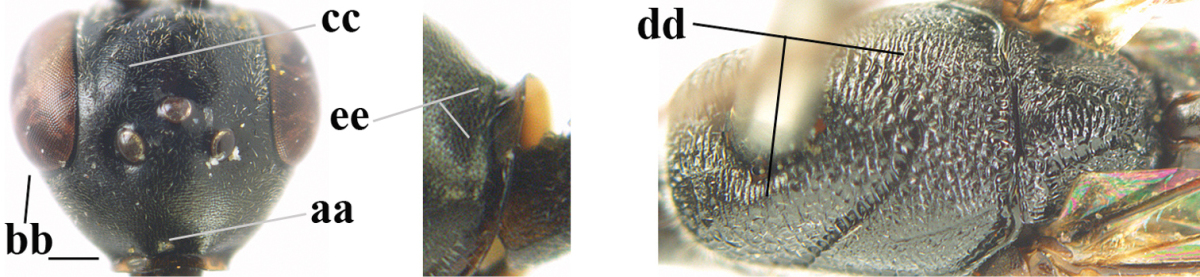		
33	Occipital carina medium-sized lamelliform, blackish and about 0.3 times as wide as diameter of posterior ocellus (a); pronotal side largely coriaceous and flattened (b); apical pale part of ovipositor sheath shorter than hind basitarsus (c)	***Gasteruption insidiosum* Semenov, 1892**
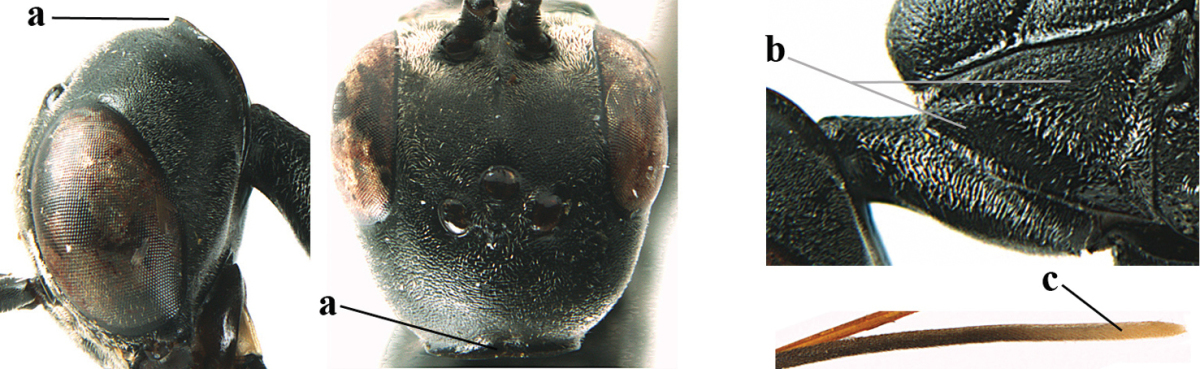		
–	Occipital carina non-lamelliform or nearly so (aa), **if** medium-sized lamelliform (aaa) then pronotal side largely coarsely reticulate-rugose (bb); apical pale part of ovipositor sheath 1.0–2.0 times as long as hind basitarsus (cc)	**34**
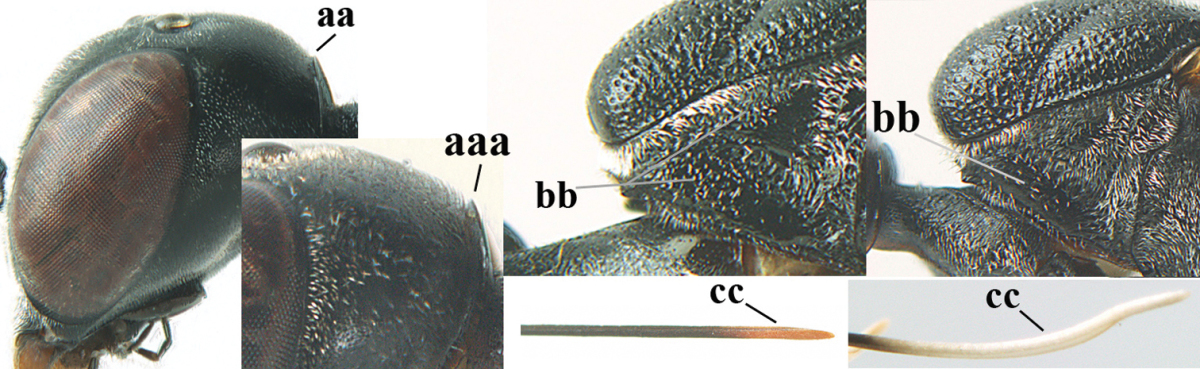		
34	Hind tibia abruptly swollen and distinctly curved dorso-laterally (a); hind basitarsus rather short and about 0.8 times as long as remainder of tarsus (b); pronotal side superficially coriaceous and with some coarse punctures ventrally (c); [hypopygium largely yellowish brown]	***Gasteruption henseni* sp. n.**
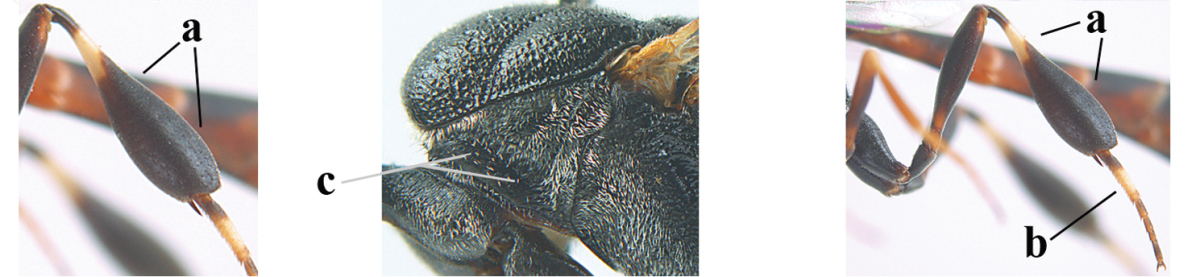		
–	Hind tibia gradually swollen and weakly curved dorso-laterally (aa); hind basitarsus medium-sized and about as long as remainder of tarsus or longer (bb); pronotal side more or less reticulate, rugulose or punctate ventrally (cc)	**35**
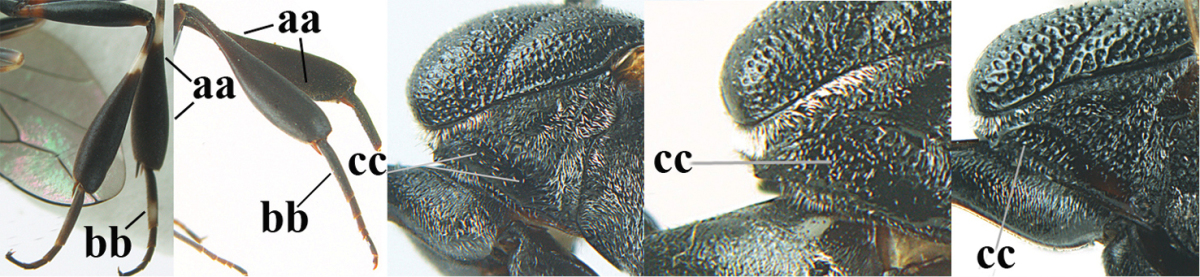		
35	Mesoscutum finely transversely rugulose medio-posteriorly (a); pronotal side largely rugulose (b); vertex finely transversely rugulose (c); head in anterior view distinctly developed below lower level of eyes (d); hind femur slender and hardly widened submedially (e); [area in front of occipital carina superficially depressed medio-posteriorly]	***Gasteruption lugubre* Schletterer, 1889, stat. rev.**
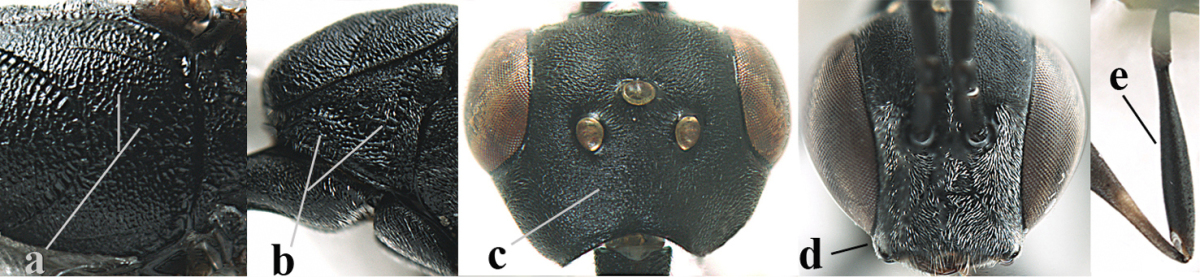		
–	Mesoscutum punctate, coarsely rugose or reticulate medio-posteriorly (aa); pronotal side partly or entirely reticulate-rugose ventrally (bb); vertex punctulate (cc); head in anterior view slightly developed below lower level of eyes (dd); hind femur less slender and widened submedially (ee)	**36**
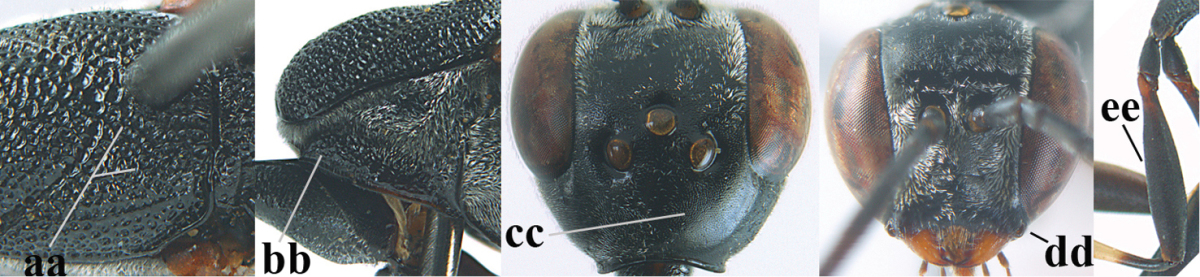		
36	Lateral lobe of mesoscutum transversely aciculate-rugulose (a); frons finely and densely aciculate-coriaceous (b); vertex flattened in lateral view (c); propleuron stout (d)	***Gasteruption aciculatum* sp. n.**
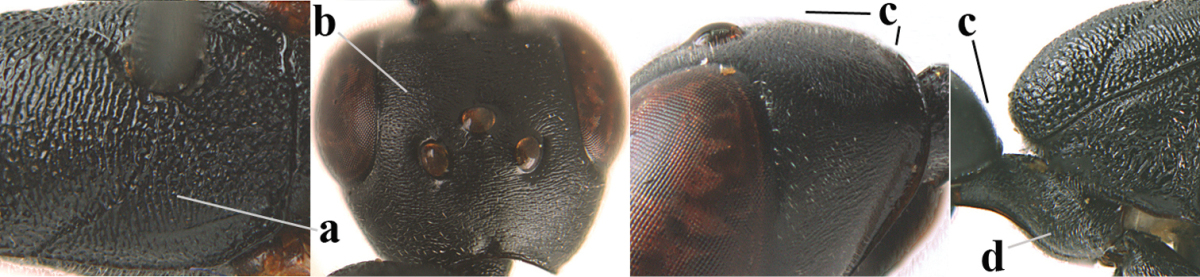		
–	Lateral lobe of mesoscutum reticulate, partly punctate or mainly coriaceous (aa); frons densely and finely rugulose-punctulate or very finely punctulate (bb); vertex convex in lateral view (cc); propleuron slenderer (dd)	**37**
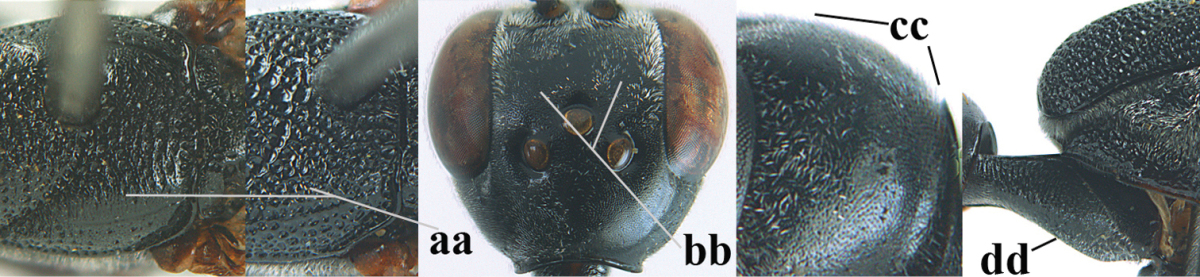		
37	Lateral lobe of mesoscutum reticulate and shiny (a); frons densely and finely rugulose-punctulate and without distinct punctures (b, rarely with some fine punctures); pronotal side only antero-ventrally reticulate-rugose (c); hind basitarsus usually bicoloured (d)	***Gasteruption schlettereri* Magretti, 1890**
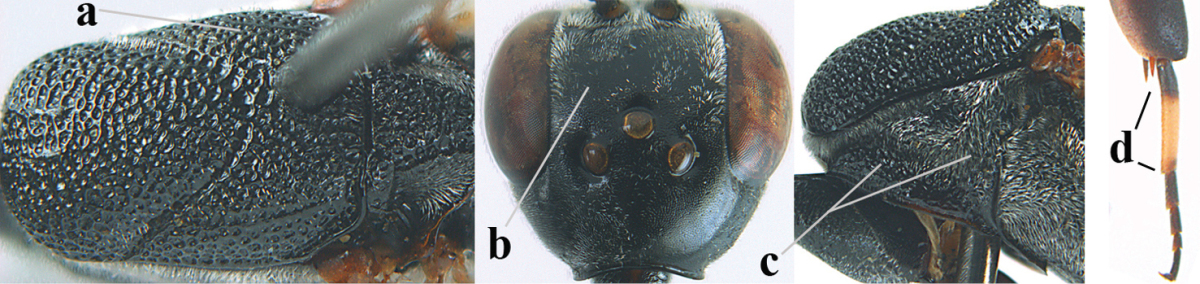		
–	Lateral lobe of mesoscutum partly punctate, coriaceous or rugulose and with satin sheen (aa); frons very finely punctulate and often mixed with punctures (bb); pronotal side largely reticulate-rugose (cc) or rugulose; hind basitarsus often tricoloured (dd) or unicoloured (ddd)	**38**
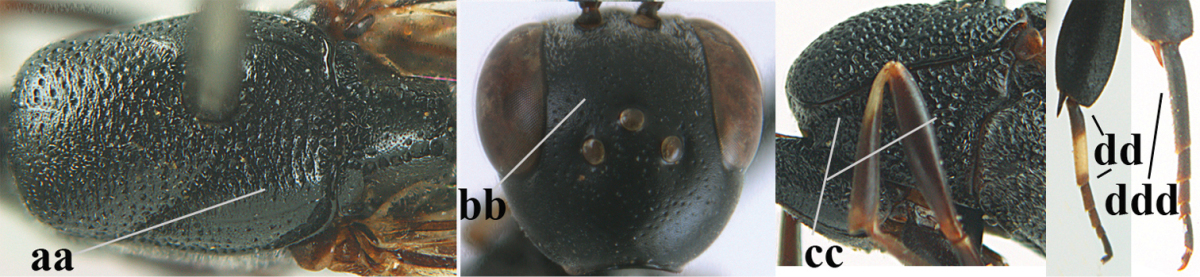		
38	Lateral lobe of mesoscutum coarsely punctate (a); pronotal side coarsely reticulate-rugose ventrally (b); punctulation of frons and vertex usually mixed with medium-sized and widely separate punctures (c), but frequently punctures obsolete; hind coxa coarsely rugose or reticulate dorsally (d); ivory part of ovipositor sheath 1.8–2.6 times as long as hind basitarsus (e); [mesosoma black or reddish brown]	***Gasteruption punctifrons* sp. n.**
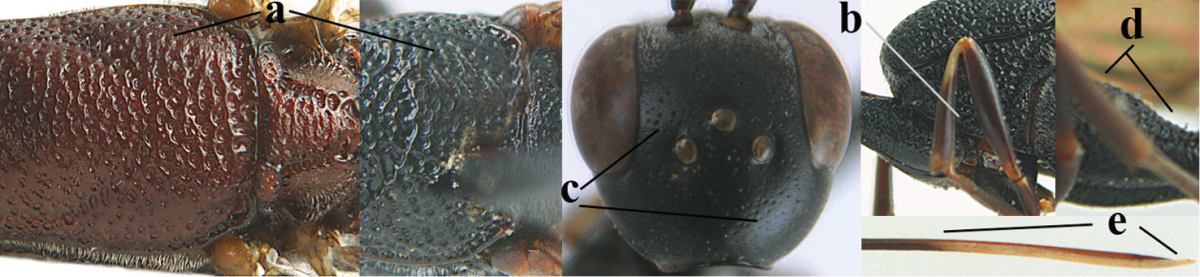		
–	Lateral lobe of mesoscutum moderately punctate (aa); pronotal side rugulose ventrally (bb); punctulation of frons and vertex without distinct punctures (cc); hind coxa rugulose dorsally (dd); ivory part of ovipositor sheath 1.0–1.4 times as long as hind basitarsus (ee); [mesosoma black]	***Gasteruption smitorum* sp. n.**
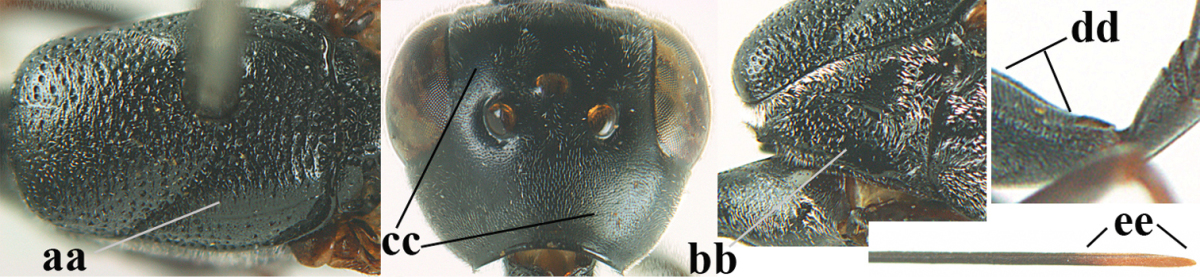		
**Males**		
39	Vertex with a deep medial depression in front of occipital carina (a) **and** in lateral view angulate in front of depression (b); mandible black or dark brown basally (c)	**40**
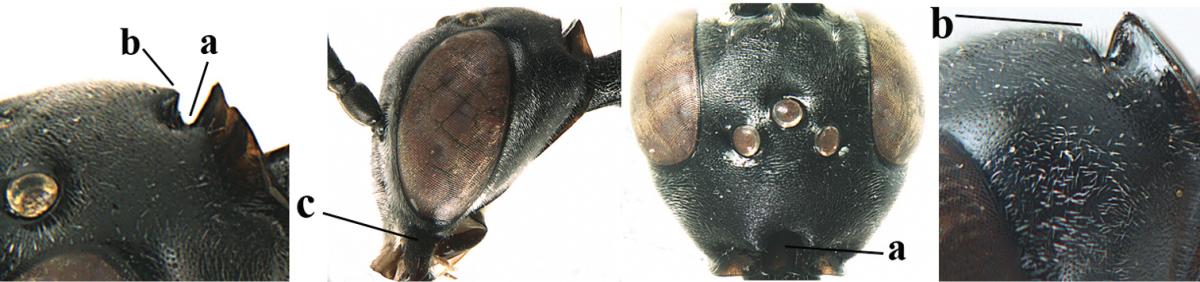		
–	Vertex without depression (aa) or with shallow depression, **if** with deep round medial depression in front of occipital carina (aaa), then in lateral view its border obtuse dorsally (bb) and mandible pale yellowish brown basally (cc)	**41**
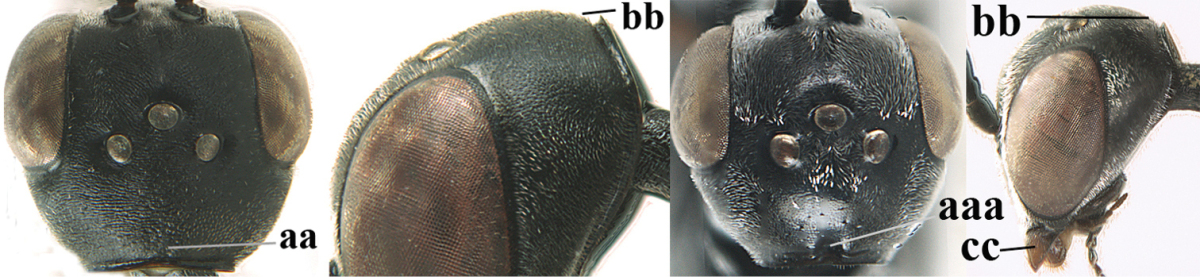		
40	Antesternal carina distinctly lamelliform and wide (a); head in dorsal view linearly narrowed behind eyes (b) and densely sculptured, with satin sheen (c); [scutellum with oblique rugae antero-laterally]	***Gasteruption caucasicum* (Guérin-Méneville, 1844)**
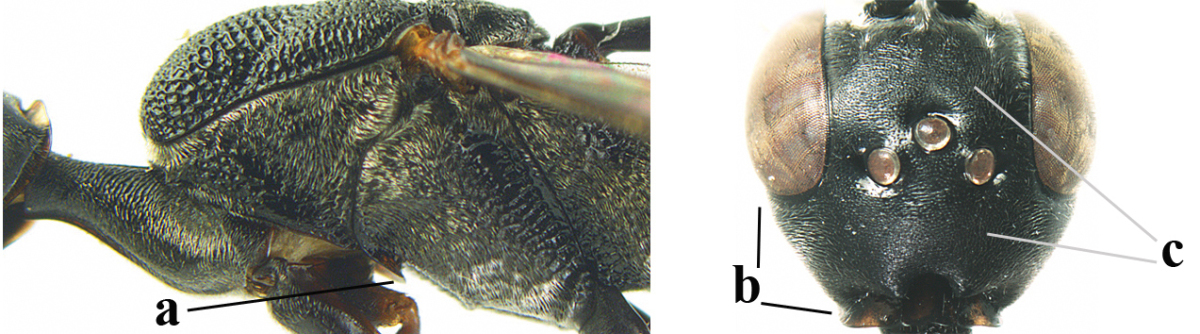		
–	Antesternal carina non-lamelliform or narrowly lamelliform and narrow, not elevated above mesosternum (aa); head in dorsal view gradually narrowed (bb) and sparsely sculptured, distinctly shiny (cc); [length of body 13–22 mm]	***Gasteruption goberti* (Tournier, 1877)**
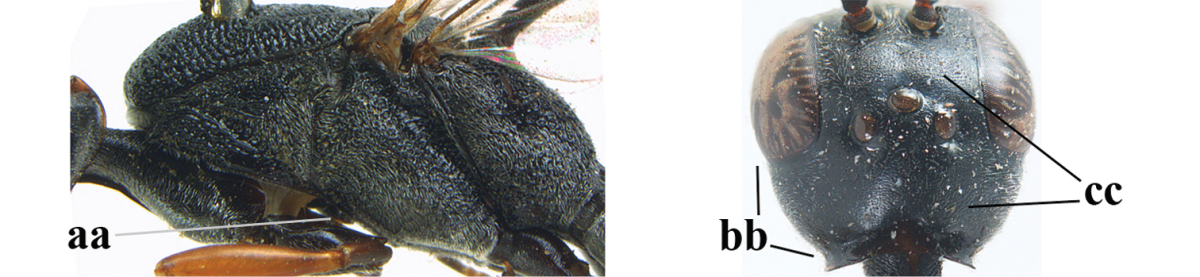		
41	Occipital carina wide medio-dorsally, 0.5–0.6 times diameter of posterior ocellus (a); dorsally pronotal side largely coriaceous (b)	**42**
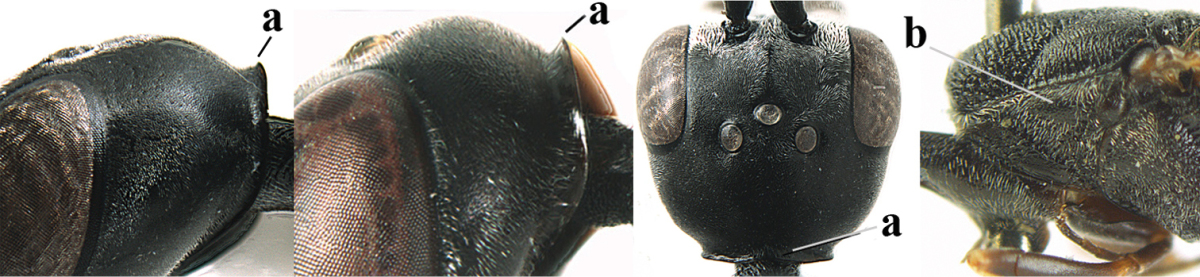		
–	Occipital carina narrow (aa) or medium-sized (aaa, up to 0.4 times diameter of posterior ocellus) medio-dorsally; sculpture of pronotal side variable dorsally (bb, bbb)	**44**
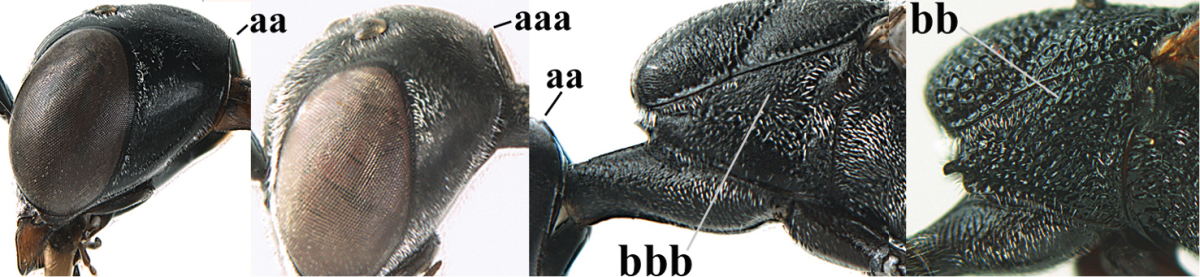		
42	Occipital carina basally thick and pointing posteriorly (a); head less narrowed in dorsal view (b); second and third antennal segments somewhat longer (c)	***Gasteruption insidiosum* Semenov, 1892**
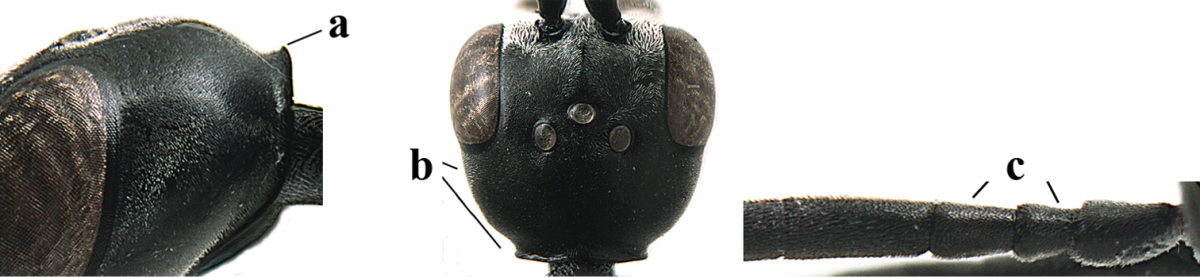		
–	Occipital carina basally thin and pointing dorsally or nearly so (aa); head more narrowed in dorsal view (bb); second and third antennal segments somewhat shorter (cc)	**43**
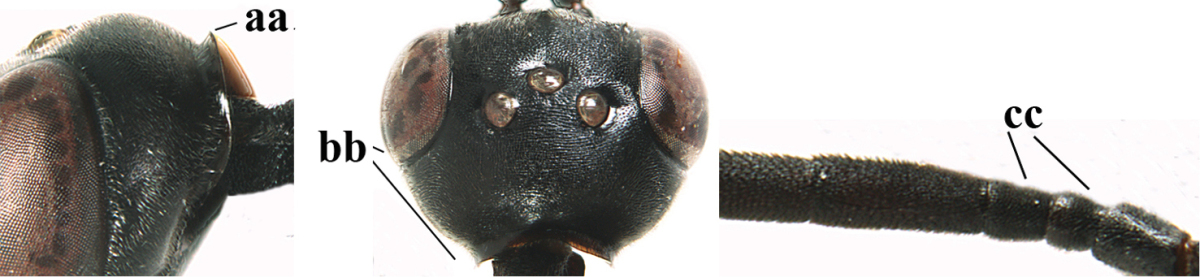		
43	Frons densely punctulate (a); temples more gradually narrowed behind eyes (b); shallow medial depression in front of occipital carina absent (c); mesoscutum more regularly transversely rugose or rugulose (d)	***Gasteruption jaculator* (Linnaeus, 1758)**
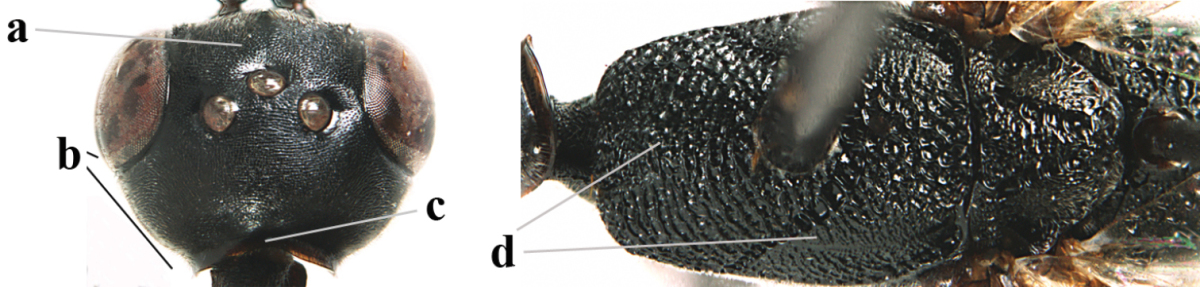		
–	Frons sparsely sculptured or mainly smooth (aa); temples linearly narrowed behind eyes (bb); shallow medial depression in front of occipital carina present (cc), but frequently obsolescent; mesoscutum irregularly transversely rugose or rugulose (dd)	***Gasteruption tournieri* Schletterer, 1885**
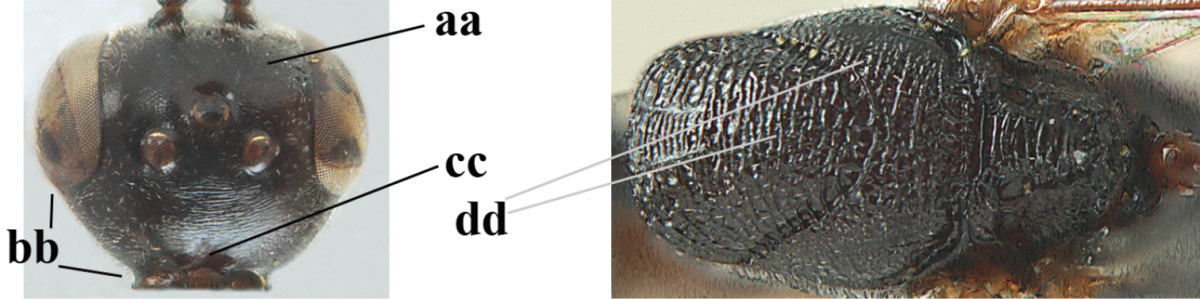		
44	Vertex distinctly bulging above dorsal level of occipital carina (a); hind basitarsus stout (b)	***Gasteruption variolosum* (Abeille de Perrin, 1879)**
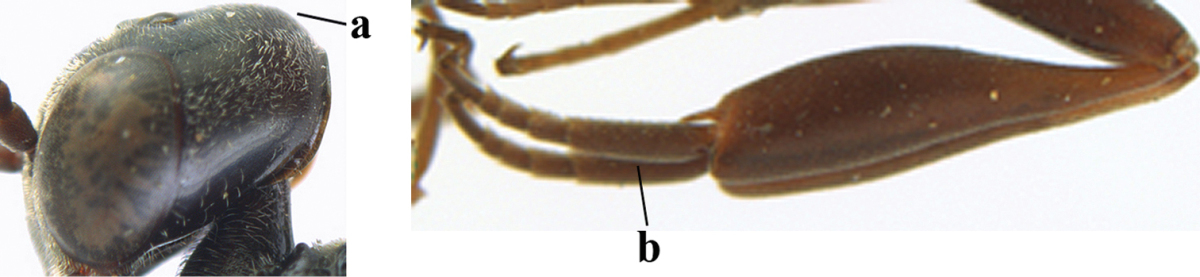		
–	Vertex moderately convex or flat and closer to dorsal level of occipital carina (aa); hind basitarsus usually slenderer (bb)	**45**
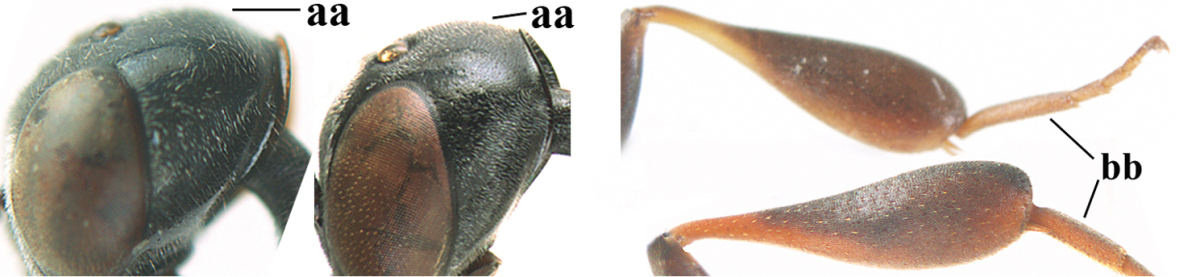		
45	Vertex flattened and elongate in lateral view (a) **and** propleuron 1.0–1.3 times distance from tegulae to anterior border of mesoscutum (b) and elongate in ventral view (c); head in anterior view distinctly elongate (d)	***Gasteruption dolichoderum* Schletterer, 1889**
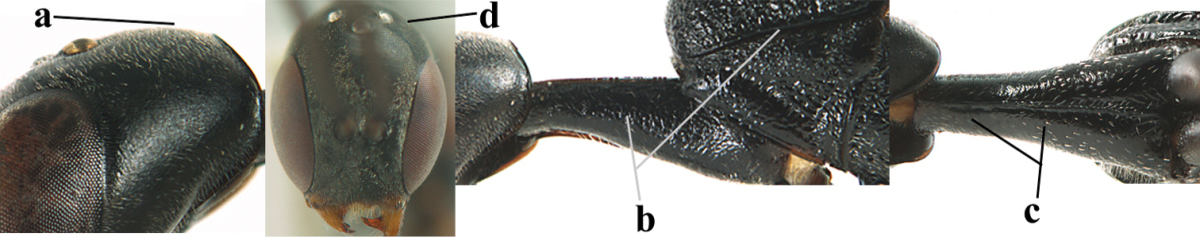		
–	Vertex more or less convex and shorter in lateral view (aa), **if** flattened then propleuron 0.7–0.8 times distance from tegulae to anterior border of mesoscutum (bb) and less elongate in ventral view (cc); head in anterior view normal or slightly elongate (dd)	**46**
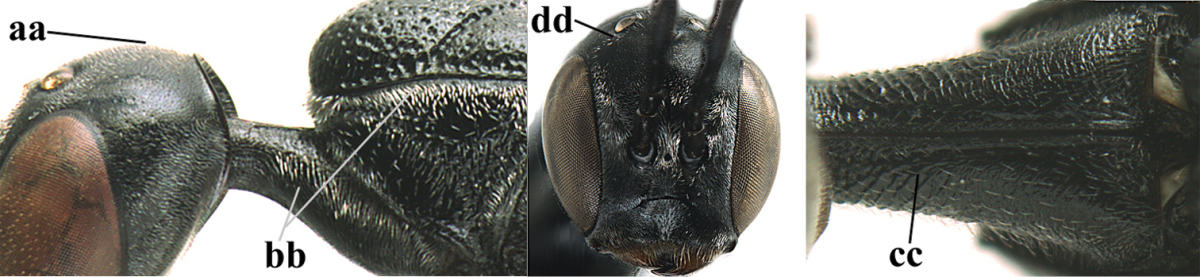		
46	Clypeus with rather large shallow depression (a); head and scapus more or less orange or reddish-brown (b), but sometimes head entirely black; hind basitarsus rather stout (c); [mesoscutum reticulate or rugose]	***Gasteruption hastator* (Fabricius, 1804)**
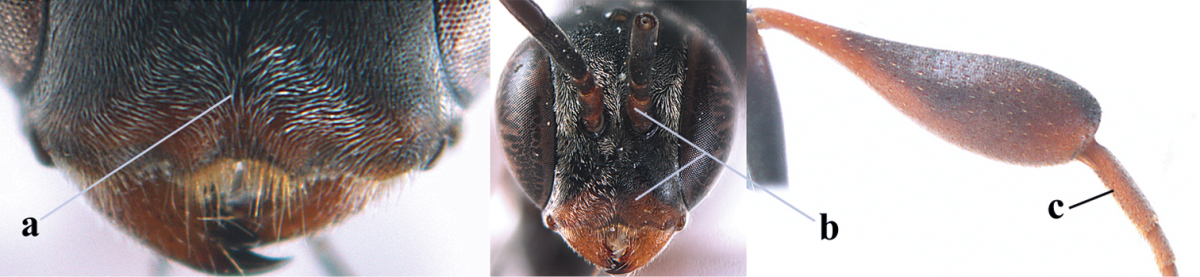		
–	Clypeus with small depression or depression obsolescent (aa); head and scapus black (bb); hind basitarsus usually slenderer (cc)	**47**
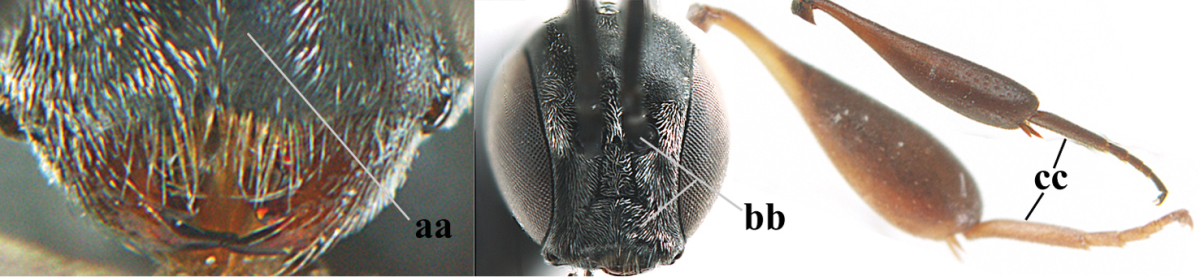		
47	Head elongate below eyes in anterior view, malar space about half as long as second antennal segment (a); propleuron stout (b)	**48**
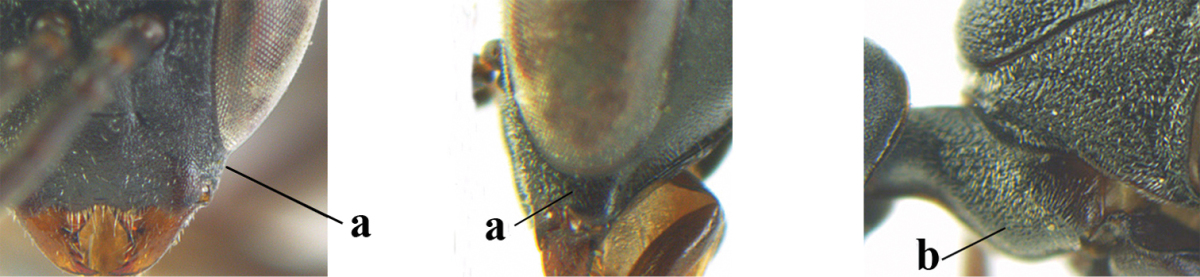		
–	Head normal in anterior view, malar space at most 0.3 times as long as second antennal segment (aa); shape of propleuron variable, usually slenderer (bb)	**49**
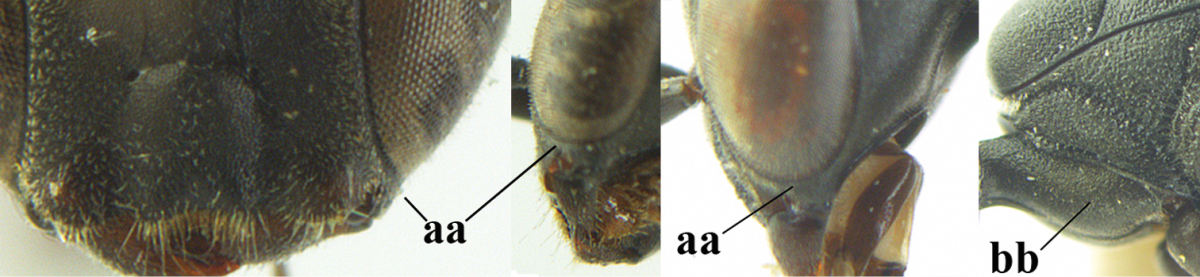		
48	Mesoscutum coriaceous (a); third antennal segment rather stout (b)	***Gasteruption minutum* (Tournier, 1877)**
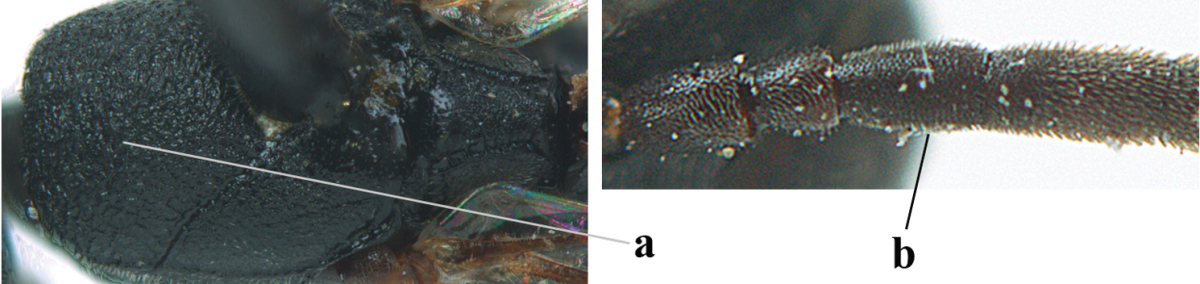		
–	Mesoscutum transversely rugulose (aa); third antennal segment slenderer (bb)	***Gasteruption lugubre* Schletterer, 1889 stat. rev.**
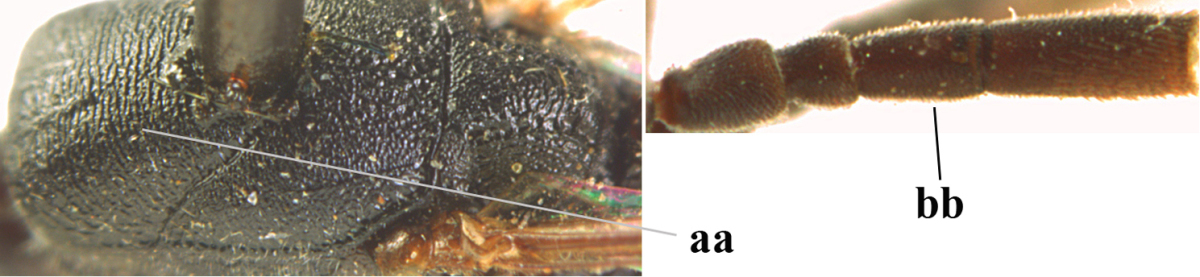		
49	Middle lobe of mesoscutum densely coriaceous, similar to fine sculpture of vertex (a) **and** occipital carina obsolescent, non-lamelliform medio-dorsally (b); hind tibia strongly swollen, resulting in a distinctly convex ventral border (c); [hind tibial spurs yellowish-brown or brown]	**50**
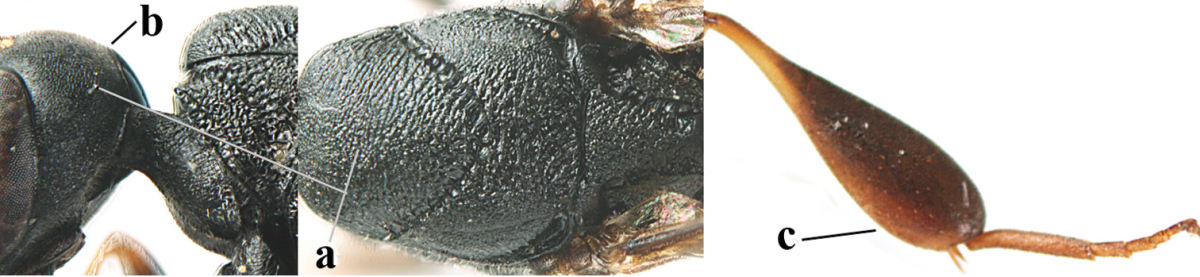		
–	Middle lobe of mesoscutum punctate, reticulate, rugose or rugulose, dissimilar to sculpture of vertex (aa); **if** similarly coriaceous then occipital carina distinctly lamelliform medio-dorsally (bb); hind tibia less swollen (cc), rarely similar (ccc)	**53**
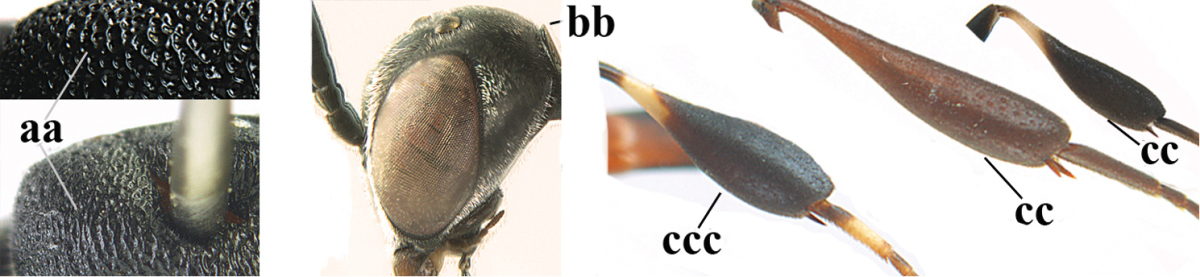		
50	Mandible pale yellowish basally (a)	**51**
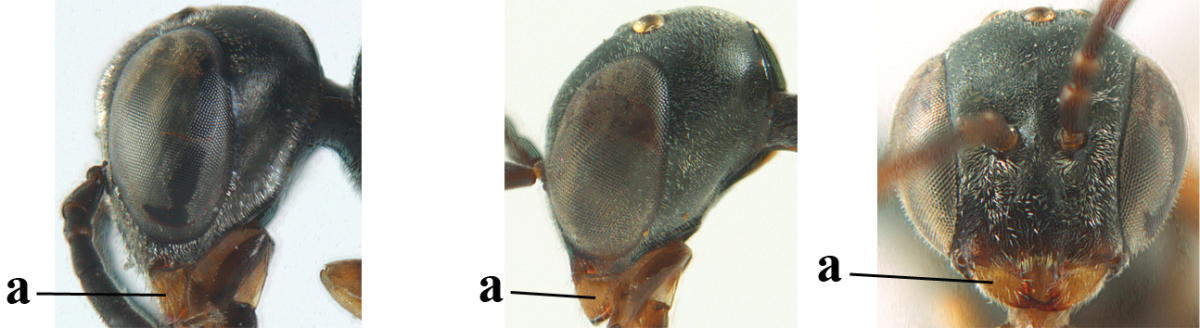		
–	Mandible dark brown or black basally (aa)	**52**
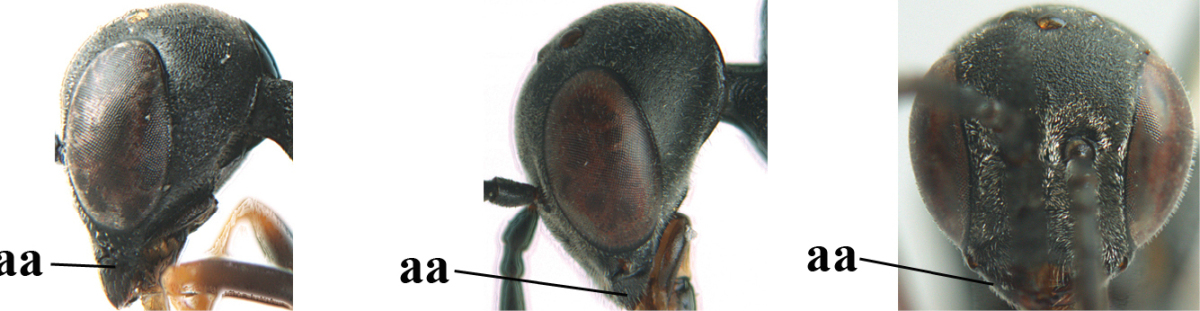		
51	Hind femur widened and shorter (a); apical half of hind tibia yellowish brown ventrally (b); apex of paramere dark brown (c)	***Gasteruption paglianoi* sp. n.**
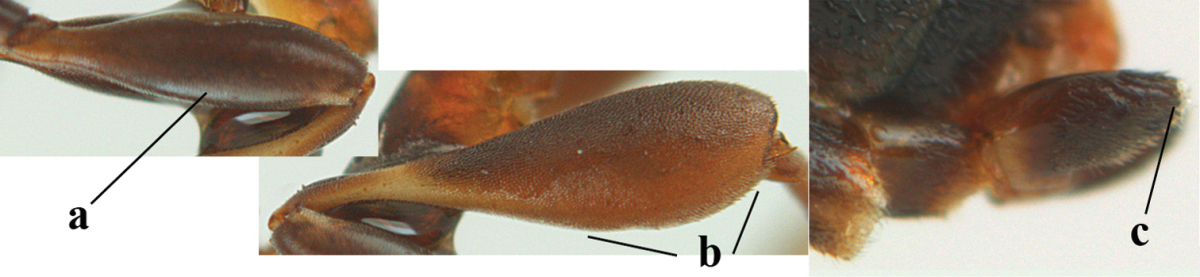		
–	Hind femur narrower and longer (aa); apical half of hind tibia dark brown ventrally (bb) or largely so; apex of paramere yellowish brown (cc)	***Gasteruption flavimarginatum* sp. n.**
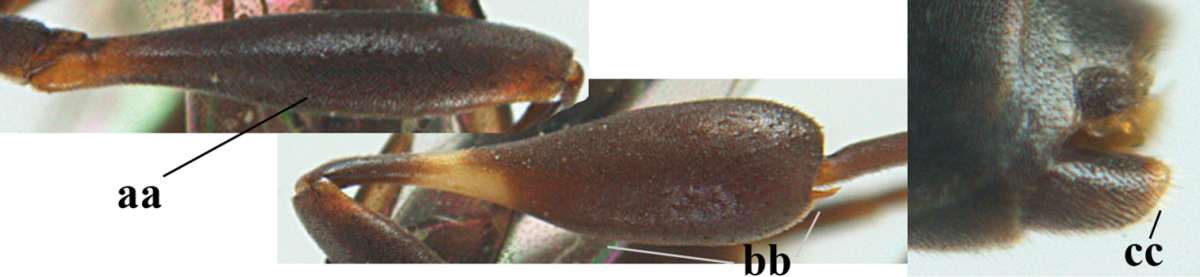		
52	Basal petiolate part of hind tibia shorter and wider in dorsal view (a); hind femur shallowly depressed ventrally (b); hind basitarsus slightly shorter than remainder of tarsus (c); [provisionally included; male unknown]	***Gasteruption brevibasale* sp. n.**
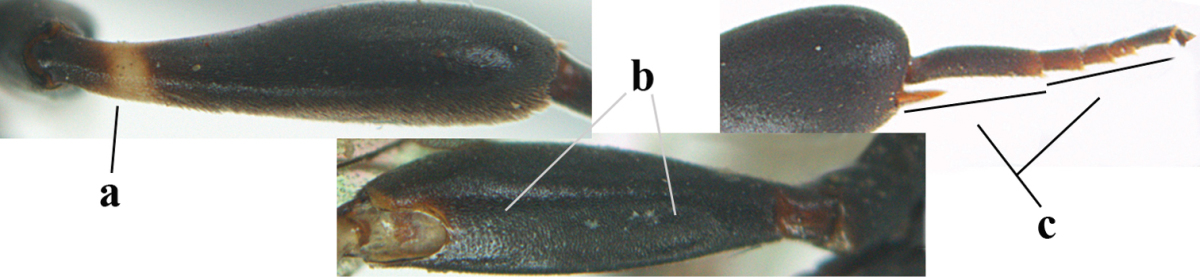		
–	Basal petiolate part of hind tibia longer and narrower in dorsal view (aa); hind femur slightly convex ventrally (bb); hind basitarsus longer than or as long as remainder of tarsus (cc)	***Gasteruption assectator* (Linnaeus, 1758)**
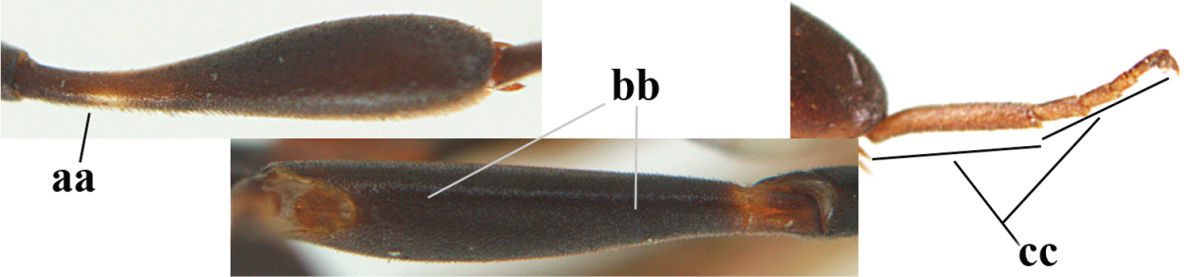		
53	Vertex strongly shiny (a) and temples rather long (b) in dorsal view; lateral lobes of mesoscutum coarsely rugose-reticulate and shiny, similar to middle lobe (c); pronotal side distinctly sculptured (d); hind tibia black or dark brown subbasally (e)	**54**
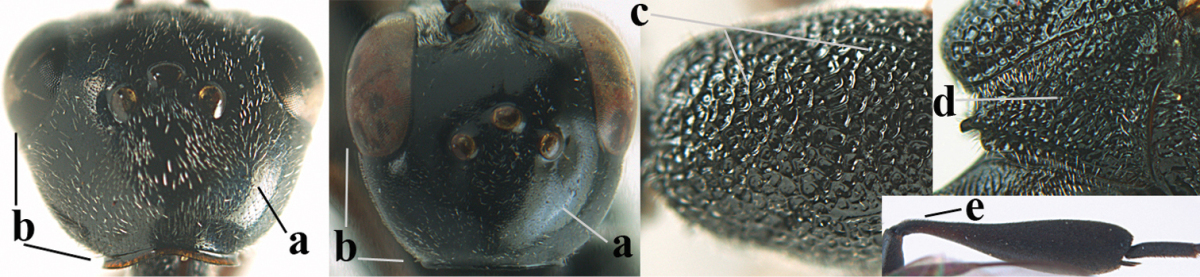		
–	Vertex with satin sheen to moderately shiny (aa) and temples shorter compared to eyes (bb) in dorsal view; lateral lobes of mesoscutum reticulate-punctate, punctate, punctulate or coriaceous and with satin sheen, if rugose-reticulate then dissimilar to middle lobe (cc), rarely similarly reticulate; pronotal side superficially sculptured, rugulose or coriaceous ventrally and at most with satin sheen (dd), at most with some rugae antero-ventrally; hind tibia with ivory patch subbasally (ee), but sometimes absent	**55**
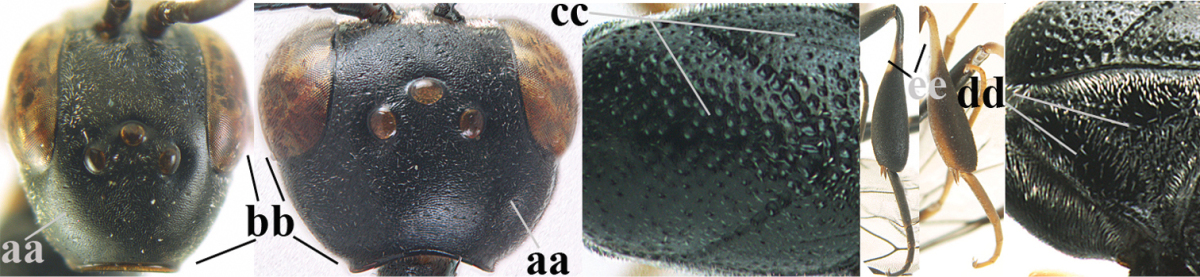		
54	Third antennal segment 1.5–1.7 times as long as second segment and similar to fourth segment (a); antesternal carina distinctly lamelliform (b); pronotal side ventrally largely reticulate (c); occipital carina often rather wide lamelliform medio-dorsally (d)	***Gasteruption merceti* Kieffer, 1904**
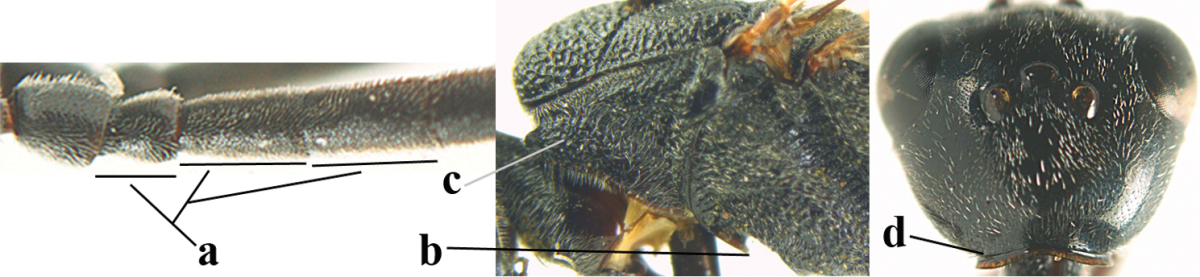		
–	Third antennal segment 1.2–1.3 times as long as second segment and distinctly shorter than fourth segment (aa); antesternal carina non-lamelliform (bb); pronotal side partly smooth between punctures (cc); occipital carina narrow lamelliform medio-dorsally (dd)	***Gasteruption agrenum* sp. n.**
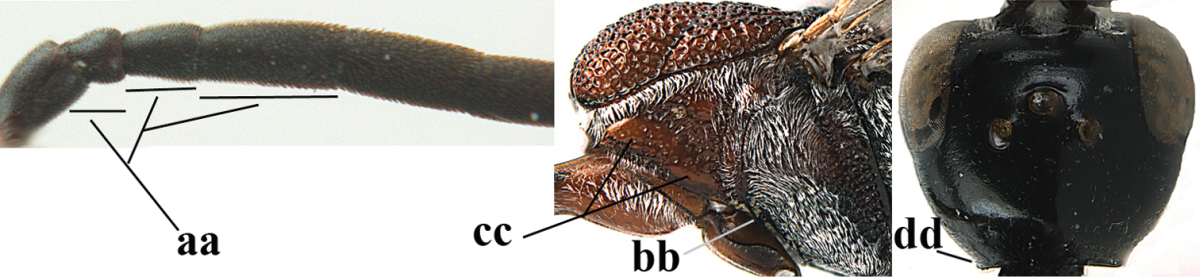		
55	Hind tibia swollen and ventral margin distinctly curved (a); head rather transverse, matt and coriaceous or finely micro-striate dorsally (b); hind basitarsus yellowish brown or partly ivory (c)	**56**
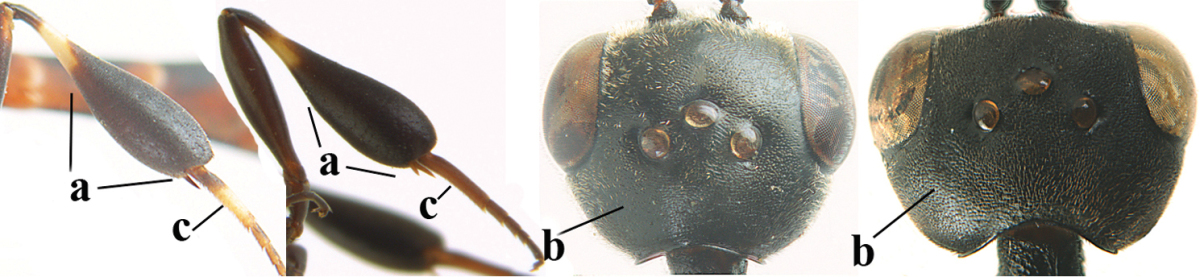		
–	Hind tibia slenderer and ventral margin hardly curved (aa); head usually less transverse and mainly punctulate (bb); hind basitarsus usually dark brown or brown (cc)	**57**
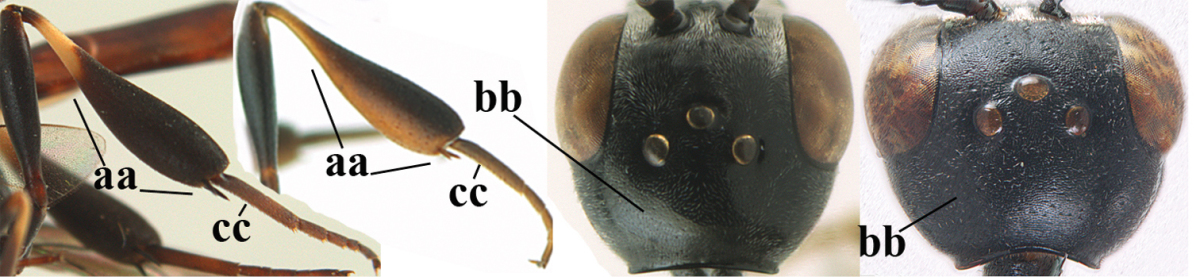		
56	Pronotal side largely smooth (except for some micro-sculpture) antero-ventrally (a); hind basitarsus partly ivory (b); third antennal segment slightly longer than second segment (c)	***Gasteruption henseni* sp. n.**
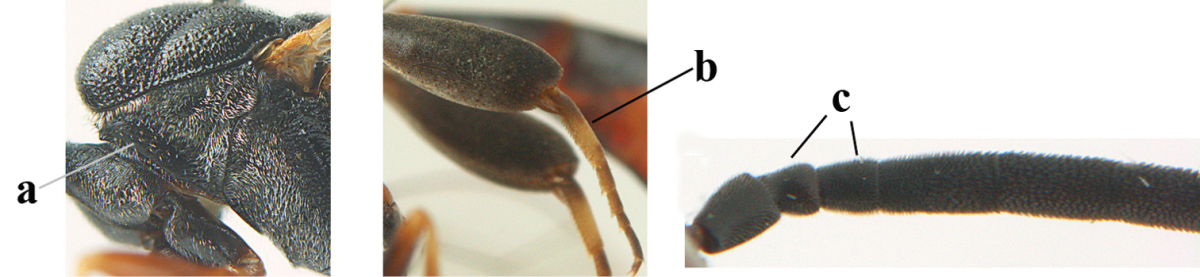		
–	Pronotal side coarsely reticulate antero-ventrally (aa); hind basitarsus yellowish brown (bb); third antennal segment distinctly longer than second segment (cc)	***Gasteruption undulatum* (Abeille de Perrin, 1879)**
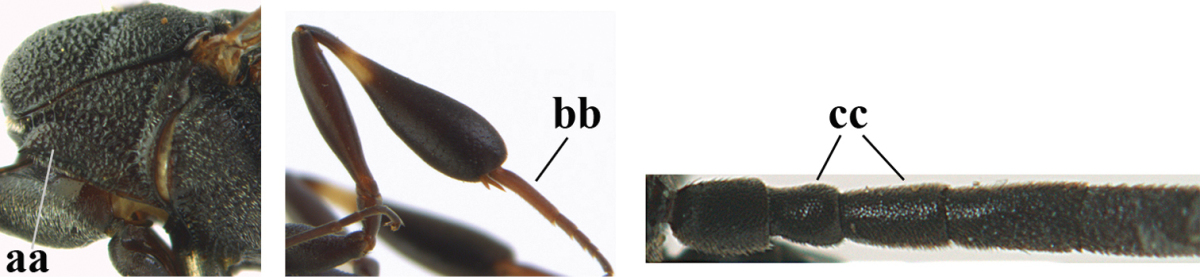		
57	Frons with several medium-sized punctures between fine dense punctulation (a), but punctures sometimes hardly developed; mesoscutum (b) and pronotal side (c) very coarsely sculptured; [hind basitarsus partly ivory or pale brown dorsally; third antennal segment distinctly longer than second segment; mandible yellowish or orange brown basally]	***Gasteruption punctifrons* sp. n.**
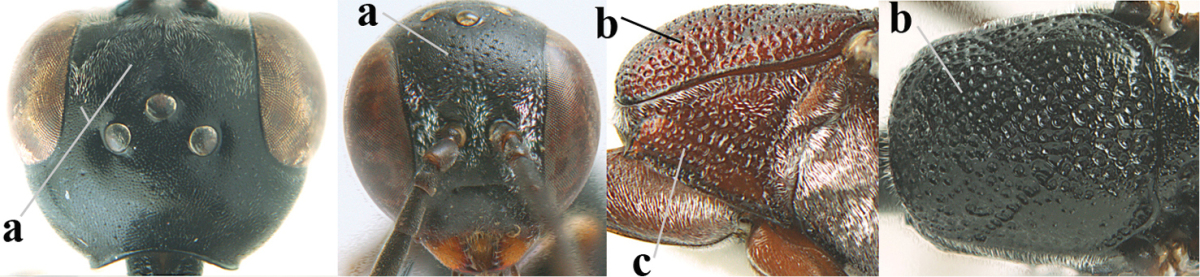		
–	Frons punctulate and without distinct punctures (aa), aciculate or largely smooth and more or less with few punctures; usually mesoscutum (bb) and pronotal side (cc) less coarsely sculptured	**58**
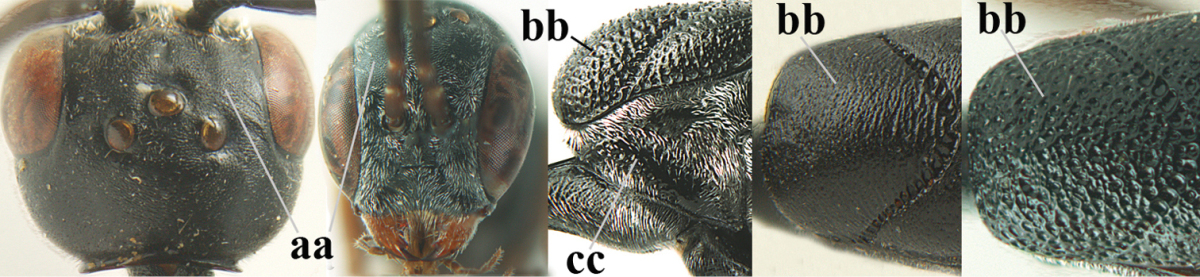		
58	Propleuron 0.9–1.1 times as long as distance between tegulae and anterior border of mesoscutum (a) **and** occipital carina brown and wider (b); middle lobe of mesoscutum with coarse punctures and sculptured interspaces (c)	**59**
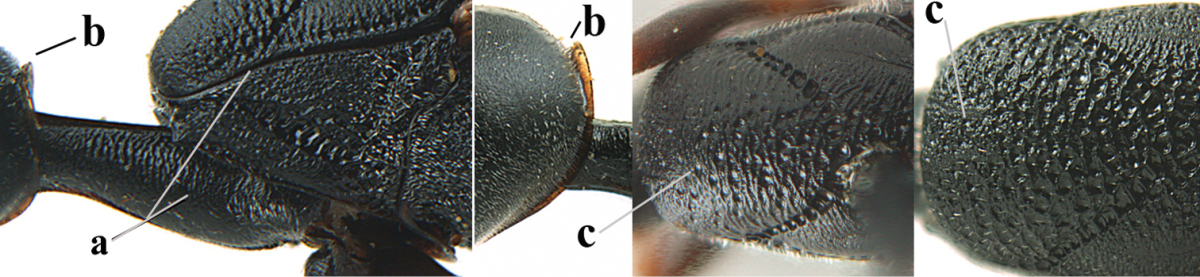		
–	Propleuron 0.8–1.0 times distance between tegulae and anterior border of mesoscutum (aa); if 0.9–1.0 times (aaa) then occipital carina black and narrower (bb); middle lobe of mesoscutum without distinctly spaced punctures (cc) or punctures with smooth interspaces (ccc)	**60**
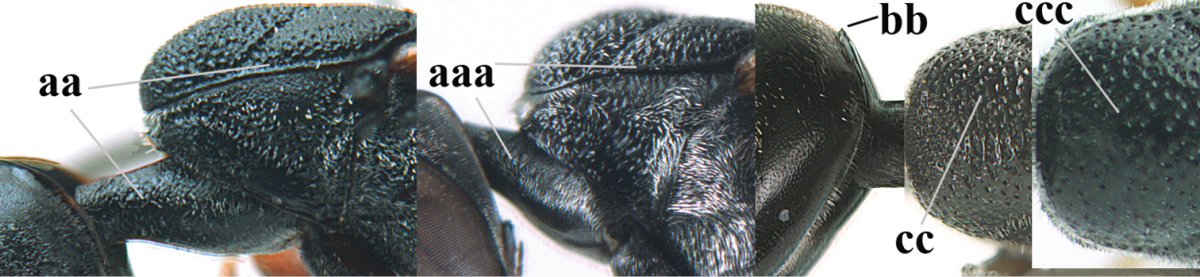		
59	Antesternal carina medium-sized lamelliform and curved apically (a); propleuron stout anteriorly in ventral view (b) and less slender in lateral view (c)	***Gasteruption opacum* (Tournier, 1877)**
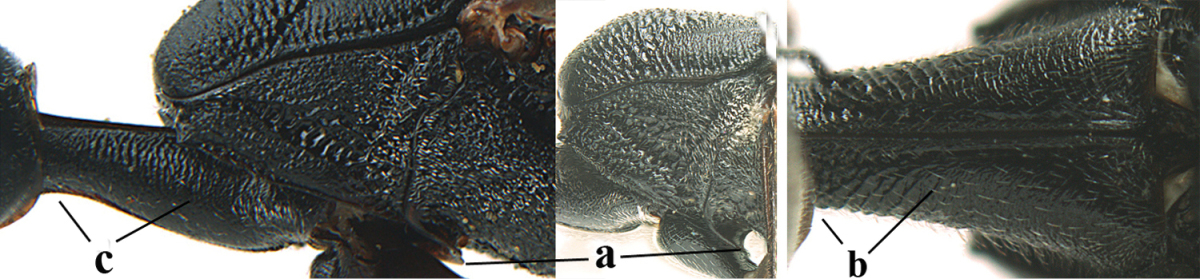		
–	Antesternal carina at most narrow lamelliform and straight apically (aa); propleuron slenderer anteriorly in ventral view (bb) and in lateral view (cc)	***Gasteruption syriacum* Szépligeti, 1903**
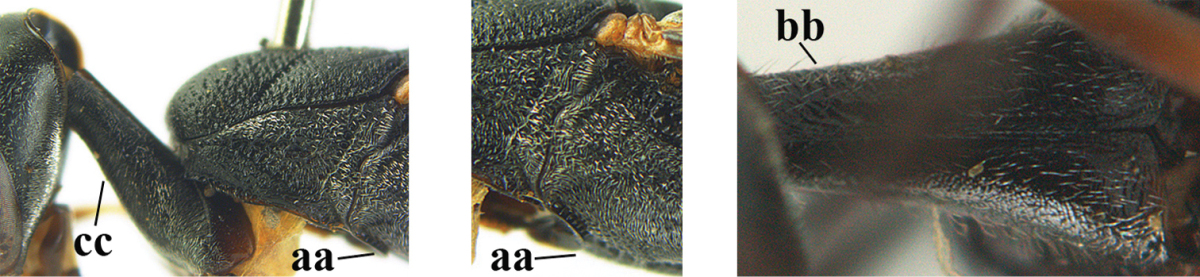		
60	Mesoscutum finely coriaceous (a), except medio-posteriorly; occipital carina moderately wide lamelliform (b); [antesternal carina non-lamelliform; if mesoscutum with some fine punctures, cf. unknown male of *Gasteruption ischnolaimum*]	***Gasteruption scorteum* sp. n.**
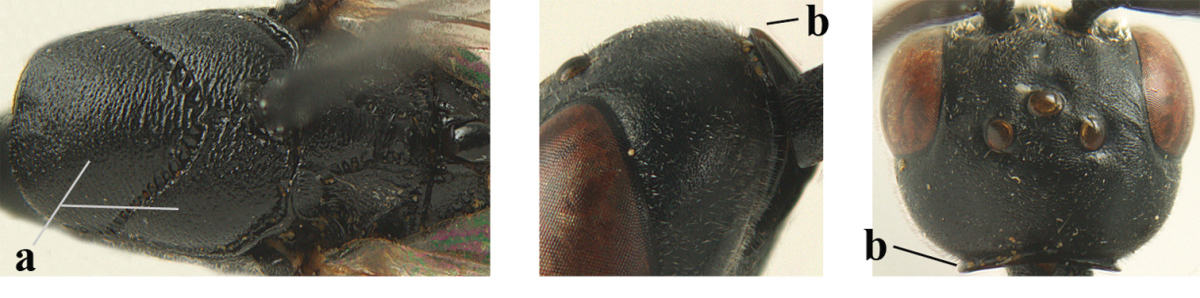		
–	Mesoscutum largely rugulose, punctate or punctate-rugulose (aa); occipital carina narrow lamelliform (bb)	**61**
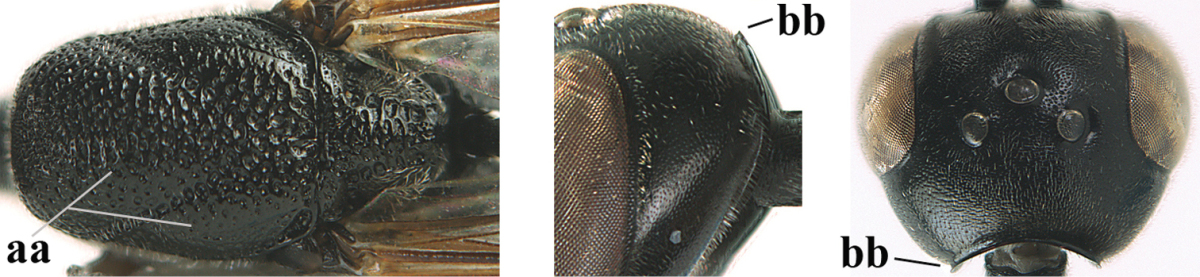		
61	Antesternal carina distinctly lamelliform (a), if carina rather narrow (*Gasteruption schlettereri*; aaa) then hind tibia pale yellowish ventrally (b) and lateral lobe of mesoscutum coarsely reticulate-punctate and shiny (c)	**62**
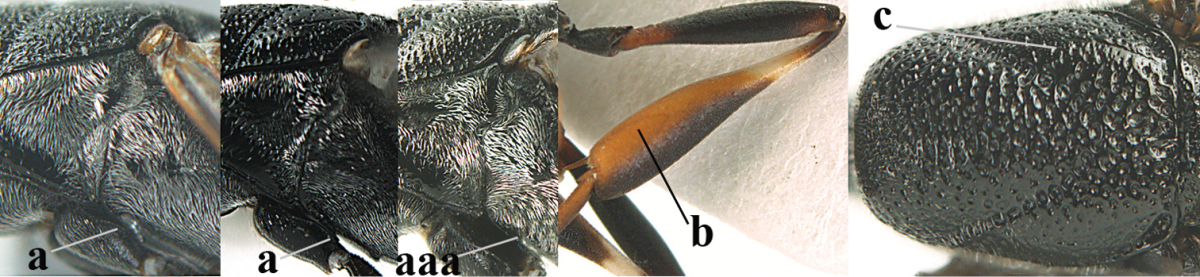		
–	Antesternal carina non-lamelliform (aa); hind tibia dark brown or largely black ventrally (bb); lateral lobe of mesoscutum usually less coarsely sculptured and/or with satin sheen or matt (cc)	**66**
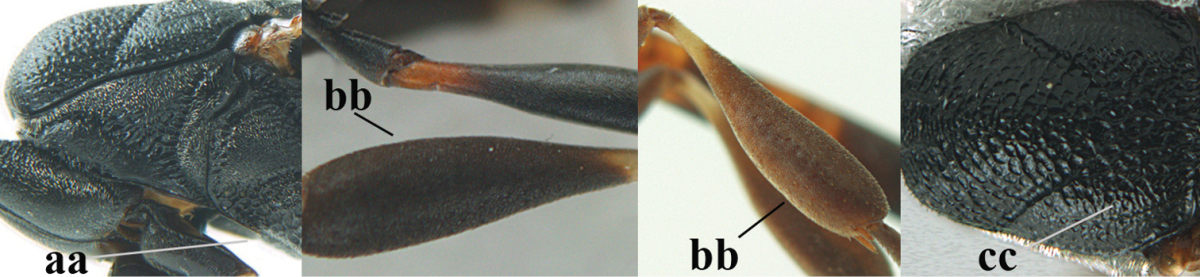		
62	Third antennal segment about as long as second segment (a); lateral lobes of mesoscutum more or less shiny (b); [propleuron 0.8–0.9 times distance between tegulae and anterior border of mesoscutum, subtriangular in ventral view]	**63**
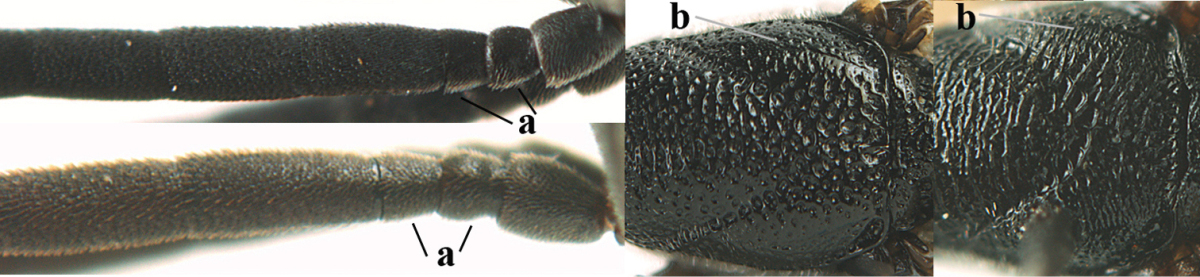		
–	Third antennal segment somewhat longer than second segment (aa); lateral lobes of mesoscutum often with satin sheen or matt (bb); [vertex usually with medio-posterior depression]	**64**
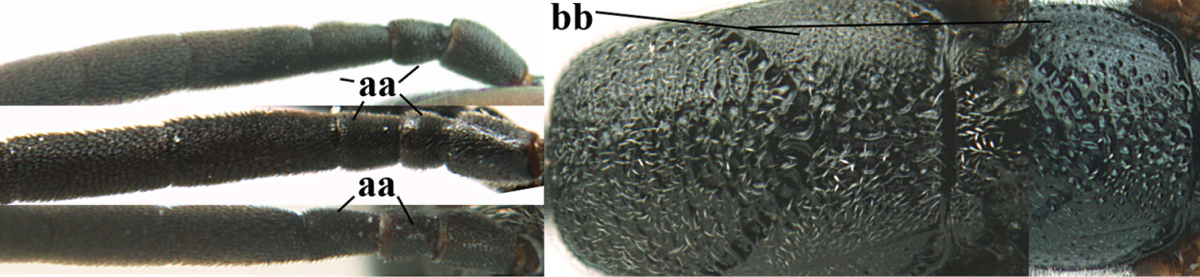		
63	Antesternal carina wide lamelliform and curved up apically (a); hind tarsus and tibia largely black or dark brown (b), rarely mainly brown or brownish yellow; third antennal segment less stout (c); lateral lobes of mesoscutum with satin sheen, rugulose, at most with few separate punctures (d)	***Gasteruption diversipes* (Abeille de Perrin, 1879)**
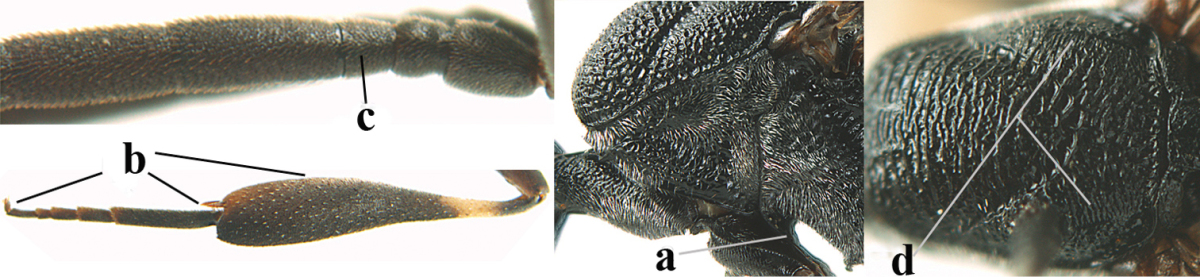		
–	Antesternal carina narrow lamelliform and straight apically (aa); hind tarsus (except telotarsus) and ventral half of hind tibia usually largely yellowish-brown or brown (bb); third antennal segment stout (cc); lateral lobes of mesoscutum distinctly shiny, coarsely rugose-punctate and smooth between punctures if interspaces are present (dd)	***Gasteruption schlettereri* Magretti, 1890**
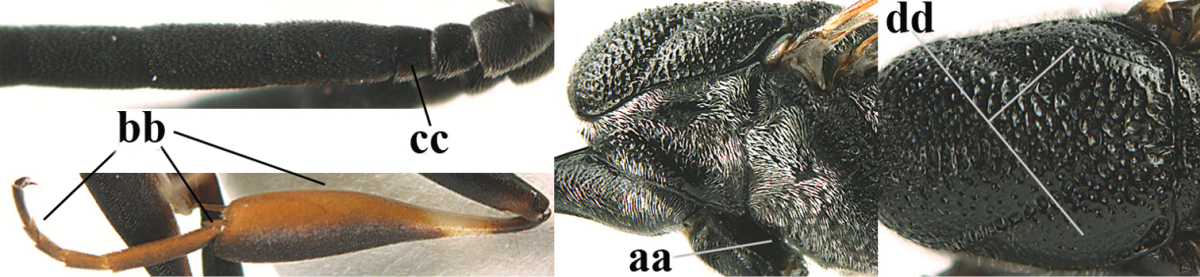		
64	Third antennal segment slenderer (a); interspaces between punctures of mesoscutum smooth (b); pronotal side smooth antero-ventrally (c); hind tibia largely brown ventrally and contrasting with dark dorsal part (d); vertex without medio-posterior depression (e)	***Gasteruption heminitidum* sp. n.**
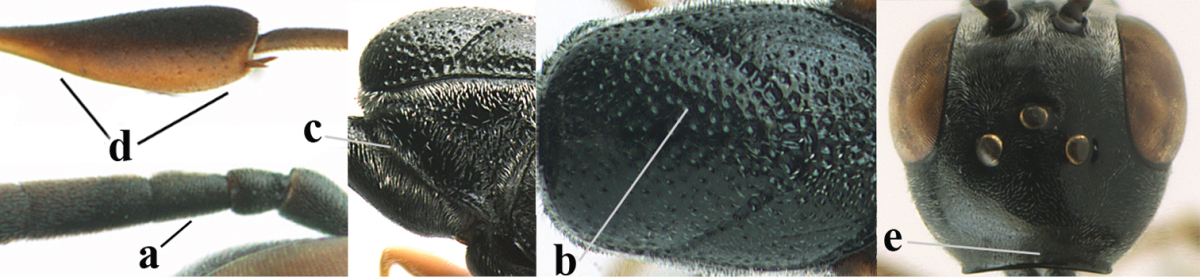		
–	Third antennal segment stout (aa); interspaces between punctures of mesoscutum punctulate or rugose (bb); pronotal side rugose or with some punctures antero-ventrally (cc); hind tibia ventrally and dorsally similarly coloured (dd); vertex more or less depressed medio-posteriorly (ee)	**65**
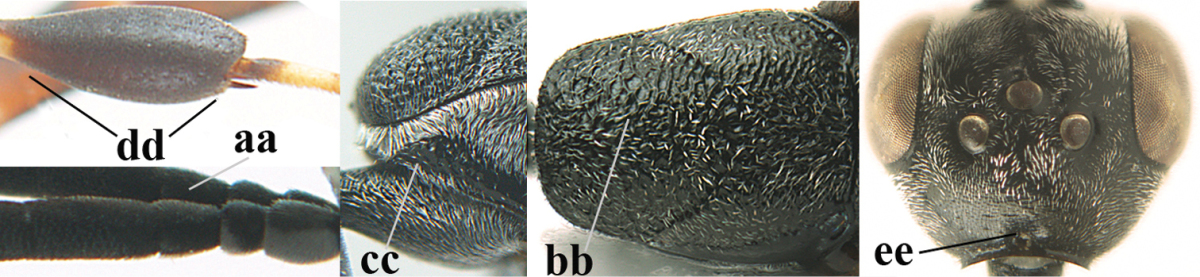		
65	Head dorsally only superficially punctulate and shiny (a); antesternal carina moderately curved up (b); pronotal side usually more elongate (c)	***Gasteruption pseudolaticeps* sp. n.**
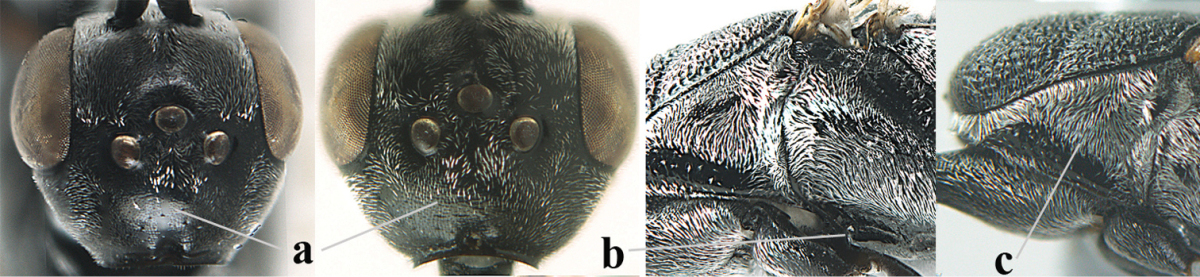		
–	Head dorsally densely micro-sculptured and with satin sheen (aa); antesternal carina strongly curved up (bb); pronotal side less elongate (cc)	***Gasteruption laticeps* (Tournier, 1877)**
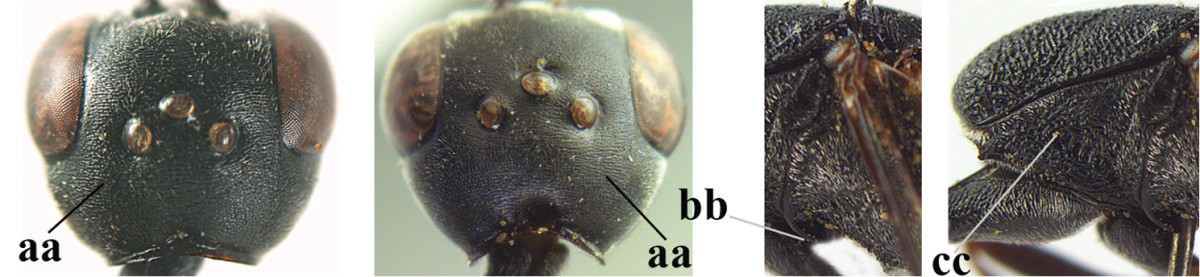		
66	Propleuron wide and short (a) **and** mesoscutum finely and densely rugulose (c); third antennal segment rather elongate (b); hind tibia black subbasally (d)	***Gasteruption freyi* (Tournier, 1877)**
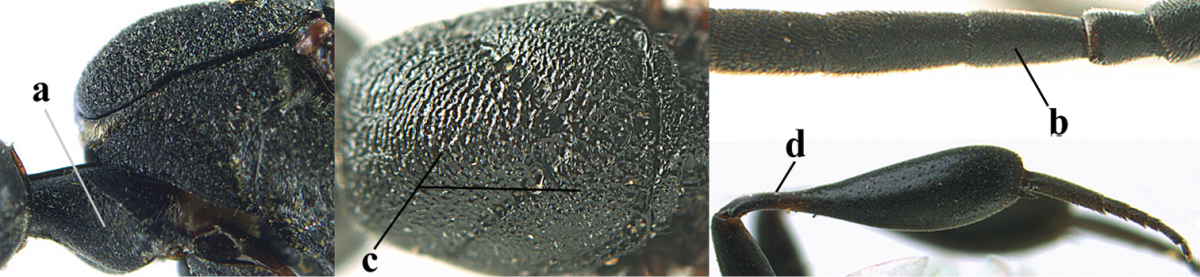		
–	Propleuron moderately slender and longer (aa); third antennal segment less elongate (bb); mesoscutum more or less punctate-rugulose or punctate-rugose (cc); base of hind tibia often ivory or pale brown subbasally (dd)	**67**
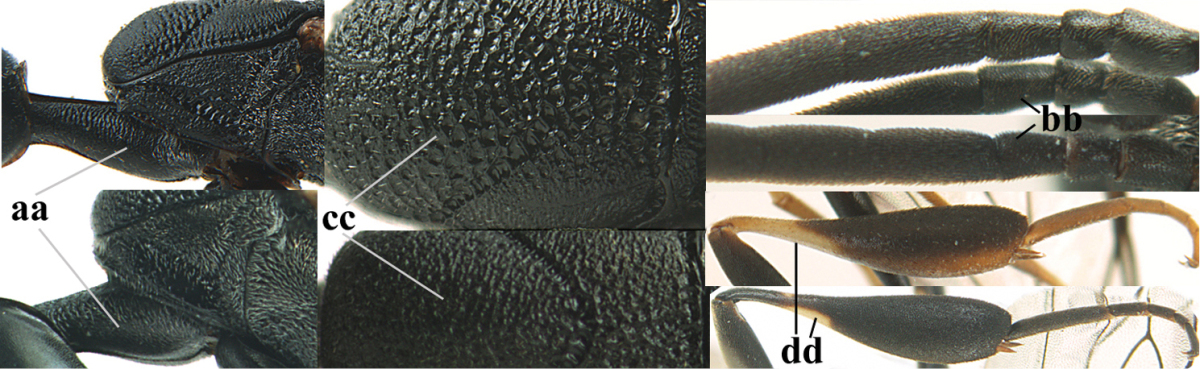		
67	Pronotal side postero-ventrally more or less coriaceous and matt (a); occipital carina non-lamelliform medio-dorsally (b); head less narrowed behind eyes (c); hind coxa rather coarsely rugulose dorsally (d)	**68**
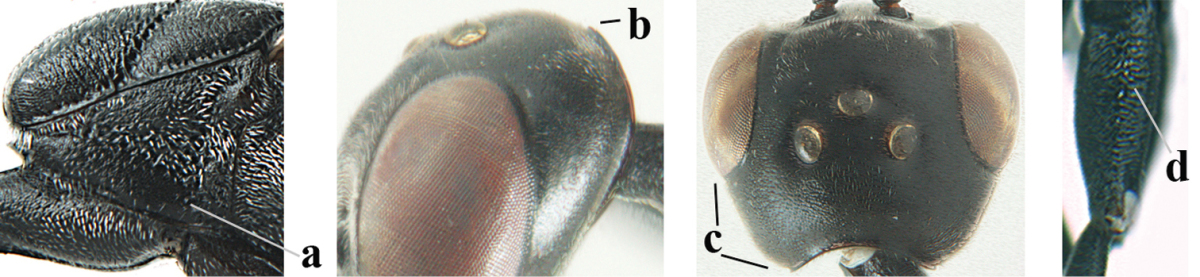		
–	Pronotal side postero-ventrally more or less rugulose, punctate or smooth and shiny (aa); occipital carina narrow to moderately wide lamelliform medio-dorsally (bb); head more narrowed behind eyes (cc); sculpture of hind coxa variable, often finely rugulose dorsally (dd); [third antennal segment rather slender]	**69**
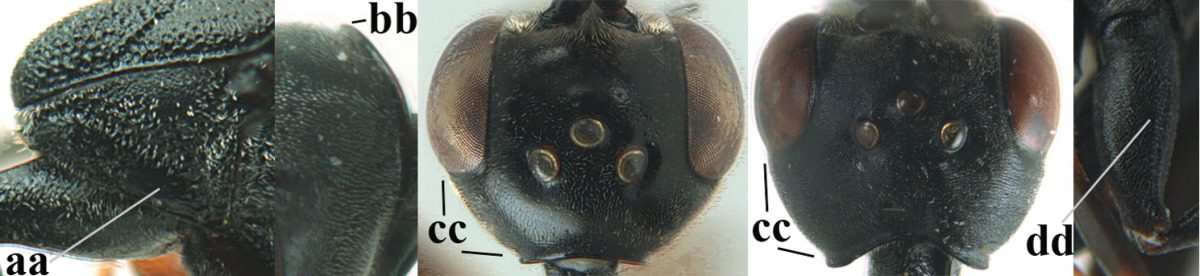		
68	Vertex rather convex medio-posteriorly in lateral view (a); head in dorsal view slightly more narrowed posteriorly (b)	***Gasteruption coriacoxale* sp. n.**
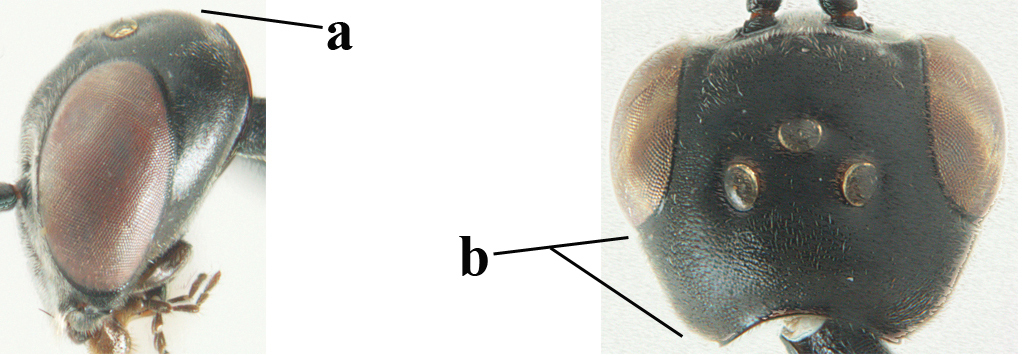		
–	Vertex rather flat medio-posteriorly in lateral view (aa); head in dorsal view less narrowed posteriorly (bb)	***Gasteruption phragmiticola* Saure, 2006**
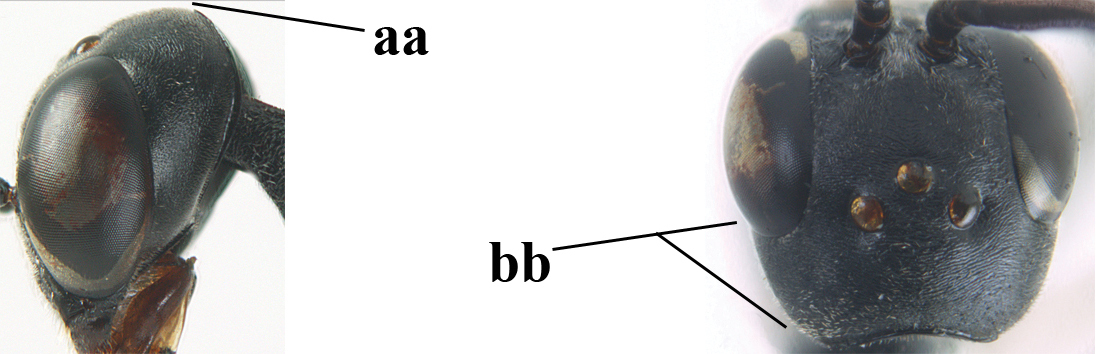		
69	Occipital carina moderately lamelliform (a); head rather wide in anterior view (b); head medio-posteriorly distinctly emarginate (c)	***Gasteruption nigrescens* Schletterer, 1885**
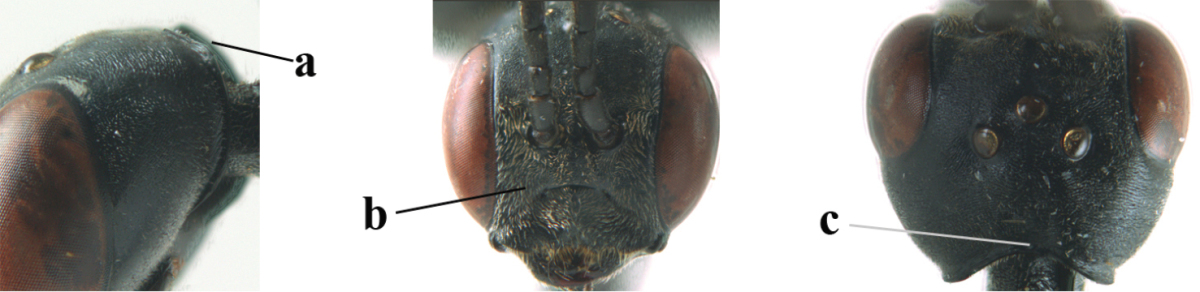		
–	Occipital carina narrow lamelliform (aa); head narrower in anterior view (bb); head medio-posteriorly hardly or not emarginate (cc)	**70**
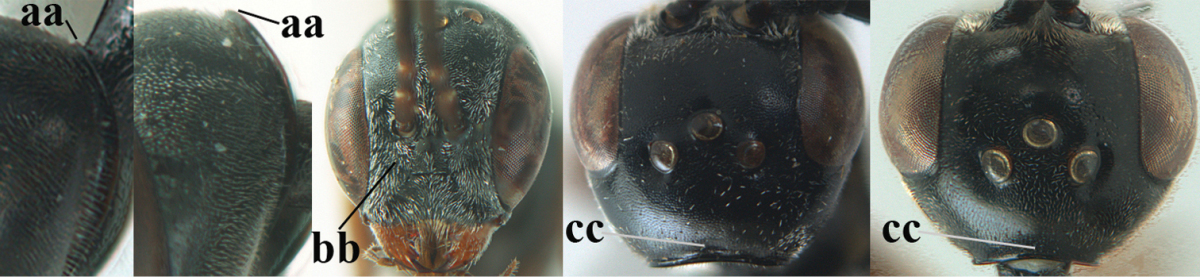		
70	Lateral lobe of mesoscutum with distinctly separated punctures (a); pronotal side often smooth or superficially coriaceous postero-ventrally (b)	**71**
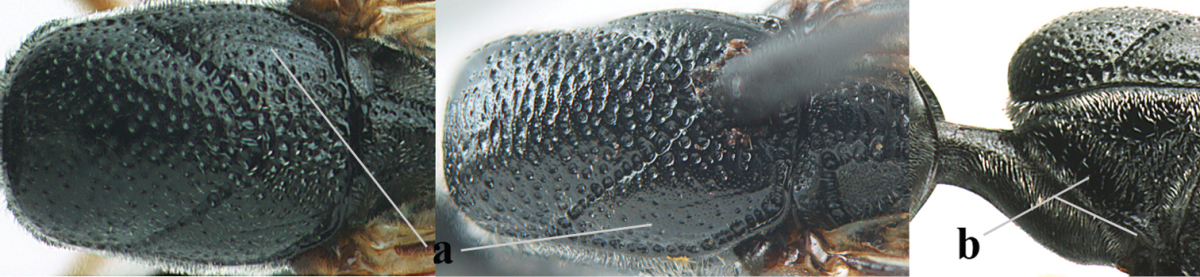		
–	Lateral lobe of mesoscutum mainly rugulose or rugose, without distinct punctures or punctures connected to rugae (aa); pronotal side often coriaceous or rugose postero-ventrally (bb); [vertex with superficial transverse elements posteriorly]	**72**
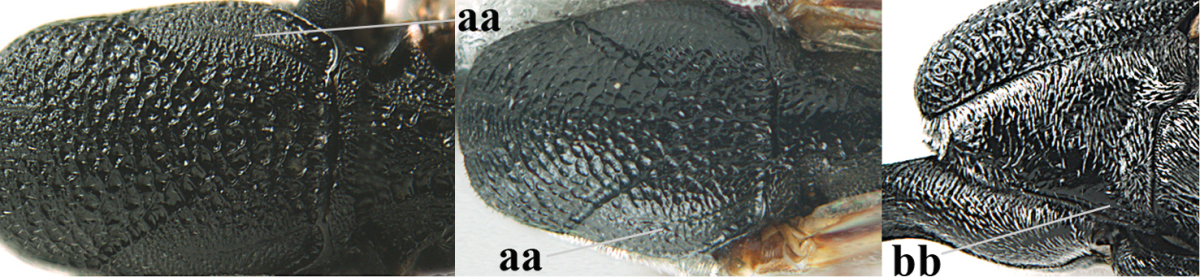		
71	Hind tibia largely black ventrally (a) and dark brown subbasally (b); head in dorsal view subglobular (c); mesoscutum more shiny (d); mandible dark brown or black basally (e)	***Gasteruption smitorum* sp. n.**
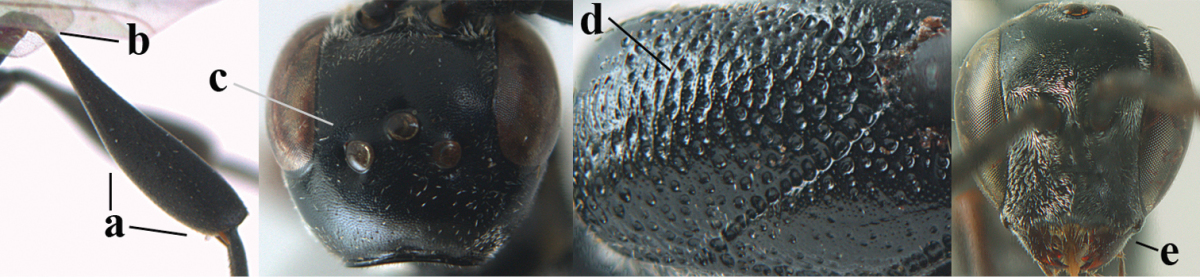		
–	Hind tibia brown or dark brown ventrally (aa) and ivory subbasally (bb); head in dorsal view trapezoid (cc); mesoscutum rather matt (dd); mandible brownish yellow basally (ee)	***Gasteruption schmideggeri* sp. n.**
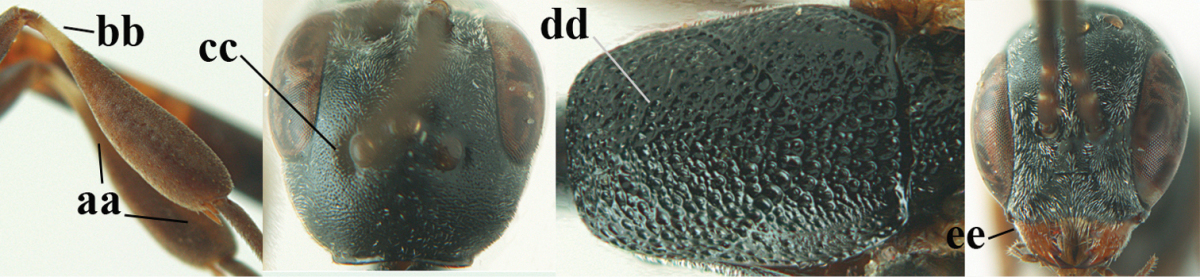		
72	Lateral lobe of mesoscutum transversely aciculate-rugulose (a); frons finely and densely aciculate-coriaceous (b); head rather square in dorsal view (c); [male unknown; figures of female]	***Gasteruption aciculatum* sp. n.**
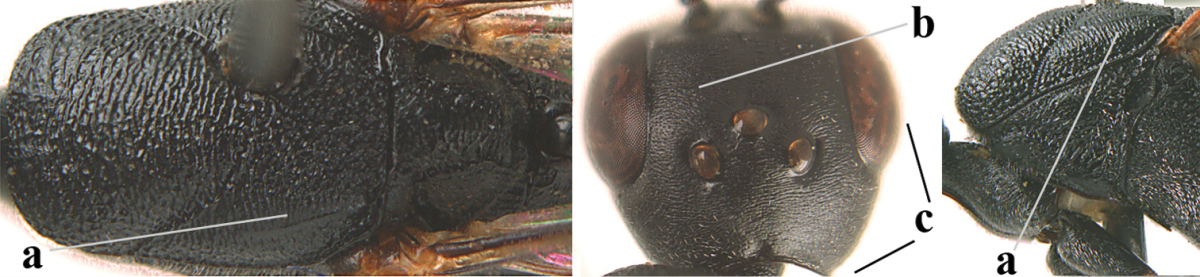		
–	Lateral lobe of mesoscutum obliquely punctate-rugulose or finely rugulose (aa); frons densely and finely rugulose-punctulate or very finely punctulate (bb); head subcircular or trapezoid in dorsal view (cc)	**73**
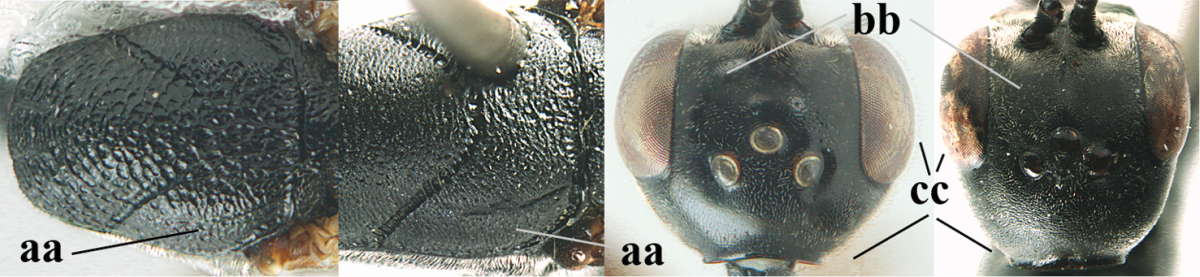		
73	Middle lobe of mesoscutum coarsely reticulate-rugose (a); pronotal side partly rugose ventrally (b); head distinctly narrowed posteriorly in dorsal view (c)	***Gasteruption nigrapiculatum* sp. n.**
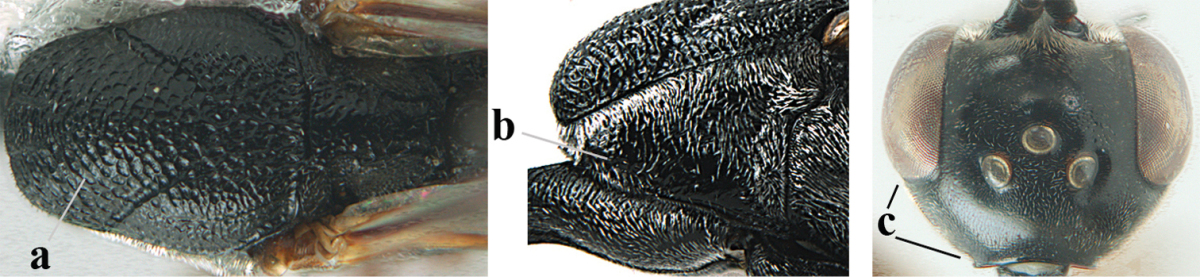		
–	Middle lobe of mesoscutum largely distinctly punctate-rugulose or finely rugulose (aa); pronotal side at most rugulose ventrally (bb); head less narrowed posteriorly in dorsal view (cc); [male unknown; figures of female]	***Gasteruption ischnolaimum* sp. n.**
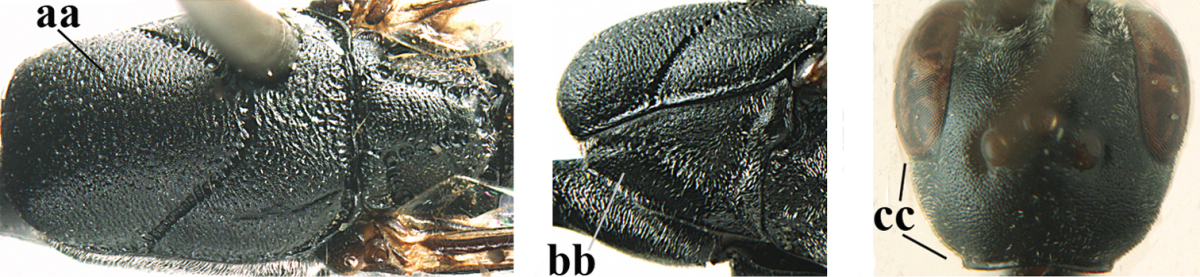		

## Descriptions

### Gasteruption aciculatum

Taxon classificationAnimaliaHymenopteraGasteruptiidae

van Achterberg sp. n.

http://zoobank.org/ABC467D2-07D9-4167-8B95-DFBA7DEFCBE8

Figs 5–13

#### Type material.

Holotype, ♀ (RMNH), “**Turkey**; (Van), 30 km N [of] Baskale, 2700 m, 11.vii.1987, R. Hensen”.

#### Diagnosis.

Head flattened dorsally, in front of occipital carina with small and shallow medio-posterior depression (Fig. 12); face moderately wide (Fig. 11); frons and vertex rather matt and densely and very finely transverse aciculate (Fig. 12); occipital carina narrowly lamelliform and dark brown; vertex without punctures; mandible dark brown basally; propleuron stout, coriaceous and 0.7 times as long as mesoscutum in front of tegulae; antesternal carina narrow and non-lamelliform; middle lobe of mesoscutum transversely rugose, without punctures and with satin sheen, lateral lobe regularly transversely rugulose with fine coriaceous interspaces and medio-posteriorly irregularly reticulate-rugose (Fig. 8); scutellum superficially coriaceous, weakly transversely rugulose and with satin sheen; ventral half of mesopleuron and metapleuron silvery pilose (Fig. 7); hind basitarsus dark brown basally, apical half largely ivory; hind tibia moderately slender and with subbasal ivory patch (Fig. 13); ovipositor sheath 0.9 times as long as body, 1.4 times as long as metasoma, 2.6 times as long as hind tibia and tarsus combined and 4.2 times hind tibia; white apical part of ovipositor sheath 2.1 times as long as hind basitarsus; length of body 11 mm.

#### Description.

Female, length of body 11.2 mm (of fore wing 5.5 mm).

**Figures 5–13.**
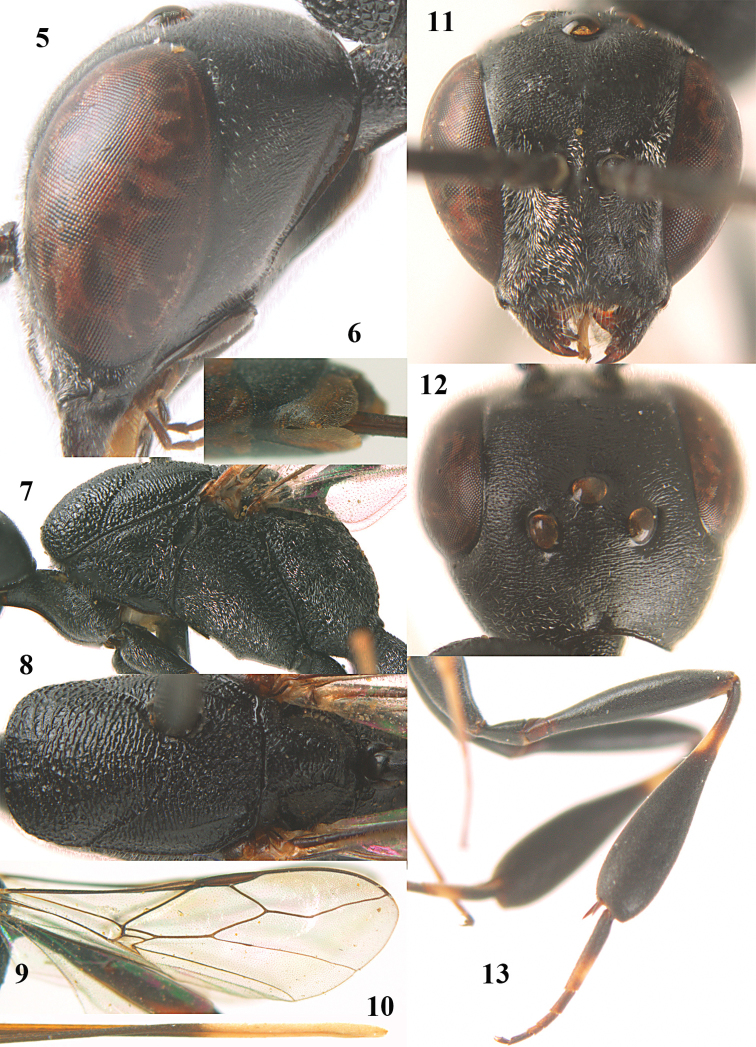
*Gasteruption aciculatum* sp. n., female, holotype. **5** head lateral **6** hypopygium, ventral **7** mesosoma lateral **8** mesonotum dorsal **9** fore wing **10** apex of ovipositor sheath **11** head anterior **12** head dorsal **13** hind leg.

*Head*. Head flattened dorsally, in front of occipital carina with small and shallow medio-posterior depression (Fig. 12); face anteriorly conspicuously silvery pilose; occipital carina narrowly lamelliform, dark brown (Figs 5, 7, 12); third and fourth antennal segments 1.6 and 2.2 times as long as second segment, apical segment 1.9 times as long as penultimate segment; face moderately wide (Fig. 11); frons and vertex rather matt and densely and very finely transverse aciculate (Fig. 12); ventrally head not enlarged in anterior view, malar space 0.3 times length of pedicellus.

*Mesosoma*. Length of mesosoma 1.7 times its height; propleuron stout and 0.7 times as long as mesoscutum in front of tegulae, matt and distinctly coriaceous; laterally pronotum largely rugose antero-ventrally and superficial coriaceous postero-ventrally; side of pronotum with medium-sized obtuse tooth antero-ventrally; antesternal carina narrow and non-lamelliform; middle lobe of mesoscutum transversely rugose, without punctures and with satin sheen, lateral lobe regularly transversely rugulose with fine coriaceous interspaces and medio-posteriorly irregularly reticulate-rugose (Fig. 8); notauli narrow and moderately impressed; scutellum superficially coriaceous, weakly transversely rugulose and with satin sheen; coriaceous dorsal area of mesopleuron large; mesopleuron ventrally and metapleuron silvery pilose (Fig. 7).

*Legs*. Length of hind femur, tibia and basitarsus 4.6, 4.4 and 5.2 times their width, respectively; hind tibia moderately slender and ventrally curved (Fig. 13); hind coxa densely rugulose antero-dorsally, transversely striate postero-dorsally; hind basitarsus moderately slender, as long as remainder of tarsus and hardly widened in dorsal view.

*Metasoma*. Ovipositor sheath 0.9 times as long as body, 1.4 times as long as metasoma, 2.6 times as long as hind tibia and tarsus combined and 4.2 times hind tibia; white apical part of ovipositor sheath 2.1 times as long as hind basitarsus (Fig. 10).

*Colour.* Black; mandible, antenna from fourth segment, tegulae, palpi, pterostigma, fore and middle femora and tibiae (except ivory base and apex of tibiae), telotarsi, hind tarsus (except apical ivory part of basitarsus and apices of second-fourth segments), metasoma (but second-fourth tergites apically and apical half of hypopygium and apices of other sternites yellowish brown), more or less dark brown; hind tibia subbasally and apical half of hind basitarsus ivory; hind tibial spurs dark brown and slightly paler than base of hind basitarsus; remainder of tarsi yellowish brown; apex of ovipositor sheath white; wing membrane slightly infuscate.

*Male.* Unknown.

#### Distribution.

Turkey.

#### Biology.

Unknown. Collected in July.

#### Etymology.

Named “*aciculatum*”, because of the very finely aciculate frons and vertex.

### Gasteruption agrenum

Taxon classificationAnimaliaHymenopteraGasteruptiidae

van Achterberg sp. n.

http://zoobank.org/77585133-BA46-4F91-9886-0472EC8593ED

Figs 14 –28

#### Type material.

Holotype, ♀ (RMNH), “N. **Iran:** Qazvin, Zereshk Road, MT5, 28.vii.-18.viii.2011, M. Khayrandish, RMNH’12”. Paratypes (34 ♀ + 25 ♂): 1 ♀ (RMNH), “N. Iran: Tehran, Shahriar, MT25, 11-18.v.2010, M. Khayrandish, RMNH’12”; 1 ♀ (TMUT), id., but MT 24, 1–8.vi.2010, A. Nadimi; 1 ♀ (MZL) [Lausanne], “Iran, Tehran”; 1 ♂ (BZL), “Iran: Azer. e Sh. prov., Sis, 10 km E [of] Shabestar, N38°26', E45°86', 1540 m, 19.vi.2010, Mi. Halada”; 1 ♀ (BZL), “Iran cent., env. Nain, 5.v.1999, K. Deneš sen.”; 1 ♂ (BZL), “**Jordan** E., Rawayshid, 24.iv.1996, Marek Halada”; 1 ♂ (RMNH), “Jordan W., 10 km N [of] Petra, 3.v.1996, Marek Halada”; 3 ♂ (BZL, RMNH), “**Syria**, 40 km NE of Damascus, 13.v.1996, Mi. Halada ing.”; 1 ♂ (BZL), “Syria west, 50 km S [of] Homs, 24.v.1996, Ma. Halada”; 1 ♂ (BZL), “Syria N, Marbij, 9.v.1996, Marek Halada”; 1 ♀ (BZL, RMNH), “**Turkey** east, 20 km W [of] Van, 5.vii.1997. Ma. Halada”; 3 ♂ (BZL, RMNH), “Turkey east, 10 km N [of] Tatvan, 24.vi.1997, Ma. Halada”; 1 ♀ + 1 ♂ (BZL), “TR. or., env. Agri, 27.vi.[19]93, Jiroušek”; 1 ♀ + 2 ♂ (BZL), “Turkey E., 40 km NE [of] Muradiye, 2200 m, 5.vii.2000, M. Halada”; 1 ♀ (BZL), “Türkei, Konya: Sille, 12.vi.1978, Max Schwarz”, “*Gasteruption psilomma* Kieff., ♀, det. Madl, 1988”; 1 ♀ (CSC), “Türkei, Konya: 10 km S [of] Karaman, 19.vi.1985, Max Schwarz”, det. id.; 1 ♂ (BZL), “Türkei, Konya: Obruk, 7.vi.1978, Max Schwarz”, det. id. but ♂; 23 ♀ + 7 ♂ (BZL, RMNH), “TR, Burdur, 20 km SW [of] Burdur, N37°37', E30°9', 940 m, 7.vii.2006, M. Halada”; 1 ♂ (BZL), “TR. or., env. Tatvan, 30.vi.[19]93, K. Deneš”; 1 ♂ (BZL), “Turkey, Hakkari prov., Akcali, 35 km S [of] Hakkari, N37°71', E44°3', 1700 m, 21.vi.2010, M. Halada”; 1 ♂ (BZL), “Türkei mer. or., Halfeti env., 3–5.v.1994, Mi. Halada”; 1 ♂ (CSC), “Türkei, Nevsehir: Ürgüp, 4.vi.1978, Max. Schwarz”; 1 ♀ (Beograd University Collection), “[**Greece**], Creta, Iraklion, 25.ix.[19]59, Stancic”; 1 ♀ (MHNG), “[S. **Russia**,] Sarepta, Becker”, (Sarepta is a former German colony of Moravian Brothers founded in 1765 near Astrakhan, Volga Delta in South Russia).

#### Diagnosis.

**Figures 14–22.**
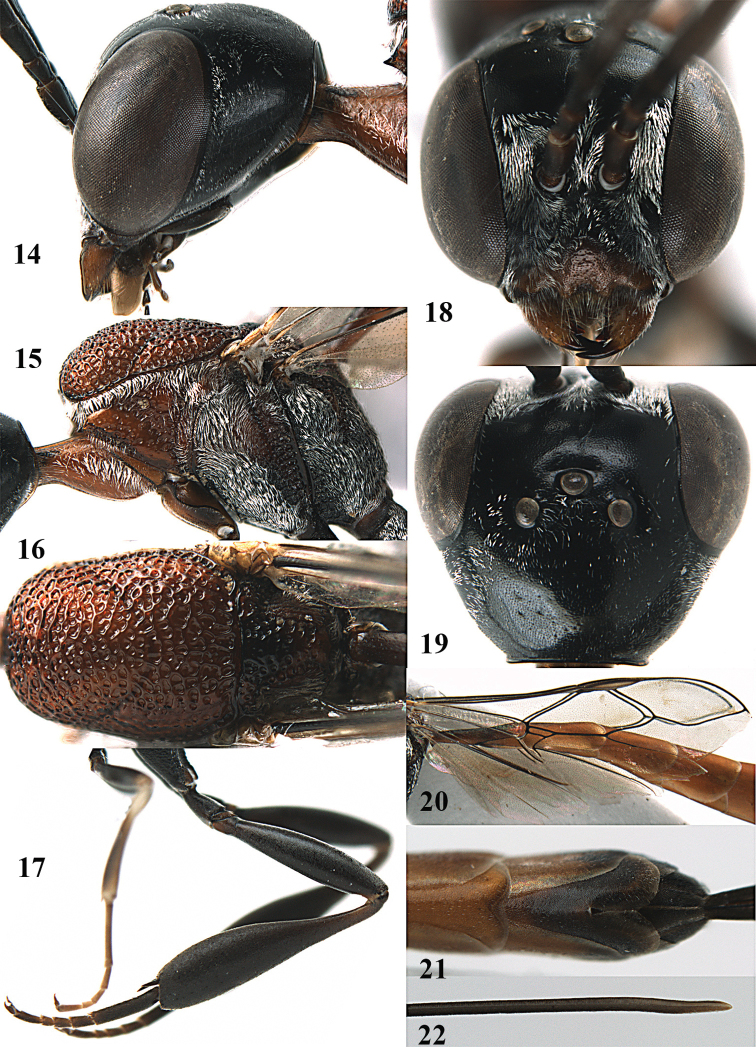
*Gasteruption agrenum* sp. n., female, holotype. **14** head lateral **15** mesosoma lateral **16** mesonotum dorsal **17** hind leg **18** head anterior **19** head dorsal **20** fore wing **21** hypopygium, ventral **22** apex of ovipositor sheath.

Head moderately convex dorsally in lateral view (Fig. 14), in front of occipital carina without medio-posterior depression; face medium-sized (Fig. 18); frons and vertex shiny and superficially finely punctulate mixed with some fine punctures (Fig. 19); occipital carina narrow, non-lamelliform; propleuron 0.8 times as long as mesoscutum in front of tegulae and large smooth and shiny; pronotal side mainly punctate and shiny ventrally (Fig. 15); antesternal carina narrow and non-lamelliform; middle and lateral lobe of mesoscutum coarsely transversely reticulate-rugose and shiny (Fig. 16); mesopleuron and metapleuron conspicuously white pilose (Fig. 15); middle lobe rounded antero-laterally (Fig. 16); fore coxa close to mesopleuron (Fig. 15); hind basitarsus entirely dark brown; hind tibia rather swollen and entirely dark brown (Fig. 17); fifth sternite of female orange brown (Fig. 21); apical 0.4–0.5 of hypopygium of female incised; ovipositor sheath 1.1–1.2 times as long as body, 1.6–1.8 times as long as metasoma, 5.0–5.7 times as long as hind tibia and 3.7–4.3 times as long as hind tibia and tarsus combined; pale brown or ivory apical part of ovipositor sheath 0.2–0.5 times as long as hind basitarsus; paramere of male black apically (Fig. 25); third antennal segment of male 1.3 times as long as second segment, fourth segment 1.8 times third segment and as long as second and third segments combined, fifth segment as long as fourth segment (Fig. 28); hind tibia of both sexes entirely dark brown or blackish; length of body 10–17 mm. Some of the paratypes has been identified as *Gasteruption psilomma* Kieffer or *Gasteruption schlettereri*. The new species disagrees from *Gasteruption schlettereri* by the length of the short pale apical part of the ovipositor sheath (long in *Gasteruption schlettereri*), smaller ocelli (larger), hind basitarsus dark brown (partly ivory), propleuron largely smooth (distinctly sculptured) and sternites (except hypopygium) orange or reddish brown (dark brown). The interpretation of the Spanish *Gasteruption psilomma* Kieffer, 1904, is problematical. The male holotype of *Gasteruption psilomma* from Spain (Ribas, Catalonia) could not be found in the Mercet Collection (Madrid), as reported before by Madl (1988b). According to his redescription *Gasteruption psilomma* is close to *Gasteruption trichotomma* from which it could be separated according to Kieffer (1904a) by having the ovipositor slightly longer than the metasoma and the distance between the posterior ocelli equal to the distance from the ocelli to the eyes. However, in 1904 Kieffer did not mention the ovipositor in the description; he had only the male holotype! For the interpretation of *Gasteruption psilomma* is better to examine carefully Spanish male specimens which agree with the original description. Most striking in the original description is the combination of red second and third metasomal tergites with a black hind leg, a short third antennal segment (1.3 times as long as second segment) and a shiny line in front of the anterior ocellus. Males of *Gasteruption forticorne* Semenov, 1892, fit well and, therefore, we synonymise *Gasteruption psilomma* with *Gasteruption forticorne* (syn. n.). The new species differs by the short malar space (distinctly developed in *Gasteruption forticorne*), the reticulate mesoscutum (transversely rugose), the short pale apical part of the ovipositor sheath (medium-sized) and dark brown hind basitarsus (partly ivory). Among the East Palaearctic species the new species is rather similar to *Gasteruption argentatum* Semenov & Kostylev, 1928. The new species has the temple distinctly shorter than the eyes in dorsal view (about as long in *Gasteruption argentatum*), the mesoscutum coarsely reticulate, sparsely setose and no smooth interspaces (punctate, densely setose and with smooth interspaces), the hind basitarsus 0.8 times as long as remainder of tarsus without claws (about of equal length) and length of ovipositor sheath 1.1–1.2 times as long as body and 5.0–5.7 times as long as hind tibia (0.7–0.8 times as long as body and 3.1–3.3 times as long as hind tibia).

#### Description.

Female, length of body 13.0 mm (of fore wing 6.1 mm).

*Head*. Head moderately convex dorsally in lateral view, in front of occipital carina without medio-posterior depression; face, frons anteriorly and temples conspicuously silvery pilose; occipital carina non-lamelliform (Fig. 14); third and fourth antennal segments 1.7 and 2.5 times as long as second segment; face medium-sized (Fig. 18); frons and vertex shiny and superficially finely punctulate mixed with some fine punctures; temples gradually narrowed behind eyes (Fig. 19); ventrally head not enlarged in anterior view, malar space 0.2 times length of pedicellus.

*Mesosoma*. Length of mesosoma 1.7 times its height; propleuron 0.8 times as long as mesoscutum in front of tegulae, stout and shiny, with long silvery setae and some punctures; pronotal side mainly punctate and shiny ventrally, remainder reticulate-punctate but with nearly smooth patch, sparsely setose except long setae dorsally and posteriorly; side of pronotum with a distinct acute tooth antero-ventrally; antesternal carina narrow lamelliform; middle and lateral lobe of mesoscutum coarsely transversely reticulate-rugose and shiny (Fig. 16); scutellum coarsely transversely rugose and with some coarse punctures; mesopleuron and metapleuron conspicuously silvery pilose (Fig. 15); propodeum without distinct median carina.

*Legs*. Length of hind femur, tibia and basitarsus 4.4, 4.5 and 5.2 times their width, respectively; hind tibia rather slender and ventrally moderately curved (Fig. 17); fore coxa close to mesopleuron; hind coxa coarsely transversely rugose antero-dorsally, silvery pilose and ventrally coriaceous; hind basitarsus rather stout and 0.8 times as long as remainder of tarsus without claws (Fig. 17), widened basally in dorsal view.

*Metasoma*. Ovipositor sheath 1.1 times as long as body, 1.8 times as long as metasoma, 5.7 times as long as hind tibia and 3.6 times as long as hind tibia and tarsus combined; dark ivory apical part of ovipositor sheath 0.3 times as long as hind basitarsus.

*Colour.* Black; tegulae pale yellowish; mandible (including base except dorsal corner), clypeus largely, pronotum, propleuron, mesoscutum, scutellum posteriorly, fore coxa mainly, mesopleuron dorsally, second-fifth tergites, sternites (but hypopygium dark brown except basally) orange brown; bases and apices of fore and middle tibiae ivory; fore and middle tarsi, base of hind tibia and pterostigma medially brown; remainder of legs and first tergite dark brown; pterostigma laterally and veins dark brown; fifth and following antennal segments (except apical dark brown segment) brownish ventrally; wing membrane subhyaline.

*Male.* Very similar to female, but mandible and sternites medially dark brown or black. Third antennal segment 1.6 times as long as second segment, fourth segment twice as long as third segment and 1.2 times as long as second and third segments combined, fifth segment 0.9 times as long as fourth segment (Fig. 28); hind tibia and basitarsus entirely dark brown or blackish; apex of paramere black (Fig. 24).

*Variation.* Length of body of ♀ 12.5–17.3 mm (of ♂ 10.3–14.1 mm); mandible yellowish or orange brown or dark brown basally; mesosoma entirely black to anterior half largely orange brown; ovipositor sheath 1.1–1.2 times as long as body, 1.6–1.8 times as long as metasoma, 5.0–5.7 times as long as hind tibia and 3.6–4.3 times as long as hind tibia and tarsus combined; pale brown or ivory apical part of ovipositor sheath 0.2–0.5 times as long as hind basitarsus.

#### Distribution.

Iran, Greece, Jordan, Syria, Turkey, Russia.

#### Biology.

Unknown. Collected in April-September.

#### Etymology.

Named after “agrenon”, (Greek for “net”) because of the reticulate sculpture of the mesoscutum.

#### Notes.

Examined from Turkey a pale specimen with frons and vertex with satin sheen, hind tibia subbasally and hind basitarsus largely ivory, ovipositor sheath about 4 times as long as hind tibia and middle and hind coxae reddish brown which may belong to this species.

**Figures 23–28.**
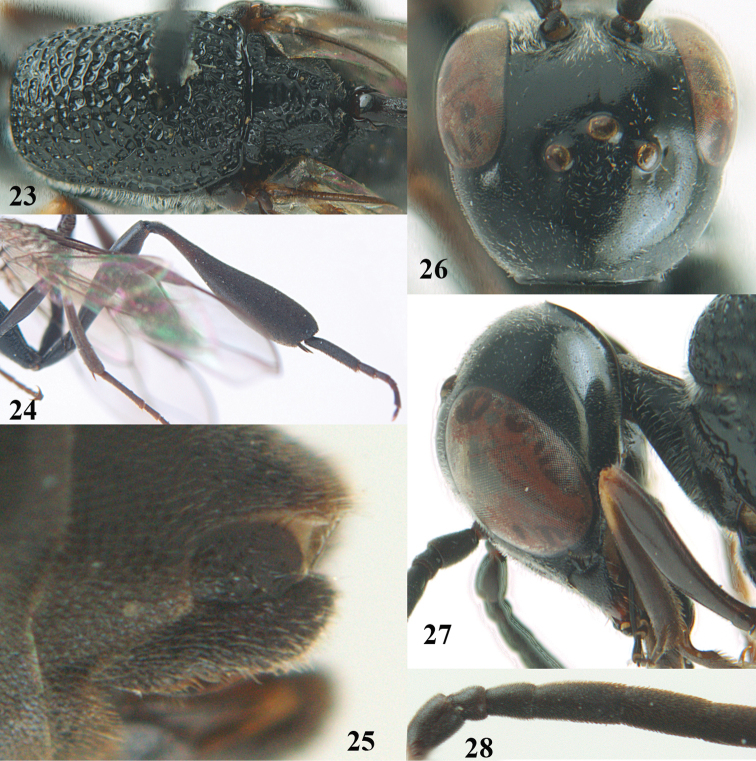
*Gasteruption agrenum* sp. n., male, paratype. **23** mesonotum dorsal **24** hind leg **25** male genitalia **26** head dorsal **27** head lateral **28** basal antennal segments.

### Gasteruption assectator

Taxon classificationAnimaliaHymenopteraGasteruptiidae

(Linnaeus, 1758)

Figs 29 –47

Ichneumon assectator Linnaeus, 1758: 566, 1761: 407, 1767: 937; Scopoli 1763: 287; Fabricius 1775: 340, 1781: 435, 1787: 268; Gmelin 1790: 2696; Villers 1789: 174; Rossi 1790: 90; Christ 1791: 375; Petagna 1792: 365; Cederhjelm 1798: 163; Schrank 1802: 263; Hentschius 1804: 112; Illiger 1807: 74; Roman 1932: 2; Hedqvist 1973: 182; Fitton 1978: 376.
Foenus assectator ; Fabricius 1798: 240; Walckenaer 1802: 75; Latreille 1805: 195; Dahlbom 1831: 77; Curtis 1832: 423; Nees 1834: 308; Stephens 1835: 121; Labram and Imhoff 1836: 24; Zetterstedt 1840: 408; Westwood 1843: 255; Taschenberg 1866: 93; Tournier 1877: ix (as *affectator*); Thomson 1883: 849.
Faenus affectator ; Abeille de Perrin 1879: 265, 266, 277.
Gasteruption assectator ; Schletterer 1885: 276, 316, 1889: 384, 393, 395, 397; Dalla Torre 1902: 1063; Szépligeti 1903: 370 (as *affectator*); Kieffer 1912: 256 (id.); Lindemans 1921: 298 (id.); Roman 1932: 2; Schmiedeknecht 1930: 380, 383 (as *affectator*); Hedicke 1939: 5 (id.); Ferrière 1946: 235, 238, 240 (id.); Leclercq 1948: 75; Hellén 1950: 4; Townes 1950: 123–128; Šedivý 1958: 36, 37; Györfi and Bajári 1962: 48, 51; Schmidt 1969: 293; Hedqvist 1973: 181; Fitton 1978: 376; Dolfuss 1982: 22; Oehlke 1984: 169, 171, 175; Ortega and Baez 1985: 509, 515; Madl 1987a: 401, 1987b: 21, 1988c: 37, 1989a: 159, 1989b: 41, 1990a: 127, 1990b: 480; Kozlov 1988: 245, 247; Kofler and Madl 1990: 320; Narolsky and Shcherbal 1991: 23, 24; Wall 1994: 150; Scaramozzino 1995: 3; Smith 1996: 492; Peeters 1996: 134; Neumayer et al. 1999: 220; Pagliano and Scaramozzino 2000: 11, 19; Saure 2001: 29; Yildirim et al. 2004: 1350; Turrisi 2004: 84; Westrich 2008: 7–8; van der Smissen 2010: 372; Zhao et al. 2012: 23–27; van Achterberg 2013: 82.
Gasteryption affectator ; Semenov 1892: 200.
Ichneumon annularis Geoffroy in Fourcroy, 1785: 398; Hedicke 1939: 7; Wall 1994: 148. Synonymized by with *Gasteruption assectator* (Linnaeus) by Olivier, 1792.
Foenus fumipennis Thomson, 1883: 848; Hedicke 1939: 7; Hedqvist 1973: 181, 182 (lectotype designation); Wall 1994: 148. Synonymized with *Gasteruption assectator* (Linnaeus) by Schletterer, 1885.
Foenus nigritarsis Thomson, 1883: 849; Schletterer 1889: 398; Hedicke 1939: 7; Hedqvist 1973: 181, 182 (lectotype designation); Wall 1994: 149. Synonymized with *Gasteruption assectator* (Linnaeus) by Schletterer, 1889.
Gasteruption nigritarse ; Schletterer 1885: 310.
Gasteruption brevicauda Kieffer 1904a: 648, 1904b: 18; 1912: 259; Hedicke 1939: 8; Madl 1987a: 401; Wall 1994: 148. Synonymized with *Gasteruption assectator* (Linnaeus) by Madl, 1987a.
Trichofoenus breviterebrae Watanabe, 1934: 285; Hedicke 1939: 45. Synonymized by Pagliano and Scaramozzino 2000: 11, 19.
Gasteruption rugulosum ; Malyshev 1965: 245.
Gasteruption affectator auct.

#### Note.

The Nearctic synonyms as given by Smith (1996) are not repeated here and need reconfirmation.

#### Type material.

Lectotype of *Gasteruption assectator* here designated, ♀ coll. no. 2652 in the Linnean Society London, “49, *assectator*”, and examined by Roman (1932) and Fitton (1978). The lectotype has been studied digitally (www.linnean-online.org) by the first author; no. 2653 is damaged and is a paralectotype. Lectotype of *Gasteruption nigritarsis* ♀ (ZIL) from “Lund, 8/53 [= viii.1853]”, “*nigritarsis*”, “Lectotypus *Foenus nigritarsis* Thoms., ♀, K.-J. Hedqvist, det. 1972”. Lectotype of *Gasteruption fumipenne* ♀ (ZIL) without metasoma from “Olle Han [? name of collector, according to original description from Gottland and Skäne, Sweden], d.15.vii.1850”, “*fumipennis*”, “Lectotypus *Foenus fumipennis* Thoms., ♀, K.-J. Hedqvist, det. 1972”; lectotype has spurious vein on vein 1-SR. Holotype of *Gasteruption margotae* in Zoological Museum Helsinki (from Finland, Suoniemi, 26. vi.1947) was re-examined by Madl (1990b). Holotype of *Gasteruption brevicauda* in MNHN, female from Algeria (Orléansville) was examined by Madl (1987a).

#### Additional material.

*Iran (Alborz, Chalous Road Shahrestanak; id., Arangeh; Qazvin, Zereshk Road; Azer. e Sh., Sis, 10 km E of Shabestar, 1540 m); **Turkey** (Anatolia, Lycia, Kemer; Pasli, 50 km S of Kars; 10 km W of Ürgüp; Bursa, near Cagliyan; 15 km W of Refahye, W of Erzincan, 1600 m; Konya, 10 km S of Aksehir Mts.; id., 30 km S of Aksehir; Sakarya, near Karasu; near Agri; near Akyaka, 40 m; near Fethiye; 40 km N of Muradye, 2200 m; Aciöl, near Cardak; Trabzon, near Macka; Avgadi, 30 km NW of Erdemli, 1300 m; Denizli, 10 km NE of Denizli, 270 m; Mansisa, 15 km SEE of Salihli, 170 m; Van, 30 km N of Baskale, 2700 m; Hakkari, Mt. Sat, SW of Yüksekova, Varegös, 1650 m; Nevsehir, Ürgüp, 1100 m; Gümüshane, Köse Dagh Gecidi, 1700 m; Pirene).

#### Diagnosis.

**Figures 29–36.**
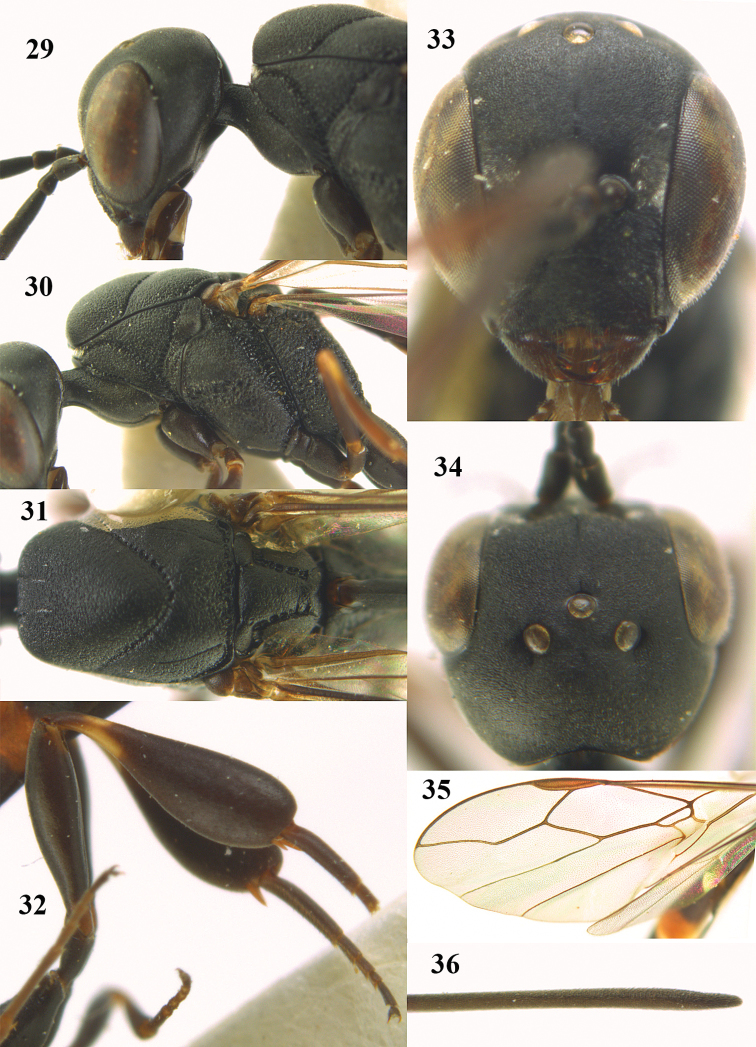
*Gasteruption assectator* (Linnaeus), female, Netherlands. **29** head lateral **30** mesosoma lateral **31** mesonotum dorsal **32** hind leg **33** head anterior **34** head dorsal **35** fore wing **36** apex of ovipositor sheath.

Apex of ovipositor sheath blackish or slightly brownish; ovipositor sheath 0.8-1.3 times as long as hind tibia and 0.4–0.8 times as long as hind tibia and tarsus combined; occipital carina obsolescent medio-dorsally (Figs 29, 39) and rather protruding ventro-posteriorly (Fig. 29); antesternal carina narrow; head, laterally mesosoma and scapus black; head in anterior view slightly protruding below lower level of eyes by less than basal width of mandible and mandibular condylus near lower level of eyes (Fig. 33); in lateral view condylar incision of malar space close to eye (Fig. 29); clypeus with small depression or depression obsolescent; eyes shortly setose; fourth and fifth antennal segment 1.2–1.3 and 1.0–1.1 (♀)-1.3 (♂) times as long as third segment, respectively (Figs 29, 38); apical antennal segment at most 1.2 times as long as third antennal segment and its colour similar to colour of medial segments; antenna of female may be partly yellowish-brown; mesoscutum and head similarly coriaceous (Fig. 30), at most mesoscutum superficially rugulose; hind coxa often transversely rugose dorsally, but sometimes mainly coriaceous; hind tibia stout, with a distinct subbasal ivory ring and swollen, resulting in a distinctly convex ventral border (Fig. 32); hind basitarsus rather long (Fig. 32); hind tibial spurs yellowish-brown or brown; hind tarsus brown, dark brown or blackish; incision of hypopygium shallow.

#### Distribution.

Holarctic, Turkey, Iran. New for the fauna of Iran.

#### Biology.

Predator-inquiline of *Hylaeus* spp. and small Megachilinae. Collected in June–August.

**Figures 37–47.**
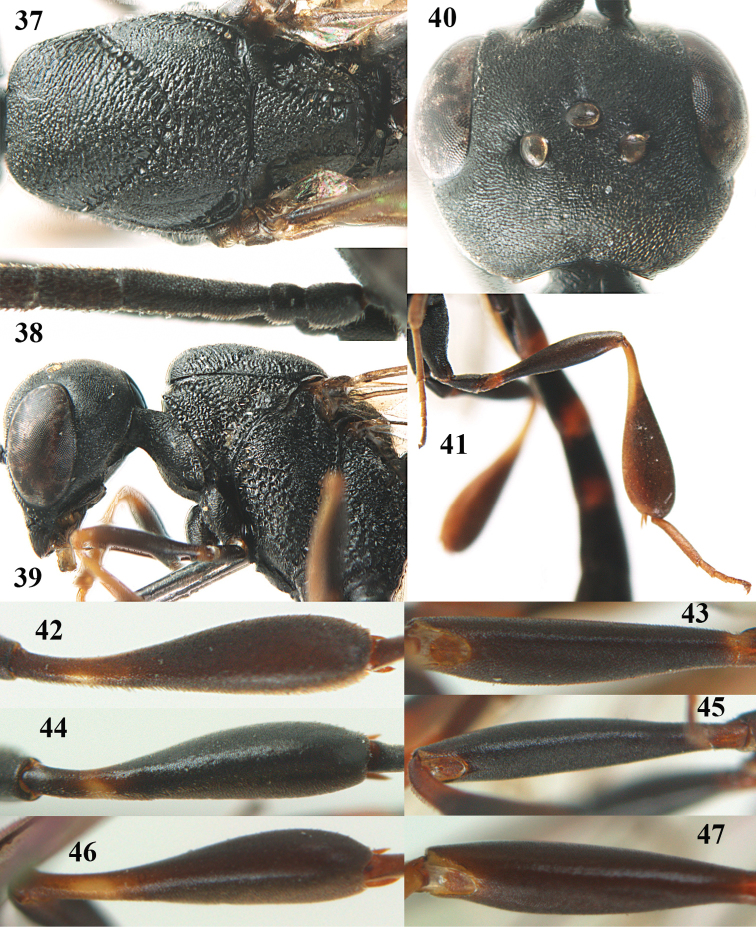
*Gasteruption assectator* (Linnaeus), male, Sweden, but **42–45** from Austria and **46–47** of female from Austria. **37** mesonotum dorsal **38** basal antennal segments **39** head lateral **40** head dorsal **41** hind leg **42,44, 46** hind tibia dorsal **43, 45, 47** hind femur latero-ventral.

### Gasteruption brevibasale

Taxon classificationAnimaliaHymenopteraGasteruptiidae

van Achterberg & Saure sp. n.

http://zoobank.org/A4C553E7-143F-4A56-89D3-6225B626DC7F

Figs 48 –62

#### Type material.

Holotype, ♀ (RMNH), “TR [= **Turkey**], 54 km W [of] Kayseri, Göreme, 38°39'N, 34°52'E, 17.vii.1998, TR-nevA, [C.] Schmid-Egger”. Paratypes (5 ♀): 1 ♀ (CSEC), same label data; 4 ♀ (BZL, RMNH) “Turkey, 15 km W [of] Refahye, W of Erzincan, 1600 m, 7.vii.2000, M. Halada”.

#### Diagnosis.

**Figures 48–58.**
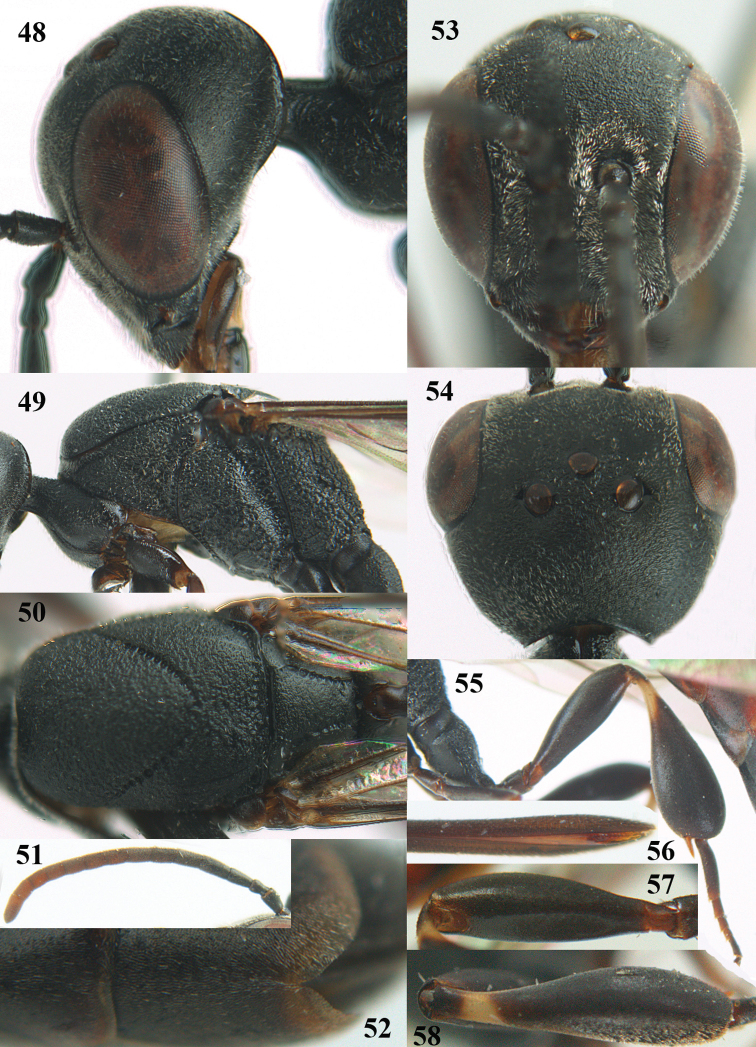
*Gasteruption brevibasale* sp. n., female, holotype. **48** head lateral **49** mesosoma lateral **50** mesonotum dorsal **51** antenna **52** hypopygium ventral **53** head anterior **54** head dorsal **55** hind leg **56** apex of ovipositor sheath **57** hind femur latero-ventral **58** hind tibia dorsal.

**Figures 59–62.**
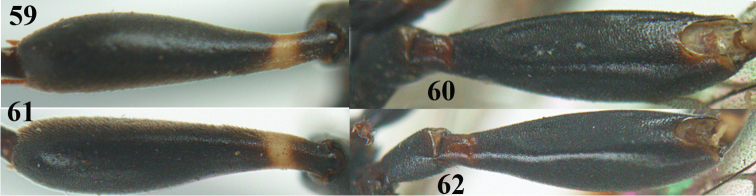
*Gasteruption brevibasale* sp. n., female, paratypes. **59, 61** hind tibia dorsal **60, 62** hind femur latero-ventral.

Head in front of occipital carina without depression (Fig. 54), in lateral view slightly convex dorsally and occipital carina narrow medio-dorsally and non-lamelliform (Fig. 48); vertex and frons matt, finely and densely coriaceous; antesternal carina narrow and non-lamelliform, antesternal carina and prepectal carina medio-ventrally similarly developed (Fig. 49); head subquadrate and gradually narrowed behind eyes in dorsal view and temples convex (Fig. 54); temple 0.9 times as long as eye in dorsal view; fourth and fifth antennal segments of ♀ 1.2 and 1.1 times as long as third segment, respectively; fourth segment of ♀ 0.7 times as long as second and third segments combined; apical antennal segment 1.8 times as long as penultimate segment; head not protruding below eyes and malar space 0.3 times length of second antennal segment and 0.2 times basal width of mandible and mandibular condylus close to lower level of eyes (Fig. 53); mandible dark brown and with deep basal depression; eye setose; propleuron stout, with satin sheen, 0.7 times as long as mesoscutum in front of tegulae and coriaceous (Fig. 49); antero-lateral teeth of pronotum absent; mesoscutum stout and inconspicuously setose (Fig. 50), anteriorly truncate, matt and largely finely and densely coriaceous; hind femur short and widened (Fig. 55), ventro-basally slightly depressed (Figs 57, 60, 62); hind coxa matt and coriaceous (but rugulose postero-dorsally); hind tibia inflated, its basal petiolar part short and wide in dorsal view (Figs 58, 59, 61); ovipositor sheath 0.4 times as long as metasoma, 0.7 times as long as hind tibia and tarsus combined and 1.0–1.2 times as long as hind tibia; apex of ovipositor sheath dark brown; length of body 9–10 mm.

The new species shares with *Gasteruption paglianoi* the widened hind femur and apically yellowish brown antenna; the new species has the hypopygium (except apically) dark brown (entirely pale yellowish brown in *Gasteruption paglianoi*), the hind femur slightly depressed ventro-basally (slightly convex) and the short and widened basal petiolar part of the hind tibia (medium-sized and narrower).

#### Description.

Holotype, female, length of body 9.0 mm (of fore wing 4.9 mm).

*Head*. Vertex and frons matt, finely and densely coriaceous and in front of occipital carina without medial depression (Fig. 54), in lateral view slightly convex and occipital carina narrow medio-dorsally and non-lamelliform (Fig. 48); head subquadrate and gradually narrowed behind eyes in dorsal view, convex laterally (Fig. 54); temple 0.9 times as long as eye in dorsal view; fifth antennal segment 1.1 times as long as third segment; clypeus nearly flat medially and only near ventral margin impressed; head not protruding below eyes and malar space 0.3 times as long as second antennal segment (Fig. 53).

*Mesosoma*. Length of mesosoma 1.7 times its height; pronotal side high, largely coriaceous with faint rugulae, matt and grooves narrow and distinct; antero-lateral teeth of pronotum absent; propleuron with satin sheen and coriaceous, 0.7 times as long as mesoscutum in front of tegulae and stout (Fig. 49); antesternal carina non-lamelliform, antesternal carina and prepectal carina medio-ventrally similarly developed (Fig. 49); mesoscutum stout and inconspicuously setose (Fig. 50), anteriorly truncate, matt and largely finely and densely coriaceous; scutellum similarly but more superficially sculptured than mesoscutum.

*Legs*. Hind coxa matt and mainly coriaceous, but rugulose postero-dorsally; hind femur distinctly widened (Fig. 55) and ventro-basally slightly concave (Figs 57, 60, 62); hind tibia strongly inflated, 3.3 times as long as hind femur and its basal petiolar part short and widened in dorsal view (Figs 58, 59, 61); length of hind femur, tibia and basitarsus 3.1, 3.0 and 3.9 times their width, respectively, and with very short setae; hind basitarsus widened basally in dorsal view and as long as remainder of tarsus; pale hind tibial spurs contrasting with dark brown hind basitarsus.

*Metasoma*. Ovipositor sheath 0.4 times as long as metasoma, 0.7 times as long as hind tibia and tarsus combined and 1.2 times as long as hind tibia; hypopygium shallowly incised (Fig. 52).

*Colour.* Black or blackish-brown; tegulae, palpi, pterostigma, ovipositor sheath (including apex), metasoma (but second-fourth tergites orange brown apically) and legs dark brown, but base of fore and middle tibiae and subbasal band of hind tibia ivory; hind tibial spurs yellowish-brown; apex of hypopygium pale brown; wing membrane subhyaline.

*Male.* Unknown.

*Variation*. Length of body of ♀ 9.0–9.8 mm; length of ovipositor sheath 1.0–1.2 times as long as hind tibia; apical 0.3–0.7 of antenna yellowish brown; coxae dark brown or black.

#### Distribution.

Turkey.

#### Biology.

Unknown. Collected in July.

#### Etymology.

Named after the short petiolate base of the hind tibia; “brevis”, is Latin for “short”, and “basis”, is Latin for “base”.

### Gasteruption caucasicum

Taxon classificationAnimaliaHymenopteraGasteruptiidae

(Guérin-Méneville, 1844)

Figs 63 –79

Foenus caucasicus Guérin-Méneville, 1844: 406.
Faenus caucasicus ; Abeille de Perrin 1879: 278.
Gasteruption caucasicum ; Schletterer 1885: 304; Kieffer 1912: 265; Hedicke 1939: 8; van Achterberg 2013: 82.
Foenus pedemontanus Tournier, 1877: vii.
Faenus pedemontanus ; Abeille de Perrin 1879: 263, 265, 268.
Gasteruption pedemontanum ; Schletterer 1885: 282, 1889: 382, 388, 394, 395, 413; Dalla Torre 1902: 1070; Szépligeti 1903: 367; Kieffer 1902: 9, 1904a: 407, 1912: 248; Lindemans 1921: 297–298; Schmiedeknecht 1930: 376, 380; Hedicke 1939: 18; Hellén 1950: 3; Ferrière 1946: 237, 238, 246; Stohl 1947a: 1, 1947b: 275; Leclercq 1948: 77; Crosskey 1951: 291; Šedivý 1958: 35, 36, 41; Györfi and Bajári 1962: 46, 49; Schmidt 1969: 295; Hedqvist 1973: 186; Dolfuss 1982: 24; Oehlke 1983: 168, 171, 180; Madl 1987a: 404, 1987b: 24, 1988a: 13, 16, 1989a: 161, 1989b: 44, 1990a: 129, 1990b: 480, 483; Kozlov 1988: 246, 247; Kofler and Madl 1990: 322; Peeters 1996: 134; Narolsky and Shcherbal 1991: 24; Wall 1994: 161; Scaramozzino 1995: 3; Neumayer et al. 1999: 220; Pagliano and Scaramozzino 2000: 15, 17, 32; Saure 2001: 29; Yildirim et al. 2004: 1351; van der Smissen 2010: 373; van Achterberg 2013: 82 (synonymized with *Gasteruption caucasicum*).
Gasteryption pedemontanum ; Semenov 1892: 206.
Foenus terrestris Tournier, 1877: viii; Thomson 1883: 847; Wall 1994: 149 (as synonym of *Gasteruption pedemontanum* (Tournier)). **Syn. n.**
Foenus terrestre ; Hedqvist 1973: 186 (as synonym of *Gasteruption pedemontanum* (Tournier).
Faenus terrestris ; Abeille de Perrin 1879: 263, 265, 269.
Gasteruption terrestre ; Schletterer 1885: 284, 1889: 382, 388, 394, 395, 414; Dalla Torre 1902: 1073; Szépligeti 1903: 368; Kieffer 1912: 247; Schmiedeknecht 1930: 376, 380; Hedicke 1939: 22; Ferrière 1946: 247 (as synonym of *Gasteruption pedemontanum* (Tournier)); Hellén, 1950: 3 (id.); Šedivý 1958: 41 (id.).
Gasteryption terrestre ; Semenov 1892: 206.
Gasteruption trifossulatum Kieffer 1904b: 557; 1912: 268; Hedicke 1939: 19 (as synonym of *Gasteruption pedemontanum* (Tournier)); Madl 1988c: 39 (as synonym of *Gasteruption pedemontanum* (Tournier)); Wall 1994: 149. **Syn. n.**
Gasteruption ignoratum Kieffer, 1912: 248; Hedicke 1939: 14. **Syn. n.**

#### Type material.

Holotype of *Gasteruption caucasicum* ♀ (MCG), “*Foenus caucasicus* Guer. Ic. R.A. [= Iconographie du Règne Animal de Georges Cuvier], (type), Caucase, Motschulsky”, “Museo Genova, coll. G. Gribodo (asquisto 1924)”. Holotype of *Gasteruption pedemontanum*, ♀ (MHNG), “[Italy], Aosta, [Piemonte], 6.vii.[18]76”, “Cn Tournier”, “Type”, *Foenus pedemontanum* Tourn., ♀”. Lectotype of *Gasteruption terrestris* here designated, ♀ (MHNG), “[Switzerland], P. [= Peney, near Genève], 16.viii.[18]76”, “Cn Tournier”, “Type”, “*Foenus terrestris* Tourn., ♀”, “Lectotypus, des. Madl, 1987”, “*Gasteruption pedemontanum* Tour., det. Madl, 1986”; paralectotypes: topotypic, 1 ♀ (MHNG) with same date of collecting as lectotype and 1 ♂ (MNHG) collected 21.vi.1875. Holotype of *Gasteruption trifossulatum* Kieffer ♂ (NHRS) from Egypt examined by Madl 1988c. Type series of *Gasteruption ignoratum* consists of 2 ♂ + 2 ♀ from S France identified by Abeille de Perrin as *Foenus terrestris*; lectotype ♂ (MNHN) from Marseille here designated, “Museum Paris, coll. Abeille de Perrin, 1919”, “*terrestris* Tourn., Mll.”; the metasoma is missing.

#### Additional material.

**Iran** (Tehran, Shahriar, Chalous Road; id., Shahrestanak; Gilan, Astaneh, Eshman Kamachal; Roodsar, Ziaz; Qazvin, Zereshk Road); **Turkey** (Hakkari, Mt. Sat, Varegös, SW of Yüksekova, 1700 m; id., Akcali, 35 km S of Hakkari, 1700 m; Birecik, Halfeti; Erkenek, 80 km SW of Malatya; Sultan Daglari, near Yalvac; Antalya, east of dunes; Mersin, Kuzucubelen; Bursa, near Caglian; Denizli, 10 km NE of Denizli, 290 m; Burdur, 20 km SW of Burdur, 940 m; Hakkari, Akcali, 35 km S of Hakkari, 1700 m; near Akyaka, 40 m; Mansisa, 35 km SEE of Salihli, 900 m; Fethiye, Mugla; Mugla, near Göktepe; Anatalya, 5 km W of Manavgat, Side, 10 m; id., 10 km SW of Manavgat, 50 m; Marmaris; Bitez, 8 km W of Bodrum; Adana, 10 km S of Karatepe, 200 m).

#### Diagnosis.

**Figures 63–70.**
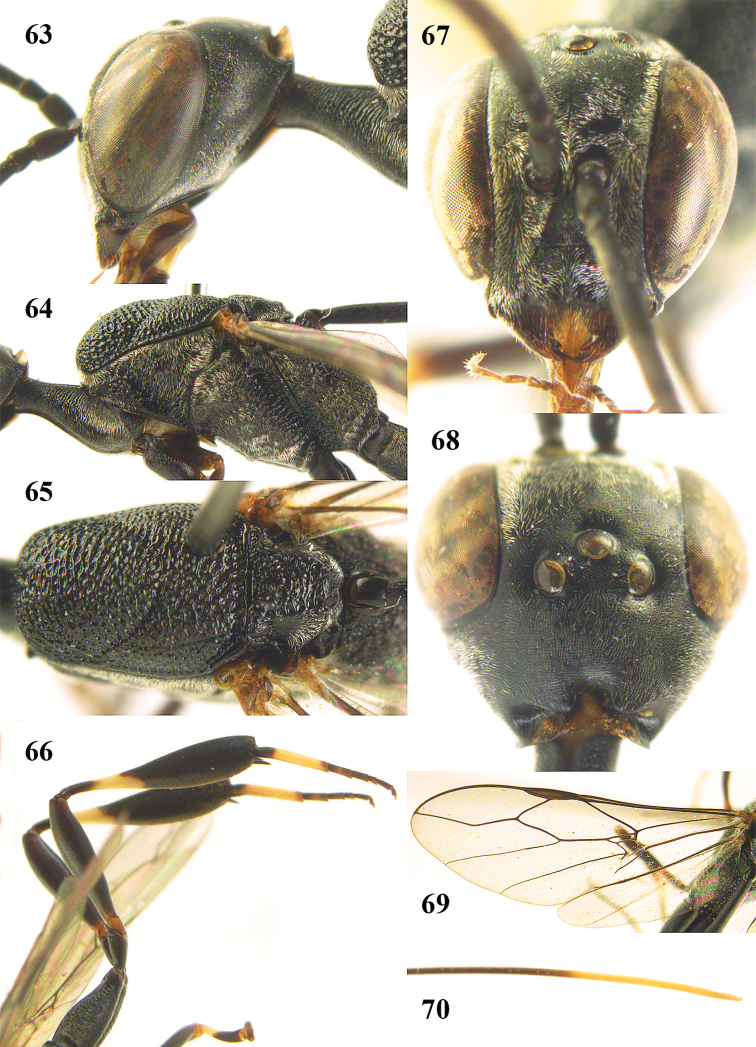
*Gasteruption caucasicum* (Guérin-Méneville), female, Turkey. **63** head lateral **64** mesosoma lateral **65** mesonotum dorsal **66** hind leg **67** head anterior **68** head dorsal **69** fore wing **70** apex of ovipositor sheath.

Apex of ovipositor sheath with a distinct white or ivory band, 0.7–1.6 times as long as hind basitarsus; head with middle depression in front of occipital carina very deep and wider than long and with two more or less developed lateral depressions (Figs 63, 68, 72, 76), rarely lateral depressions obsolescent; occipital carina distinctly lamelliform and medium-sized to wide (Figs 63, 72); antesternal carina rather wide and lamelliform, distinctly elevated above mesosternum (Fig. 64); frons densely punctulate or densely very finely aciculate and without distinct interspaces; vertex more or less finely transversely aciculate and with satin sheen; head in dorsal view strongly narrowed behind eyes (Fig. 68); propleuron distinctly shiny and with distinct rugulae; ovipositor sheath about 1.2 times as long as body and about 1.8 times as long as metasoma; pterostigma dark brown medially. Small specimens have scutellum largely smooth.

#### Distribution.

Europe, Caucasus, Iran, Turkey. One of the first two species of *Gasteruption* reported from Iran (Semenov 1892; as *Gasteruption pedemontanum*) and originating from NE Iran (Gorgan, Astrabad).

#### Biology.

Predator-inquiline of Colletinae (*Colletes* and *Hylaeus* spp.). Collected in May-September.

**Figures 71–79.**
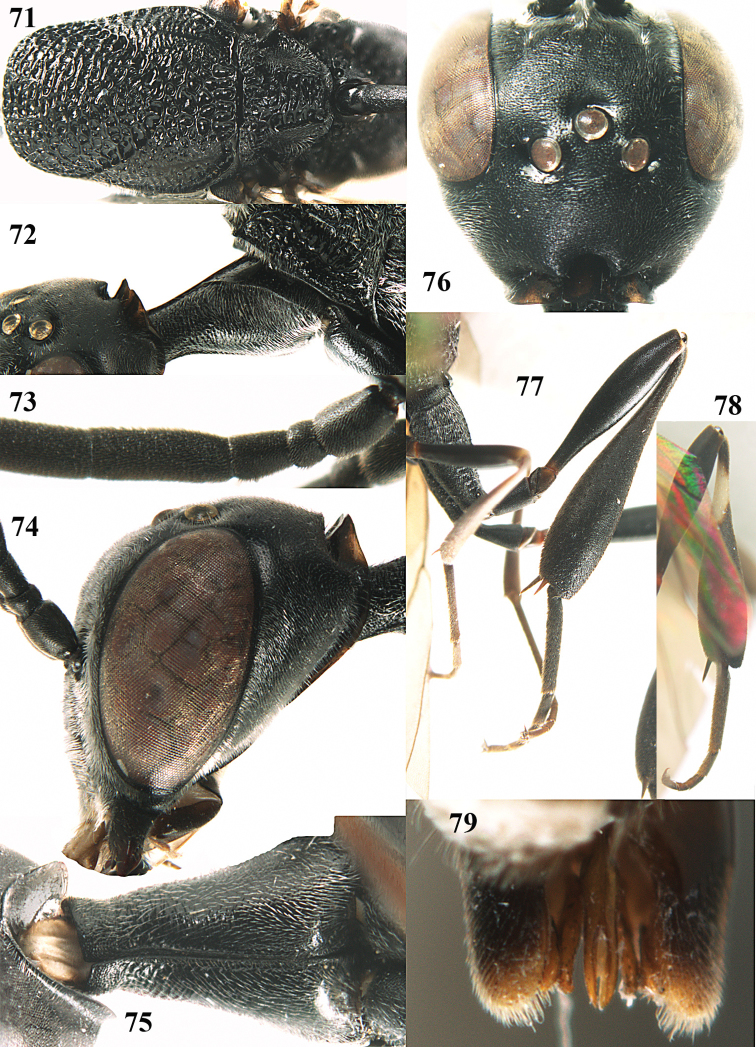
*Gasteruption caucasicum* (Guérin-Méneville), males, Iran. **71** mesonotum dorsal **72** head dorso-lateral **73** basal segments of antenna **74** head lateral **75** propleuron ventral **76** head dorsal **77, 78** hind leg **79** genitalia.

### Gasteruption coriacoxale

Taxon classificationAnimaliaHymenopteraGasteruptiidae

van Achterberg sp. n.

http://zoobank.org/AEC7621B-17AC-498D-B99B-F1C75E32C576

Figs 80 –100

#### Type material.

Holotype, ♀ (RMNH), “N. **Iran:** Tehran, Shahriar, M[alaise]T[rap] 25, 11–18.v.2010, M. Khayrandish, RMNH’12”. Paratypes (24 ♀ + 27 ♂): 1 ♂ (RMNH), same label data as holotype; 2 ♀ (RMNH), id., but 7–14.ix.2010; 1 ♀ + 3 ♂ (RMNH, TMUT), id., but 22–29.vi.2010; 1 ♀ (RMNH), id., but 18–25.v.2010; 2 ♂ (RMNH), id, but 5–13.x.2010; 2 ♀ (RMNH, TMUT), id., but 8–15.vi.2010, G 5 or G14; 1 ♀ (RMNH), id., 1–7.ix.2010, G11; 1 ♀ (RMNH), id, but MT 24, 14–20.vii.2010; 1 ♀ (TMUT), id, but 11–18.v.2010; 1 ♀ (RMNH), id, but 1–7.ix.2010; 1 ♀ + 1 ♂ (RMNH), id, but 22–28.ix.2010; 1 ♀ (TMUT), id, but 7–14.ix.2010; 1 ♀ + 1 ♂ (RMNH), id, but 8–15.vi.2010; 1 ♀ (RMNH), id, but 9–16.viii.2010; 1 ♀ + 3 ♂ (RMNH), id, but 15–22.vi.2010; 1 ♀ + 1 ♂ (RMNH, TMUT), id, but 1–8.vi.2010; 1 ♂ (RMNH), id, but 4–11.v.2010; 1 ♂ (RMNH), id, but 29.vi.–6.vii.2010; 2 ♂ (RMNH, TMUT), id., but 28.ix.-5.x.2010; 1 ♀ + 2 ♂ (RMNH), id, but 5–13.x.2010; 1 ♂ (RMNH), id, but 29.vi.-6.vii.2010; 1 ♀ + 1 ♂ (RMNH), id, but 15–22.vi.2010; 2 ♂ (RMNH, TMUT), id., but 15–22.vi.2010; 1 ♀ (RMNH), “N. Iran: Alborz, Karaj, MT 27, 22–28.ix.2010, M. Khayrandish, RMNH’12”; 1 ♀ (RMNH), id, but 15–22.vi.2010, G4; 1 ♀ (RMNH), id, but 5–13.x.2010; 1 ♂ (RMNH), “N. Iran: Qazvin, Zereshk Road, MT5, 7–22.vi.2011, A. Nadimi, RMNH’12”; 3 ♂ (RMNH, TMUT), “N. Iran: Qazvin, Zereshk Road, MT 3 or 5, 26.v.–9.vi.2011, A. Nadimi, RMNH’12”; 1 ♂ (RMNH), id, but 28.vii.-18.viii.2011; 1 ♀ (BZL), “Iran cent., Pasargad env., 8.v.1999, K. Deneš sen.”; 1 ♀ (BZL), “**Turkey**, 15 km E Malatya, 27.vi.2000, M. Halada”; 1 ♀ (BZL), “Turkey, 80 km SW Malatya, Erkenek, 9.vii.[19]97, Ma. Halada”; 1 ♀ (BZL), “Turkey, 20 km W Van, 5.vii.1997, Ma. Halada”.

#### Diagnosis.

Head evenly convex dorsally, in front of occipital carina without medio-posterior depression; face rather narrow (Fig. 84); frons and vertex superficially coriaceous and with satin sheen (Fig. 85), frons with medium-sized punctures; occipital carina non-lamelliform medio-dorsally and dark brown; mandible dark brown basally; propleuron 0.8 times as long as mesoscutum in front of tegulae; antesternal carina narrow and non-lamelliform; middle lobe of mesoscutum with satin sheen, protuberant and coriaceous, medially finely transversely rugulose and with more or less isolated and hardly visible punctures, medio-posteriorly with some rugae and lateral lobe mainly finely coriaceous (Fig. 82); scutellum finely coriaceous; mesosoma laterally largely silvery pilose (Fig. 81); hind basitarsus entirely dark brown; hind tibia rather swollen and subbasally pale brown (Fig. 83), ivory in male (Figs 91, 98); ovipositor sheath 0.9–1.2 times as long as body, 1.4–1.7 times as long as metasoma and 4.8–6.5 times hind tibia; white or ivory apical part of ovipositor sheath 0.2–0.7 (rarely 1.0) times as long as hind basitarsus (Fig. 88); length of body 7–13 mm; paramere dark brown apically (Fig. 94).

Close to *Gasteruption schlettereri* Magretti, but the new species has the antesternal carina non-lamelliform (rather narrow lamelliform in *Gasteruption schlettereri*), the frons with medium-sized punctures (absent), the hypopygium pale brown apically (dark brown) and the hind basitarsus tricoloured (uni- and bicoloured of males and females, respectively).

Similar to the East Palaearctic *Gasteruption gracilis* Alekseev, 1995, and *Gasteruption dimidiatum* Semenov, 1892. The new species has the mesoscutum with small punctures anteriorly and with transverse rugae medio-posteriorly (entirely very finely coriaceous in *Gasteruption gracilis* and with large isolated punctures in *Gasteruption dimidiatum*), the hypopygium of female is black (orange-brown in *Gasteruption dimidiatum*), the pronotal side is at least partly conspicuously setose (inconspicuously shortly setose in *Gasteruption gracilis*) and the apex of the ovipositor sheath ivory (dark brown or yellowish-brown in *Gasteruption dimidiatum*). Resembles the Central Asian *Gasteruption praestans* Semenov & Kostylev, 1928, but the new species has the occipital carina non-lamelliform (narrow lamelliform in *Gasteruption praestans*), the apex of the ovipositor sheath ivory (dark brown) and the head rather slender (rather wide). Specimens with rather long parallel-sided head may be easily confused with the European *Gasteruption phragmiticola* Saure, 2006. The new species has the hind coxa coriaceous or finely rugulose dorsally (distinctly rugose (male) or rugulose (female) in *Gasteruption phragmiticola*), the face narrower (rather wide), the propleuron in ventral view slightly slenderer (less slender), and part of the punctures of the middle lobe of mesoscutum separated from rugulae or punctures obsolescent (punctures as far as differentiated connected to rugae). The head in dorsal view is subparallel-sided in *Gasteruption phragmiticola* and usually more narrowed in the new species, but sometimes also subparallel-sided in the new species.

#### Description.

Female, length of body 7.5 mm (of fore wing 3.7 mm).

**Figures 80–88.**
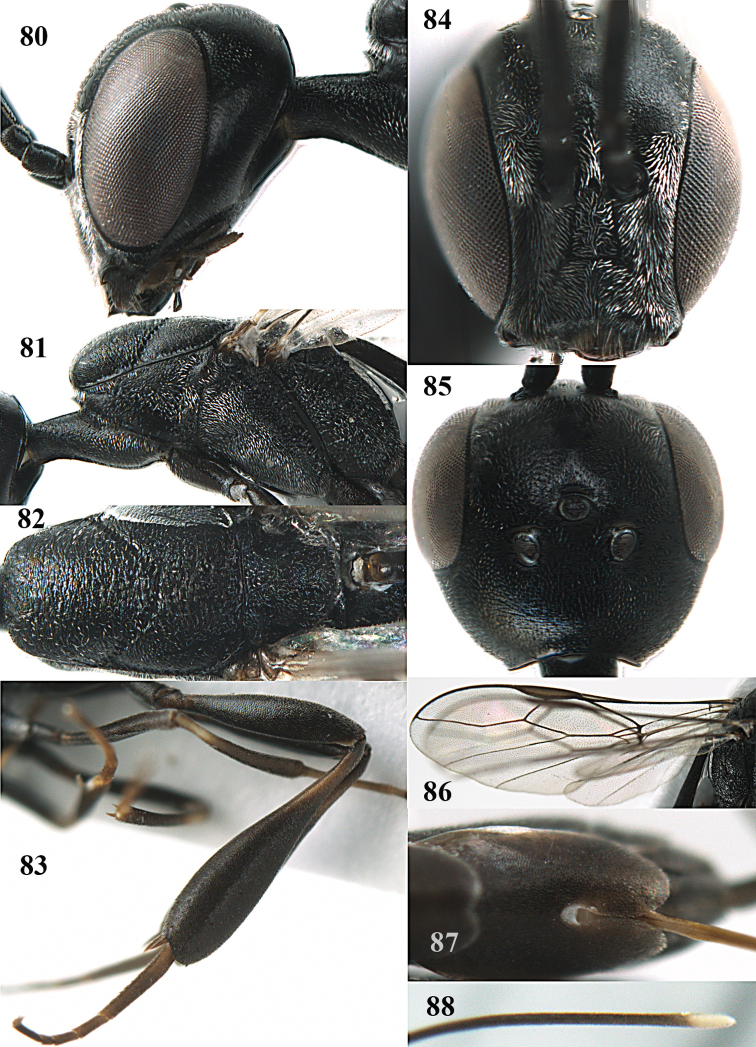
*Gasteruption coriacoxale* sp. n., female, holotype. **80** head lateral **81** mesosoma lateral **82** mesonotum dorsal **83** hind leg **84** head anterior **85** head dorsal **86** fore wing **87** hypopygium ventral **88** apex of ovipositor sheath.

*Head*. Head evenly convex dorsally, without medio-posterior depression; face, frons anteriorly and temples inconspicuously pilose; occipital carina non-lamelliform, dark brown (Fig. 80); third and fourth antennal segments 1.3 and 1.8 times as long as second segment, apical segment 1.7 times as long as penultimate segment; face rather narrow (Fig. 84); frons and vertex superficially coriaceous and with satin sheen (Fig. 85), frons with separate punctures; ventrally head not enlarged in anterior view (Fig. 84), malar space short, 0.3 times as long as second antennal segment.

*Mesosoma*. Length of mesosoma 1.8 times its height; propleuron 0.8 times as long as mesoscutum in front of tegulae, stout posteriorly; laterally pronotum entirely coriaceous except for crenulate grooves and partly pilose, with a small acute tooth antero-ventrally; antesternal carina non-lamelliform and narrow; middle lobe of mesoscutum with satin sheen, protuberant and coriaceous, medially finely transversely rugulose and with more or less isolated and hardly visible punctures, medio-posteriorly with some rugae and lateral lobe mainly finely coriaceous (Fig. 82); scutellum finely coriaceous; laterally most of mesosoma silvery pilose (Fig. 81).

*Legs*. Length of hind femur, tibia and basitarsus 4.7, 4.3 and 5.0 times their width, respectively; hind tibia rather swollen and ventrally curved (Fig. 83); fore coxa close to mesopleuron; hind coxa finely coriaceous dorsally; hind basitarsus moderately widened dorso-basally.

*Metasoma*. Ovipositor sheath 0.9 times as long as body, 1.4 times as long as metasoma, 2.8 times as long as hind tibia and tarsus combined and 4.8 times hind tibia; ivory apical part of ovipositor sheath 0.2 times as long as hind basitarsus; apical half of hypopygium incised.

*Colour.* Dark brown or blackish brown; mandible dark brown basally; trochantelli, palpi, tegulae, hind tibia basally and hind tarsus, brown; fore and middle tarsi pale brown; bases of fore and middle tibiae and apex of ovipositor sheath ivory; apex of second tergite of metasoma yellowish brown, apex of hypopygium dark brown; wing membrane subhyaline.

**Figures 89–95.**
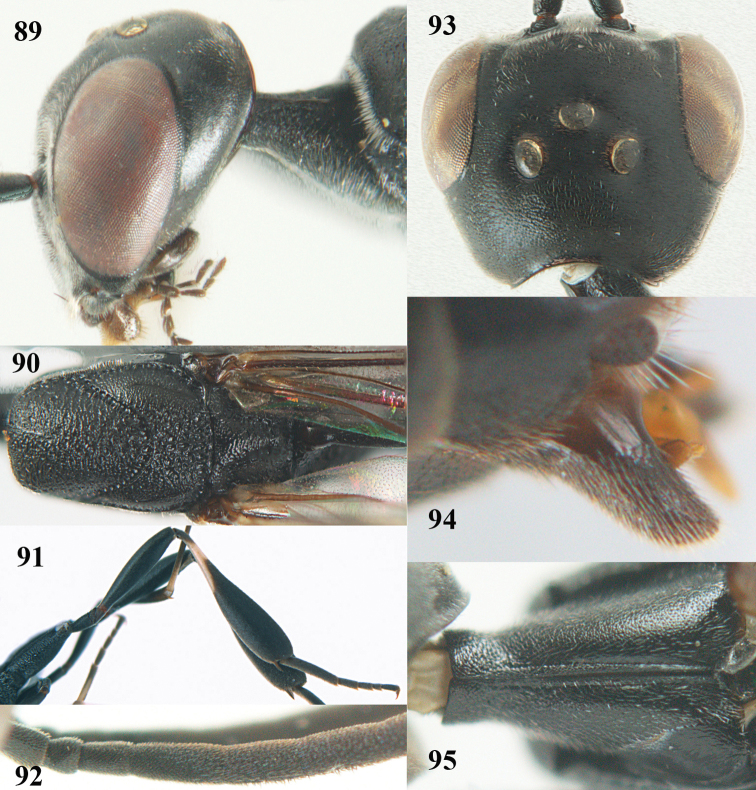
*Gasteruption coriacoxale* sp. n., dark form of male, paratype, but 95 of female paratype. **89** head lateral **90** mesonotum dorsal **91** hind leg **92** basal segments of antenna **93** head dorsal **94** genitalia **95** propleuron ventral.

**Figures 96–100.**
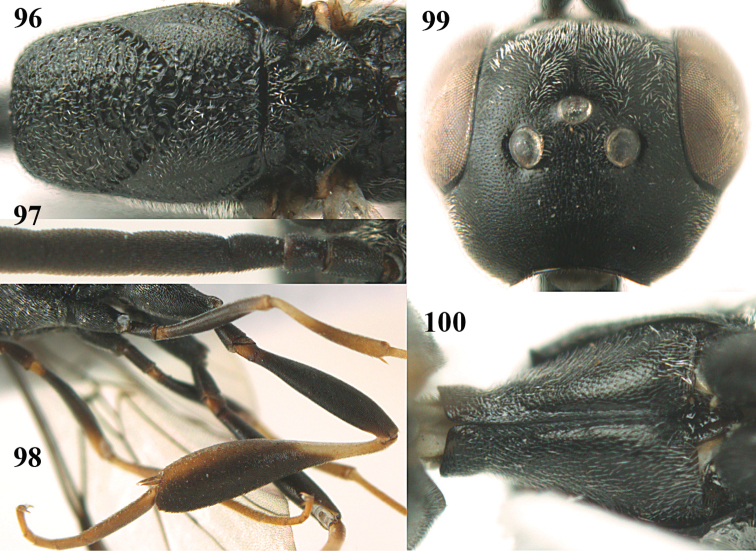
*Gasteruption coriacoxale* sp. n., pale form of male, paratype. **96** mesonotum dorsal **97** basal segments of antenna **98** hind leg **99** head dorsal **100** propleuron ventral.

*Male.* Very similar to female, but middle lobe of mesoscutum rugulose with some punctures to mainly rugose (Figs 90, 96), pronotal side with some rugulae ventrally, hind coxa usually rugulose dorsally and malar space nearly absent (Fig. 89). Third antennal segment 1.5–1.6 times as long as second segment, fourth segment 1.6–1.8 times third segment and 0.9–1.0 times as long as second and third segments combined, fifth segment about as long as fourth segment (Figs 92, 97); hind tibia dark brown and with wide subbasal white or ivory band, only ventrally white and dorsally ivory or pale brown (Figs 91, 98), rarely (as in holotype) brown subbasally; hind tibia usually dark brown ventrally (except subbasally), but more or less yellowish brown in pale specimens (Fig. 98); mandible usually dark brown basally, but sometimes yellowish basally; hind tarsus brown or dark brown; apex of paramere dark brown (Fig. 94).

*Variation.* Length of body of ♀ 7.5–13.4 mm (of ♂ 7.6–9.8 mm); variable in colour: dark forms (as holotype) have metasoma and mandible dark brown and hind tibia subbasally brown or rarely dark brown; pale forms have second-fourth tergites largely and fifth tergite partly orange brown and hind tibia ivory subbasally; most of specimens are intermediate, either mainly dark brown or black, some pale specimens have also the mandible yellowish brown basally and males have the hind tibia more or less yellowish-brown ventrally (Fig. 98). Vertex matt or with satin sheen; mesoscutum often with some large but shallow punctures medially; ovipositor sheath 0.9–1.2 times as long as body, 1.4–1.7 times as long as metasoma and 4.8–6.5 times hind tibia; white or ivory apical part of ovipositor sheath 0.2–0.7 (rarely 1.0) times as long as hind basitarsus; palpi brown or dark brown.

#### Distribution.

Iran, Turkey.

#### Biology.

Unknown. Collected in May-October.

#### Etymology.

Name derived from “coriaceus”, (Latin for “leathery”) and “coxis”, (Latin for “hip”) because of the leathery sculptured hind coxae.

#### Notes.

Especially small specimens are darker than large specimens and have usually a shorter ovipositor sheath.

### Gasteruption diversipes

Taxon classificationAnimaliaHymenopteraGasteruptiidae

(Abeille de Perrin, 1879)

Figs 101 –114

Faenus diversipes Abeille de Perrin, 1879: 264, 265, 272.
Gasteruption diversipes ; Schletterer 1885: 305, 1889: 408; Kieffer 1904a: 641, 1912: 255; Hedicke 1939: 9; Ferrière 1946: 237, 239, 245 (p.p.); Leclercq 1948: 77; Hellén 1950: 4; Šedivý 1958: 35, 36, 38; Györfi and Bajári 1962: 48, 49; Malyshev 1968: 49; Schmidt 1969: 294; Dolfuss 1982: 23; Oehlke 1984: 169, 172, 175; Madl 1987a: 402, 1987b: 21; 1988a: 13, 15, 1988b 404, 1989a 160, 1989b 41, 1990a 128, 1990b 480, 481; Kozlov 1988: 246, 247; Kofler and Madl 1990: 321; Wall 1994: 153; Scaramozzino 1995: 3; Neumayer et al. 1999: 220; Pagliano and Scaramozzino 2000: 13, 17, 21; Saure 2001: 29; Yildirim et al. 2004: 1350; Wisniowski 2004: 118; Turrisi 2004: 84; van der Smissen 2010: 372; Samin and Bagriacik 2012: 386–387; van Achterberg 2013: 82.
Gasteruption distinguendum Schletterer, 1885: 277; Kieffer 1904a: 649; Schmiedeknecht 1930: 377, 381; Hedicke 1939: 12 (as synonym of *Gasteruption granulithorax* (Tournier)); Schmidt 1969: 294 (as synonym of *Gasteruption diversipes* (Abeille de Perrin)); Hedqvist, 1973 (as synonym of *Gasteruption jaculator* (Linnaeus)); Wall 1994: 148. Synonymized (as *Gasteruption distingendum*) with *Gasteruption granulithorax* Tournier by Schletterer, 1889. Synonymized with *Gasteruption diversipes* (Abeille de Perrin) by Schmidt, 1969 and Oehlke, 1984.
Gasteruption granulithorax ; Schletterer 1885: 279, Schletterer 1889: 389, 395, 396, 427; Šedivý 1958: 35, 36, 39.
Gasteryption granulithorax ; Semenov 1892: 213.
Gasteruption dusmeti Kieffer, 1904a: 643, 1912: 263; Hedicke 1939: 9; Wall 1994: 148. Synonymized with *Gasteruption diversipes* (Abeille de Perrin) by Madl, 1987a.
Gasteruption kriechbaumeri var. striaticeps Kieffer 1904b: 551, 1912: 267; Hedicke 1939: 15; Madl 1988b: 404; Wall 1994: 148. Synonymized with *Gasteruption diversipes* (Abeille de Perrin) by Madl, 1988b.

#### Type material.

Lectotype of *Gasteruption diversipes* here designated, ♀ (MNHN) from S France, “Museum Paris EY 0000003926”, “Museum Paris, coll. Abeille de Perrin, 1919”, “*Gasteruption diversipes* Ab., ♀, det. Madl, 1987 / lectotypus des. Madl”, “Lectotypus, des. Madl, 1987”; according to the original description there are additional types from Province (rare), Marseille (common), Pyrenees, Languedoc and Gascoigne. Lectotype of *Gasteruption distinguendum* is here designated ♀ (NMW; in collection under *Gasteruption granulithorax*) “[Austria], Piesting, Tschek, 1872”; according to the original description there should be additional paralectotypes from Italy (Bozen, Triest, Fiume, Ragusa, Livorno), France (Versailles) and “Balkan”. Holotype of *Gasteruption dusmeti* ♀ (MNCN) “[Spain], Alcalá, Mz. Escalera”, “MNCN_Ent. Cat. No. 43293”, “Holotipo”, “*Gasteruption Dusmeti* K.”, “*Gasteruption diversipes* Abeille = *dusmeti* Kieff., n. syn., C. Rey det.”, “MNCN Cat. Tipos No. 2044”. Holotype of *Gasteruption striaticeps* ♂ (NHRS) “German. [Germany]”, “Mewe”, “Type”, “Gasteruption kriechbaumeri var. striaticeps”, “Riksmuseum Stockholm”, “*Gasteruption diversipes* Ab., det. Madl, 1986”, “NHRS-HEVA 000000009”; the metasoma and the hind legs are missing.

#### Additional material.

**Turkey** (Konya, Konya, Alaâdin hill, 1050m; Urfa, Halfeti, 400 m).

#### Diagnosis.

**Figures 101–108.**
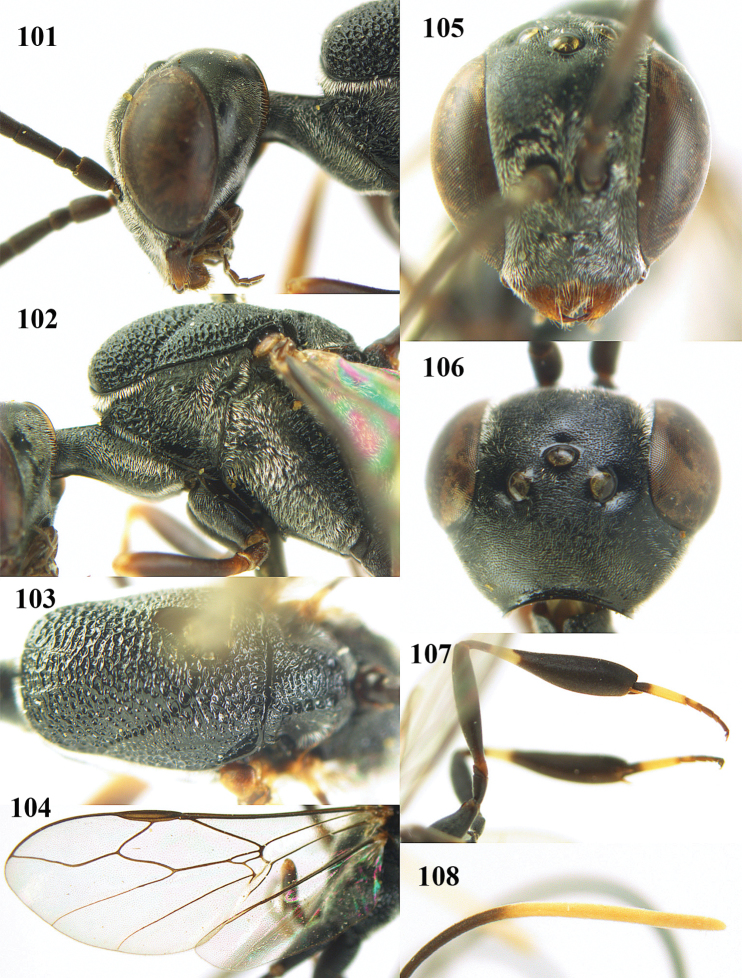
*Gasteruption diversipes* (Abeille de Perrin), female, France. **101** head lateral **102** mesosoma lateral **103** mesonotum dorsal **104** fore wing **105** head anterior **106** head dorsal **107** hind leg **108** apex of ovipositor sheath.

**Figures 109–114.**
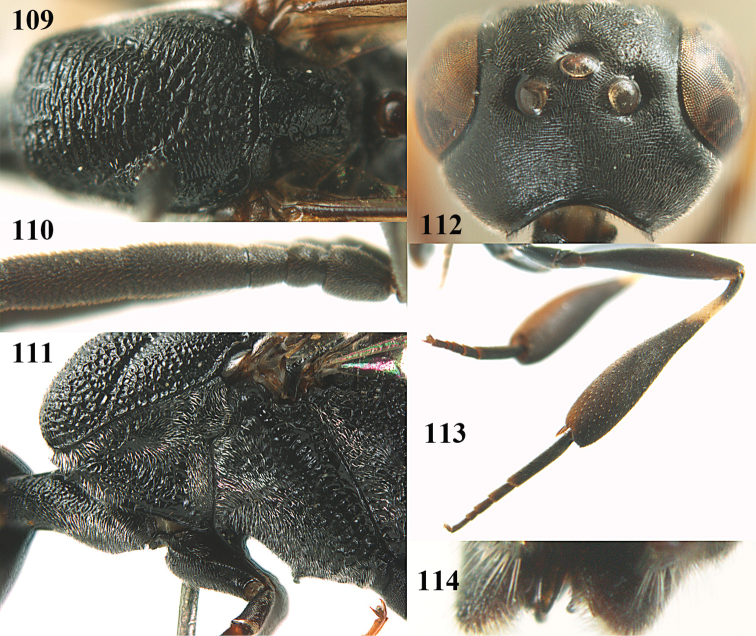
*Gasteruption diversipes* (Abeille de Perrin), male, Italy, but 111, 113 and 114 from France. **109** mesonotum dorsal **110** basal antennal segments **111** mesosoma lateral **112** head dorsal **113** hind leg **114** genitalia.

Apex of ovipositor sheath with a distinct white or ivory band, 1.4–2.5 times as long as hind basitarsus; head flat in front of occipital carina, without any depression; antesternal carina lamelliform and distinctly curved up, rather wide lamelliform (Fig. 102), but wider in male (Fig. 111); propleuron normal, 0.8–0.9 times distance between tegulae and anterior border of mesoscutum or less (Fig. 102); head below eyes in frontal view short, narrowed (Fig. 105); temple in lateral view rather wide (Fig. 101); third antennal segment of female 1.9–2.1 times as long as second segment; occipital carina at most moderately lamelliform and much less than diameter of posterior ocellus (Fig. 102); frons flattened and finely transversely rugulose; vertex finely aciculate; pronotal teeth small; mesoscutum coarsely reticulate-rugose or rugose-reticulate (Figs 103, 109), its lateral lobes without separate punctures; hind coxa finely and densely striate or aciculate dorsally; hind tibia rather slender (Fig. 107); hind basitarsus largely pale (Fig. 107); ovipositor sheath 1.0–1.6 times as long as metasoma and 3.3–4.7 times as long as hind tibia. Male has third antennal segment rather short, hardly longer than second segment (Fig. 110) and fourth antennal segment about 1.2–1.4 times as long as second and third segments combined.

#### Distribution.

Europe, Iran, Turkey.

#### Biology.

Unknown. Collected in July-September.

#### Notes.

*Gasteruption diversipes* was reported by Yildirim et al. (2004) from Turkey and by Samin and Bagriacik (2012) from NW. Iran (West Azarbaijan, Ourmiech, 1426 m); but it may concern the similar and more common *Gasteruption schlettereri* Magretti.

### Gasteruption dolichoderum

Taxon classificationAnimaliaHymenopteraGasteruptiidae

Schletterer, 1889

Figs 115 –142

Gasteruption dolichoderum Schletterer, 1889: 383, 394, 404; Dalla Torre 1902: 1066; Szépligeti 1903: 369; Kieffer 1912: 270; Hedicke 1939: 9; Madl 1988b: 404, 1990a 128; Wall 1994: 153.
Gasteruption daisyi Alekseev, 1993: 152. **Syn. n.**

#### Type material.

Holotype male from Greece (Rhodes) lost (Madl 1988b). Holotype of *Gasteruption daisyi* not available; synonymy based on original description.

#### Material.

*Iran (Tehran, Shahriar, Karaj; Kerman, Jupar, 1900 m; Kerman, Deh Bakri, Gebal Barez Mts, 1640 m); *Turkey (Capadocia, Ürgüp; Cornelek, 40 km E of Mut; Yayladagi; 60 km E of Mut, Kirobasi; Mersin, Kuzucebelen; 30 km N of Erdemli Aslanci; 20 km E of Alanya; Manavgat; SE of Elazig, Hazar Gölü; 10 km N of Konya; 10 km E of Ercis, Van Gölü; 25 km E of Malatya, Kopeksiz; Osmaneli; Antakya, Harbie; Canakkale, 8 km N of Ezine, 35 m; Mansisa, 15 km SEE of Salihli, 170 m; id., 40 km NW of Salihli, 150 m; Acigöl, near Cardak; Eskisehir, Sakri ilica, near Gumele; Kahramanmaras, Pazarcik; Denizli, 10 km NE of Denizli, 290 m; Burdur, 5 km NE of Yesilova, 1060 m; Anatolia, E of Civril; 800 m; Uzuncaburc, 30 km N of Silifke; Anatalya, Demirtas, 100 m; Nevsehir, 5 km S of Avanos, Zelve, 1000 m; id., 10 km S of Avanos, Göreme, 1000 m; Anatalya, 5 km W of Manavgat, Side, 10 m; Mardin, Mardin, 1000 m; id., Midyat, 1000 m).

#### Diagnosis.

**Figures 115–124.**
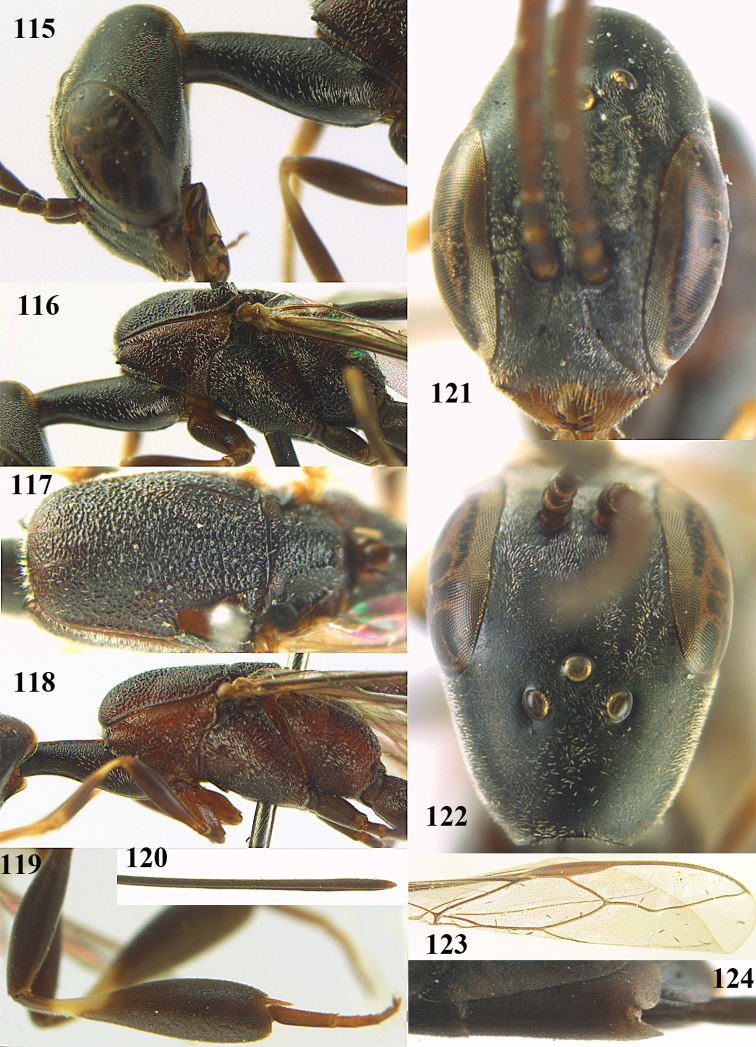
*Gasteruption dolichoderum* Schletterer, female, Greece. **115** head lateral **116, 118** mesosoma lateral **117** mesonotum dorsal **119** hind leg **120** apex of ovipositor sheath **121** head anterior **122** head dorsal **123** fore wing **124** hypopygium ventral.

**Figures 125–133.**
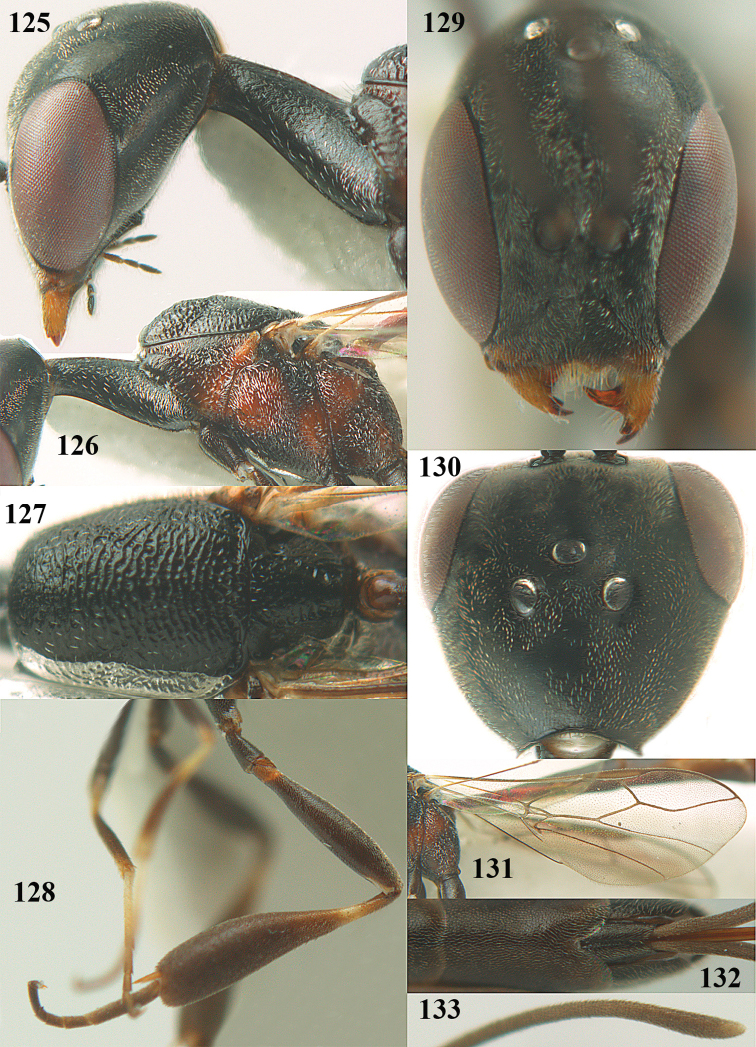
*Gasteruption dolichoderum* Schletterer, small female, Iran. **125** head lateral **126** mesosoma lateral **127** mesonotum dorsal **128** hind leg **129** head anterior **130** head dorsal **131** fore wing **132** hypopygium ventral **133** apex of ovipositor sheath.

**Figures 134–142.**
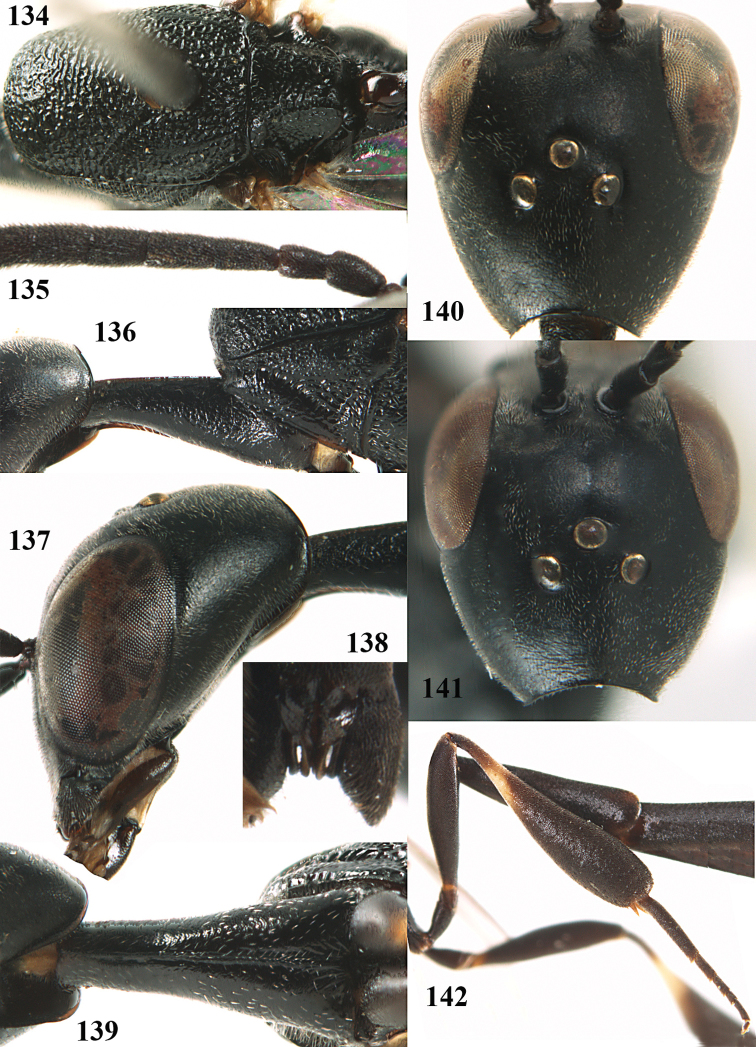
*Gasteruption dolichoderum* Schletterer, male, Greece, but 141 from Iran. **134** mesonotum dorsal **135** basal segments of antenna **136** propleuron lateral **137** head lateral **138** genitalia **139** propleuron ventral **140, 141** head dorsal **142** hind leg.

Apex of ovipositor sheath blackish or slightly brownish (Fig. 120), if rather pale apically then pale part distinctly shorter than hind basitarsus (Fig. 133); ovipositor sheath 2.3–2.5 times as long as hind tibia, 0.7–0.9 times as long as metasoma and at least 1.1 times as long as hind tibia and tarsus combined; head in anterior view “fez-shaped” (Fig. 121); occipital carina variable, obsolescent or lamelliform medio-dorsally (Figs 115, 125, 137); vertex smooth; temple strongly elongate (Figs 115, 125), about as long as eye in dorsal view, but sometimes 0.7–0.8 times; genal bridge about as long as third antennal segment; vertex smooth or nearly so, at most with very superficial punctulation and with satin sheen; eyes more or less setose; face narrow (Fig. 121); temples rather weakly narrowed behind eyes in dorsal view (Fig. 122); POL 2.0–2.6 times diameter of posterior ocellus; fourth antennal segment of female about as long as third segment, and fifth antennal segment as long as third segment or slightly shorter; propleuron 1.1–1.3 times distance from tegulae to anterior border of mesoscutum and elongate (Figs 116, 126, 136, 139); anterior half of mesoscutum densely coriaceous-rugulose to largely smooth; notauli obsolescent (Figs 117, 127; of male usually shallowly impressed: Fig. 134); hind tibia with ivory subbasal ring; incision of hypopygium rather shallow (Figs 124, 132); body predominantly black or dark brown, but may be largely reddish-brown, with face and basal half of hind leg of female more or less black. Male has third antennal segment 1.3–1.6 times as long as second segment and fourth and fifth segments 1.0–1.2 and 1.3 times as long as third segment, respectively (Fig. 135).

#### Distribution.

Southeast Europe, *Turkey, *Jordan, *Iran, Central Asia. New for the fauna of Iran. Jordan and Turkey.

#### Biology.

Unknown. Collected in May-September.

#### Notes.

One small female from Shahriar (22–28.ix.2010; fore wing 3.0 mm and body 7.0 mm; Figs 125–133) has the head in dorsal view slightly curved laterally, the first subdiscal cell of fore wing narrow triangular (rarely found also in other specimens), the wing membrane subhyaline, the pronotal side only coriaceous ventrally and the hind tibia slightly slenderer than other specimens from Iran. Body is often more or less reddish-brown; a female from Jordan (RMNH) and Kyrgyzstan (BZL) have the body (including head) nearly entirely reddish-brown. The African *Gasteruption ifan* Berland, 1950, is very similar (e.g. by the shape of the head and the shortened antennal segments), but *Gasteruption dolichoderum* has wider hind tibia (as in *Gasteruption assectator*; slenderer in *Gasteruption ifan*), mesoscutum sparsely setose (densely setose) and head more narrowed posteriorly in dorsal view (less narrowed).

### Gasteruption flavimarginatum

Taxon classificationAnimaliaHymenopteraGasteruptiidae

van Achterberg sp. n.

http://zoobank.org/5961B7CA-844A-4627-BB98-00242B9C67F9

Figs 143 –162

#### Type material.

Holotype, ♀ (BZL), “**Jordan** west.sept., Jarash env., 1.v.1996, M. Halada ing.”. Paratypes (22 ♀ + 9 ♂): 2 ♀ + 1 ♂ (BZL, RMNH), same label data; 1 ♂ (BZL), “Jordan W, Jordan Valley, Dayr Alla, 27.iv.[19]96, Marek Halada”; 1 ♂ (BZL), “Jordan NW, W of Jarash, NE Rajib, 14.iv.2009, Snizek”; 1 ♂ (BZL), “Jordan NW, Jarash 10 km W, 1.v.1996, Ma. Halada”; 1 ♂ (RMNH), id., but Jarash; 1 ♂ (BZL), “NW Jordan, Irbid reg., 350 m, Saham vill., 3.v.2003, I. Pljushtch”; 1 ♀ (BZL), “Jordan sept.west., N. Shuna env., 29–30.iv.1996, Mi. Halada ing.”; 1 ♀ (BZL) “**Turkey** east, 50 km S [of] Kars, Pasli, 1.vii.1997, Ma. Halada”; 1 ♀ (RMNH), “Turkey south, 40 km E [of] Mut, Cornelek, 18.vi.[19]97, Marek Halada”; 2 ♀ (BZL, RMNH), “Türkei mer.or., Halfeti env., 3–5.v.1994, Mi. Halada”; 1 ♀ (BZL) “Turkey E, 40 km NE [of] Muradiye, 2200 m, 5.vii.2000, M. Halada”; 4 ♀ + 2 ♂ (BZL, RMNH), “**Uzbekistan** or., Czirczik, 41,1N 69,1E, 28.v.[19]94, Ma. Halada”; 1 ♀ (BZL), “Uzbekistan, Ugam Mt. R., Kainarsai gorge, 1100 m, 41°42'N, 70°02'E, 21.vii.1999, Makogonova”; 5 ♀ + 1 ♂ (BZL, RMNH), “Uzbekistan or., Aktaš, 41,2N 69,4E, 70 km NO Tachkent, 27.v.[19]94, Ma. Halada”; 1 ♀ (BZL), “Tadjik [= **Tajikistan**], W. Pamir Mt., 30 km N of Rushan, 3500 m, viii.1999, Gurko”; 3 ♀ (BZL, RMNH), “**China** c, [Shanxi, Ruicheng], Monan, 111,7'-34,7', river Huang He, 26–28.v.1996, J. Halada”.

Excluded from type series: 1 ♀ (BZL), “**Mongolia** – SE, 70 km S Saynshand, 1100 m, 6.viii.2007, M. Halada”; 3 ♀ (BZL, RMNH), “MGL – Bayankhongor, 2 km S Bayankhongor, N46°12', E100°43', 1880 m, 10.vii.2004, J. Halada”; 1 ♂ (RMNH), “Mongolia – SE, Domogov reg., stepp, 28 km SE Chatan-Bulag, 3.viii.2007, M. Halada”; 1 ♂ (BZL), “Mong. Atayn Mts., Gichigniy Nuruu, 10 km SW Talshand, 12.vii.2005, J. Halada”; 2 ♂ (BZL), “Mongolia – C, 90 km NE Tsetserleg, N45°03' E102°25', 1400 m, 27.vii.2005, J. Halada”;

#### Diagnosis.

**Figures 143–151.**
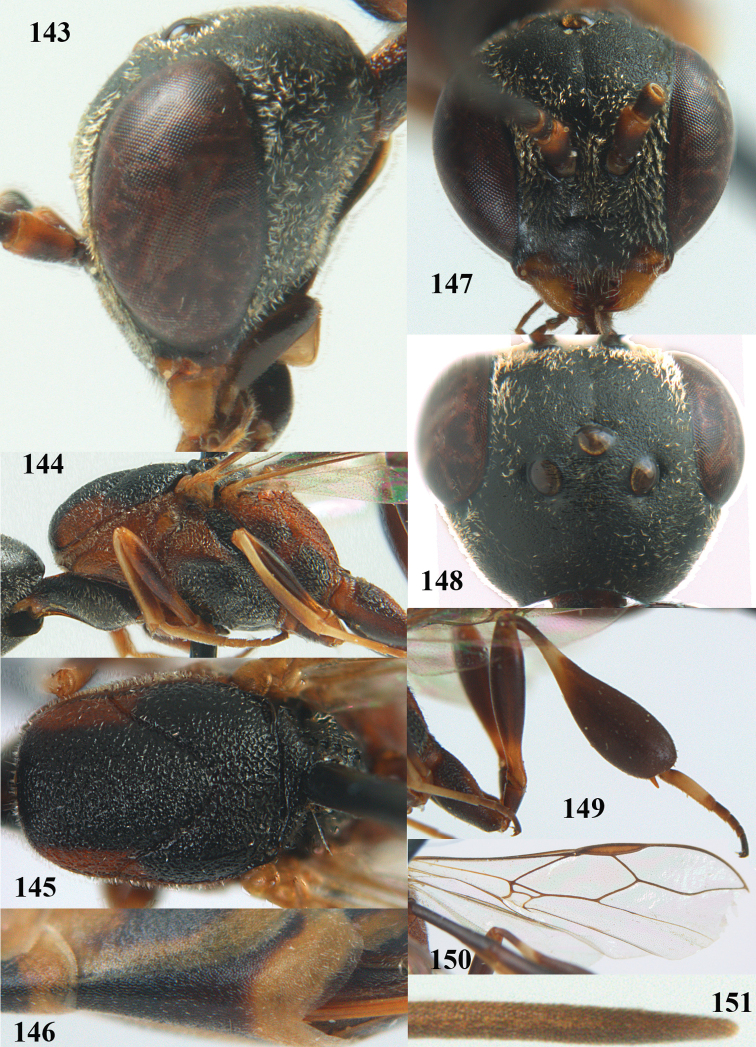
*Gasteruption flavimarginatum* sp. n., female, holotype. **143** head lateral **144** mesosoma lateral **145** mesonotum dorsal **146** hypopygium ventral **147** head anterior **148** head dorsal **149** hind leg **150** fore wing **151** apex of ovipositor sheath.

Head in front of occipital carina without depression (Fig. 148), in lateral view nearly flat dorsally and occipital carina narrow medio-dorsally and non-lamelliform (Fig. 143); vertex and frons matt, finely and densely coriaceous; antesternal carina narrow and non-lamelliform, antesternal carina and prepectal carina medio-ventrally similarly developed; head trapezoid and linearly narrowed behind eyes in dorsal view (Fig. 148); temple 0.7 times as long as eye in dorsal view; fourth and fifth antennal segments of ♀ 1.2 and 1.1 times as long as third segment, respectively; fourth segment of ♀ 0.8 times as long as second and third segments combined; apical antennal segment 2.4 times as long as penultimate segment; head not protruding below eyes and malar space 0.3 times length of second antennal segment and 0.2 times basal width of mandible and mandibular condylus close to lower level of eyes (Fig. 147); mandible yellow and with obsolescent or shallow basal depression; eye largely glabrous; propleuron stout, with satin sheen, 0.7 times as long as mesoscutum in front of tegulae and coriaceous (Fig. 144); antero-lateral teeth of pronotum absent; mesoscutum stout and inconspicuously setose (Fig. 145), anteriorly truncate, with satin sheen and largely finely and densely rugulose; hind femur medium-sized and slender (Fig. 149); hind coxa with satin sheen and coriaceous (but rugulose postero-dorsally); hind tibia inflated and with medium-sized basal petiolus; ovipositor sheath 0.3 times as long as metasoma, 0.6 times as long as hind tibia and tarsus combined and as long as hind tibia; apex of ovipositor sheath dark brown; length of body 8.5–11.5 mm. Male has third antennal segment 1.6 times as long as second segment, fourth segment 1.4 times third segment and 0.9 times as long as second and third segments combined (Fig. 155); paramere narrowly ivory apically (Fig. 154). The new species shares with West Palaearctic *Gasteruption paglianoi* and the mainly Oriental *Gasteruption brevicuspis* Kieffer, 1911, the yellow mandible, but the new species has apical 0.7 of the antenna dark brown (yellowish brown in *Gasteruption paglianoi*), the head narrowed behind the eyes (parallel-sided), basal 0.6 of the hypopygium dark brown (pale yellowish brown) and the hind femur slenderer (distinctly inflated). It differs from *Gasteruption brevicuspis* by having head shorter and less directly narrowed in dorsal view (longer and directly narrowed in *Gasteruption brevicuspis*), the metasoma with distinct yellowish pattern (largely absent) and the head nearly flat dorsally in lateral view (moderately convex).

#### Description.

Holotype, female, length of body 11.5 mm (of fore wing 5.0 mm).

*Head*. Vertex and frons matt, finely and densely coriaceous and in front of occipital carina without medial depression (Fig. 148), in lateral view nearly flat and occipital carina narrow medio-dorsally and non-lamelliform (Fig. 143); head trapezoid and linearly narrowed behind eyes in dorsal view (Fig. 148); temple 0.7 times as long as eye in dorsal view; fifth antennal segment 1.1 times as long as third segment; clypeus slightly impressed medio-ventrally; head not protruding below eyes and malar space 0.3 times as long as second antennal segment (Fig. 147); mandible with obsolescent basal depression.

*Mesosoma*. Length of mesosoma 1.7 times its height; pronotal side high, mainly finely punctate, with satin sheen and grooves narrow and rather shallow; antero-lateral teeth of pronotum absent; propleuron with satin sheen and coriaceous, 0.7 times as long as mesoscutum in front of tegulae and stout (Fig. 144); antesternal carina non-lamelliform, antesternal carina and prepectal carina medio-ventrally similarly developed (Fig. 144); mesoscutum stout and inconspicuously setose (Fig. 145), anteriorly truncate, with satin sheen and largely finely and densely rugulose; scutellum similarly but more superficially sculptured than mesoscutum.

*Legs*. Hind coxa with satin sheen and coriaceous (but rugulose postero-dorsally); hind femur rather slender; hind tibia inflated, with short pale bristles and with medium-sized basal petiolus (Figs 149, 162); length of hind femur, tibia and basitarsus 4.3, 3.5 and 3.8 times their width, respectively; hind basitarsus widened basally in dorsal view and as long as remainder of tarsus; pale hind tibial spurs similar to brown base of hind basitarsus.

*Metasoma*. Ovipositor sheath 0.3 times as long as metasoma, 0.6 times as long as hind tibia and tarsus combined and as long as hind tibia; hypopygium shallowly incised (Fig. 146).

*Colour.* Black or blackish-brown; mandible, clypeus laterally, scapus apically and dorsally, pedicellus apically, tegulae, second-sixth tergites apically and laterally, sternites apically and apical third of hypopygium yellow; fore tibia basally and anteriorly, middle tibia basally and apically, fore and middle basitarsus largely, subbasal band of hind tibia and hind basitarsus (except brown base and apex) ivory; mesoscutum antero-laterally, pronotum, mesopleuron dorsally, metapleuron dorsally, propodeum, remainder of fore leg (but base of coxa, femur medially and patch on tibia dark brown), middle and hind coxae dorsally, trochanters, base and apex of femora yellowish brown; palpi, pterostigma (but medially brown), remainder of hind leg and of metasoma and ovipositor sheath (including apex) dark brown, hind tibial spurs yellowish-brown; wing membrane subhyaline.

*Male.* Very similar to female; two basal antennal segments and mesosoma black and only sometimes pedicellus pale apically. Third antennal segment 1.6 times as long as second segment, fourth segment 1.4 times third segment and 0.9 times as long as second and third segments combined, fifth segment 0.9 times as long as fourth segment (Fig. 155); pronotal side partly rugulose ventrally; mesoscutum regularly transversely rugulose and shiny; colour and shape of hind leg as of female, but coxa black and basitarsus largely dark brown; apex of paramere ivory apically (Fig. 154).

*Variation*. Length of body of ♀ 7.0–11.5 mm (of ♂ 6.8–10.3 mm); pronotal side usually largely finely rugulose ventrally; length of ovipositor sheath 0.8–1.0 times as long as hind tibia; mesosoma and coxa entirely black or partly yellowish brown; apex of ovipositor sheath dark brown or brown; hind basitarsus of C. Asian specimens only basally ivory or pale brownish, of Chinese and Mongolian specimens entirely dark brown and slenderer than of holotype.

#### Notes.

The series from Mongolia is excluded from the type series because the head is somewhat protruding below the eyes and the malar space is 0.5 times length of the second antennal segment and 0.4 times basal width of the mandible and the mandibular condylus is below lower level of the eyes.

#### Distribution.

China, Jordan, ?Mongolia, Tajikistan, Turkey, Uzbekistan.

#### Biology.

Unknown. Collected in April-August.

#### Etymology.

Name derived from “flavus”, (Latin for “yellow”) and “marginis”, (Latin for “border”) because of the yellowish margins of the metasomal tergites.

**Figures 152–162.**
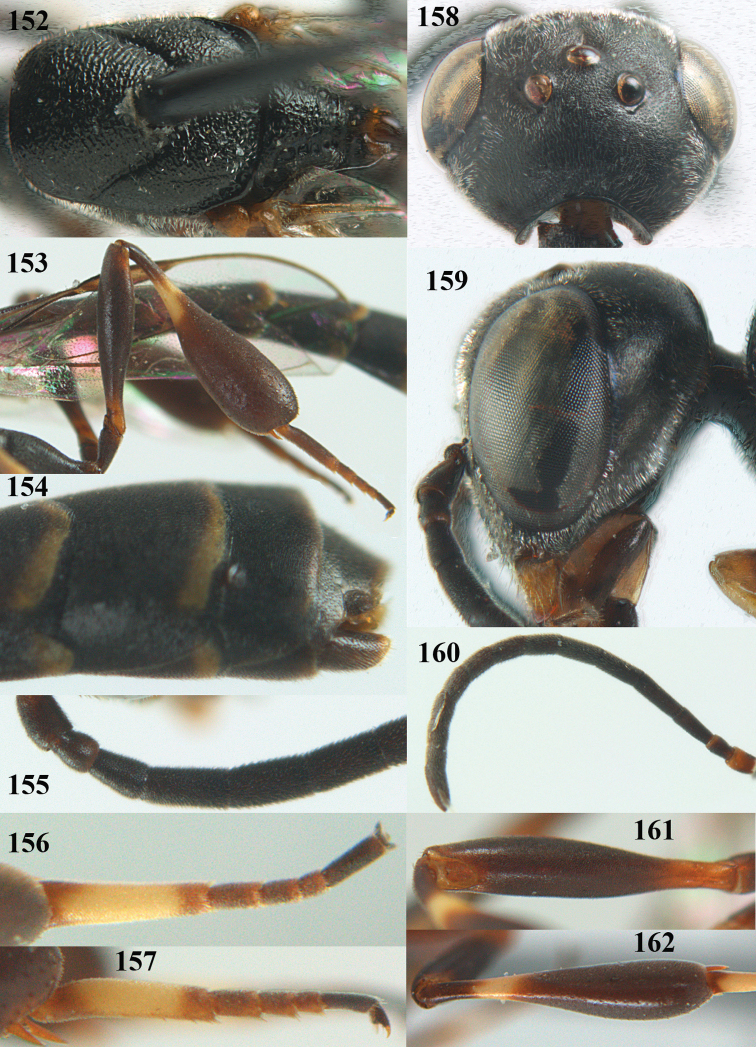
*Gasteruption flavimarginatum* sp. n., male, paratype, but **160** of female holotype and **156, 157, 161, 162** of female paratype from Turkey. **152** mesonotum dorsal **153** hind leg **154** apex of metasoma lateral **155** basal segments of antenna **156** hind tarsus dorsal **157** hind tarsus lateral **158** head dorsal **159** head lateral **160** antenna **161** hind femur latero-ventral **162** hind tibia dorsal.

### Gasteruption freyi

Taxon classificationAnimaliaHymenopteraGasteruptiidae

(Tournier, 1877)

Figs 163 –173

Foenus freyi Tournier, 1877: ix.
Faenus freyi ; Abeille de Perrin 1879: 264, 267, 276.
Gasteruption freyi ; Schletterer 1885: 278; Semenov 1892: 205; Dalla Torre 1902: 1067; Szépligeti 1903: 368; Kieffer 1912: 251; Schmiedeknecht 1930: 379; Hedicke 1939: 9 (as synonym of *Gasteruption erythrostomum* (Dahlbom)); Ferrière 1946: 236, 239, 242; Leclercq 1948: 76; Šedivý 1958: 36, 38; Györfi and Bajári 1962: 46, 50; Oehlke 1984: 170; Madl 1987b: 22, 1988c 38, 1989b 43, 1990b 480, 482; Kozlov 1988: 244, 247; Wall 1994: 155; Scaramozzino 1995: 3 (as *Gasteruption frey*); Pagliano and Scaramozzino 2000: 11, 17, 26; Saure 2001: 29; Yildirim et al. 2004: 1350; van der Smissen 2010: 372; van Achterberg 2013: 83.
Foenus nigripes Tournier 1877: ix. Synonymized with *Gasteruption freyi* (Tournier) by Šedivý, 1958.
Faenus nigripes ; Abeille de Perrin 1879: 264, 266, 276.
Gasteruption nigripes ; Schletterer 1885: 310, 1889: 407; Dalla Torre 1902: 1069; Kieffer 1912: 251; Schmiedeknecht 1930: 379, 381; Hedicke 1939: 17; Ferrière 1946: 242 (as form of *Gasteruption freyi* (Tournier)); Šedivý 1958: 39 (formalized synonymy with *Gasteruption freyi* (Tournier)); Wall 1994: 149.
Faenus rugulosus Abeille de Perrin, 1879: 264, 267, 275; Schmidt 1969: 294; Wall 1994: 149. Synonymized with *Gasteruption freyi* (Tournier) by Györfi and Bajári, 1962, Schmidt, 1969 and Oehlke, 1984.
Gasteruption rugulosum ; Schletterer 1885: 313, 1889: 384, 393, 395, 397, 401; Dalla Torre 1902: 1071; Szépligeti 1903: 369; Kieffer 1912: 258; Schmiedeknecht 1930: 380, 382; Hedicke 1939: 20; Oehlke 1984: 170; Györfi and Bajári 1962: 46, 50.
Gasteryption rugulosum ; Semenov 1892: 201.
Faenus nigripes var. annulata Abeille de Perrin 1879: 266, 276; Dalla Torre 1902: 1069; Hedicke 1939: 17.
Gasteruption nigripes var. annulatum ; Kieffer 1912: 251.
Gasteruption assectator var. nitidulum Schletterer 1885: 276; Hedicke 1939: 7. **Syn. n.**
Gasteruption kohlii Schletterer, 1885: 280; Madl 1988c: 38; Wall 1994: 148. Synonymized with *Gasteruption rugulosum* (Abeille de Perrin) by Schletterer, 1889 and with *Gasteruption freyi* (Tournier) by Madl, 1988c.
Gasteruption kohli ; Hedicke 1939: 20; Madl 1990b: 482, 2004 (FE website). Invalid emendation.

#### Type material.

Lectotype of *Gasteruption freyi* here designated, ♀ (MHNG) “[Switzerland], Sierre, [Wallis or Valais], Frey [= E. Frey-Gessner, 1826–1917]”, “Cn Tournier”, “Type”, *Foenus Freyi* Tourn., ♀”, “Lectotypus des. Madl”; 1 paralectotype, ♂ (MHNG), same labels but with male sign. Lectotype of *Gasteruption rugulosum* from S France (Marseille) here designated, ♀ (MNHN), “Museum Paris EY 0000003934”, “*rugulosus*”, “Museum Paris, coll. Abeille de Perrin, 1919”, “Monotypus, des. Madl, 1987”, “*Gasteruption freyi* Tourn., ♀, det. Madl, 1987”; according to original description additional type specimen (♀) from Marseille. Lectotype of *Gasteruption nigripes* here designated, ♀ (MHNG) “[Switzerland], P. [= Peney, near Genève], vii.[18]76”, “Cn Tournier”, “Type”, *Foenus nigripes* Tourn., ♀”, “Lectotypus, des. Madl, 1987”, “*Gasteruption freyi* Tourn., ♀, det. Madl, 1986”; paralectotypes: 4 ♀ (MHNG) from Peney, vii.1876 and 1 ♀ + 1 ♂ (MHNG) from Italy (“Turin, Gribodo”). Type specimens of *Gasteruption kohlii* not found in NMW; males from Italy (Südtirol, Bozen) and collected by F. Kohl. The specimen labelled “*Kohlii*, Typ., det. Schletterer”, (♂, NMW), and belonging to *Gasteruption freyi*, cannot be a type because it originates from Germany (“Thuringia, Gumperda, [O. Schmiedeknecht]”). Type series of *Gasteruption annulatum* from Marseille and Landes not found in MNHN. Holotype of *Gasteruption nitidulum* has not been identified with certainty, there is a male in NMW (“Wien, 10.vii.[18]83”) that may be the holotype and belongs to *Gasteruption freyi*. Considered to be a full synonym; the dark hind tarsus of males is not differentiating it from the typical *Gasteruption freyi*.

#### Material.

**Turkey** (Isparta, Egirdir Gölu, 5 km N of Akkecili, 920 m).

#### Diagnosis.

**Figures 163–170.**
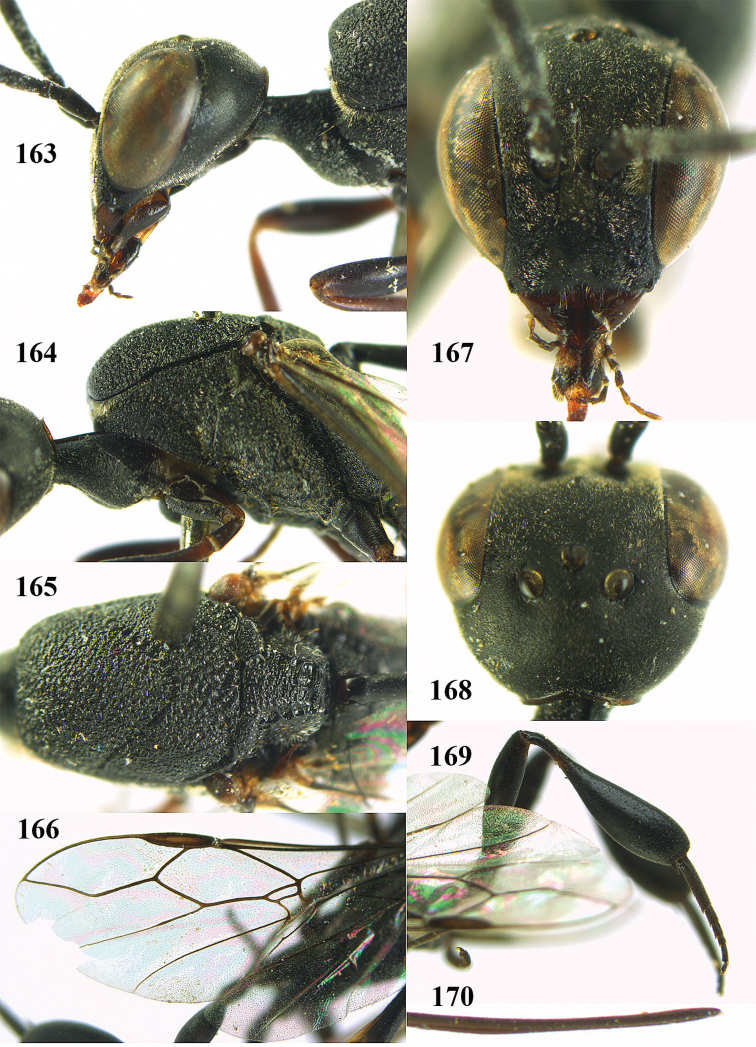
*Gasteruption freyi* (Tournier), female, France. **163** head lateral **164** mesosoma lateral **165** mesonotum dorsal **166** fore wing **167** head anterior **168** head dorsal **169** hind leg **170** apex of ovipositor sheath.

Apex of ovipositor sheath blackish or slightly brownish, if rather pale apically then pale part distinctly shorter than hind basitarsus; ovipositor sheath 1.2–1.4 times as long as hind tibia and 0.6–0.9 times as long as hind tibia and tarsus combined; occipital carina narrowly lamelliform medio-dorsally (Figs 163, 164, 168); head, mesosoma laterally and scapus black; clypeus with small depression or depression obsolescent; apical antennal segment at most 1.2 times as long as third antennal segment and its colour similar to colour of medial segments; antesternal carina narrow; hind tibia stout, subbasal pale ring or subbasal ventral patch of hind tibia usually absent or obsolescent (Fig. 169), but sometimes developed; hind basitarsus rather long (Fig. 169); hind tibial spurs blackish or dark brown; incision of hypopygium shallow. Male has third antennal segment usually rather long, significantly longer than second segment (Fig. 172) and apical antennal segment 1.0–1.2 times as long as third segment.

#### Distribution.

Europe, Turkey.

#### Biology.

Predator-inquiline of *Hylaeus* spp. Collected in June-September.

**Figures 171–173.**
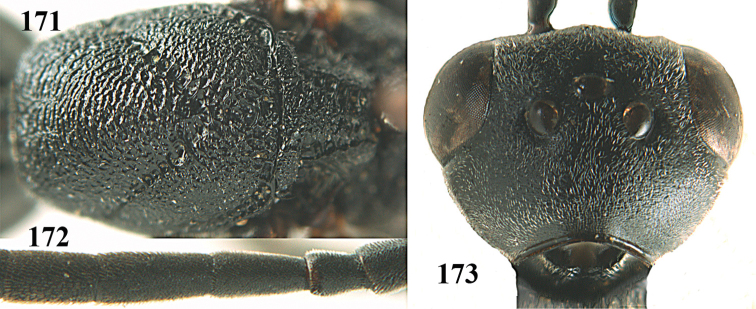
*Gasteruption freyi* (Tournier), male, Czech Republic. **171** mesonotum dorsal **172** basal antennal segments **173** head dorsal.

### Gasteruption goberti

Taxon classificationAnimaliaHymenopteraGasteruptiidae

(Tournier, 1877)

Figs 174 –186

Foenus goberti Tournier, 1877: vii; Schletterer 1889: 413 (as synonym of *Gasteruption pedemontanum* Tournier).
Faenus goberti ; Abeille de Perrin 1879: 263, 265, 267.
Gasteruption goberti ; Schletterer 1885: 319; Dalla Torre 1902: 1067; Szépligeti 1903: 368; Kieffer 1912: 249; Schmiedeknecht 1930: 376, 380; Hedicke 1939: 11; Ferrière 1946: 237, 238, 248; Šedivý 1958: 35, 36, 39; Györfi and Bajári 1962: 45, 49; Madl 1989b: 43; Wall 1994: 155; Scaramozzino 1995: 3; Pagliano and Scaramozzino 2000: 15, 17, 26.
Gasteruption sowae Schletterer, 1901: 219; Kieffer 1912: 273; Hedicke 1939: 21; Madl 1989b: 43; Wall 1994: 149. Synonymized with *Gasteruption goberti* (Tournier) by Madl, 1989b.

#### Type material.

Lectotype of *Gasteruption goberti* here designated, ♀ (MHNG), “[SW France, Landes], Mont de Marsan, Gobert [= E. Gobert, ?-1927]”, “Cn Tournier”, “Type”, *Foenus Goberti* Tourn., ♀”; female paralectotype not found, but male from type locality present in MHNG. Lectotype of *Gasteruption sowae* here designated, ♀ (NMW), “Pola [= Pula, Istria, Croatia], Schlett.”, “*sowae* Schlett., det. Schletterer”, “Typus”; 1 paralectotype, ♂ (NMW), with same labels as lectotype.

#### Additional material.

*Turkey (Eskisehir, Sakari Illica, near Gumele; near Halfeti; Burdur, 5 km NE of Yesilova, 1060 m; Istanbul; Hakkari, Esendere).

#### Diagnosis.

Apex of ovipositor sheath white or ivory and about 1.2 times as long as hind basitarsus; head with middle and lateral depressions in front of occipital carina deep and interconnected (Figs 179, 180, 185); occipital carina distinctly lamelliform and very wide, more or less concave medio-dorsally (Fig. 180); antesternal carina narrow and non-lamelliform or nearly so, not or slightly elevated above mesosternum (Fig. 175); fourth and fifth antennal segments of female 1.4–1.5 and 1.1–1.2 times as long as third segment, respectively; frons sparsely punctulate and with distinct interspaces or very finely and densely punctulate; vertex more or less finely punctulate and distinctly shiny; face narrow (Fig. 179); temples rather gradually roundly narrowed behind eyes (Fig. 180); propleuron 0.8 times as long as mesoscutum up to tegulae (Fig. 175); lateral lobes of mesoscutum rugose and partly punctate (Fig. 176); anterior half of mesoscutum largely coarsely reticulate-rugose (Fig. 182); outer side of hind tibia subbasally and apical half of hind basitarsus dark brown (Fig. 181); fore and middle legs mainly reddish-brown and without white or ivory markings; ovipositor about 1.2 times as long as body; body rather stout. Male has third antennal segment 1.5 times longer than second segment and fourth and fifth segments 2.0 and 1.5–1.7 times as long as third segment, respectively (Fig. 184).

#### Distribution.

France, Italy, Balkan, Turkey, Caucasus.

#### Biology.

Unknown. Collected in June-August.

**Figures 174–181.**
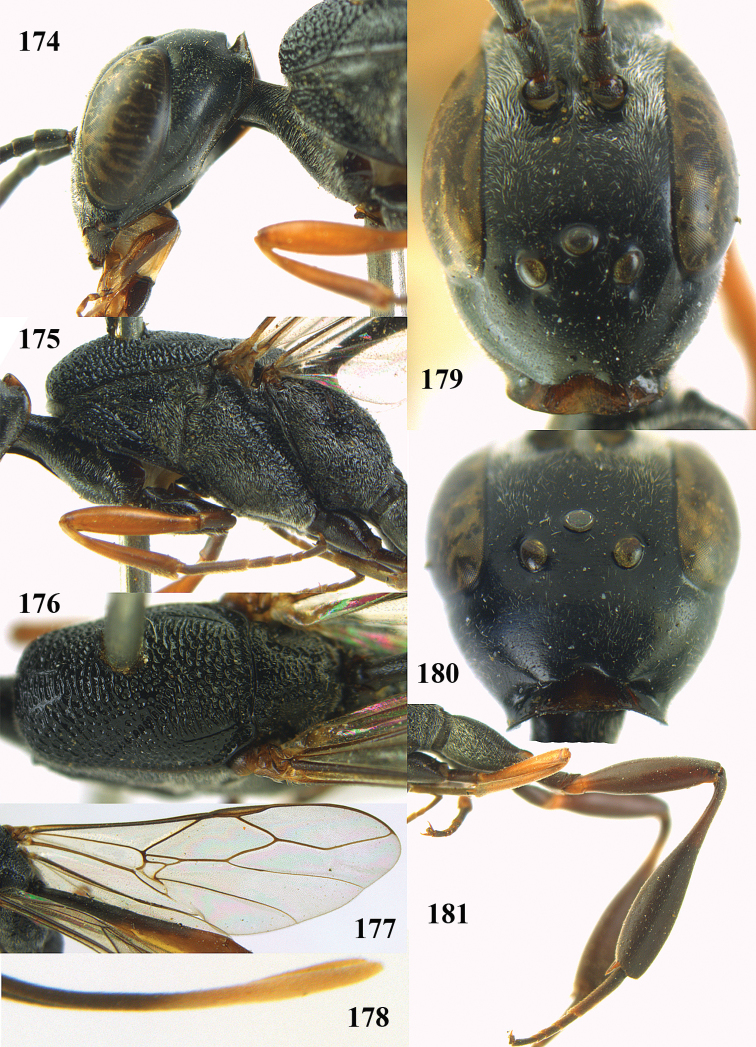
*Gasteruption goberti* (Tournier), female, lectotype. **174** head lateral **175** mesosoma lateral **176** mesonotum dorsal **177** fore wing **178** apex of ovipositor sheath **179** head antero-dorsal **180** head dorsal **181** hind leg.

**Figures 182–186.**
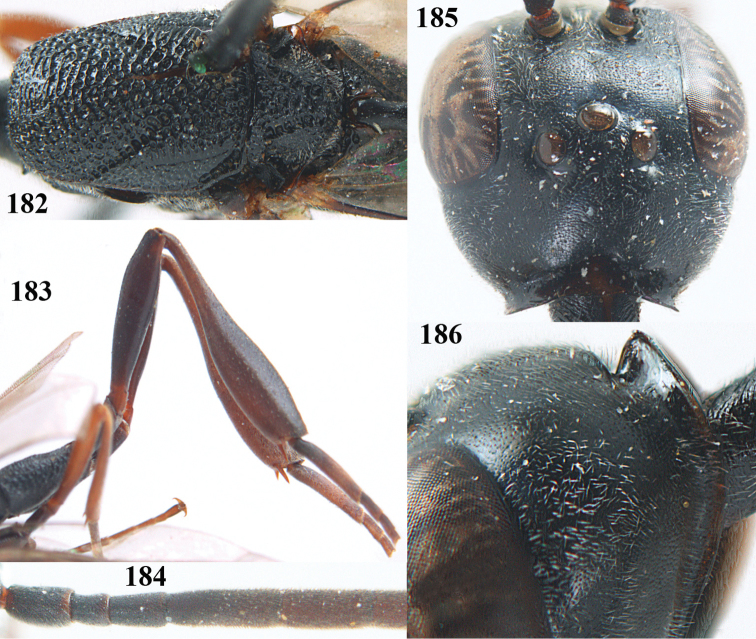
*Gasteruption goberti* (Tournier), male, paralectotype of *Gasteruption sowae*. **182** mesonotum dorsal **183** hind leg **184** basal antennal segments **185** head dorsal **186** head dorso-lateral.

### Gasteruption hastator

Taxon classificationAnimaliaHymenopteraGasteruptiidae

(Fabricius, 1804)

Figs 187 –204

Foenus hastator Fabricius, 1804: 142.
Gasteruption hastator ; Schletterer 1885: 307; Hedicke 1939: 13; Šedivý 1958: 35, 37, 40; Györfi and Bajári 1962: 47, 51; Schmidt 1969: 294; Oehlke 1984: 169, 170, 177; Madl 1987a: 402, 1987b: 22, 1988a: 13, 15, 1989b 43, 1990b 480, 482; Kozlov 1988: 244, 247; Kofler and Madl 1990: 321; Wall 1994: 156; Scaramozzino 1995: 3; Neumayer et al. 1999: 220; Pagliano and Scaramozzino 2000: 11, 17, 27; Saure 2001: 29; Turrisi 2004: 84; van der Smissen 2010: 372; van Achterberg 2013: 83.
Foenus dorsalis Westwood, 1841: 537, 1843: 258; Schletterer 1889: 400; Hedicke 1939: 13; Wall 1994: 148. Synonymized with *Gasteruption hastator* (Fabricius) by Oehlke, 1984.
Foenus esenbeckii Westwood, 1841: 537, 1843: 256; Oehlke 1984: 177 (as synonym of *Gasteruption hastator* (Fabricius)); Wall 1994: 148. Synonymized with *Gasteruption rubricans* (Guérin-Méneville) by Schletterer, 1889.
Faenus esenbeckii ; Abeille de Perrin 1879: 264, 265 (as *esenbecki*), 274.
Gasteruption esenbeckii ; Schletterer 1885: 305, 319, 1889: 400.
Gasteruption esenbecki ; Hedicke 1939: 13. Invalid emendation.
Foenus rubricans Guérin-Méneville, 1844: 407; Abeille de Perrin 1879: 274; Schmiedeknecht 1930: 379, 382; Hedicke 1939: 13; Ferrière 1946: 234, 238, 241; Leclercq 1948: 76; Wall 1994: 149. Synonymized with *Gasteruption esenbeckii* (Westwood) by Abeille de Perrin, 1879 and with *Gasteruption hastator* (Fabricius) by Schulz, 1912.
Gasteruption rubricans ; Schletterer 1885: 283, 1889: 384, 393, 395, 397, 400; Dalla Torre 1902: 1071; Szépligeti 1903: 370; Höppner 1904: 101; Kieffer 1912: 257; Ferrière 1946: 234, 241; Crosskey 1951: 294.
Gasteryption rubricans ; Semenov 1892: 200.
Gasteruption hastator *Gasteruption ? rubricans*; Hedwig 1957: 117 (SE. Iran: Sistan & Baluchestan, Damen)
Gasteruption rubricans ; Tirgari 1975: 57 (W. Iran: Khuzestan; N. Iran: Mazandaran, Chalus).
Gasteruption tibiale Schletterer, 1885: 286, 1889: 384, 393, 395, 397, 402; Dalla Torre 1902: 1075; Szépligeti 1903: 370; Kieffer 1904a: 647, 1912: 259; Schmiedeknecht 1930: 379, 382; Hedicke 1939: 25; Ferrière 1946: 234, 238, 241; Leclercq, 1948: 76; Hellén 1950: 4 (as synonym of *Gasteruption bidentulum* (Thomson)); Schmidt, 1969: 294 (id.); Oehlke 1984: 169, 171, 181; Madl 1987a: 402, 1988a: 12; Kozlov 1988: 246, 247; Wall 1994: 149; Yildirim et al. 2004: 1351 (Turkey). Synonymized with *Gasteruption hastator* (Fabricius) by Madl, 1987a.
Gasteryption tibiale ; Semenov 1892: 201.
Gasteruption schossmannae Madl, 1987: 37, 1990b 480; Wall 1994: 163. **Syn. n.**
Gasteruption graecum Schletterer, 1885: 279, 1889: 400; Kieffer 1912: 257 (as synonym of *Gasteruption rubricans*); Hedicke 1939: 14; Madl 1988b: 405, 1990a 128; Hedicke 1939: 13; Wall 1994: 148. Synonymized with *Gasteruption rubricans* (Guérin-Méneville) by Schletterer, 1889 and with *Gasteruption hastator* (Fabricius) by Oehlke (1984).

#### Type material.

The holotype of *Gasteruption hastator* (described from N. Africa) has been examined by Dr L. Vilhelmsen, Copenhagen; it has the typical widened hind basitarsus. The two female syntypes of *Gasteruption esenbeckii* Westwood, 1841 (described from Central Europe (Sickershausen, Germany) by Nees, 1834) are lost. Holotype of *Gasteruption tibiale* examined: ♂ (NMW), “[Italy], Tirol, St. Pauls [Bozen]”, “*tibiale* det. Schlett.”. Holotype of *Gasteruption graecum* is probably lost (a male in NMW from NW Greece, Epirus (Tinos) and collected by Erber). Holotype of *Gasteruption schossmannae* examined: ♂ (NMW), “[Austria], Winden am See, 16.vii.1962, leg. Priesner”, and holotype label by M. Madl.

#### Additional material.

**Iran** (Gilan, Astaneh, Eshman Kamachal; Ardabil, 1900 m; Kerman, Jupar, 1900 m; Boyer-A. o Kohg, Kuh Gol, near Sisakht, 2500 m; Azer. E Sh., Sis, 10 km E of Shabestar, 1540 m); **Turkey** (Bilecik, Baycicoy; Bolu, 17 km S of Seben; 10 km N of Tatvan; 20 km NW of Igdir; Mezikiran Gecidi, 20 km E of Gurun; Osmaneli; Cornelek, 40 km E of Mut; Sultan Daglari, near Yalvac; 10 km N of Muradya; Pasli, 50 km S of Kars; Eksiler, 20 km W of Silifke; 20 km SW of Bitlis; Canakkale, Gelibolu; 20 km W of Van; Konya, 10 km S of Aksehir Mts.; id., 30 km S of Aksehir; Capadocia, Urgup; near Izmir; 15 km E of Malatya; Tatvan, Van Gölü; Gevas, id.; 20 km SW of Burdur, 940 m; Karadut, 50 km NE of Agiyaman, 1000 m; Karadut, Nemrut Dagi; Bursa, near Caglian; 40 km NE of Muradiye, 2200 m; Muradiye; Hakkari, Akcali, 35 km S of Hakkari, 1700 m; Sile, Konya; near Agri; Sebran, Porsuk Baraji; Adapazar, Sakatya, 25 km S of Adapazar; Aciöl, near Cardak; SE of Elazig, Hazar Gölü; Burdur, 20 km SW of Burdur, 940 m; id., 28 km SEE of Burdur, 1350 m; id., 5 km NE of Yesilova, 1060 m; Kütahya, 28 km SSE of Kütahya, 1110 m; id., 20 km NEE of Kütahya; Denizli, 35 km SSE of Denizli, 970 m; Mansisa, 30 km SEE of Salihli, 430 m; id., 35 km SEE of Salihli, 900 m; Canakkale, 6 km N of Ezine, 35 m; Hakkari, Yüksekova, 1800 m; Hakkari, Mt. Sat, SW of Yüksekova, Varegös, 1650 m; Adiyaman, Gölbasi, 900 m; Konya, Beysehir, 1150 m; Nevsehir, Ürgüp, 1200 m; id., 20 km S of Nevsehir, Kaymakli, 1200 m; id., 20 km S of Nevsehir, Kaymakli; Bursa, Bursa, 300 m; SSE of Milas, Çamköy-Sek; Antalya, E Manevgat; id., Side-Titreyengöl; id., W Karabucak; 60 km W of Konya, Eflatun Pinar; Burdur, 20 km N of Aglasun, Koruglubeli, 950 m; Sivas, 10 km S of Gürün, near Gökpinar, 12500–1700 m).

#### Diagnosis.

**Figures 187–196.**
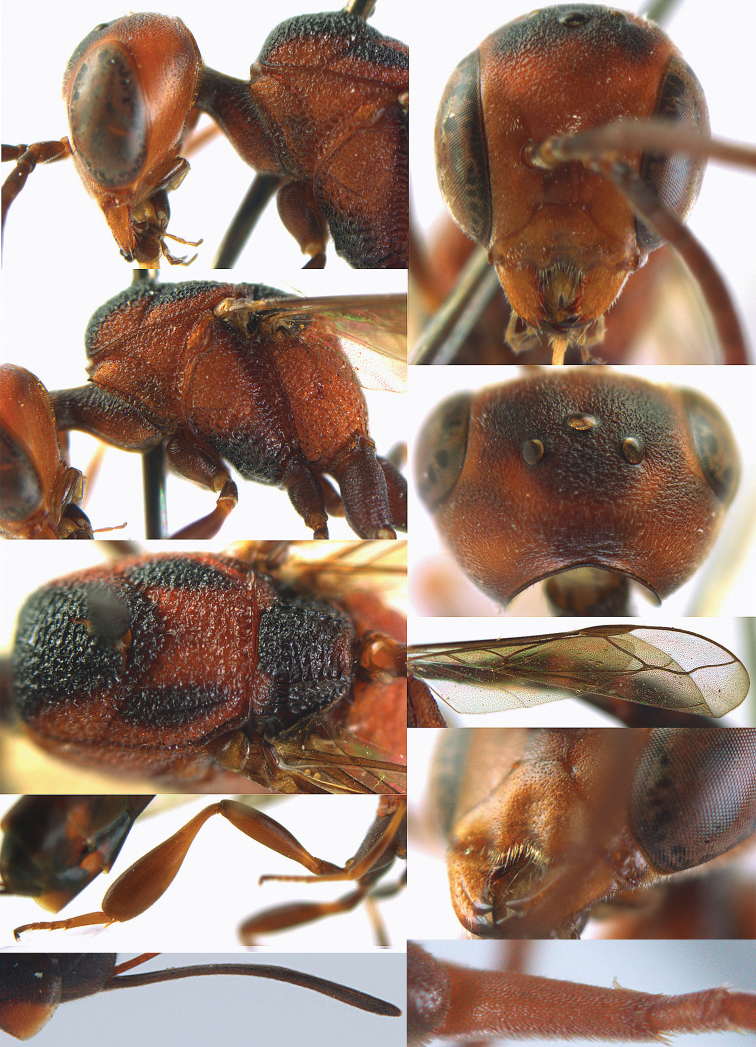
*Gasteruption hastator* (Fabricius), female, France. **187** head lateral **188** mesosoma lateral **189** mesonotum dorsal **190** hind leg **191** ovipositor sheath **192** head anterior **193** head dorsal **194** fore wing **195** clypeus antero-lateral **196** hind basitarsus dorsal.

Apex of ovipositor sheath blackish or dark brown; ovipositor sheath 0.6–1.5 times as long as hind tibia and 0.3–0.9 times as long as hind tibia and tarsus combined; head and laterally mesosoma of female mainly reddish-brown (Figs 187–189); scapus reddish-brown; head more (typical; Fig. 193) or less (“*graecum*”; Fig. 201) transverse in dorsal view; face rather wide (Figs 192, 202); shallow depression of clypeus rather large, more (typical; Fig. 195) or less (“*graecum*”; Fig. 203) extended; fifth antennal segment 0.9–1.0 times (♀) or 1.4–2.0 (♂) times as long as third segment (Fig. 199); apical antennal segment 1.4–1.5 times third antennal segment and darker than medial segments; occipital carina obsolescent to narrowly lamelliform medio-dorsally (Figs 187, 193, 204); antesternal carina narrow; hind tibia stout; hind basitarsus rather short (Figs 190, 200), in dorsal view more (typical; Fig. 196) or less (“*graecum*”) widened basad; mesoscutum anteriorly largely coarsely rugose and remainder rugose (to variable extend) and between them more or less coriaceous; incision of hypopygium shallow.

#### Distribution.

Europe, N. Africa, Iran, Turkey, Russia (including Far East).

#### Biology.

Predator-inquiline of *Osmia* and *Hylaeus* spp. in *Rubus* stems and of *Systropha* nests. Collected in May-August.

#### Notes.

One specimen from Iran is exceptionally large (length of body 13.8 mm and fore wing 5.5 mm) but agrees in other aspects with typical specimens. First species of *Gasteruption* reported from Turkey (by Semenov (1892) from “Tauria”).

**Figures 197–204.**
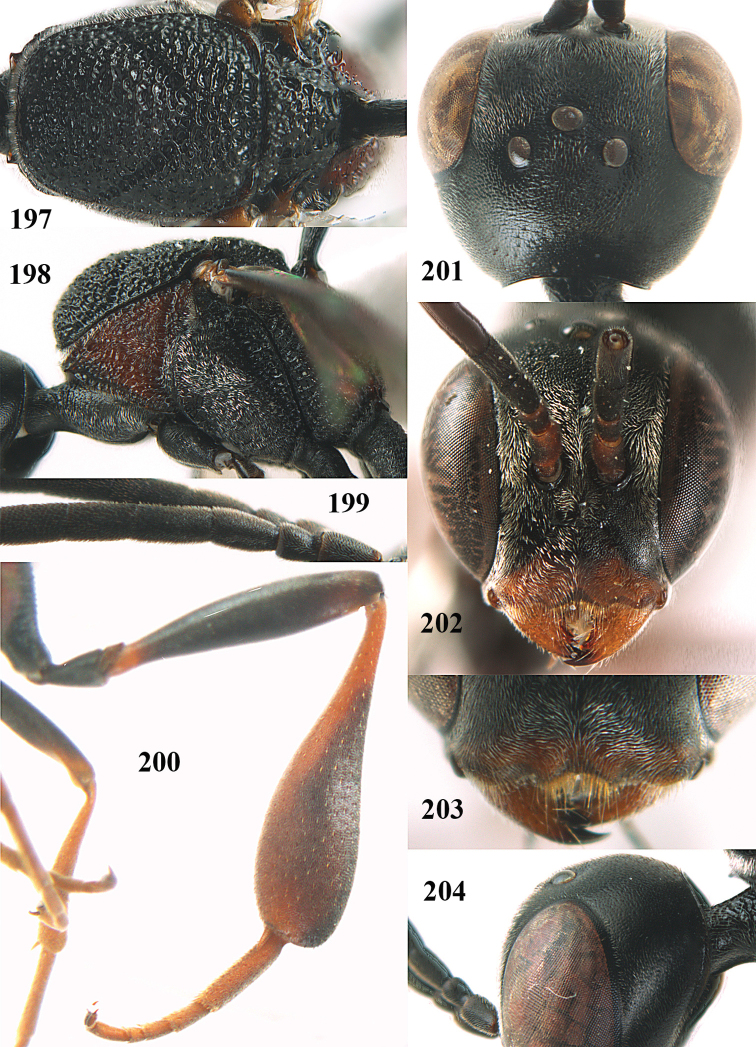
*Gasteruption hastator* (Fabricius), male, Bulgaria. **197** mesonotum dorsal **198** mesosoma lateral **199** basal antennal segments **200** hind leg **201** head dorsal **202** head anterior **203** clypeus anterior **204** head lateral.

### Gasteruption heminitidum

Taxon classificationAnimaliaHymenopteraGasteruptiidae

van Achterberg sp. n.

http://zoobank.org/BA2C3876-D8EC-4735-836F-29A35A3317B7

Figs 205 –219

#### Type material.

Holotype, ♀ (RMNH), “N. **Iran:** Tehran, Shahriar, MT [= Malaise trap] 25, 8–15.vi.2010, A. Nadimi, RMNH’12”. Paratypes (9 ♀ + 14 ♂): 1 ♂ (RMNH), with same label data as holotype; 1 ♀ + 1 ♂ (RMNH), id., but MT 24; 1 ♀ + 4 ♂ (RMNH, TMUT), id., but 1–8.vi.2010, G19 or G20; 1 ♀ + 1 ♂ (RMNH, TMUT), id., but 15–22.vi.2010, M. Khayrandish; 2 ♀ + 1 ♂ (RMNH, TMUT), id., but Karaj, 15–22.vi.2010, MT 27; 1 ♀ + 2 ♂ (RMNH), id., but 1–8.vi.2010, G9; 2 ♀ (RMNH, TMUT), id., but 22–28.vi.2010; 1 ♂ (RMNH), 8–15.vi.2010, MT 26; 1 ♂ (RMNH), “N. Iran: Alborz, Shahrestanak. Chalous Road, MT 29, 15–22.vi.2010, S. Farahani, RMNH’12”; 1 ♀ (RMNH), id., but 6–14.vii.2010; 1 ♂ (RMNH), “N. Iran: Qazvin, Zereshk Road, MT 5, 7–22.vi.2011, A. Nadimi, RMNH’12”; 1 ♂ (RMNH), id., but 22.vi.–6.vii.2011, MT 3, A. Mohammadi.

#### Diagnosis.

Head weakly convex dorsally, in front of occipital carina without medio-posterior depression; face moderately wide (Fig. 209); frons with satin sheen and densely finely punctulate; occipital carina narrowly lamelliform and dark brown; vertex rather shiny and moderately spaced finely punctulate; mandible yellowish brown basally, but partly darkened dorso-basally; propleuron 0.9 times as long as mesoscutum in front of tegulae; antesternal carina medium-sized lamelliform, directed posteriorly; mesoscutum shiny, coarsely punctate, and with smooth interspaces, medio-posteriorly reticulate-punctate (Fig. 207); scutellum shiny, partly smooth and with transverse rugae; mesosoma laterally (except pronotal side medially and ventrally) silvery pilose (Fig. 206); middle lobe protuberant (Fig. 207); hind basitarsus entirely dark brown, darker than yellowish brown hind tibial spurs (Fig. 208); hind tibia slender, outer side with punctures and short pale bristles and with large subbasal ivory patch (Fig. 208); ovipositor sheath 0.9–1.0 times as long as body, 1.4–1.6 times as long as metasoma, 2.8–3.1 times as long as hind tibia and tarsus combined and 4.0–5.1 times hind tibia; apex of ovipositor sheath dark brown or brown; length of body 8–11 mm; paramere dark brown or black apically (Fig. 216). Similar to *Gasteruption saharense* Benoit considering its sculpture, but the new species has the ovipositor sheath 1.4–1.6 times as long as metasoma (ovipositor sheath about as long as metasoma in *Gasteruption saharense*), pronotal side medially and dorsally sculptured (largely smooth), the occipital and antesternal carinae moderately lamelliform (non-lamelliform or nearly so), the scapus and mesosoma black (largely yellowish brown) and pterostigma dark brown (brown). Close to Central Asian *Gasteruption dimidiatum* Semenov, but the new species has the head trapezoid in dorsal view (subglobular in *Gasteruption dimidiatum*), the occipital carina wider (narrow), the head slightly emarginate medio-posteriorly (distinctly emarginate), the metasoma black (largely orange or yellowish brown), the hind tibia of male yellowish brown or brown ventrally (black) and third antennal segment of male dissimilar to second segment and 1.2–1.5 times as long as second segment (similar and 1.1–1.2 times longer).

#### Description.

Female, length of body 9.4 mm (of fore wing 4.4 mm).

**Figures 205–213.**
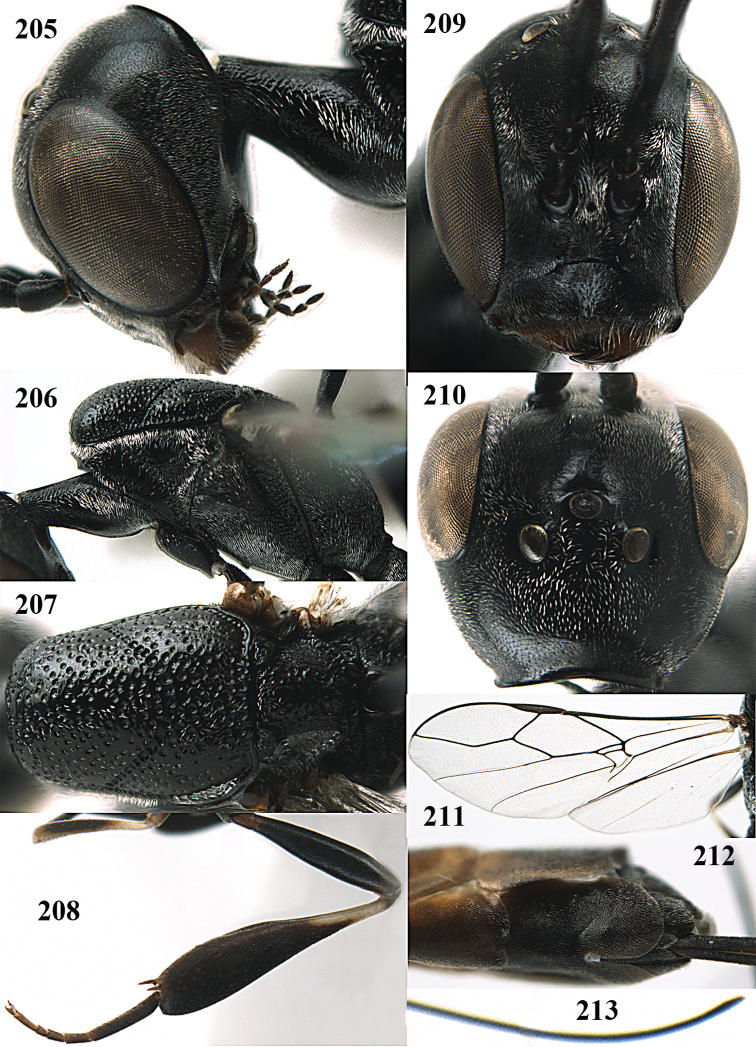
*Gasteruption heminitidum* sp. n., female, holotype. **205** head lateral **206** mesosoma lateral **207** mesonotum dorsal **208** hind leg **209** head anterior **210** head dorsal **211** fore wing **212** hypopygium ventral **213** apex of ovipositor sheath.

*Head*. Head weakly convex dorsally, posteriorly gradually narrowed, without medio-posterior depression; face and frons conspicuously silvery pilose; occipital carina narrowly lamelliform, dark brown (Fig. 205); third and fourth antennal segments 1.7 and 2.6 times as long as second segment, apical segment 1.7 times as long as penultimate segment; face moderately wide (Fig. 209); frons with satin sheen and densely finely punctulate; vertex rather shiny and moderately spaced finely punctulate; ventrally head not enlarged in anterior view, malar space 0.3 times length of pedicellus.

*Mesosoma*. Length of mesosoma 1.9 times its height; propleuron 0.9 times as long as mesoscutum in front of tegulae, silvery pilose and moderately stout posteriorly; laterally pronotum largely smooth and shiny ventrally, medially and ventrally without pilosity; side of pronotum with obsolescent tooth antero-ventrally; antesternal carina medium-sized lamelliform, directed posteriorly (Fig. 206); mesoscutum shiny, coarsely punctate, and with smooth interspaces, medio-posteriorly reticulate-punctate (Fig. 207), its middle lobe moderately protuberant and glabrous (Fig. 207); notauli rather shallow; scutellum shiny, partly smooth and with transverse rugae; mesopleuron and metapleuron silvery pilose (Fig. 206); eyes distinctly setose.

*Legs*. Length of hind femur, tibia and basitarsus 4.6, 4.5 and 5.6 times their width, respectively; hind tibia slender (Fig. 208); fore coxa close to mesopleuron; hind coxa shiny and rugulose dorsally; hind basitarsus moderately slender, as long as remainder of tarsus, distinctly widened in dorsal view.

*Metasoma*. Ovipositor sheath as long as body, 1.5 times as long as metasoma, 2.9 times as long as hind tibia and tarsus combined and 4.5 times hind tibia.

*Colour.* Black; metasoma dark brown, but basally and apically darker than medially; mandible (but dorsally basally darkened) and tegulae yellowish brown; fore and middle tibiae basally and basitarsi and hind tibia subbasally ivory; remainder of legs (except coxae) largely dark brown; palpi, pterostigma and hind basitarsus entirely dark brown; hind tibial spurs yellowish brown, paler than base of hind basitarsus; apex of ovipositor sheath dark brown; wing membrane subhyaline.

*Male.* Very similar to female. Third antennal segment 1.2–1.5 times as long as second segment, fourth segment 1.7–1.8 times third segment and as long as second and third segments combined, fifth segment 0.8–0.9 times as long as fourth segment (Fig. 218); mandible yellowish brown; scutellum often largely smooth and mesoscutum densely punctate; antesternal carina medium-sized; hind tibia dark brown and with subbasal ivory band; hind tibial spurs similarly coloured as outer side of basitarsus; hind tarsus dark brown; hind tibia yellowish brown or brown ventrally, dorsally mainly dark brown and its base entirely ivory or basally narrowly brown as in female; hind coxa transversely rugose dorsally; apex of paramere black or dark brown (Fig. 216).

*Variation.* Length of body of ♀ 8.3–11.3 mm (of ♂ 7.7–10.4 mm); mesoscutum often more densely punctate than in holotype; hind basitarsus entirely dark brown or apically ivory as two following segments; apical half of hypopygium dark brown or largely yellowish brown; ovipositor sheath 0.9–1.0 times as long as body, 1.4–1.6 times as long as metasoma, 2.8–3.1 times as long as hind tibia and tarsus combined and 4.0–5.1 times hind tibia.

#### Distribution.

Iran.

#### Biology.

Unknown. Collected in June-July.

#### Etymology.

Name derived from “hemi”, (Greek for “half”) and “nitidus”, (Latin for “shining”) because of the partly smooth and shiny mesoscutum.

**Figures 214–219.**
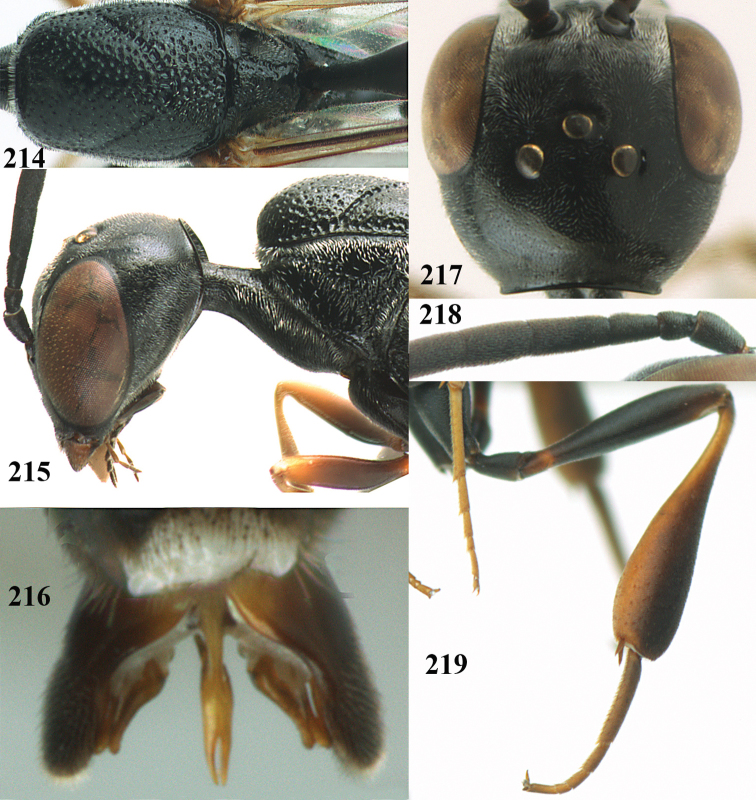
*Gasteruption heminitidum* sp. n., male, paratype. **214** mesonotum dorsal **215** head lateral **216** genitalia dorsal **217** head dorsal **218** basal antennal segments **219** hind leg.

### Gasteruption henseni

Taxon classificationAnimaliaHymenopteraGasteruptiidae

van Achterberg sp. n.

http://zoobank.org/3D793A2B-3B80-42FA-9267-2D38D30D1A8A

Figs 220 –234

#### Type material.

Holotype, ♀ (RMNH), “**Turkey**; Agri, 30 km W [of] Eleskirt, 2200 m, 14.vii.1987, R. Hensen”. Paratypes (6 ♀ + 2 ♂): 1 ♂ (RMNH), with same label data asa holotype; 1 ♀ (RMNH), “Turkey; Erzurum, Tortum, 1700 m, 16.vii.1987, R. Hensen”; 5 ♀ + 1 ♂ (BZL, RMNH), “Turkey east, Pasli, 50 km S [of] Sars, 1.vii.1997, Ma. Halada”;

#### Diagnosis.

**Figures 220–228.**
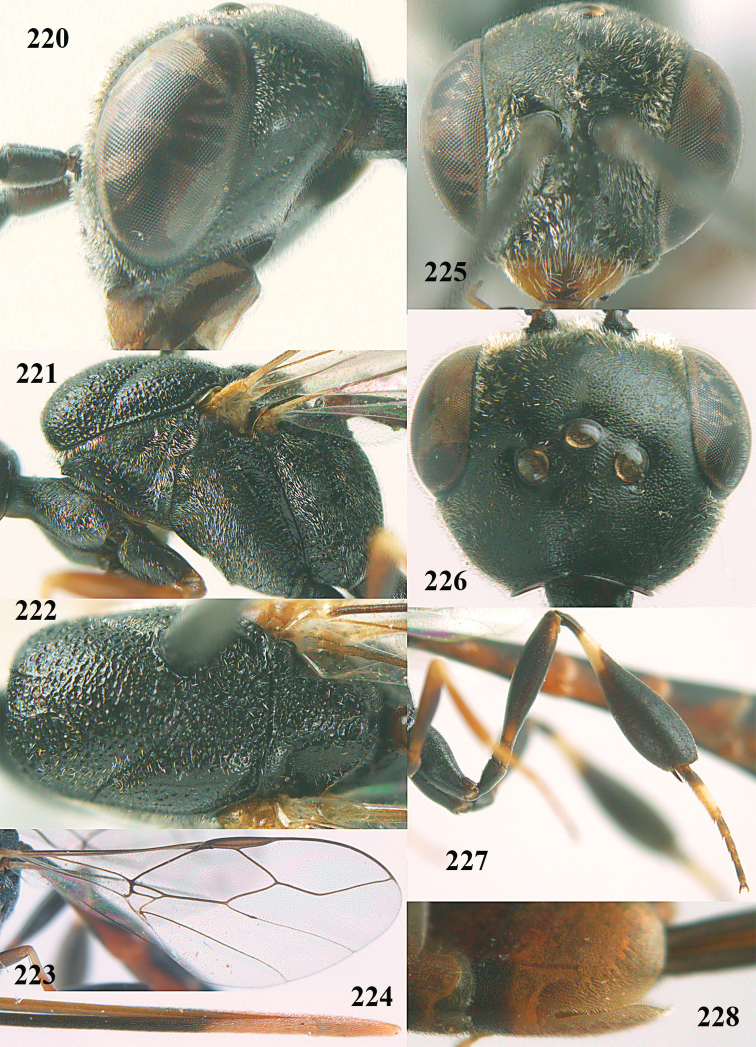
*Gasteruption henseni* sp. n., female, holotype. **220** head lateral **221** mesosoma lateral **222** mesonotum dorsal **223** fore wing **224** apex of ovipositor sheath **225** head anterior **226** head dorsal **227** hind leg **228** hypopygium ventral.

Head evenly convex dorsally, in front of occipital carina without medio-posterior depression; face wide (Fig. 225); frons and vertex with satin sheen and densely coriaceous-punctulate (Fig. 226); occipital carina narrowly lamelliform and dark brown (Fig. 220); mandible yellowish brown, but basally brown; propleuron 0.7 times as long as mesoscutum in front of tegulae and stout; antesternal carina narrow and non-lamelliform; middle lobe of mesoscutum with coarse punctures connected to rugulae, with satin sheen and interspaces largely smooth, lateral lobe similar but medially superficially coriaceous (Fig. 222); scutellum mainly transversely rugose; laterally mesosoma largely silvery pilose (Fig. 221); middle lobe slightly protuberant (Fig. 222); hind basitarsus dark brown basally, apically narrowly brown and remainder white or ivory; hind tibia distinctly swollen and with subbasal ivory ring (Fig. 227); hind basitarsus stout and 0.8 times as long as remainder of tarsus (without claws); ovipositor sheath 0.8 times as long as body, 1.3 times as long as metasoma, 2.1 times as long as hind tibia and tarsus combined and 3.4 times hind tibia; white or ivory apical part of ovipositor sheath 1.6–1.8 times as long as hind basitarsus; length of body 9–12 mm; hypopygium largely yellowish brown (Fig. 228); paramere broadly ivory apically (Fig. 231). Close to *Gasteruption schlettereri* Magretti, but the new species has the antesternal carina non-lamelliform (rather narrow lamelliform in *Gasteruption schlettereri*), the hind tibia distinctly swollen (slenderer), the hypopygium yellowish brown apically (dark brown) and the hind basitarsus tricoloured and shorter than remainder of tarsus without claws (uni- and bicoloured of males and females, respectively, and about as long as remainder of tarsus).

#### Description.

Female, length of body 9.8 mm (of fore wing 4.9 mm).

*Head*. Head evenly convex dorsally, without medio-posterior depression; face, frons laterally and temples distinctly pilose; occipital carina narrowly lamelliform, dark brown (Fig. 220); third and fourth antennal segments 1.5 and 2.2 times as long as second segment, apical segment twice as long as penultimate segment; face wide (Fig. 225); frons and vertex with satin sheen and densely coriaceous-punctulate (Fig. 226); ventrally head not enlarged in anterior view, malar space 0.3 times as, long as second antennal segment.

*Mesosoma*. Length of mesosoma 1.6 times its height; propleuron 0.7 times as long as mesoscutum in front of tegulae, stout; ventrally pronotal side coriaceous with some large punctures and only posteriorly with pilosity, with a small blunt tooth antero-ventrally; antesternal carina non-lamelliform and narrow; middle lobe of mesoscutum with coarse punctures connected to rugulae, with satin sheen and interspaces largely smooth, lateral lobe similar but medially superficially coriaceous (Fig. 222); scutellum mainly transversely rugose; middle lobe slightly protuberant (Fig. 222).

*Legs*. Length of hind femur, tibia and basitarsus 4.4, 3.8 and 4.3 times their width, respectively; hind tibia distinctly swollen and ventrally curved (Fig. 227); fore coxa close to mesopleuron; hind coxa moderately transversely rugose dorsally; hind basitarsus stout, 0.8 times as long as remainder of tarsus, widened basally in dorsal view.

*Metasoma*. Ovipositor sheath 0.8 times as long as body, 1.3 times as long as metasoma, 2.1 times as long as hind tibia and tarsus combined and 3.4 times hind tibia; white or ivory apical part of ovipositor sheath 1.7 times as long as hind basitarsus.

*Colour.* Black; mandible (but dorsally basally brown) yellowish-brown; trochantelli, base of hind femur, fore and middle tibia (except ivory base) and tarsi, tegulae, sternites apically and hypopygium (except dark brown base) yellowish brown; bases of fore and middle tibiae, subbasal ring of hind tibia and hind basitarsus (except dark brown basal third and narrowly brown apex) ivory; apex of ovipositor sheath ivory; palpi, pterostigma, remainder of legs and veins dark brown; metasoma laterally orange brown; wing membrane hyaline.

*Male.* Similarly stout as female, but frons and vertex coarser coriaceous-rugulose; pronotal side rugulose ventrally and mesoscutum more coarsely sculptured. Third antennal segment 1.2 times as long as second segment, fourth segment 1.8 times third segment and as long as second and third segments combined, fifth segment nearly as long as fourth segment (Fig. 234); hind tibia dark brown, but ventrally largely brown except for subbasal ivory band; hind tarsus brown, but basitarsus with ivory dorsal patch and laterally mainly pale brown; apex of paramere broadly pale yellowish or ivory (Fig. 231).

*Variation*. Length of body of both sexes 9.5–12.2 mm; length of ovipositor sheath 3.3–3.4 times as long as hind tibia and 1.1–1.3 times as long as metasoma and ivory apex 1.6–1.8 times as long as hind basitarsus; hind tibia of female 3.8–4.1 times as long as wide.

#### Distribution.

Turkey.

#### Biology.

Unknown. Collected in July.

#### Etymology.

Named after the collector of the holotype, the hymenopterist Raymond Hensen (Amsterdam).

**Figures 229–234.**
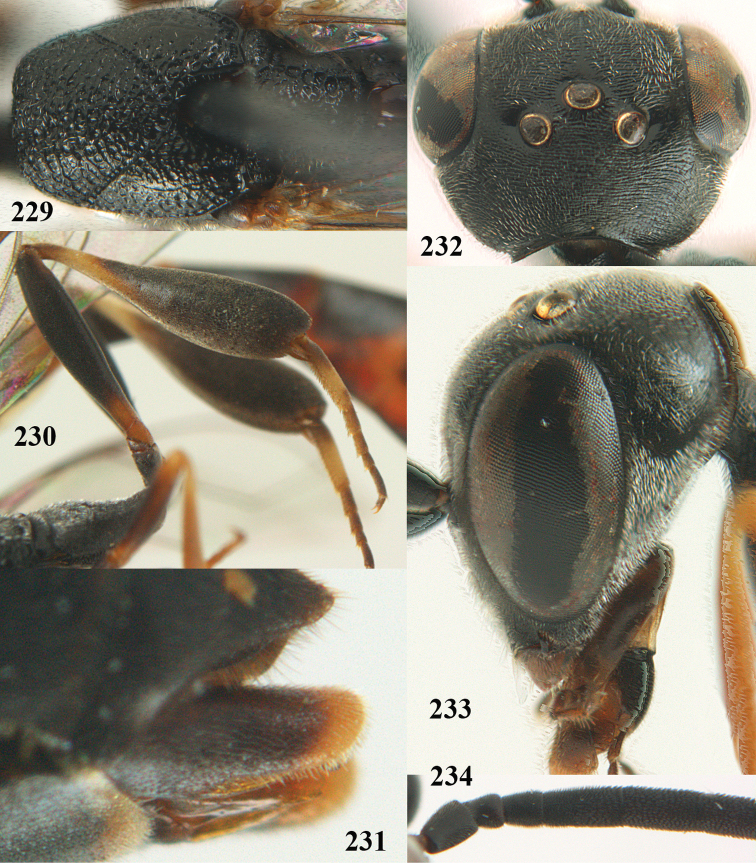
*Gasteruption henseni* sp. n., male, paratype. **229** mesonotum dorsal **230** hind leg **231** genitalia lateral **232** head dorsal **233** head lateral **234** basal antennal segments.

### Gasteruption insidiosum

Taxon classificationAnimaliaHymenopteraGasteruptiidae

Semenov, 1892

Figs 235 –249

Gasteryption insidiosum Semenov, 1892: 203–204.
Gasteruption insidiosum ; Dalla Torre 1902: 1068; Szépligeti 1903: 368; Kieffer 1912: 255; Hedicke 1939: 15.
Gasteryption fallaciosum Semenov, 1892: 202, 205; Dalla Torre 1902: 1067; Szépligeti 1903: 368, 371; Kieffer 1912: 252; Hedicke 1939: 10. **Syn. n.**
Gasteryption dubiosum Semenov, 1892: 203, 205; Dalla Torre 1902: 1066; Kieffer 1912: 252; Hedicke 1939: 10. **Syn. n.**
Gasteryption obsoletum Semenov, 1892: 203, 205; Dalla Torre 1902: 1069; Kieffer 1912: 252; Hedicke 1939: 10. **Syn. n.**
Gasteruption ? near *caudatum*; Hedwig 1957: 117 (SE Iran: Sistan & Baluchestan, Hamnat Kuh).
Gasteruption erythrostomum ; Yildirim et al. 2004: 1350 (Turkey); Samin and Bagriacik 2012: 387 (NW. Iran: East Azarbaijan (Arasbaran, 867 m)).

#### Type material.

Holotypes of *Gasteruption insidiosum*, ♀ (ZISP) from Russia (South of Volgograd, Kalmuck-steppe), of *Gasteruption fallaciosum* ♀ (ZISP) from Belorussia (Minsk), of *Gasteruption obsoletum* ♀ (ZISP) from Russia (Pensa) and of *Gasteruption dubiosum* ♀ (ZISP) from Russia (St. Petersburg) examined by the first author.

#### Additional material.

**Iran** (Alborz, Chalous Road, Shahrestanak; id., Sarziarat; Qazvin, Zereshk Road); **Turkey** (60 km W of Konya, Erflatun Pinar; 50 km S of Kars, Pasli; Konya, 30 km S of Aksehir, 20 km W of Van; 15 km E of Malatya; Zelve, Nevsehir; 25 km E of Malatya, Kopeksiz; near Karabulak; 30 km E of Mansisa; Burdur, 28 km SEE of Burdur, 1350 m; id., 20 km SW of Burdur, 940 m; id., 5 km NE of Yesilova, 1060 m; Isparta, Egirdir Gölu, 5 km N of Akkecili, 920 m; 54 km W of Kayseri, Göreme; Kayseri, Göreme, 1000 m; Maras, Goksün, 1400 m; Hakkari, Hakkari, 1750 m; Van, Van, 1800 m; Adiyaman, Gölbasi, 900 m; Hakkari, Mt. Sat, SW of Yüksekova, Varegös, 1650 m; Van, Baskale, 2200 m).

#### Diagnosis.

**Figures 235–243.**
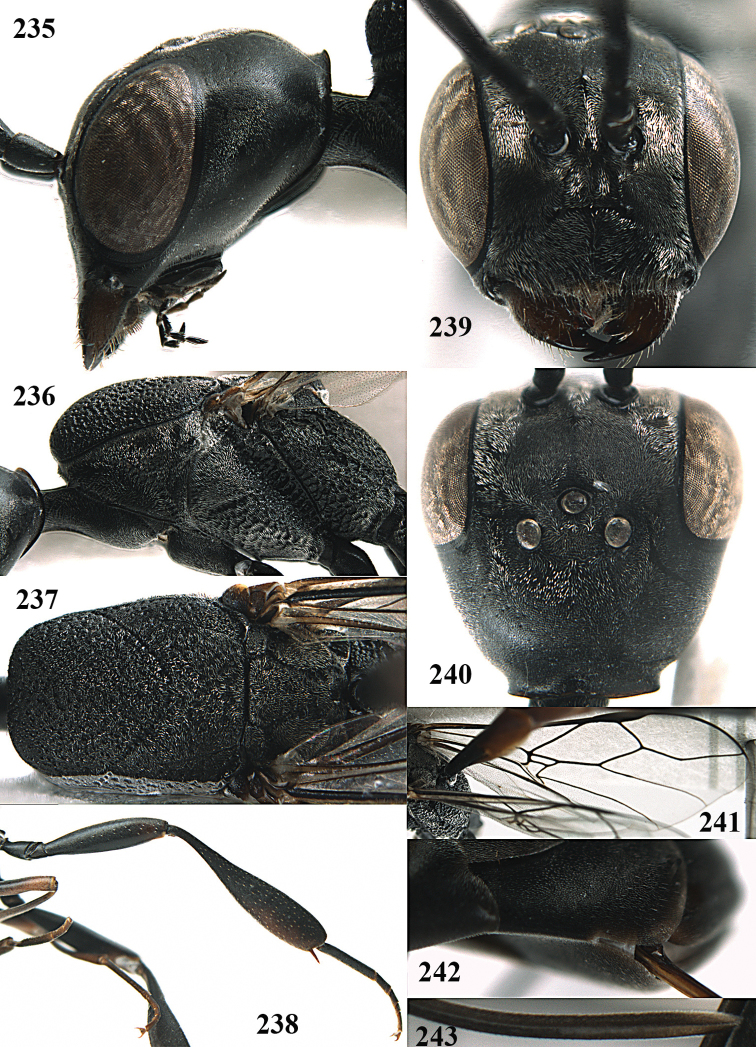
*Gasteruption insidiosum* Semenov, female, Iran. **235** head lateral **236** mesosoma lateral **237** mesonotum dorsal **238** hind leg **239** head anterior **240** head dorsal **241** fore wing **242** hypopygium lateral **243** apex of ovipositor sheath.

**Figures 244–249.**
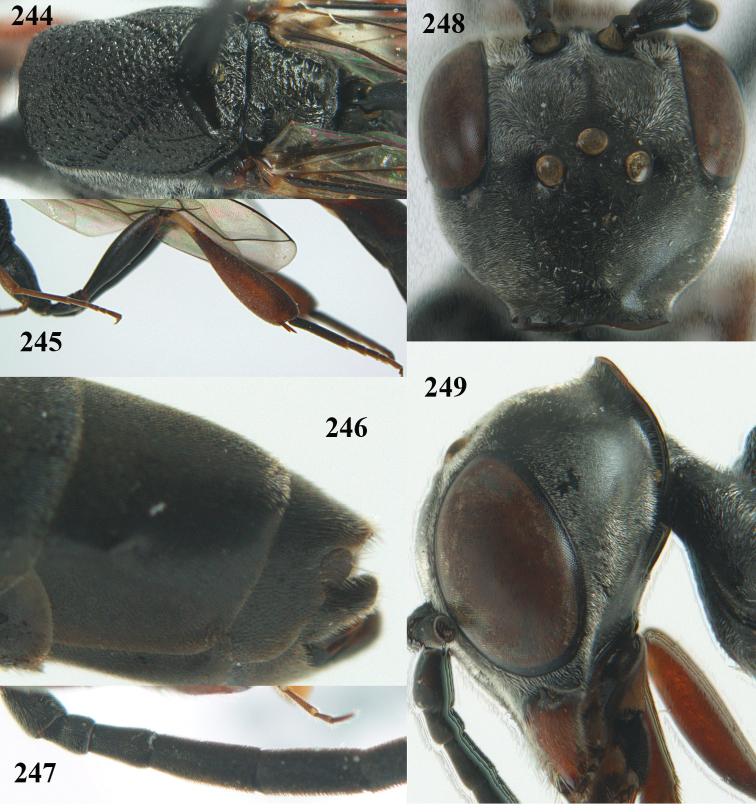
*Gasteruption insidiosum* Semenov, male, Turkey. **244** mesonotum dorsal **245** hind leg **246** apex of metasoma lateral **247** basal antennal segments **248** head dorsal **249** head lateral.

Length of ovipositor sheath 2.8–3.1 (rarely up to 4.3) times as long as hind tibia, 0.8–0.9 (rarely up to 1.2) times metasoma and 0.6–0.9 times as long as body; occipital carina widely collar-shaped and area in front of carina more or less aciculate (Figs 235, 249); mandible yellowish or orange brown basally; apical pale brown part of ovipositor sheath 0.1–0.3 times as long as hind basitarsus; mesoscutum more or less crater-like punctate because of deep medium-sized punctures (Figs 237, 244); lateral lobe of mesoscutum largely coarsely punctate; apical half of hind tibia more or less reddish brown and with pale yellowish setae; stout species. Male has third antennal segment 1.3–1.5 times longer than second segment and fourth and fifth segments 1.7–1.8 and 1.6–1.7 times as long as third segment, respectively (Fig. 247).

Close to *Gasteruption erythrostomum* (Dahlbom) but this species has the apical half of the hind tibia black or dark brown ventrally and with brown setae (more or less reddish brown and with pale yellowish setae (Fig. 238) in *Gasteruption insidiosum*), the ovipositor sheath shorter (1.7–2.6 times as long as hind tibia, 0.6–0.8 times metasoma, 1.1–1.6 times hind tibia and tarsus combined versus 2.5–3.1 times as long as hind tibia, 0.8–0.9 times metasoma and 1.7–1.9 times hind tibia and tarsus combined in *Gasteruption insidiosum*), the mesoscutum of female (especially lateral lobe) mainly coriaceous with at most small superficial punctures and rather shiny antero-dorsally (punctures deep, medium-sized and more or less finely crater-like and mixed with fine punctures between punctures, rarely only coarsely punctate) and matt antero-dorsally; mesoscutum of male transversely rugulose or moderately rugose, more coarsely punctate in *Gasteruption insidiosum*), the hind tibia black ventrally (usually partly reddish brown ventrally, rarely black), the occipital carina less collar-shaped and straight medio-dorsally (wide collar-shaped and more or less sinuate medio-dorsally) and the mesoscutum hardly setose (rather setose). Also similar to *Gasteruption nigrescens* Schletterer, 1885, but *Gasteruption insidiosum* has the ovipositor sheath 0.5–0.6 times as long as body and 2.6–2.9 times hind tibia (0.8–1.0 times body and 3.5–4.7 times hind tibia in *Gasteruption nigrescens*), the mesoscutum more crater-like punctate, the occipital carina wide (usually narrower in *Gasteruption nigrescens*) and the hind tibia wider and ventral half often partly dark brown (slenderer and black).

#### Distribution.

East Europe, Iran, Turkey.

#### Biology.

Unknown. Collected in June-July.

#### Notes.

The occurrence of *Gasteruption erythrostomum* in Iran and Turkey is questionable. The reported specimens most likely belong to the similar *Gasteruption insidiosum* Semenov.

### Gasteruption ischnolaimum

Taxon classificationAnimaliaHymenopteraGasteruptiidae

van Achterberg sp. n.

http://zoobank.org/04B7B9D6-80FF-4354-8694-2C589107C740

Figs 250 –258

#### Type material.

Holotype, ♀ (RMNH), “**Turkey**, Hakkari, S [of] Yüksecova, Varegös, 1650 m, 29.vi.1985, C.J. Zwakhals”. Paratypes (3 ♀): 2 ♀ (RMNH), “Museum Leiden, Turkey, prov. Hakkari, Sat Dag, Varegös, SW [of] Yüksecova, 1700 m, 4–8.viii.1983, W. Schacht”; 1 ♀ (RMNH), “N. **Iran:** Alborz, Shahrestanak, Chalous Road, MT 31, 15–22.vi.2010, S. Farahani, RMNH’12”.

#### Diagnosis.

**Figures 250–258.**
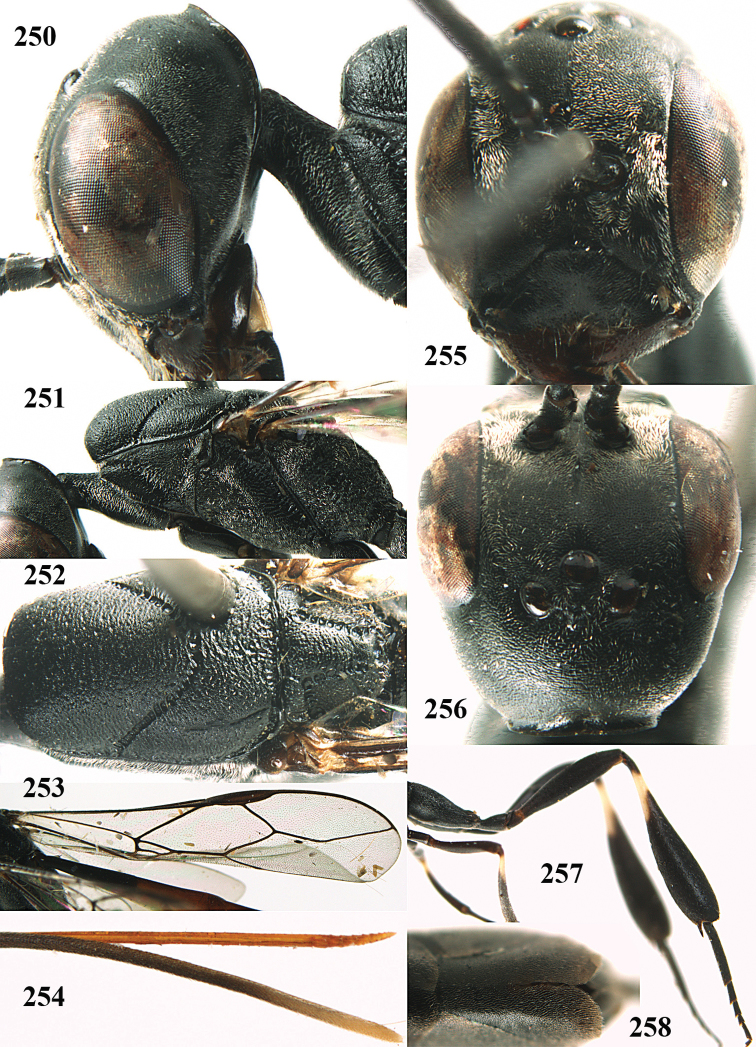
*Gasteruption ischnolaimum* sp. n., female, holotype. **250** head lateral **251** mesosoma lateral **252** mesonotum dorsal **253** fore wing **254** apex of ovipositor sheath **255** head anterior **256** head dorsal **257** hind leg **258** hypopygium ventral.

Head distinctly convex dorsally in lateral view, in front of occipital carina with obsolescent medio-posterior depression; face wide (Fig. 255); frons and vertex with satin sheen and densely punctulate, vertex anteriorly with some fine punctures between punctulation and posteriorly somewhat coriaceous; occipital carina medium-sized lamelliform, smooth and largely dark brown (Fig. 250); propleuron 0.9 times as long as mesoscutum in front of tegulae and laterally largely coriaceous and with satin sheen; pronotal side finely coriaceous except rather narrow crenulate groove; antesternal carina narrow and slightly lamelliform; middle lobe of mesoscutum finely coriaceous with medium-sized superficial punctures and with satin sheen, lateral lobe and scutellum coriaceous with fine superficial punctures (Fig. 252); mesosoma laterally white pilose except pronotal side medially and largely ventrally (Fig. 251); fore coxa close to mesopleuron; hind basitarsus entirely dark brown; hind tibia slender and with ivory subbasal patch (Fig. 257); fifth and sixth sternites of female dark brown; apical 0.5 of hypopygium of female incised; ovipositor sheath 0.8–0.9 times as long as body, 1.1–1.3 times as long as metasoma, 3.6–3.8 times as long as hind tibia and 2.2–2.4 times as long as hind tibia and tarsus combined; ivory apical part of ovipositor sheath 0.4–0.7 times as long as hind basitarsus (Fig. 254); length of body 11–12 mm.

#### Description.

Female, length of body 11.0 mm (of fore wing 5.8 mm).

*Head*. Head distinctly convex dorsally in lateral view, in front of occipital carina with obsolescent medio-posterior depression; face wide (Fig. 255); face and frons anteriorly silvery pilose; occipital carina medium-sized lamelliform and smooth (Fig. 250); third and fourth antennal segments 1.6 and 2.0 times as long as second segment, apical segment 1.2 times as long as penultimate segment; frons and vertex with satin sheen and densely punctulate, vertex anteriorly with some fine punctures between punctulation and posteriorly somewhat coriaceous; temples gradually narrowed behind eyes and resulting in trapezoid head in dorsal view (Fig. 256); ventrally head not enlarged in anterior view, malar space 0.2 times length of pedicellus; mandible dark reddish brown; tibial spurs nearly as dark as hind basitarsus; inner tooth of mandible minute.

*Mesosoma*. Length of mesosoma twice its height; propleuron 0.9 times as long as mesoscutum in front of tegulae, laterally coriaceous, stout and with satin sheen; pronotal side finely coriaceous except rather narrow crenulate groove; side of pronotum with minute tooth antero-ventrally; antesternal carina narrow and slightly lamelliform; middle lobe of mesoscutum finely coriaceous with medium-sized superficial punctures and with satin sheen, lateral lobe and scutellum coriaceous with fine superficial punctures; medio-posteriorly with some rugae (Fig. 256); notauli distinctly impressed; mesosoma laterally white pilose except pronotal side medially and largely ventrally (Fig. 251); propodeum with complete median carina.

*Legs*. Length of hind femur, tibia and basitarsus 4.6, 4.9 and 6.1 times their width, respectively; hind tibia and basitarsus slender (Fig. 257); fore coxa close to mesopleuron; hind coxa mainly coriaceous dorsally; hind tibia with some punctures and short greyish bristles; hind basitarsus somewhat widened basally in dorsal view; hind tibial spurs nearly as dark as basitarsus.

*Metasoma*. Ovipositor sheath 0.9 times as long as body, 1.3 times as long as metasoma, 3.8 times as long as hind tibia and 2.4 times as long as hind tibia and tarsus combined; ivory apical part of ovipositor sheath 0.4 times as long as hind basitarsus (Fig. 254).

*Colour.* Black; mandible, tegulae and hind tibial spurs dark reddish brown; bases of fore and middle tibiae and subbasal patch of hind tibia ivory; second and third segments orange brown; remainder of legs and metasoma dark brown or blackish brown; pterostigma and veins dark brown; wing membrane subhyaline.

*Male.* Unknown.

*Variation.* Length of body of ♀ 10.5–11.6 mm; pronotal side entirely coriaceous or with some rugulae; paratype from Iran has middle lobe of mesoscutum mainly finely transversely rugulose and mesoscutum medio-posteriorly extensively rugose; ovipositor sheath 0.8–0.9 times as long as body, 1.1–1.3 times as long as metasoma, 3.6–3;8 times as long as hind tibia and 2.2–2.4 times as long as hind tibia and tarsus combined; ivory apical part of ovipositor sheath 0.4–0.7 times as long as hind basitarsus.

#### Distribution.

Iran, Turkey.

#### Biology.

Unknown. Collected in June and August.

#### Etymology.

Named after the collector of the holotype and specialist of Ichneumonidae for his contribution to our knowledge of Ichneumonidae and for 50 years collecting of parasitoid Hymenoptera. “Ischnolaimum”, is from “ischnos”, (Greek for “weak”) and “laimos”, (Greek for “throat, neck”) and is a translation of the name “Zwakhals”.

### Gasteruption jaculator

Taxon classificationAnimaliaHymenopteraGasteruptiidae

(Linnaeus, 1758)

Figs 259 –271

Ichneumon jaculator Linnaeus, 1758: 565, 1761: 406, 1767: 937; Müller 1764: 71, 1775: 856; Fabricius 1775: 340, 1781: 435, 1787: 268; DeGeer 1776: 25; Villers 1789: 173; Gmelin 1790: 2696; Rossi 1790: 50; Christ 1791: 375; Petagna 1792: 365; Cederhjelm 1798: 163; Schrank 1802: 271; Hentschius 1804: 112; Latreille 1805: 194; Illiger 1807: 74; Roman 1932: 8; Fitton 1978: 378.
Foenus jaculator ; Fabricius 1798: 240, 1804: 141; Walckenaer 1802: 75; Panzer 1805: xcvi-16; Latreille 1805: 194, 1807: 258, 1810: 486; Lamarck 1817: 148, 1835: 359–360, 1839: 125; Dahlbom 1831: 76; Curtis 1832: 423; Nees 1834: 307; Oken 1835: 843; Stephens 1835: 170; Labram and Imhoff 1836: pl. 24; Zetterstedt 1840: 408; Westwood 1840: 134, 1843: 225; Blanchard 1840: 300; Taschenberg 1866: 93; Giraud 1877: 417; Thomson 1883: 846.
Gasteruption jaculator ; Kieffer 1912: 249; Roman 1932: 8; Crosskey 1951: 289; Hedqvist 1973: 183; Tirgari 1975: 57 (W. Iran: Khuzestan; C. Iran: Isfahan, Kashan); Fitton 1978: 378; Oehlke 1984: 170, 171, 178; Madl 1987a: 403, 1987b: 22, 1988a: 13, 15, 1988c 38, 1989a 160, 1989b 43, 1990a 128, 1990b 480, 482; Kozlov 1988: 246, 247; Flynn 1989: 117; Kofler and Madl 1990: 321; Narolsky and Shcherbal 1991: 23, 24; Wall 1994: 157; Scaramozzino 1995: 3; Neumayer et al. 1999: 220; Pagliano and Scaramozzino 2000: 13, 17, 28; Saure 2001: 29; Yildirim et al. 2004: 1350; Turrisi 2004: 84; van der Smissen 2010: 372; van der Spek 2012: 12; van Achterberg 2013: 83.
Foenus granulithorax Tournier, 1877: viii; Thomson 1883: 846; Wall 1994: 148. Synonymized with *Gasteruption jaculator* (Linnaeus) by Thomson, 1883 and Ferrière, 1946.
Faenus granulithorax ; Abeille de Perrin 1879: 264, 267, 273.
Gasteruption granulithorax ; Schletterer 1885: 279; Dalla Torre 1902: 1067; Szépligeti 1903: 369; Kieffer 1912: 266; Schmiedeknecht 1930: 377, 378, 381; Hedicke 1939: 12; Ferrière 1946: 237, 238, 245; Leclercq 1948: 76; Hellén 1950: 3; Šedivý 1958: 35, 36, 39; Györfi and Bajári 1962: 45, 50; Schmidt 1969: 294; Dolfuss 1982: 23.
Gasteryption granulithorax ; Semenov 1892: 213.
Faenus obliteratus Abeille de Perrin, 1879: 264, 266, 272; Hedqvist 1973: 183; Wall 1994: 148. Synonymized with *Gasteruption granulithorax* (Tournier) by Ferrière, 1946 and with *Gasteruption jaculator* (Linnaeus) by Hedqvist, 1973 and Oehlke, 1984.
Gasteruption obliteratum ; Schletterer, 1885, 310, 1889: 410; Dalla Torre 1902: 1069; Kieffer 1912: 255; Schmiedeknecht 1930: 377, 378, 381; Hedicke 1939: 17; Ferrière 1946: 245.
Foenus rugidorsus Costa, 1885: 22; Wall 1994: 149.
Gasteruption rugidorsum ; Kieffer 1912: 255.
Gasteruption rugidorsum ; Schletterer 1885: 325, 1889: 417, 419; Dalla Torre 1902: 1074; Hedicke 1939: 20; Ferrière 1946: 245. Synonymized with *Gasteruption thomsonii* Schletterer by Schletterer, 1889 (with a question mark) and Ferrière, 1946 (without comment).
Gasteruption thomsonii Schletterer, 1885: 285, Schletterer 1889: 382, 388, 394, 396, 417; Dalla Torre 1902: 1073; Kieffer 1912: 254; Höppner 1904: 101; Lindemans 1921: 298; Schmiedeknecht 1930: 377, 381; Hedicke 1939: 22; Ferrière 1946: 245; Hedqvist 1973: 183; Malyshev 1965: 248, 1968: 44; Wall 1994: 149. Synonymized with *Gasteruption granulithorax* Tournier by Ferrière, 1946 and with *Gasteruption jaculator* (Linnaeus) by Hedqvist, 1973 and Oehlke, 1984.
Gasteryption thomsoni ; Semenov 1892: 208.
Gasteryption thomsoni var. monochropus Semenov 1892: 208; Kieffer 1912: 255; Hedicke 1939: 24.
Gasteryption schewyrewi Semenov, 1892: 207–208; Dalla Torre 1902: 1072; Szépligeti 1903: 368; Kieffer 1912: 268; Hedicke 1939: 21. **Syn. n.**

#### Type material.

Lectotype of *Gasteruption jaculator* here designated, ♀ coll. no. 2651 [from Sweden, probably Uppsala] in the Linnean Society, London and examined by Roman (1932) and Fitton (1978). The lectotype has been studied digitally (www.linnean-online.org) by the first author; no. 2650 is a paralectotype, it has the antenna damaged and is labelled “48, *jaculator*”. Lectotype of *Gasteruption granulithorax* here designated, ♀ (MNHG), “[France], Bordeaux, Perez C.”, “Cn Tournier”, “Type”, “*granulithorax* Tourn., Soc. Br. C. rend., 1876”, “Lectotypus des. Madl, 1987”, “*Gasteruption jaculator* L., ♀, det. Madl, 1986”; 1 ♂ paralectotype (MNHG), “[France], Bord[eaux], Perez, a!”, “Cn Tournier”, “Type”, “*Foenus granulithorax* Tourn., ♂”, “Ce type ♂ n', Est pas la meme espèces que le type ♀ de *granulithorax*! C', Est probablement ♂ de *tibiale* Schl., Ch. Ferrière”, “Paralectotypus des. Madl, 1987”, “*Gasteruption bidentulum* Thoms., det. Madl, 1986”. Lectotype of *Gasteruption thomsonii* here designated: ♀ (ZIL) with small pink label, possibly indicating the type locality Gotland. Schletterer had material in NMW, but he listed in the original description only “Schweden”, as locality. Lectotype of *Gasteruption obliteratum* here designated, ♀ (MNHN), “Museum Paris EY 0000003930”, “*obliteratus* ab.”, “[Austria,] Dibg, 28.vi.[18]78”, “Museum Paris, coll. Abeille de Perrin, 1919”, “Lectotypus, des. Madl, 1987”, “*Gasteruption jaculator* L., ♀, det. Madl, 1987”; according to original description additional syntypes from France (Marseille, Bordeaux, les Landes, Pyrenees), but not examined. Holotype of *Gasteruption monochropus* ♀ (ZISP) from Russia examined. Holotype of *Gasteruption schewyrewi* ♂ (ZISP) from Ukraine (Poltawa) examined.

#### Additional material.

**Iran** (Mazandaran, Noor, Chamestan, Gaznasara; Alborz, Chalous Road, Shahrestanak); **Turkey** (Van, 20 km W of Van; Konya, 30 km S of Aksehir; 10 km W of Gaziantep; 15 km W of Refahye, W of Erzincan, 1600 m; near Tatvan; Anatolia, Paliklicesme, 50 m; Hakkari, Beytüsebab, 1400 m; id., Habur Deresi valley, S of Beylisebap, 1100 m; Van, Van, 1800 m; Hakkari, Mt. Sat, SW of Yüksekova, Varegös, 1650 m).

#### Diagnosis.

**Figures 259–266.**
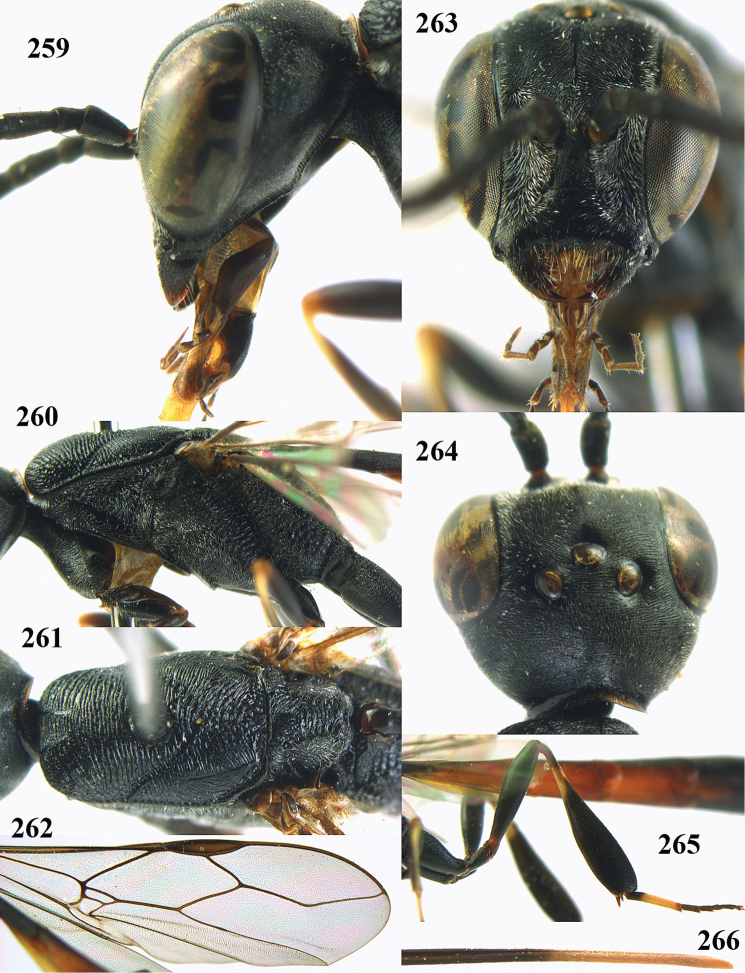
*Gasteruption jaculator* (Linnaeus), female, Netherlands. **259** head lateral **260** mesosoma lateral **261** mesonotum dorsal **262** fore wing **263** head anterior **264** head dorsal **265** hind leg **266** apex of ovipositor sheath.

**Figures 267–271.**
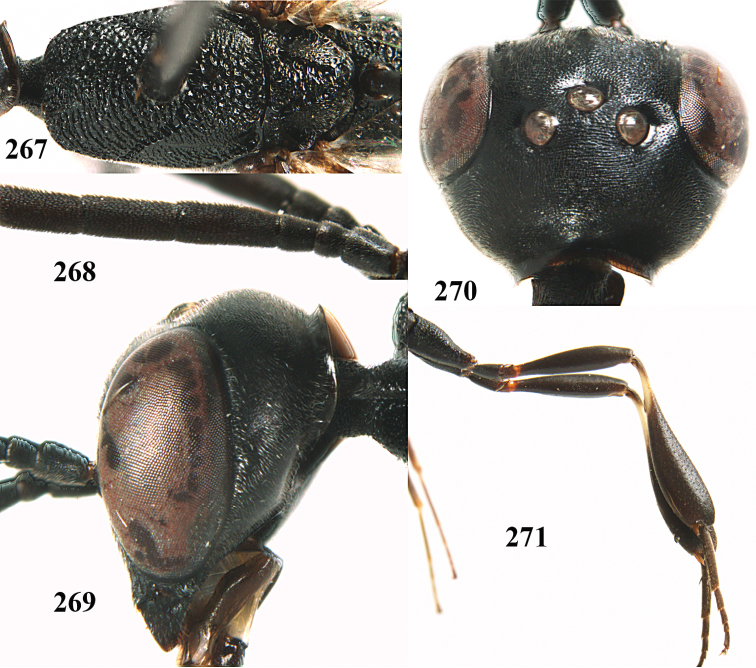
*Gasteruption jaculator* (Linnaeus), male, Norway. **267** mesonotum dorsal **268** basal antennal segments **269** head lateral **270** head dorsal **271** hind leg.

Apex of ovipositor sheath with a distinct white or ivory band, 1.5–2.7 times as long as hind basitarsus (up to 3.0 times in N. African specimens); head flat in front of occipital carina, without any depression; occipital carina strongly lamelliform and somewhat shorter than diameter of posterior ocellus (Figs 259, 269); fifth antennal segment of female 1.0–1.4 times as long as third segment; vertex rather matt and very finely aciculate; malar space short; antesternal carina narrow; length of propleuron 0.8–0.9 times distance between tegulae and anterior border of mesoscutum (Fig. 260); mesoscutum coarsely punctate-rugose anteriorly and interspaces more or less smooth, lateral lobes more or less coriaceous, contrasting with middle lobe (Figs 261, 267); hind tibia rather swollen (Fig. 265); hind tibia and basitarsus more or less ivory or white subbasally; ovipositor sheath 1.0–1.2 times as long as body and 1.5–1.7 times as long as metasoma (but only 1.4 times metasoma in N. African specimens). Males have shape of third antennal segment similar to second segment, rather short and usually 1.1–1.3 times as long as second segment and fourth antennal segment distinctly longer than (about 1.2 times as long as) second and third segments combined.

#### Distribution.

Europe, N. Africa, Iran, Turkey.

#### Biology.

Predator-inquiline of Colletinae (*Colletes* and *Hylaeus* spp.). Collected in May-August.

#### Notes.

The specimens from Mazandaran have the middle lobe of the mesoscutum more dominantly punctate than European and Turkish specimens and lack the transverse rugulosity, but a female from Gaznasara has superficial punctures on the mesoscutum between the transverse rugulosity and a second female has the mesoscutum mainly strongly punctate. Two males seen from slight impression near occipital carina medio-dorsally and considered to be an unknown species.

### Gasteruption laticeps

Taxon classificationAnimaliaHymenopteraGasteruptiidae

(Tournier, 1877)

Figs 272 –283

Foenus laticeps Tournier, 1877: viii.
Faenus laticeps ; Abeille de Perrin 1879: 278.
Gasteruption laticeps ; Schletterer 1885: 309, 1889: 414; Dalla Torre 1902: 1068; Kieffer 1912: 263; Hedicke 1939: 16; Ferrière 1946: 237, 238, 246; Šedivý 1958: 35, 36, 40; Györfi and Bajári 1962: 45, 48, 49; Madl 1987b: 23, 1988b 406, 1989a 160, 1989b 43, 1990a 128, 1990b 480; Kofler and Madl 1990: 321; Wall 1994: 158; Scaramozzino 1995: 3; Pagliano and Scaramozzino 2000: 15, 29; Saure 1999: 16, 2001: 29; van der Smissen 2010: 372; van Achterberg 2013: fig. 181.
Gasteruption foveolatum Schletterer, 1889: 381, 387, 394, 397, 410; Semenov 1892: 204; Dalla Torre 1902: 1067; Szépligeti 1903: 368; Kieffer 1912: 246; Schmiedeknecht 1930: 378, 380; Hedicke 1939: 11; Wall 1994: 148. Synonymized with *Gasteruption laticeps* (Tournier) by Ferrière, 1946.
Gasteruption foveolum Szépligeti, 1903: 368, 370; Kieffer 1904a: 640, 1912: 246; Schmiedeknecht 1930: 376; Hedicke 1939: 11; Györfi and Bajári 1962: 45; Malyshev 1965: 255; Tirgari 1975: 57; Wall 1994: 148. Synonymized with *Gasteruption laticeps* (Tournier) by Györfi and Bajári, 1962.

#### Type material.

Holotype of *Gasteruption laticeps* ♀ (MNHG) “Italie, Huet”, “Cn Tournier”, “Type”, “*Foenus laticeps* Tourn., ♀”. Lectotype of *Gasteruption foveolatum* here designated, ♂ (Naturhistorisches Museum, Bern), “Th. Steck”, “vii. [18]86, Siders [= Sierre, Wallis, Switzerland]”, “Schlettr. det.”, “Lectotypus des. Madl, 1987”; paralectotype from Italy *Gasteruption foveolatum* in MHNG. Lectotype of *Gasteruption foveolum* here designated: ♀ (MTMA), “[Hungary], Budapest, Kinestári-erdö, 1895.vii.29, Szépligeti %/ teste Papp J., 1986”, “Szépligeti, Kinestari / 985-7-29”, “*Gasteruption laticeps* Tourn., det. Bajari”, “*Gasteruption foveolum* Szépl., det. Stohl”, “Lectotypus *Gasteruption foveolum* Szépl. %/ des. Madl, 1987”, ovipositor sheath broken off; 3 ♀, 2 ♂ and one specimen without metasoma are paralectotypes from Hungary (Pilis-Maróth, Székes, Fehérvar, Kalocsa, Duna-Örs, S.-A. Ujhely), Romania (Nagyvárad) and Greece (Attica).

#### Additional material.

*Turkey (Capadocia, Ürgüp; Isparta, Egirdir Gölu, 5 km N of Akkecili, 920 m; Mansisa, 15 km SEE of Salihli, 170 m; Denizli, 35 km SSE of Denizli, 970 m; id., 10 km NE of Denizli, 290 m; id., 20 km NE of Denizli, Pamukkale, 1000 m; Burdur, 20 km SW of Burdur, 940 m; Nevsehir, 10 km S of Avanos, Göreme, 1000 m; Konya, Konya, Alaâdin Hill, 1050 m; Maras, Göksun, 1400 m; Bodrum, Salmakis; Kayseri, Göreme, 1000 m; Inner Anatolia, 8 km W of Karakaya, 230 m; Antalya, Cavusköyi, Andrasan, 50 m; id., Perge, 50 m).

#### Diagnosis.

**Figures 272–280.**
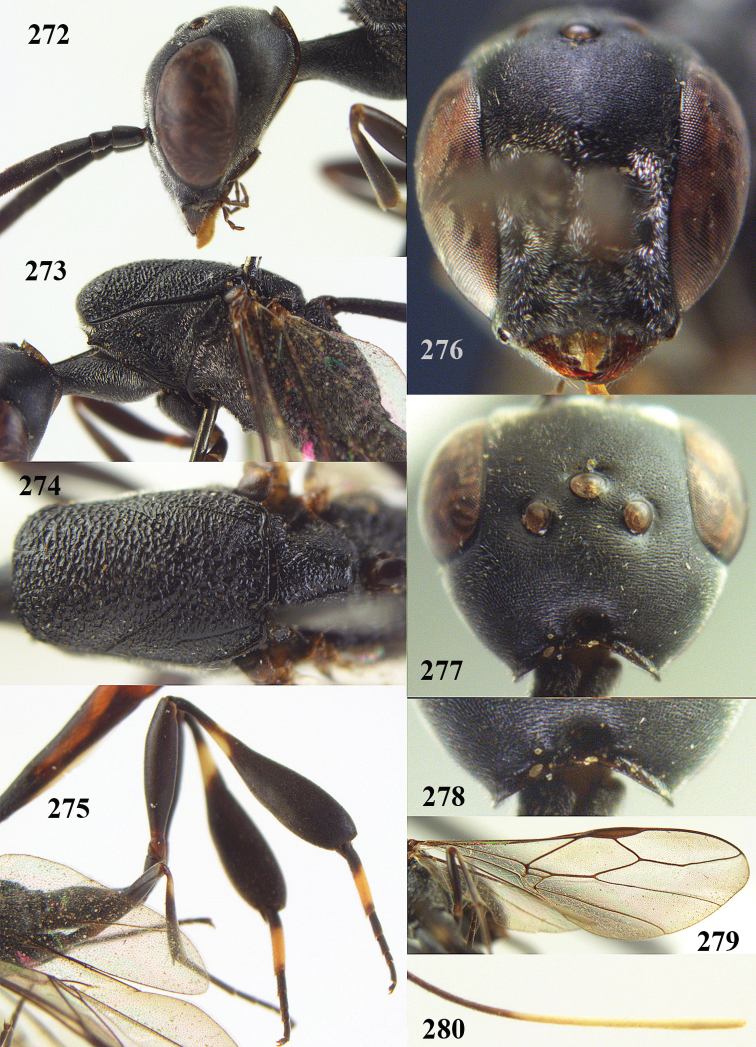
*Gasteruption laticeps* (Tournier), female, Italy. **272** head lateral **273** mesosoma lateral **274** mesonotum dorsal **275** hind leg **276** head anterior **277** head dorsal **278** detail of depression of vertex **279** fore wing **280** apex of ovipositor sheath.

Apex of ovipositor sheath with a distinct white or ivory band, 1.2–1.3 times as long as hind basitarsus; head with middle depression in front of occipital carina moderately deep and nearly round and no lateral depressions (Figs 277, 278, 283); occipital carina distinctly lamelliform and medium-sized to wide (Figs 272, 278); antesternal carina rather wide and lamelliform, distinctly elevated above mesosternum (Fig. 273); frons densely punctulate or densely very finely aciculate and without distinct interspaces; vertex more or less finely transversely aciculate and with satin sheen; head in dorsal view moderately narrowed (Figs 277, 283); propleuron with satin sheen, smooth, coriaceous or finely rugulose; ovipositor sheath 1.0–1.1 times as long as body and about 1.6 times as long as metasoma; pterostigma dark brown medially. Males have fourth antennal segment 0.8–0.9 times as long as second and third segments combined and apical antennal segment about as long as fourth segment (Fig. 282).

#### Distribution.

Europe, Iran, Turkey. New for the fauna of Turkey.

#### Biology.

Uncertain, according to Malyshev (1965) predator-inquiline of *Hylaeus* nests. Collected from mid-May till early September.

#### Notes.

Especially males may have the medio-posterior depression of the vertex shallowly impressed (Fig. 283) or nearly absent.

**Figures 281–283.**
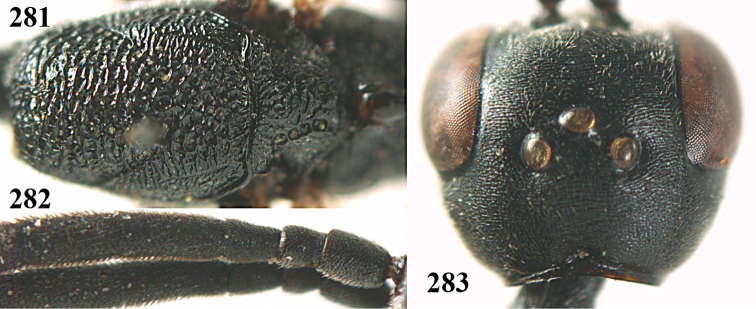
*Gasteruption laticeps* (Tournier), male, Italy. **281** mesonotum dorsal **282** basal antennal segments **283** head dorsal.

### Gasteruption lugubre

Taxon classificationAnimaliaHymenopteraGasteruptiidae

Schletterer, 1889 stat. rev.

Figs 284 –302

Gasteruption lugubre Schletterer, 1889: 391, 396, 412; Dalla Torre 1902: 1068; Szépligeti 1903: 370; Kieffer 1904a: 642, 1912: 262; Schmiedeknecht 1930: 378; Hedicke 1939: 16; Ferrière 1946: 236, 247; Wall 1994: 149 (as synonym of *Gasteruption diversipes* (Abeille de Perrin)).
Gasteruption floreum Szépligeti, 1903: 370, 372; Kieffer 1904a: 641, 1912: 267; Maidl 1923: 35; Schmiedeknecht 1930: 377; Hedicke 1939: 10; Györfi and Bajári 1962: 42, 51; Madl 1989a: 160, 1989b 42–43, 1990b 480; Wall 1994: 154–155; Scaramozzino 1995: 3; Pagliano and Scaramozzino 2000: 13, 19, 25. **Syn. n.**

#### Type material.

Holotype of *Gasteruption lugubre*, ♀ (ETHZ) “799", [according to original description from Switzerland, Wallis], “*Gasteruption lugubre* n. sp., Typ.”, “*Gasteruption assectator* (Linnaeus), ♀, C. Saure, det. 1999”. Lectotype of *Gasteruption floreum* here designated, ♂ (MTMA) “[Croatia], Buccari [= Bakar], 15.vi.”, “Jugoslavia”, “*Gasteruption floreum* Szépl., det. Stohl”, “Lectotypus *Gasteruption floreum* Szépl./ des. Madl, 1987”; 1 ♂ paralectotype (MTMA) “[Romania], Orsova, Transylvania”, is much damaged and probably belongs to *Gasteruption undulatum* (Abeille de Perrin).

#### Additional material.

*Turkey (Pasli, 50 km S of Kars; Nevşeher, 10 km E of Ürgüp, W of Aksalur, 1350 m).

#### Diagnosis.

**Figures 284–292.**
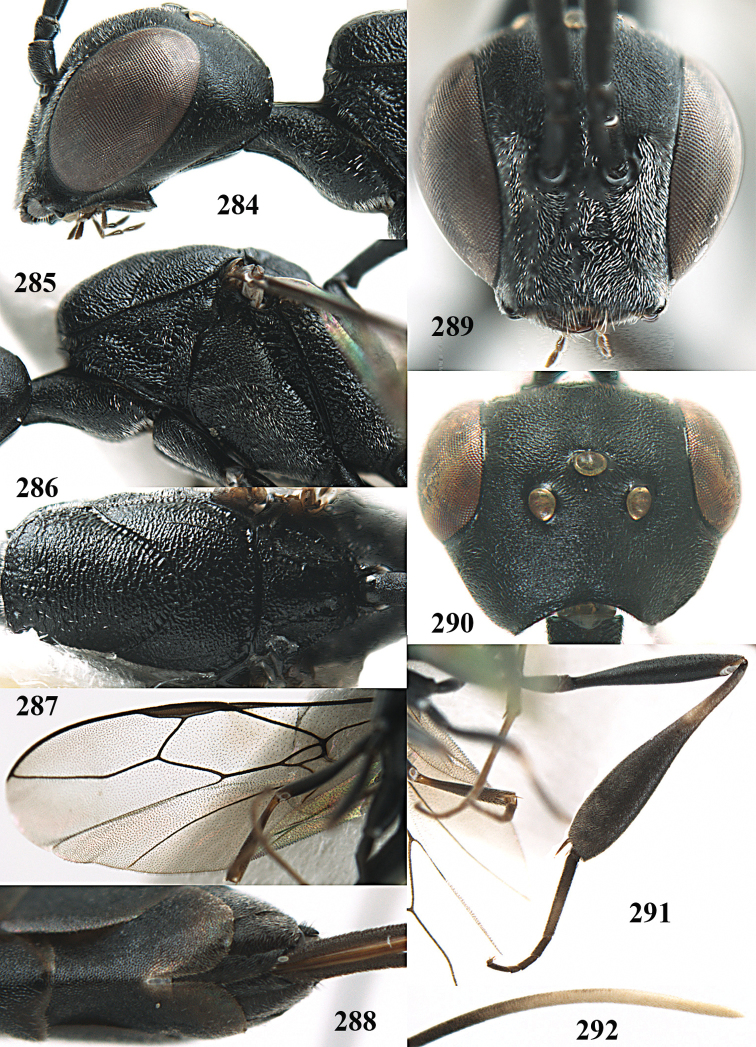
*Gasteruption lugubre* Schletterer, female, Montenegro. **284** head lateral **285** mesosoma lateral **286** mesonotum dorsal **287** fore wing **288** hypopygium ventral **289** head anterior **290** head dorsal **291** hind leg **292** apex of ovipositor sheath.

Apex of ovipositor sheath with a distinct white or ivory band, 1.5–1.9 times as long as hind basitarsus; dorsally head in front of occipital carina with minute medial depression (Fig. 290), in lateral view flat and occipital carina very narrow medio-dorsally and non-lamelliform (Fig. 284) or narrowly lamelliform; antesternal carina with narrow lamelliform rim, antesternal carina and prepectal carina medio-ventrally similarly developed (Fig. 295); head ventrally elongate below eyes (Figs 289, 298), gradually narrowed behind eyes in dorsal view and temples slightly convex (Fig. 290); temple about half as long as eye in dorsal view; fourth and fifth antennal segments of ♀ 1.6–1.7 and 1.4–1.6 times as long as third segment, respectively; fourth segment of ♀ 1.0–1.1 times as long as second and third segments combined; head parallel-sided below eyes and malar space about 0.9 times as long as second antennal segment (= pedicellus); vertex and frons rather matt, finely and densely aciculate-rugulose; propleuron with satin sheen, 0.8 times as long as mesoscutum in front of tegulae, densely rugose or rugulose and stout (Fig. 286); pronotal side largely rugulose; antero-lateral teeth of pronotum small and rather acutely angled; mesoscutum slender and sparsely setose (Fig. 286), with satin sheen and largely finely and densely transversely rugulose, without separate punctures and medio-posteriorly rugose; hind femur narrow and nearly parallel-sided (Fig. 291); hind coxa mainly granulate, but rugulose dorsally (Fig. 297); ovipositor sheath 0.8–1.0 times as long as body, 1.2–1.3 times as long as metasoma and 3.7–3.9 times as long as hind tibia; hind coxa and pronotal side dark brown or blackish; hind basitarsus dark brown or black (dark form) or partly ivory (pale form); length of body 8–11 mm. Male has third antennal segment twice as long as second segment, fourth segment 1.6 times as long as third segment and as long as second and third segments combined (Fig. 302); malar space slightly longer than basal width of third antennal segment.

#### Distribution.

Mountainous parts of C. Europe and Turkey. New for the fauna of Turkey.

#### Biology.

Unknown. Collected in June-July and rarely encountered.

#### Notes.

The holotype of *Gasteruption lugubre* is mutilated and the metasoma is missing. According to the original description the ovipositor sheath is longer than the metasoma, shorter than the body and black with white apex. And the fourth antennal segment (“drittes Geisselglied”) of the female holotype as long as second and third segments combined. The surviving part shows an elongate head and in combination with the characters mentioned above it is obvious that it concerns most likely the dark form of *Gasteruption floreum* Szépligeti.

**Figures 293–302.**
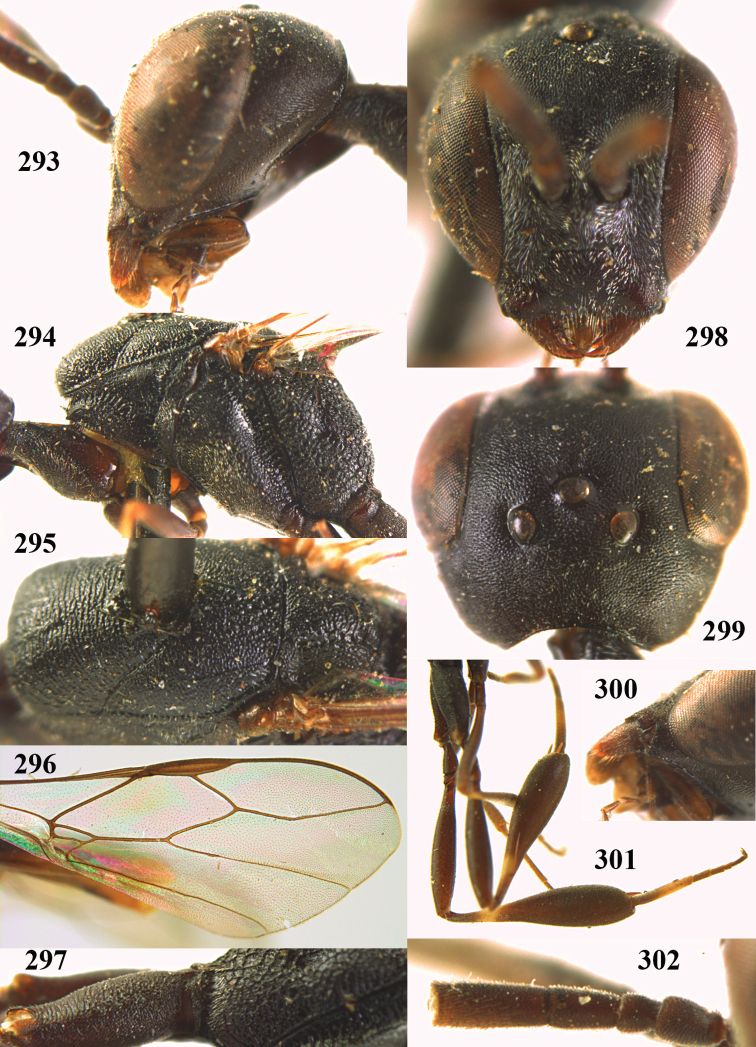
*Gasteruption lugubre* Schletterer, male, lectotype of *Gasteruption floreum* Szépligeti. **293** head lateral **294** mesosoma lateral **295** mesonotum dorsal **296** fore wing **297** hind coxa dorsal **298** head anterior **299** head dorsal **300** detail of malar space lateral **301** hind leg **302** basal antennal segments.

### Gasteruption merceti

Taxon classificationAnimaliaHymenopteraGasteruptiidae

Kieffer, 1904

Figs 303 –314

Foenus pyrenaicus ; Tournier 1877: ix; Wall 1994: 149.
Faenus pyrenaicus ; Abeille de Perrin 1879: 262, 266, 267.
Trichofoenus pyrenaicus ; Kieffer 1912: 213, 214; Maidl 1923: 34; Hedicke 1939: 44; Ferrière 1946: 236, 238, 239; Malyshev 1965: 265.
Gasteruption pyrenaicum ; Schletterer 1885: 283, 1889: 382, 388, 394, 397, 405; Szépligeti 1903: 368; Kieffer 1912: 214; Schmiedeknecht 1930: 379, 380; Ferrière 1946: 239; Stohl 1947a: 8, 1947b: 278; Šedivý 1958: 35, 36, 42; Györfi and Bajári 1962: 46, 49; Malyshev 1965: 265; Schmidt 1969: 295; Oehlke 1984: 169, 170, 180; Kozlov 1988: 244, 247; Wall 1994: 149; Yildirim et al. 2004: 1351.
Gasteryption pyrenaicum ; Semenov 1892: 201.
Gasteruption merceti Kieffer, 1904a: 639; Hedicke 1939: 43; Madl 1988a: 13, 16, 1989b 44, 1990a 128, 1990b 480; Rey del Castillo 1990: 133; Kofler and Madl 1990: 322; Wall 1994: 158; Scaramozzino 1995: 3; Jakubzik and Colln 1998: 211; Pagliano and Scaramozzino 2000: 11, 15, 29; Saure 2001: 29; van der Smissen 2010: 373; van Achterberg 2013: fig. 178.
Trichofoenus merceti ; Kieffer 1912: 213, 214.
Gasteruption trichotomma Kieffer, 1904a: 645; Hedicke 1939: 45; Wall 1994: 165. **Syn. n.**
Trichofoenus trichotomma ; Kieffer 1912: 214, 215.
Gasteruption palaestinum Pic, 1916: 23; Hedicke 1939: 18. **Syn. n.**
Gasteruption jekylljaechi Madl, 1987a: 403, 1987b: 22, 1988b 406, 1988c 38; Wall 1994: 148; Yildirim et al. 2004: 1351. Synonymized with *Gasteruption merceti* Kieffer by Madl, 1989b.

#### Type material.

Lectotype of *Gasteruption merceti* here designated ♀ (MNCN) “[Spain], Los Molinos, G. Mercet”, “MNCN_Ent. Cat. No. 43301”, “Lectotipo, *Gasteruption merceti* Kieff., C. Rey”, “*Gasteruption Merceti* K.”, “Coleccion Ga. Mercet”, “MNCN Cat. Tipos No. 2046”; 1 ♂ paralectotype (MNCN) from Madrid, MNCN_Ent. Cat. No. 43302; 1 ♀ paralectotype (MTMA), “Madrid, G. Mercet”, “*Gasteruption Merceti*”, “*Gasteruption Merceti* Kieff., type”, and lectotype label by Dr J. Papp (1980, unpublished). Holotype of *Gasteruption jekylljaechi* ♀ (NMW), “N. Ö[sterreich], Aspern, Sach”, “*Gasteruption pyrenaicum* Guér., J. Pasteels, det. 1953", and type label by Madl, 1986. Holotype of *Gasteruption trichotomma* ♀ (MNHN), “[Algeria], Oran”, “Museum Paris, EY000 000 2453”, “Museum Paris, Collection Ernest André, 1914”, “*Gasteruption trichotomma*”, “Kieffer det.”. “Typus?", and holotype label by CvA. Holotype of *Gasteruption palaestinum* ♂ (MNHN), “[Israel], Jericho”, “Museum Paris, EY0000003922”, “Type”, “Museum Paris, coll. Pic,”, “voir species *hungaricum*”, “*palaestinum* Pic”, “[illegible] special”, “Monotypus det. Madl, 1987", and holotype label by CvA.

#### Additional material.

*Iran (Alborz, Karaj; Qazvin, Zereshk Road; Mazandaran, Noor; Gilan, Orkom; Teheran, Rayne; Azerb. e Garbi, Serou, 1650 m; Kerman, 30 km S of Sirjan, 1730 m); **Turkey** (Ankara, Beynam; Konya, 30 km S of Aksehir; 50 km S of Kars Parsli; 20 km NW of Igdir; Kabahaydar Urfa; 20 km E of Gurun, Mezikiran Gecidi; Capadocia, Ürgüp; Birecik, Halfeti; Antakya; 15 km E of Malatya; 60 km E of Mut Kirobasi; Osmaneli; near Akyaka, 40 m; Burdur, 20 km SW of Burdur, 940 m; id., 5 km NE of Yesilova, 1060 m; id., 28 km SEE of Burdur, 1350 m; Sivas, 45 km E of Yarhisar; Göreme; near Halfeti; Gevas, Van Gölü; Isparta, Karakus Dagi centr., 1460 m; Mansisa, 30 km SEE of Salihi, 430 m; Denizli, 35 km SEE of Denizli, 970 m; Nevsehir, 20 km S of Nevsehir, Kaymakli, 1200 m; Hakkari, Mt. Sat, SW of Yüksekova, Varegös, 1650 m; Van, 30 km N of Baskale, 2700 m; Kayseri, Pinarbasi, 1500 m; Nevsehir, 5 km S of Avanos, Zelve, 1000 m; Agri, 30 km W of Eleskirt, 2200 m; Maras, Göksun, 1400 m; Diyarbakir, Diyarbakir, 650 m; SSE of Milas, Çamköy – Sek; Inner Anatolia, 5 km W of Koyulhisar).

#### Diagnosis.

**Figures 303–311.**
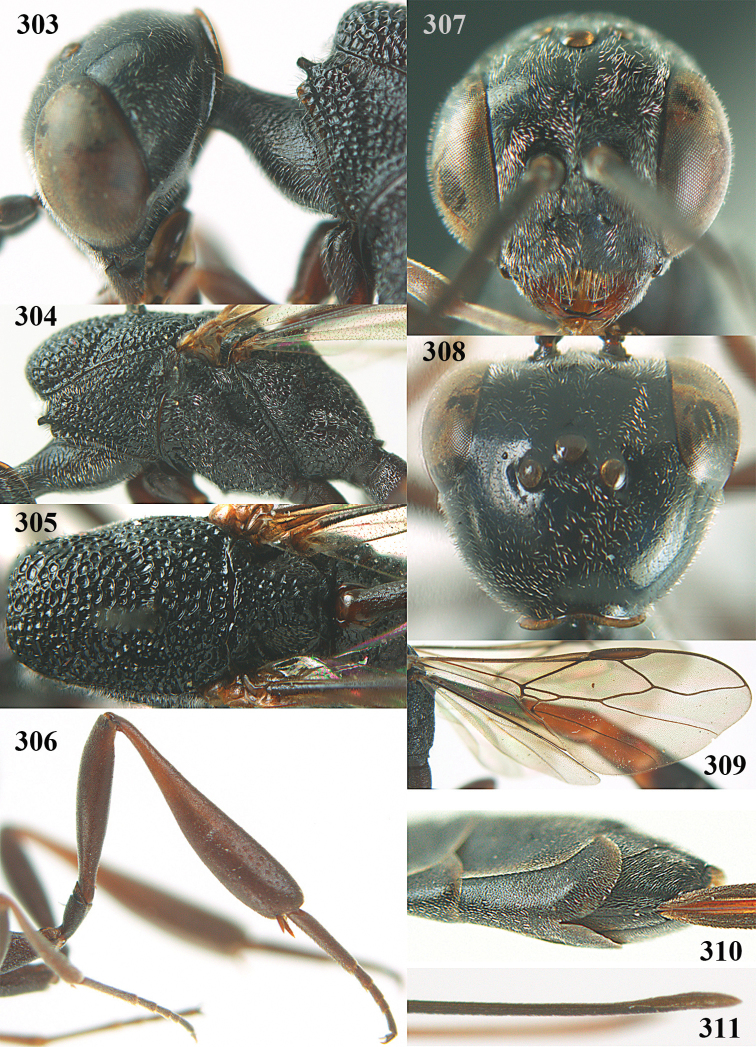
*Gasteruption merceti* Kieffer, female, France. **303** head lateral **304** mesosoma lateral **305** mesonotum dorsal **306** hind leg **307** head anterior **308** head dorsal **309** fore wing **310** hypopygium latero-ventral **311** apex of ovipositor sheath.

Apex of ovipositor sheath blackish or slightly brownish (Fig. 311), if rather pale apically then pale part distinctly shorter than hind basitarsus; ovipositor sheath 2.6–3.8 times as long as hind tibia and 0.7–1.2 times as long as metasoma; occipital carina at least 0.3 times as wide as diameter of posterior ocellus; eyes often densely setose; temple strongly elongate (Figs 303, 308, 314), about as long as eye in dorsal view, but sometimes 0.8 times; vertex smooth or nearly so, at most with very superficial punctulation and with satin sheen; medial antennal segments slightly widened; face rather wide (Fig. 307); temples strongly narrowed behind eyes in dorsal view (Fig. 308); occipital carina lamelliform, medium-sized and distinctly upcurved (Figs 303, 308); vertex hardly bulging near occipital carina (Fig. 303); genal bridge at most half as long as third antennal segment; antesternal carina distinctly lamelliform and moderately wide (Fig. 304); pronotum with pair of rather large acute protuberances antero-laterally (Fig. 303); propleuron 0.7–0.8 times distance from tegulae to anterior border of mesoscutum, with satin sheen; mesoscutum coarsely rugose-reticulate and sometimes reddish brown; hind tibia rather slender (Fig. 306); hind basitarsus slender to rather stout; incision of hypopygium deep, often slit-like (Fig. 310). Males have third antennal segment about twice as long as second segment and fourth antennal segment about 0.7 times as long as second and third segments combined (Fig. 313).

#### Distribution.

Central and South Europe, N. Africa, Israel, Turkey, Iran. New for the fauna of Iran.

#### Biology.

Predator-inquiline of *Ceratina* spp. Collected in April-July and September.

#### Notes.

Sometimes the mesosoma is anteriorly (male from Kerman, Iran) or largely reddish brown. Eastern populations have the occipital carina lamelliform but narrower (width of carina medio-dorsally 0.2–0.4 times transverse diameter of posterior ocellus) than in European populations (0.5–0.6 times transverse diameter of posterior ocellus); the differences are clinal.

**Figures 312–314.**
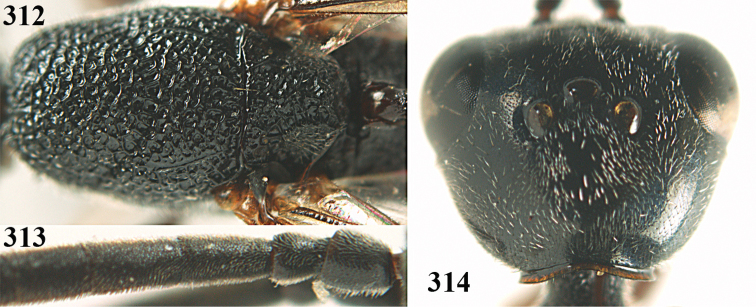
*Gasteruption merceti* Kieffer, male, France. **312** mesonotum dorsal **313** basal antennal segments **314** head dorsal.

### Gasteruption minutum

Taxon classificationAnimaliaHymenopteraGasteruptiidae

(Tournier, 1877)

Figs 315 –330

Foenus minutus Tournier, 1877: ix; Capron 1880: 89; Schletterer 1889: 398 (as synonym of *Gasteruption assectator* (Linnaeus)).
Faenus minutus ; Abeille de Perrin 1879: 265, 267, 277.
Gasteruption minutum ; Kieffer 1912: 257; Hedicke 1939: 16; Ferrière 1946: 235, 238, 240; Hellén 1950: 4; Crosskey 1951: 295; Šedivý 1958: 36, 37, 40; Schmidt 1969: 295; Hedqvist 1973: 185; Dolfuss 1982: 24; Alexander 1983: 150; Allen 1983: 82; Madl 1987a: 403, 1987b: 23, 1988c 38, 1989a 160, 1989b 44, 1990a 128, 1990b 480, 482; Kozlov 1988: 245, 247; Neumayer et al. 1999: 220; Kofler and Madl 1990: 322; Wall 1994: 159; Scaramozzino 1995: 3; Pagliano and Scaramozzino 2000: 11, 19, 30; Saure 2001: 29; Wisniowski 2004: 118; van der Smissen 2010: 373; van Achterberg 2013: fig. 172.
Foenus longigena Thomson, 1883: 849; Ferrière 1946: 240; Hedqvist 1973: 185; Madl 1988c: 39. Synonymized with *Gasteruption minutum* (Tournier) by Ferrière, 1946, Schmidt, 1969 and Hedqvist, 1973.
Gasteruption longigena ; Schletterer 1889: 399; Dalla Torre 1902: 1068; Kieffer 1912: 270; Schmiedeknecht 1930: 380, 381; Hedicke 1939: 16; Hellén 1950: 4 (as *G. ”longiserra*”, and as synonym of *Gasteruption minutum* (Tournier)); Hedqvist 1973: 185 (lectotype designation); Wall 1994: 149.
Foenus borealis Thomson, 1883: 849; Hedicke 1939: 7; Hedqvist 1973: 181, 182 (invalid lectotype designation); Wall 1994: 148. Synonymized with *Gasteruption assectator* (Linnaeus) by Schletterer, 1889. **Syn. n.**
Gasteruption boreale ; Schletterer 1885: 303.
Gasteruption abeillei Kieffer, 1912: 228, 231, 251; Hedicke 1939: 5; Ferrière 1946: 235, 240; Leclercq 1948: 75; Wall 1994: 148. Synonymized with *Gasteruption assectator* (Linnaeus) by Madl, 1989a. **Syn. n.**

#### Type material.

Lectotype of *Gasteruption minutum* here designated, ♀ (MHNG) “[Switzerland], Peney, [near Genève], vii.[18]75”, “Cn Tournier”, “Type”, *Foenus minutus* Tourn., ♀”, “Lectotypus, des. Madl, 1987”; Paralectotypes (4 ♀, MHNG) and all from Peney, 2 ♀ collected vii.1876, 1 ♀ vii.1875 and 1 ♀ 10.vi.1875; the paralectotypes from France and Italy were not found. Lectotype of *Gasteruption longigena* ♀, (ZIL) “Rõn”, [= Rönnemölla, Skane-Norrland], “Lectotypus *Foenus longigena* Thoms., ♀, K.-J. Hedqvist, det. 1972”. Lectotype of *Gasteruption boreale* here designated ♂ (ZIL) from Lappland (“Lpl.”, “*borealis*”); the female lectotype from Norway designated by Hedqvist (1973) is invalid because it is not from the type locality. The type series of *Gasteruption abeillei* from Pereneese and Landes should be in the Abeille de Perrin collection, but no specimen was found. Kieffer gives in his description as main character the short ovipositor (“Bohrer etwas kürzer als das 1. Segment”), which fits better with *G. boreale/minutum* than with *Gasteruption assectator*.

#### Additional material.

*Iran (Kerman, Sirac, 1640 m; Isfahan, Najafabad); *Turkey (27 km SE of Aksaray, Ihlara; Van, 30 km N of Baskale, 2700 m; Tunceli, 17 km W of Ovacik, 1250 m; Kerman, Sirac, 1640 m; Bolu, lake).

#### Diagnosis.

**Figures 315–322.**
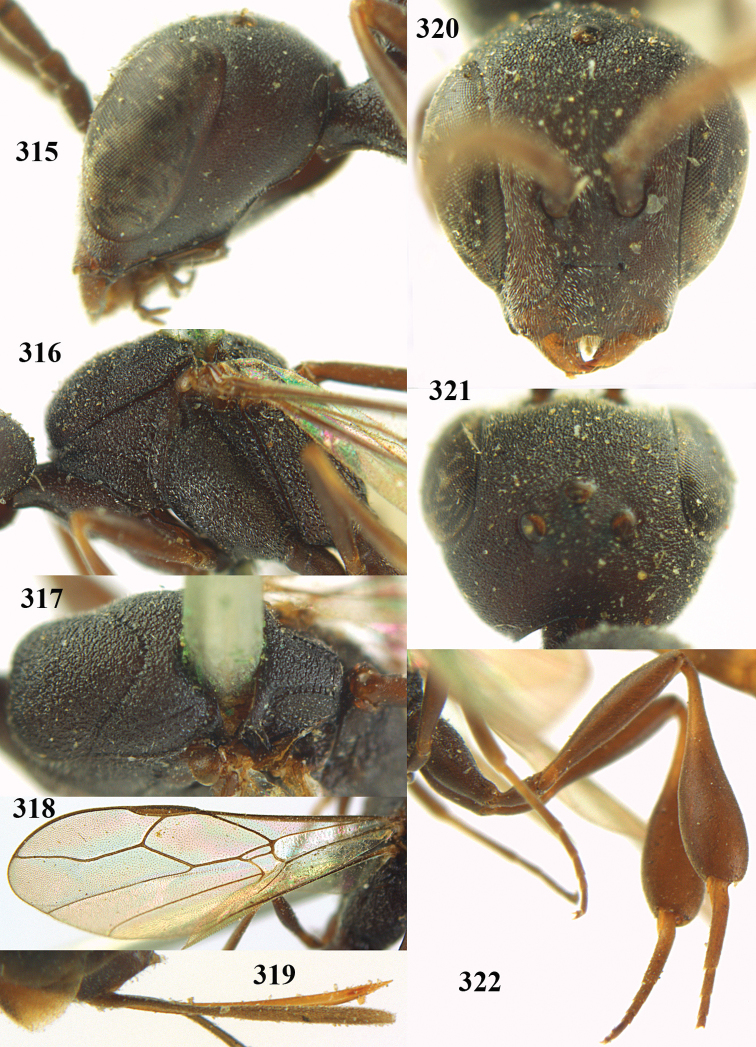
*Gasteruption minutum* (Tournier), female, lectotype. **315** head lateral **316** mesosoma lateral **317** mesonotum dorsal **318** fore wing **319** ovipositor sheath **320** head anterior **321** head dorsal **322** hind leg.

**Figures 323–330.**
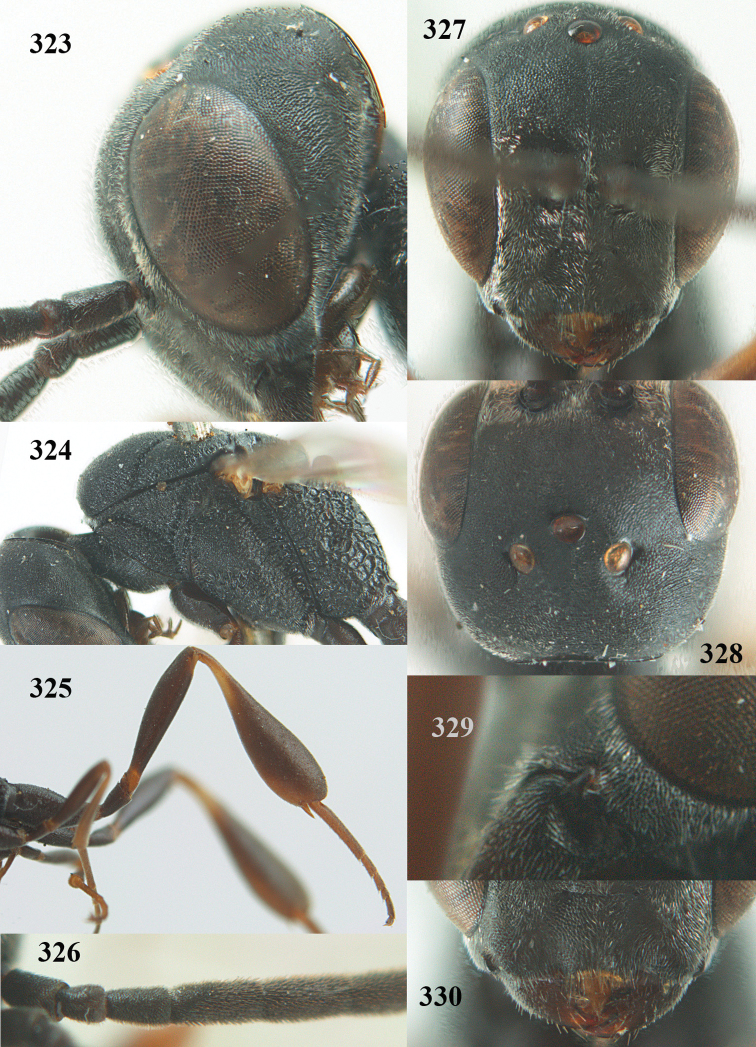
*Gasteruption minutum* (Tournier), male, lectotype of *Gasteruption boreale* (Thomson). **323** head lateral **324** mesosoma lateral **325** hind leg **326** basal antennal segments **327** head anterior **328** head dorsal **329** detail of malar space lateral **330** clypeus and malar space anterior.

Apex of ovipositor sheath blackish or slightly brownish, if rather pale apically then pale part distinctly shorter than hind basitarsus; ovipositor sheath 0.5–0.9 times as long as hind tibia and 0.3–0.6 times as long as hind tibia and tarsus combined; occipital carina obsolescent (Figs 315, 323) and hardly protruding ventro-posteriorly; head in anterior view protruding below lower level of eyes by about basal width of mandible and mandibular condylus distinctly below lower level of eyes (Figs 320, 327), either subparallel (= typical form) or narrowed ventrally (f. *boreale*)); in lateral view condylar incision of malar space remains far removed from eye (Fig. 329); antesternal carina narrow; head, mesosoma laterally and scapus black; clypeus with small depression or depression obsolescent; apical antennal segment at most 1.2 times as long as third antennal segment and its colour similar to colour of medial segments; antenna of ♀ slightly shiny and blackish or dark brown; mesoscutum and head similarly coriaceous, at most mesoscutum superficially rugulose; hind coxa very densely and finely sculptured dorsally; hind tibia stout, with a distinct subbasal ivory ring and swollen, resulting in a distinctly convex ventral border (Fig. 322); hind basitarsus stout to slender (Figs 322, 325); hind tibial spurs yellowish-brown or brown; hind tarsus brown, dark brown or blackish; incision of hypopygium shallow. Males have third antennal segment usually rather long, significantly longer than second segment (Fig. 326) and fourth antennal segment shorter than second and third segments combined.

#### Distribution.

Europe, Iran, Turkey. New for the fauna of Iran and Turkey.

#### Biology.

Probably predator-inquiline of *Hylaeus* nests (Wall, 1994). Collected from end of May till early August.

### Gasteruption nigrapiculatum

Taxon classificationAnimaliaHymenopteraGasteruptiidae

van Achterberg sp. n.

http://zoobank.org/00BD104B-26C2-488C-BF69-2F78C192B812

Figs 331 –345

#### Type material.

Holotype (RMNH), ♀, “N. **Iran:** Qazvin, Zereshk Road, MT [= Malaise trap] 3, 7–22.vi.2011, A. Mohammadi, RMNH’12“. Paratypes (1 ♀ + 1 ♂): 1 ♂ (RMNH), “N. Iran: Alborz, Shahrestanak, Chalous Road, MT 28, 15–22.vi.2010, S. Farahani, RMNH‘12“; 1 ♀ (BZL), “**Jordan** NW, N of Janesh, 15.v.2010, Snižek“.

#### Diagnosis.

Head weakly convex dorsally, in front of occipital carina without medio-posterior depression; face moderately wide (Fig. 335); frons with satin sheen and densely finely punctulate; occipital carina narrowly lamelliform and dark brown; vertex rather shiny and transversely finely aciculate, anteriorly punctulate and without punctures; mandible yellowish brown basally, but somewhat darkened dorso-basally; propleuron 0.8 times as long as mesoscutum in front of tegulae; antesternal carina medium-sized lamelliform, directed posteriorly; middle lobe of mesoscutum shiny, densely transversely rugulose, without smooth interspaces, medio-posteriorly reticulate-rugose, lateral lobe shiny, densely obliquely rugulose and medially irregularly punctate (Fig. 333); scutellum shiny, partly smooth medially and with indistinct transverse rugulae and anteriorly punctate-rugulose; mesosoma laterally (except pronotal side ventrally) silvery pilose (Fig. 332); middle lobe rather protuberant (Fig. 333); hind basitarsus entirely dark brown, darker than yellowish brown hind tibial spurs (Fig. 334); hind tibia rather slender, outer side with punctures and short dark bristles and with large subbasal ivory patch (Fig. 334); ovipositor sheath 0.9 times as long as body, 1.3–1.4 times as long as metasoma, 2.5–2.7 times as long as hind tibia and tarsus combined and 4.1 times hind tibia; apex of ovipositor sheath dark brown or brown; length of body 9–11 mm; paramere ivory apically (Fig. 345).

#### Description.

Female, length of body 9.1 mm (of fore wing 4.4 mm).

**Figures 331–339.**
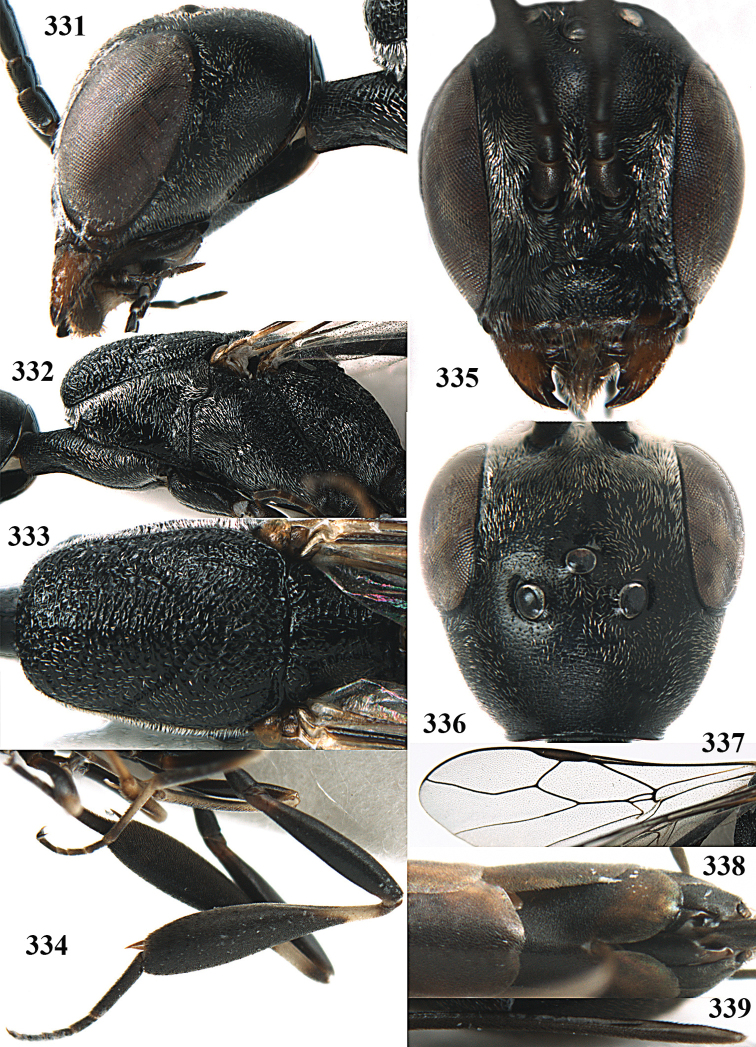
*Gasteruption nigrapiculatum* sp. n., female, holotype. **331** head lateral **332** mesosoma lateral **333** mesonotum dorsal **334** hind leg **335** head anterior **336** head dorsal **337** fore wing **338** hypopygium ventral **339** apex of ovipositor sheath.

*Head*. Head weakly convex dorsally, posteriorly rather directly narrowed, without medio-posterior depression; face and anteriorly frons conspicuously silvery pilose; occipital carina narrowly lamelliform, dark brown (Figs 331, 337); third and fourth antennal segments 1.6 and 2.3 times as long as second segment, apical segment 1.5 times as long as penultimate segment; face moderately wide (Fig. 335); frons with satin sheen and densely finely punctulate; vertex rather shiny and transversely finely aciculate, anteriorly punctulate and without punctures; ventrally head not enlarged in anterior view, malar space 0.2 times length of pedicellus.

*Mesosoma*. Length of mesosoma 1.9 times its height; propleuron 0.8 times as long as mesoscutum in front of tegulae, silvery pilose and moderately stout posteriorly; laterally pronotum largely smooth postero-ventrally and rugose antero-ventrally and shiny, ventrally without pilosity; side of pronotum with obsolescent tooth antero-ventrally; antesternal carina medium-sized lamelliform, directed posteriorly; middle lobe of mesoscutum shiny, densely transversely rugulose, without smooth interspaces, medio-posteriorly reticulate-rugose, lateral lobe shiny, densely obliquely rugulose and medially irregularly punctate (Fig. 333); notauli rather shallow; scutellum shiny, partly smooth medially and with indistinct transverse rugulae and anteriorly punctate-rugulose; mesopleuron and metapleuron silvery pilose (Fig. 333); eyes inconspicuously setose.

*Legs*. Length of hind femur, tibia and basitarsus 5.1, 4.7 and 5.2 times their width, respectively; hind tibia rather slender (Fig. 334); fore coxa close to mesopleuron; hind coxa shiny and rugulose dorsally; hind basitarsus moderately slender, as long as remainder of tarsus and distinctly widened in dorsal view.

*Metasoma*. Ovipositor sheath 0.9 times as long as body, 1.4 times as long as metasoma, 2.7 times as long as hind tibia and tarsus combined and 4.1 times as long as hind tibia.

*Colour.* Black; metasoma dark brown, but second-sixth tergites yellowish brown apically and ventrally and sternites basally and apically brown, but apical half of hypopygium largely brown; mandible (but dorso-basally darkened) and tegulae yellowish brown; fore and middle femora apically and tibiae basally and basitarsi and hind tibia subbasally ivory; remainder of legs (except coxae) largely dark brown; palpi, pterostigma and hind basitarsus entirely dark brown; hind tibial spurs yellowish brown and paler than base of hind basitarsus; apex of ovipositor sheath dark brown; wing membrane subhyaline.

*Male.* Third antennal segment 1.2 times as long as second segment, fourth segment 2.3 times as long as third segment and 1.2 times as long as second and third segments combined, fifth segment as long as fourth segment (Fig. 342); mandible yellowish brown; scutellum partly smooth and shiny medially and mesoscutum irregularly rugose; antesternal carina medium-sized; hind tibia dark brown ventrally and with subbasal ivory band; hind tibial spurs paler than base of basitarsus; hind tarsus dark brown; hind coxa transversely rugulose dorsally; apex of paramere ivory (Fig. 345).

*Variation.* Length of body of ♀ 9.1–11.4 mm (of ♂ 9.5 mm); mesoscutum in paratype somewhat coarser sculptured than in holotype; apical half of hypopygium dark brown or largely yellowish brown; ovipositor sheath 0.9 times as long as body, 1.3–1.4 times as long as metasoma, 2.5–2.7 times as long as hind tibia and tarsus combined and 4.1 times hind tibia.

#### Distribution.

Iran, Jordan.

#### Biology.

Unknown. Collected in May-June.

#### Etymology.

Derived from “nigra”, (Latin for “black”) and “apiculus”, (Latin for “small top”), because of the blackish apex of the ovipositor sheath.

**Figures 340–345.**
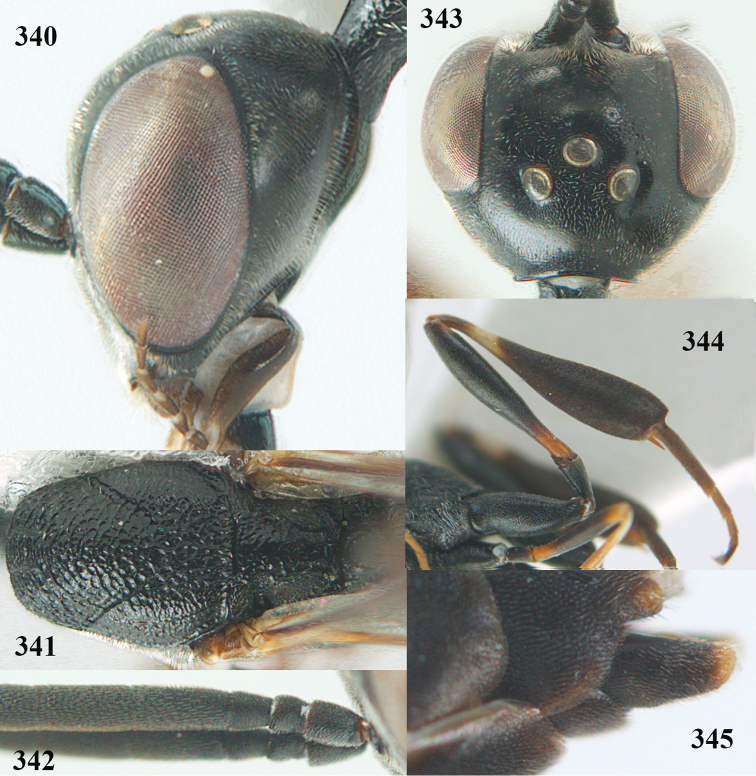
*Gasteruption nigrapiculatum* sp. n., male, paratype. **340** head lateral **341** mesonotum dorsal **342** basal antennal segments **343** head dorsal **344** hind leg **345** genitalia lateral.

### Gasteruption nigrescens

Taxon classificationAnimaliaHymenopteraGasteruptiidae

Schletterer, 1885

Figs 346 –361

Gasteruption vagepunctatum var. nigrescens Schletterer 1885: 288.
Gasteruption nigrescens Schletterer, 1889: 391, 396, 420; Kieffer 1904a: 645, 1912: 250; Dalla Torre 1902: 1069; Schmiedeknecht 1930: 379; Hedicke 1939: 17; Ferrière 1946: 236, 239, 243; Šedivý 1958: 35, 36, 41; Györfi and Bajári 1962: 46, 50; Madl 1989b: 44, 1990a 129, 1990b 480; Wall 1994: 160; Scaramozzino 1995: 3; Pagliano and Scaramozzino 2000: 13, 17, 30; Schmid-Egger and Saure 2010: 40.
Gasteryption nigrescens ; Semenov 1892: 208.
Gasteryption foveiceps Semenov, 1892: 205–206. **Syn. n.**
Gasteruption foveiceps ; Dalla Torre 1902: 1067; Szépligeti 1903: 369; Kieffer 1912: 246; Hedicke 1939: 11.
Gasteruption caudatum Szépligeti, 1903: 369, 371; Kieffer 1904a: 639, 1912: 260; Schmiedeknecht 1930: 376; Hedicke 1939: 8; Györfi and Bajári 1962: 42; Malyshev 1965: 247; Madl 1990a: 129; Wall 1994: 148. Synonymized with *Gasteruption nigrescens* Schletterer by Madl, 1989b.

#### Type material.

Holotype of *Gasteruption nigrescens* (♀ from Italy, Toscana, Mt. Falterone) not found: (?NMW) and probably lost. Lectotype of *Gasteruption caudatum* here designated: ♀ (MTMA), “[Hungary]. Nagyvárad, leg. Mocsáry %/ teste Papp J., 1986”, “Nvarad, Mocsary”, “*Gasteruption caudatum* Szépl., det. Stohl”, “Lectotypus *Gasteruption caudatum* Szépl. / des. Madl 1987”; paralectotype ♀ from Pápa (Hungary) and collected by Wachsmann. Holotype of *Gasteruption foveiceps* ♀ (ZISP) from Ukraine (Charkov) examined.

#### Additional material.

*Iran (Alborz, Chalous Road, Shahrestanak; Karaj, Sarziarat; Qazvin, Zereshk Road; Azer. e Sh., Sis, 10 km E of Shabestar, 1540 m; Boyer-A. o Kohg., Kuh Gol near Sisakht, 2500 m); **Turkey** (50 km S of Kars, Pasli; Konya, 30 km S of Aksehir; 20 km W of Van; 25 km E of Malatya, Kopeksiz; Muradiye; Sivas, near Gökpinar, 10 km S Gürün, 1500 m; Sivas, Cumhuriyet Univ.; Sivas, 45 km E of Yarhisar; 15 km E of Malatya; 20 km E of Gurun, Mezikiran Gecidi; Konya, 30 km S of Aksehir; Yüksekova; 15 km W of Refahye, W of Erzibcan, 1600 m; near Karabulak; Akyaka, 3 m; Avgadi, 30 km NW of Erdemli, 1300 m; Nemrut Dagi, Karadut; Hakkari, Aksali, 35 km S of Hakkari, 1700 m; Bolu, near lake; Isparta, Egirdir Gölu, 5 km N of Akkecili, 920 m; id., 8 km NE of Isparta, 1020 m; Kütahya, 20 km NEE of Kütahya; Mansisa, 35 km NW of Salihli, 900 m; 60 km W of Konya, Erflatun Pinar; Bursa, near Caglian; Cornelek, 40 km E of Mut; Burdur, 28 km SEE of Burdur, 1350 m; id., 20 km SW of Burdur, 940 m; id., 5 km NE of Yesilova, 1060 m; Van, Van, 1800 m; Agri, 30 km W of Eleskirt, 2200 m; Nevsehir, 5 km S of Avanos, Zelve, 1000 m; id., 10 km S of Avanos, Göreme, 1000 m; Ankara, Kizilcohaman, 1100 m; Adiyaman, Gölbasi, 900 m; Erzurum, Tortum, 1700 m; Hakkari, Mt. Sat, SW of Yüksekova, Varegös, 1650 m; Sivas, near Gökpinar, 10 km S of Gürün, 1500–1700 m).

#### Diagnosis.

**Figures 346–354.**
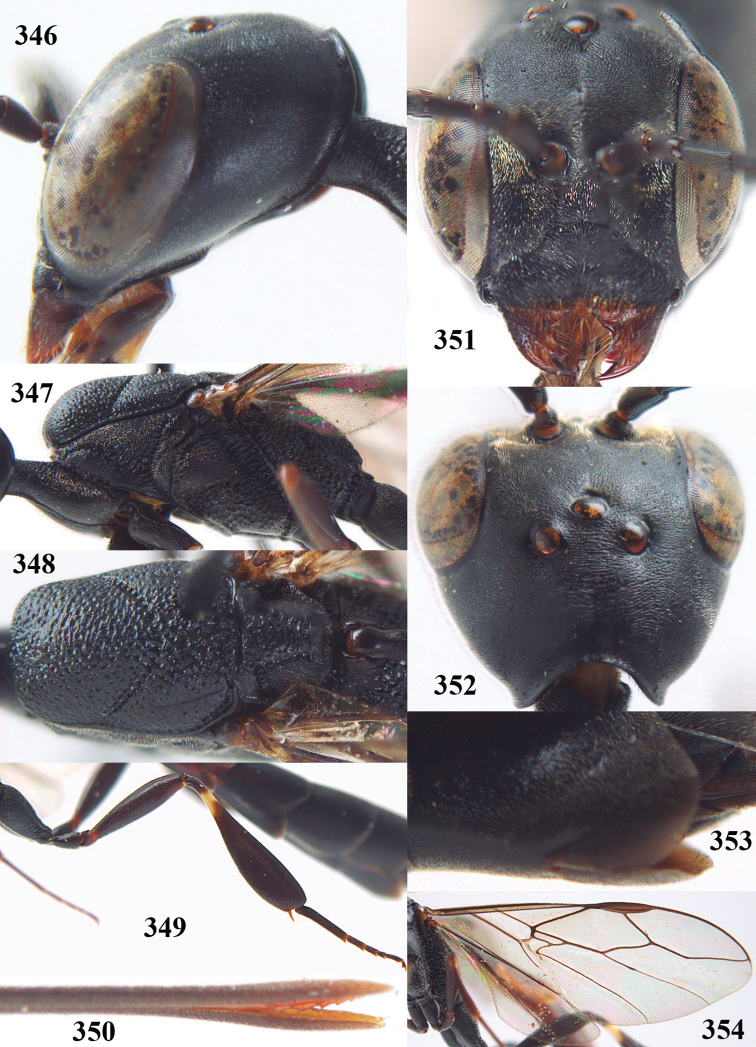
*Gasteruption nigrescens* Schletterer, female, Montenegro. **346** head lateral **347** mesosoma lateral **348** mesonotum dorsal **349** hind leg **350** ovipositor sheath **351** head anterior **352** head dorsal **353** hypopygium latero-ventral **354** fore wing.

Apex of ovipositor sheath dark brown, light brown (Fig. 350) or ivory, pale part 0.1–0.9 times as long as hind basitarsus; vertex slightly impressed or flattened medio-posteriorly in front of occipital carina; antesternal carina usually narrow; length of propleuron 0.8–0.9 times distance between tegulae and anterior border of mesoscutum (Fig. 347); occipital carina rather wide. but narrower than diameter of posterior ocellus (Figs 346, 352, 360); vertex medio-basally in front of occipital carina flat, without impression; third and fourth antennal segments rather stout; third antennal segment of female 1.5–1.7 times as long as second segment; segments of apical half of antenna rather long and ventrally black; frons more or less coriaceous, without discrete fine punctation; antero-lateral teeth of pronotum small; mesoscutum mainly coriaceous and anterior half of mesoscutum with large punctures or densely crater-like punctate, medio-posteriorly with coarse reticulation, its lateral lobes more or less sculptured as middle lobe; dorsally hind coxa mainly coriaceous; hind tibial spurs blackish or dark brown; ovipositor sheath 2.4–4.3 times as long as hind tibia and 0.9–1.1 times as long as body. Males have third antennal segment usually rather long and significantly longer than second segment (Fig. 358); paramere black or brown apically (Fig. 361).

#### Distribution.

C. and SE. Europe, Turkey (Schmid-Egger and Saure 2010), new for Iran.

#### Biology.

Unknown. Collected in June-August in Turkey and Iran.

#### Notes.

Close to *Gasteruption erythrostomum* (Dahlbom) but this species has the ovipositor sheath shorter (1.7–2.6 times as long as hind tibia, 0.6–0.8 times metasoma, 1.1–1.6 times hind tibia and tarsus combined versus 2.7–4.3 (rarely 2.4–2.6) times as long as hind tibia, 0.9–1.3 (rarely 0.8) times metasoma and 1.7–2.7 times hind tibia and tarsus combined in *Gasteruption nigrescens*), the mesoscutum of female mainly coriaceous with at most small superficial punctures and rather shiny antero-dorsally (punctures deep, medium-sized and more or less finely crater-like and mixed with fine punctures between punctures, rarely only coarsely punctate) and matt antero-dorsally; of male transversely rugulose or moderately rugose (more coarsely punctate in *Gasteruption nigrescens*), the hind tibia of male black or dark brown ventrally (usually partly yellowish brown ventrally, rarely black), the occipital carina narrower collar-shaped and at most slightly sinuate (wider collar-shaped and often distinctly sinuate) and the mesoscutum hardly setose (rather setose) and the anterior half of pronotal side usually coriaceous ventrally, sometimes partly rugulose (mainly rugulose). *Gasteruption foveiceps* is synonymized despite that the head is more directly narrowed in dorsal view, the area between the antesternal carina and prepectal carina smooth or largely so, the occipital carina less widened, the mesoscutum usually more shiny and densely transversely rugulose or coriaceous-rugulose and its lateral lobes rugulose or rugose. However, this was not enough to separate *Gasteruption foveiceps* as a distinct taxon because intermediates were examined from Turkey.

**Figures 355–361.**
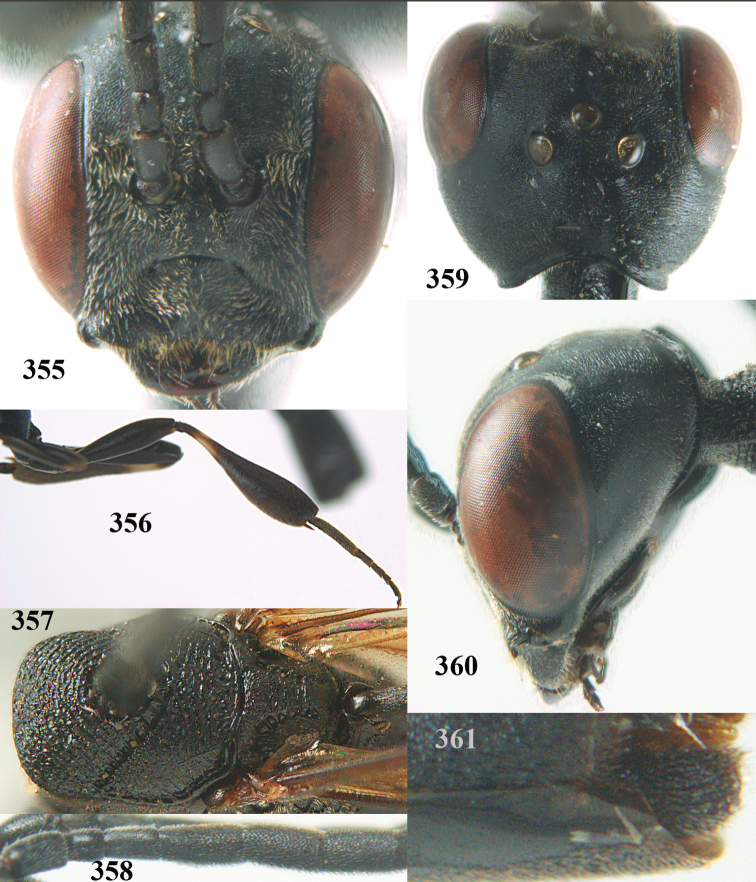
*Gasteruption nigrescens* Schletterer, male, Czech Republic. **355** head anterior **356** hind leg **357** mesonotum dorsal **358** basal antennal segments **359** head dorsal **360** head lateral **361** genitalia lateral.

### Gasteruption opacum

Taxon classificationAnimaliaHymenopteraGasteruptiidae

(Tournier, 1877)

Figs 362 –379

Foenus opacus Tournier, 1877: viii.
Faenus opacus ; Abeille de Perrin 1879: 263, 267, 271.
Foenus opacus var. minor Magretti, 1882: 298; Hedicke 1939: 18.
Gasteruption opacum ; Schletterer 1885: 311, 1889: 387, 396, 424; Dalla Torre 1902: 1069; Szépligeti 1903: 368; Kieffer 1912: 252; Ferrière 1946: 236, 239, 243; Leclercq 1948: 76; Šedivý 1958: 35, 36, 41; Györfi and Bajári 1962: 43, 49; Schmidt 1978: 117; Madl 1987a: 403, 1987b: 24, 1988a: 13, 16, 1988b 406, 1989a 161, 1989b 44, 1990a 129, 1990b 480, 483; Kozlov 1988: 246, 247; Kofler and Madl 1990: 322; Wall 1994: 160; Scaramozzino 1995: 3; Neumayer et al. 1999: 220; Pagliano and Scaramozzino 2000: 13, 17, 31; Saure 2001: 29; Yildirim et al. 2004: 1351; Wisniowski 2004: 118; van der Smissen 2010: 373; van Achterberg 2013: fig. 184.
Foenus vagepunctatus Costa, 1877: xxi; Wall 1994: 149. Synonymized with *Gasteruption opacum* (Tournier) by Ferrière, 1946.
Faenus vagepunctatus ; Abeille de Perrin 1879: 263, 266, 271.
Gasteruption vagepunctatum ; Schletterer 1885: 287, 1889: 382, 386, 394, 396, 422; Dalla Torre 1902: 1075; Szépligeti 1903: 368; Kieffer 1912: 253; Schmiedeknecht 1930: 377, 381; Hedicke 1939: 23 (as synonym of *Gasteruption thomsonii* Schletterer), 26; Ferrière 1946: 243.
Gasteryption vagepunctatum ; Semenov 1892: 209–210.
Gasteruption obscurum ? Schletterer, 1889: 384, 395, 419; Dalla Torre 1902: 1069; Szépligeti 1903: 369; Kieffer 1912: 272; Schmiedeknecht 1930: 381; Hedicke 1939: 18; Ferrière 1946: 243 (as probable synonym of *Gasteruption opacum* (Tournier)); Györfi and Bajári 1962: 43 (as synonym of *Gasteruption opacum* (Tournier)); Wall 1994: 149 (as probable synonym of *Gasteruption opacum* (Tournier)). Synonymized by Györfi and Bajári, 1962.

#### Type material.

Holotype of *Gasteruption opacum* ♀ (MNHG), “[Switzerland], Peney, [near Genève], vii.[18]75”, “Cn Tournier”, “Type”, “*Foenus opacus* Tourn., ♀”.

#### Additional material.

*Iran (Alborz, Arangeh, Shahriar, Shahrestanak, Karaj; Qazvin, Zereshk Road, Koohin, Loshan; Tehran, Peykanshahr (botanical garden); Damavand, 40 km E. Tehran; North Khorasan); **Turkey** (Muradiye; Artvin, Damar, near Murgul; Burdur, 20 km SW of Burdur, 940 m; Gevas, Van Gölü; Istanbul, 12 km SW of Yalova, Termal, 300 m).

#### Diagnosis.

Apex of ovipositor sheath with a distinct white or ivory band, 1.7–2.2 times as long as hind basitarsus (Fig. 369); head flat in front of medium-sized lamelliform occipital carina (Fig. 368; rarely slightly depressed); propleuron elongate, 0.9–1.1 times as long as distance between tegulae and anterior border of mesoscutum and resulting in a slender neck (Figs 363, 371, 377); head distinctly narrowed behind eyes in dorsal view; temple slightly convex in dorsal view (Figs 368, 376); vertex and frons matt and very finely and densely punctate-coriaceous; mesoscutum with separate punctures (Fig. 364); medio-posteriorly mesoscutum coarsely punctate-rugose; antesternal carina wide lamelliform and coxa remain distinctly removed from mesopleuron (Figs 363, 374); hind basitarsus usually largely or completely dark brown or brown, but especially Asian specimens with dorsal ivory patch; ovipositor sheath 1.0–1.2 times as long as body. Males have third antennal segment about half as long as fourth segment (Fig. 373) and paramere narrowly pale apically (Fig. 379).

#### Distribution.

Iran, Turkey, Central and South Europe. New for the fauna of Iran.

#### Biology.

Unknown. Collected in May-October.

**Figures 362–369.**
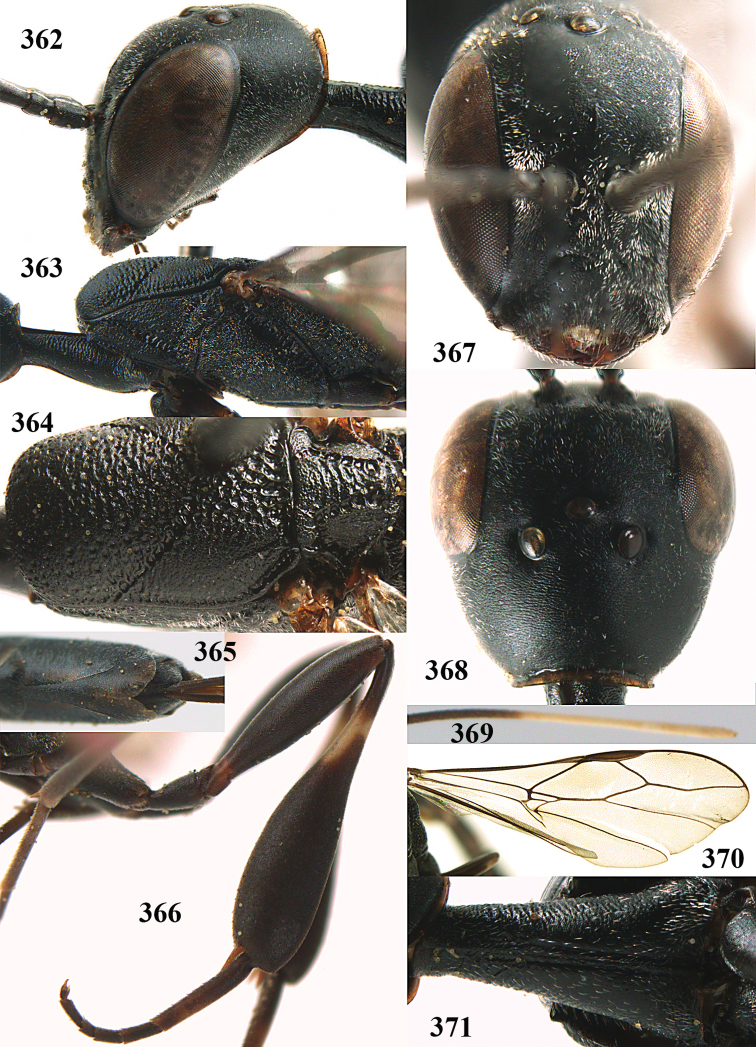
*Gasteruption opacum* (Tournier), female, France. **362** head lateral **363** mesosoma lateral **364** mesonotum dorsal **365** hypopygium ventral **366** hind leg **367** head anterior **368** head dorsal **369** apex of ovipositor sheath **370** fore wing **371** propleuron ventral.

**Figures 372–379.**
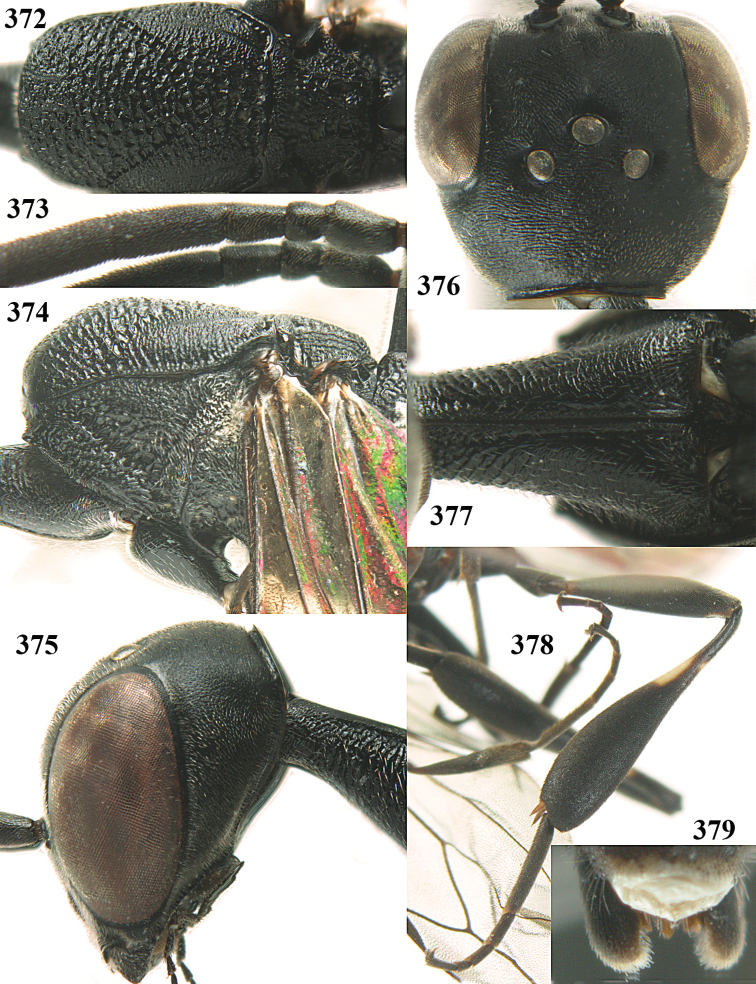
*Gasteruption opacum* (Tournier), male, Hungary. **372** mesonotum dorsal **373** basal antennal segments **374** mesosoma lateral **375** head lateral **376** head dorsal **377** propleuron ventral **378** hind leg **379** genitalia dorsal.

### Gasteruption paglianoi

Taxon classificationAnimaliaHymenopteraGasteruptiidae

van Achterberg & Saure sp. n.

http://zoobank.org/57964B9C-F367-4158-9D19-A1C8BA46573F

Figs 380 –396

#### Type material.

Holotype (RMNH), ♀, “Grecia [= **Greece**], Peloponesso[s], Pirgos, 21.vi.1995, I. Pagliano”. Paratypes (7 ♀ + 6 ♂): 1 ♀ + 2 ♂ (CSC, RMNH), same label data as holotype; 1 ♀ (BZL), “**Turkey** south, 10 km E of Manavgat, 16.iv.1997, Ma. Halada”; 1 ♀ (BZL), “Turkey mer., coast, Side, 70 km E of Antalya, 29.vii–7.viii.2001, P. Tymer”; 1 ♀ + 2 ♂ (BZL, RMNH), “Turkey, Vil. Bursa, near Caglian, 14.vii.1997, M. Riha”; 2 ♀ (BZL, RMNH), “Turkey S., Harbie, Antakya, 17.vi.2000. M. Halada”; 1 ♂ (BZL), “Türkei mer. or, Halfeti env., 3–5.v.1994, Mi. Halada”; 1 ♀ (BZL), “TR – Man[s]isa, 40 km NW of Salihli, 150 m, N38°40', E27°45', 28.vi.2006, J. Halada”; 1 ♂ (CSC), “**Cyprus**, 8 km N [of] Pafos, Mavrokolympos Res., 34.85N 32.40E, 20.vi.2013, Schmid-Egger, cyp-03”.

#### Diagnosis.

**Figures 380–389.**
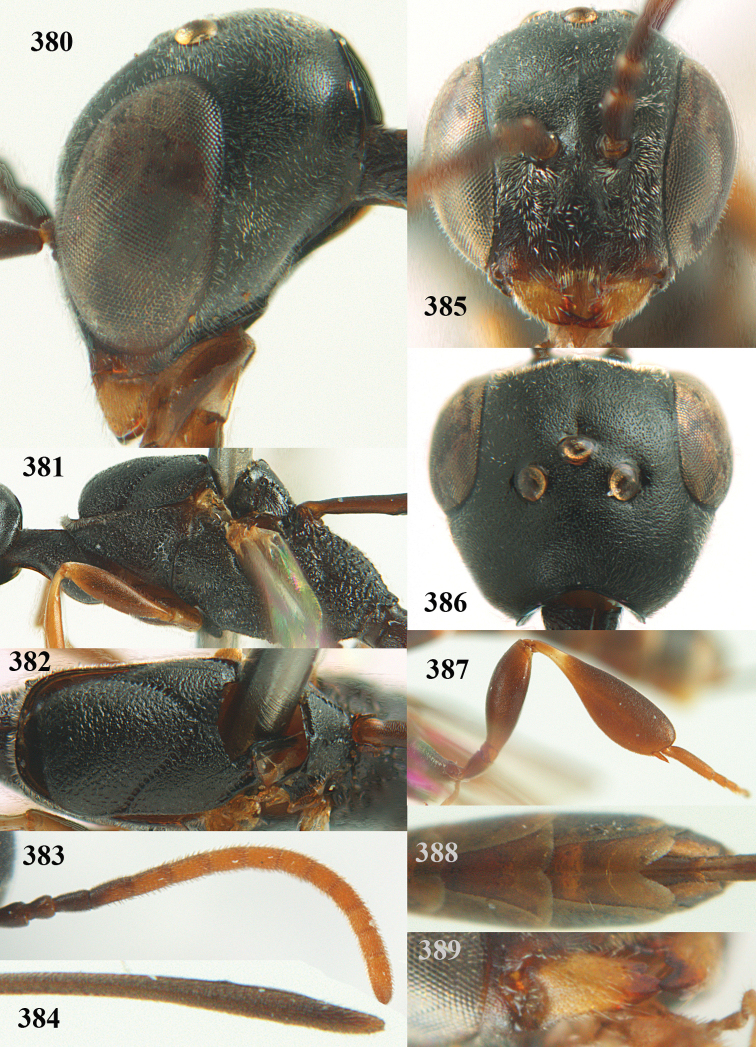
*Gasteruption paglianoi* sp. n., female, holotype. **380** head lateral **381** mesosoma lateral **382** mesonotum dorsal **383** antenna **384** apex of ovipositor sheath **385** head anterior **386** head dorsal **387** hind leg **388** hypopygium ventral **389** mandible lateral.

**Figures 390–396.**
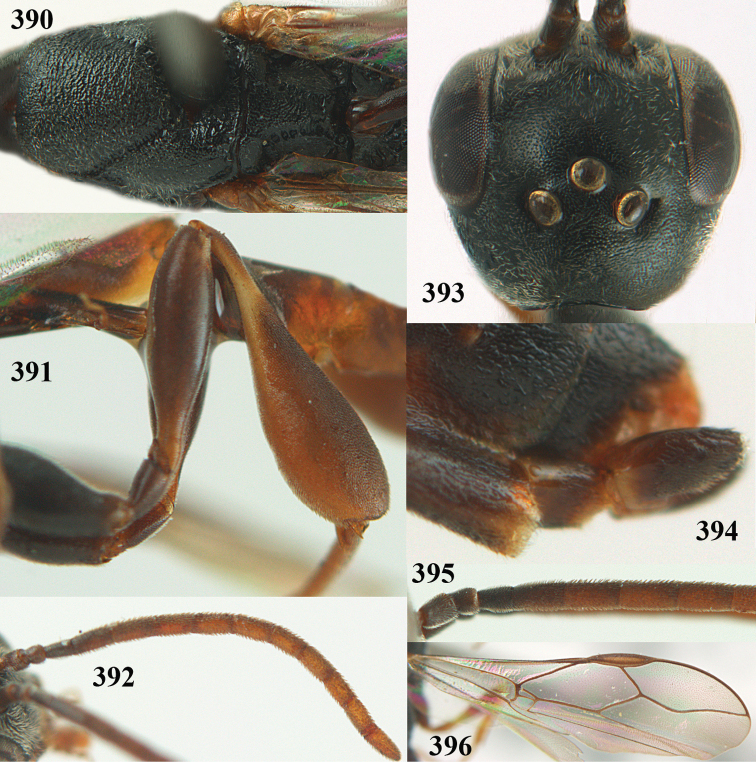
*Gasteruption paglianoi* sp. n., male, paratype, but **396** of ♀ holotype. **390** mesonotum dorsal **391** hind leg **392** antenna **393** head dorsal **394** genitalia lateral **395** basal antennal segments **396** fore wing.

Head weakly convex dorsally, in front of occipital carina without medio-posterior depression; face rather wide (Fig. 385); frons and vertex matt and densely punctulate, of frons less fine than of vertex (Fig. 386); occipital carina narrow, non-lamelliform and dark brown (Fig. 380); mandible pale yellow and basal depression deep; propleuron 0.9 times as long as mesoscutum in front of tegulae; antesternal carina narrow and non-lamelliform; middle lobe of mesoscutum coriaceous and partly slightly rugulose and rather matt, lateral lobe coriaceous and with some fine punctures (Figs 382, 390); scutellum coriaceous; mesosoma indistinctly pilose (Fig. 381); middle lobe rather protruding anteriorly (Fig. 383); hind basitarsus entirely yellowish-brown; hind femur swollen; hind tibia narrow basally (also in dorsal view), stout and with subbasal ivory patch (Fig. 387); ovipositor sheath 1.0–1.1 times as long as hind tibia and entirely dark brown; length of body 6–8 mm; basal four antennal segments of antenna of ♀ dark brown and remainder yellowish brown (Fig. 383), but of ♂ more or less darkened (Figs 392, 394); paramere dark brown apically (Fig. 394).

#### Description.

Female, length of body 7.5 mm (of fore wing 4.0 mm).

*Head*. Head weakly convex dorsally, without medio-posterior depression and subparallel-sided behind eyes; face, frons anteriorly and temples inconspicuously pilose; clypeus distinctly concave ventrally, but without medio-ventral depression; occipital carina narrow and non-lamelliform, dark brown (Fig. 380); third and fourth antennal segments 1.2 and 1.4 times as long as second segment, apical segment 1.8 times as long as penultimate segment; face rather wide (Fig. 385); vertex and frons matt and densely punctulate, of frons less fine than of vertex; ventrally head not enlarged in anterior view, width of malar space 0.3 times length of second antennal segment.

*Mesosoma*. Length of mesosoma 1.8 times its height; propleuron 0.9 times as long as mesoscutum in front of tegulae, stout and coriaceous posteriorly (Fig. 381); laterally pronotum largely coriaceous except for narrow crenulate groove and medio-posteriorly with some coarse punctures, no pilosity; side of pronotum with a small obtuse tooth antero-ventrally; antesternal carina non-lamelliform and narrow; middle lobe of mesoscutum coriaceous and partly slightly rugulose and rather matt, lateral lobe coriaceous and with some fine punctures (Fig. 382); scutellum coriaceous; mesosoma indistinctly pilose (Fig. 381); middle lobe rather narrowly truncate anteriorly (Fig. 382).

*Legs*. Length of hind femur, tibia and basitarsus 2.9, 3.4 and 4.4 times their width, respectively; hind femur rather swollen and trochantellus short; hind tibia narrow basally (also in dorsal view), stout and ventrally curved (Fig. 387); fore coxa close to mesopleuron; hind coxa coriaceous dorsally; hind basitarsus rather stout dorsally and slightly widened basally.

*Metasoma*. Ovipositor sheath 0.2 times as long as body, 0.4 times as long as metasoma and 1.1 times as long as hind tibia; apical emargination of hypopygium shallow; apically ovipositor sheath dark brown.

*Colour.* Black; mandible pale yellow; trochantelli, apices and bases of femora narrowly brownish yellow; bases of fore and middle tibiae and subbasal ring of hind tibia ivory; tegulae, fore and middle tarsi pale brown; antenna (except four basal dark brown segments), hind tibia ventrally, hind tarsus, second-fifth tergites of metasoma apically, metasomal sternites (including hypopygium) and most of palpi yellowish brown; apex of ovipositor sheath dark brown; remainder of legs dark brown; pterostigma brown medially and dark brown laterally; wing membrane subhyaline.

*Male.* Very similar to female; three basal antennal segments blackish or dark brown and remainder of antenna brown but more or less darkened (Figs 392, 395). Third antennal segment 1.3 times as long as second segment, fourth segment 1.6 times third segment and 0.9 times as long as second and third segments combined, fifth segment about as long as fourth segment (Fig. 395); colour and shape of hind leg as of female, but femur slightly less widened (Fig. 391); apex of paramere dark brown (Fig. 394).

*Variation.* Length of body of both sexes 6.2–8.0 mm; ovipositor sheath 1.1–1.2 times as long as hind tibia; hind femur more widened in females from Turkey than in holotype, but less so in males from Turkey (about similar to males of *Gasteruption assectator*); first discal cell of fore wing glabrous and strongly narrowed apically or cell parallel-sided; metasomal sternites (except hypopygium) entirely brownish yellow or largely dark brown; pronotum laterally black or dark brown.

#### Distribution.

Cyprus, Greece, Turkey.

#### Biology.

Unknown. Collected in April-August.

#### Etymology.

Named after its collector and after Dr Guido Pagliano (Turin) who reviewed the Italian Gasteruptiidae.

### Gasteruption phragmiticola

Taxon classificationAnimaliaHymenopteraGasteruptiidae

Saure, 2006

Figs 397 –412

Gasteruption sp. Saure 2001: 30.
Gasteruption phragmiticola Saure, 2006: 126; Westrich 2008: 7–8; van Achterberg 2013: fig. 177.

#### Type material.

Paratypes from Germany examined.

#### Additional material.

*Iran (near Pasargad; near Persepolis); *Turkey (Adiyaman, Gölbasi, 900 m; Denizli, 10 km NE of Denizli, 290 m).

#### Diagnosis.

**Figures 397–406.**
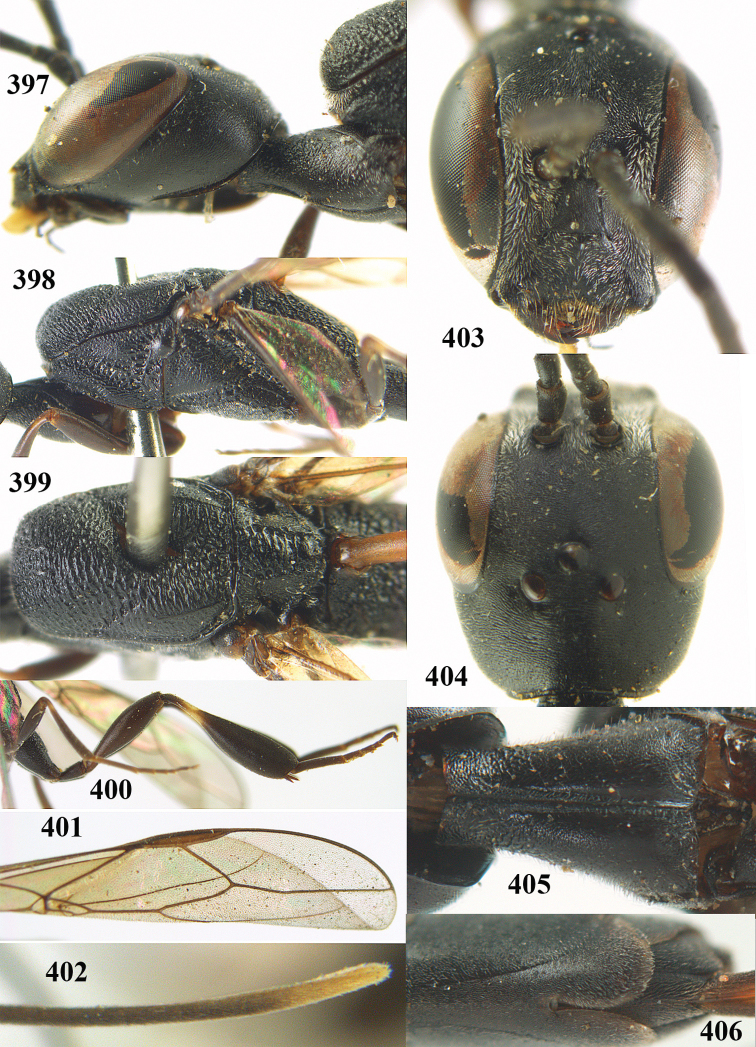
*Gasteruption phragmiticola* Saure, female, paratype. **397** head lateral **398** mesosoma lateral **399** mesonotum dorsal **400** hind leg **401** fore wing **402** apex of ovipositor sheath **403** head anterior **404** head dorsal **405** propleuron ventral **406** hypopygium ventral.

**Figures 407–412.**
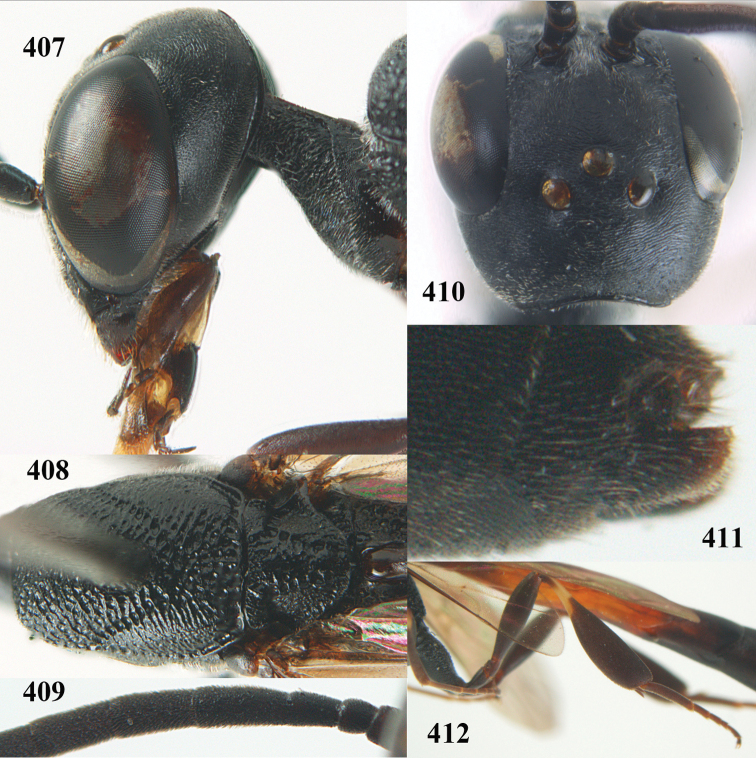
*Gasteruption phragmiticola* Saure, male, paratype. **407** head lateral **408** mesonotum dorsal **409** basal antennal segments **410** head dorsal **411** genitalia lateral **412** hind leg.

Apex of ovipositor sheath more or less pale apically, pale part 0.4–0.9 times as long as hind basitarsus; ovipositor sheath 0.9–1.2 times as long as body and 5.1–6.3 times as long as hind tibia; vertex hardly convex in lateral view, finely aciculate and matt; occipital carina narrow, hardly protruding (Figs 397, 404, 407); head slightly narrowed behind eyes (Figs 404, 410); temple at most moderately elongate (Fig. 407), about 0.8 times as long as eye in dorsal view; malar space short; mandible dark brown or blackish; pronotal side matt and largely sculptured (Fig. 399); mesoscutum coarsely transversely rugose and mixed with large punctures, without smooth and shiny spaces between coarse punctures and laterally black, its lateral lobe with some medium-sized punctures and rugulae; first metasomal tergite reddish-brown; incision of hypopygium deep, slit-like (Fig. 406); fore and middle coxae black. Males have third antennal segment usually rather long, significantly longer than second segment (Fig. 409) and fourth antennal segment about as long as second and third segments combined; paramere dark brown apically (Fig. 411).

#### Distribution.

Europe, *Turkey, *Iran.

#### Biology.

Reared as predator-inquiline from old *Lipara* galls in reed (*Phragmites australis* (Cav.)) with *Hierococcyx pectoralis* Förster nesting inside. Reared between March and August, but collected from late May to early August.

### Gasteruption pseudolaticeps

Taxon classificationAnimaliaHymenopteraGasteruptiidae

van Achterberg sp. n.

http://zoobank.org/AB07AAC0-FD82-4A36-819B-9B2ABDF47D87

Figs 413 –430

Gasteruption foveolum ?; Tirgari 1975: 57 (Tehran).

#### Type material.

Holotype, ♀ (RMNH), 1 ♀, “N. **Iran:** Qazvin: Zereshk Road, MT 6, 22.vi-6.vii.2011, A. Nadimi, RMNH’12”. Paratypes (47 ♀ + 87 ♂; RMNH, TMUT unless otherwise indicated): 1 ♀, same label data as holotype; 3 ♂, id., but 17.viii–4.ix.2011, S. Farahani; 3 ♂, id., but 7–26.vii.2011; 1 ♀ + 2 ♂, id., but MT 3, 22.vi–6.vii.2011, A. Mohammadi; 1 ♂, id., but 7–22.vi.2011; 1 ♂, id., but 26.v-9.vii.2011; 1 ♀ + 2 ♂, id., but MT 5, 5–27.ix.2011; 1 ♀, id., but MT3; 1 ♂, id., but MT 3, 10–25.v.2011, M. Khayrandish; 1 ♀ + 8 ♂, id., but MT 5, 28.vii–18.viii.2011; 3 ♂, id., but MT 5, 7–22.vi.2011; 3 ♂, id., but MT 6, 25.vii-16.viii.2011; 2 ♀ + 1 ♂, “N. Iran: Alborz: Shahriar, MT 25, 11–18.v.2010, M. Khayrandish, RMNH’12”; 1 ♂, id., but 13–20.iv.2010; 2 ♀, id., but 15.v-1.vi.2010; 1 ♀, id., but MT 24, 15–22.vi.2010, A. Nadimi; 1 ♂, id., but 29.vi.-6.vii.2010; 1 ♀, id., but MT 25, 18–25.v.2010; 3 ♂, id., but 1–8.vi.2010; 1 ♂, id., but 4–11.v.2010; 1 ♀, id., but 7–14.ix.2010; 1 ♂, id., but 22–28.ix.2010; 2 ♀, id., but MT 24, 18–25.v.2010, A. Mohammadi; 2 ♂, “N. Iran: Alborz, Sarziarat, Chalous Road, MT 29, 8–15.vi.2010, S. Farahani, RMNH’12”; 2 ♀, “N. Iran: Alborz, Shahrestanak, Chalous Road, MT 29, 15–22.vi.2010, S. Farahani, RMNH’12”; 1 ♀ + 2 ♂, id., but 1–8.vi.2010; 2 ♀ + 4 ♂, “N. Iran: Alborz, Karaj, MT 27, 18–25.v.2010, M. Khayrandish, RMNH’12”; 2 ♂, id., but 11–18.v.2010; 4 ♂, id., but 1–8.vi.2010; 1 ♀, id., but 28.vi-6.vii.2010; 1 ♂, id., but 22–28.vi.2010; 1 ♂, id., but 6–14.vii.2010, A. Nadimi; 1 ♂, id., but 9–16.viii.2010; 1 ♂, “N. Iran: Tehran, Peykanshahr, Bot. Garden, MT 33, 4–13.v.2010, S. Farahani, RMNH’12”; 2 ♀, id., but 18.v-18.vi.2010; 1 ♀ (MZL), “Iran (Teher[an]), Kamalabad, 28.viii.1958, F. Schmid”; 1 ♂ (BZL), Iran, Golestan prov., 70 km E [of] Minudasht, N37°26', E55°99', 1050 m, 12.vi.2010, M. Halada”; 1 ♀ (BZL), “**Kazakhstan** mer., Issik 3 km S, 22–23.vi.1992, K. Denes”; 1 ♀ (BZL), “Kasakhstan ridge Malaysari, 144 km N Alma-Ata, 21.vi.[19]92, Jirousek”; 2 ♀ (BZL), “Kazakhstan, Talas Mt. R., 3 km W Dzhabagly, 42°26'N, 69°58'E, 5.viii.2000, Makogonova”; 1 ♂ (BZL), “Kirg. [= **Kyrgyzstan**], Kirghisky Mt. R., 1700 m, Alamedin riv., viii.2000, V. Gurko”; 1 ♂ (BZL), “Kirg – Ferhgansky Mt. R., Toskool-Ala, *Pistacea* forest, viii.[20]00, Gurko”; 1 ♀ (BZL), id., but 29.vii.2000, 1500 m; 1 ♀ (BZL), “Kirg – Fergan. Mt. R., Alash-Too Mts., Alash forest, viii.[20]00, Gurko”; 2 ♀ + 3 ♂ (BZL, RMNH), “Kirgizia mer.-west, Kizil-kiya, 40,2N 72,1E, 15.v.[19]94, Ma. Halada”; 1 ♂ (BZL), “Kirgisistan, Oshkaya, distrikt Uzgen, Seren-Berge, Tchanget-Pass, zw. Tchanget u. Irisu, ca. 20–25 km N Uzgen, 40°58'N, 73°20'E, 1550 m, 19.vi.1996, H. Rausch”; 1 ♀ + 1 ♂ (BZL), “**Turkey** east, 20 km NW [of] Igdir, 29.vi.1997, Ma. Halada”; 1 ♀ (BZL), “Turkey east, 20 km W [of] Agri, 4.vii.1997, Ma. Halada”; 1 ♀ (RMNH), “Turkey east, 10 km S [of] Ahlat, 24.vi.1997, Ma. Halada”; 6 ♀ + 4 ♂ (BZL, RMNH), “TR, Burdur, 20 km SW [of] Burdur, N37°37', E30°9', 940 m, 7.vii.2006, M. Halada”; 1 ♂ (BZL), “Turkey, Hakkari prov., Akcali, 35 km S [of] Hakkari, N37°71', E44°3', 1700 m, 21.vi.2010, M. Halada”; 1 ♀ (BZL), “TR or., 29.vi.[19]93, Gevas, Van Gölü, K. Deneš”; 1 ♂ (BZL), “TR – Man[s]isa, 40 km NW [of] Salihli, N38°40', E27°45', 150 m, 28.vi.2006, J. Halada”; 1 ♂ (BZL), “TR – Isparta, Egirdir Gölu, 5 km N [of] Akkecili, 920 m, N38°06', E30°46', 10.vii.2006, J. Halada”; 1 ♀ (BZL), “TR – Isparta, 8 km NE [of] Isparta, 1020 m, N37°52', E30°40', 9.vii.2006, M. Kadlecova”; 4 ♂ (CSC), “Türkei, S. Ägäis, Bodrum, Salmakis, *Bupleurum*?, 21.vii.2001, F. Burger”; 1 ♂ (CSEC). “TR, Kayseri, Göreme, 1000 m, NN, 9.vii.[19]88, [C.] Schmid-Egger”; 1 ♀ (RMNH), “Turkey; Nevsehir, 5 km S of Avanos, Zelve, 1000 m, 22.vi.1987, R. Hensen”; 1 ♀ (RMNH), “Turkey; Van, Van, 1800 m, 13.vii.1987, R. Hensen”; 1 ♂ (RMNH), “Turkey; Urfa, Halfeti, 400 m, 18.vi.1987, R. Hensen”; 1 ♀ (BZL), “Turkey, 30 km E [of] Malatya, Kale, 27.viii.2000, M. Halada”; 1 ♀ (BZL), “**Tajikistan**, Varzob riv., VI., Zogar-Varzob, viii.[20]00, V. Gurko”; 1 ♀ (RMNH), “U.S.S.R.: Tadzhikistan, 30 km N Dushambe, n[ea]r Varzab, Kondara, VI.1991, P. Schoorl, RMNH’91”; 1 ♀ (RMNH), “U.S.S.R.: Tadzhikistan, E[ast] of Dushambe, n[ea]r Nurek, Zordolu, 1–2.VII.1991, at light, P. Schoorl, RMNH’91”; 7 ♀ + 21 ♂ (BZL. RMNH), “**Uzbekistan**, Samarkand env., 19–21.v.1994, Ma. Halada”; 1 ♀ (BZL), “Uzbekistan, Hissar Mt. R., foothills n[ea]r Yakkabagh, 30°56'N, 66°53'E, 21.vii.1999, Makogonova”; 3 ♂ (BZL. RMNH), “Uzbekistan or., Aktaš, 41,2'N, 69,4'E, 70 km NO Tachkent, 27.v.[19]94, Ma. Halada”; 1 ♀ + 1 ♂ (BZL), “Uzbekistan or., Yangikichlak, 40,3'N, 66,9'E, 100 km NW Ddjizak, 25.v.[19]94, Ma. Halada”; 1 ♀ + 1 ♂ (BZL), “Uzbekistan or., Czirczik, 41,1'N, 69,1'E, 28.v.[19]94, Ma. Halada”.

**Other material.** 1 ♀ (BZL), “Marocco SE, 45 km N Er Rachidia, Oued Ziz, 14.v.2003, M. Snizek”; 1 ♀ (BZL), “Marocco SW, Taroudant env., 11.v.2003, M. Halada”.

#### Diagnosis.

**Figures 413–422.**
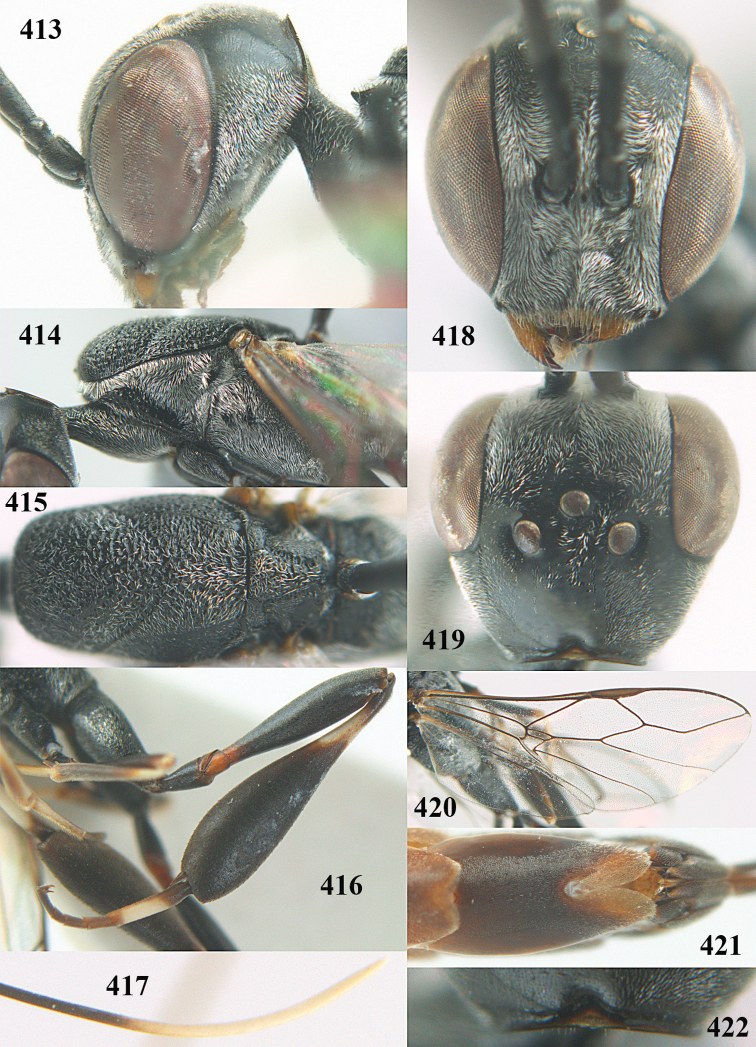
*Gasteruption pseudolaticeps* sp. n., female, holotype. **413** head lateral **414** mesosoma lateral **415** mesonotum dorsal **416** hind leg **417** apex of ovipositor sheath **418** head anterior **419** head dorsal **420** fore wing **421** hypopygium ventral **422** detail of depression of vertex.

Head slightly convex dorsally, in front of occipital carina with a rather shallow medio-posterior depression (shallower in Central Asian specimens than in Iranian and Turkish specimens); face rather narrow (Fig. 418); frons shiny and finely punctulate; occipital carina moderately to widely lamelliform (Fig. 413; narrower in Central Asian specimens than in Iranian and Turkish specimens); vertex shiny and punctulate; mandible pale yellowish brown basally (but dark brown in part of Central Asian specimens); propleuron 0.8 times as long as mesoscutum in front of tegulae; antesternal carina moderately wide lamelliform; middle and lateral lobe of mesoscutum mainly coarsely punctate and with shiny smooth interspaces (Fig. 415); scutellum only punctate antero-laterally; mesosoma conspicuously white pilose laterally (Fig. 414); middle lobe slightly protuberant (Fig. 415); fore coxa distinctly removed from mesopleuron (Fig. 414); hind basitarsus dark brown basally and remainder white; hind tibia rather swollen and with subbasal ivory patch (Fig. 416); ovipositor sheath 1.0–1.1 times as long as body, 1.5 times as long as metasoma, 3.1–3.5 times as long as hind tibia and tarsus combined and 4.1–5.6 times hind tibia; white apical part of ovipositor sheath 1.8–2.5 times as long as hind basitarsus (Fig. 417); length of body 6–13 mm; paramere distinctly ivory apically (Fig. 430).

Close to *Gasteruption laticeps* (Tournier, 1877), but this species has the mesoscutum sparsely setose (rather densely setose in *Gasteruption pseudolaticeps*, but sometimes secondarily lost), the mandible dark brown or reddish brown basally (pale yellowish brown, but darker in part of Central Asian specimens), the vertex densely micro-sculptured and rather dull (superficially punctulate and shiny), the mesopleuron rather sparsely setose (largely densely silvery pilose, but sometimes (?secondarily) sparsely setose), the head in anterior view rather wide (narrower), the antesternal carina curved up and wider medio-ventrally (especially in males less curved up and narrower), the paramere of the male dark brown or blackish apically (ivory apically) and area behind the antesternal carina more or less coarsely transversely striate (often largely smooth except for some rugulae or rugae). Also similar to *Gasteruption diversiceps*, but *Gasteruption pseudolaticeps* has the vertex less sculptured (more sculptured in *Gasteruption diversiceps*), the hind tibia more widened (less widened), the mesosoma conspicuously white pilose laterally (less conspicuous setose) and the vertex weakly depressed medio-posteriorly (absent). Runs in the key by Pasteels (1958) to *Gasteruption belutchistanense* Cameron, 1906, and *Gasteruption sericeipes* Kieffer, 1911, but both differs by having the hind tibia rather slender (wider in *Gasteruption pseudolaticeps*), the antesternal carina narrow (antesternal carina widened), the head of female more elongate in lateral view (less elongate) and the spaces between punctures of the mesoscutum wider than the punctures and coriaceous (narrower and smooth). Also similar to the enigmatic N. African *Gasteruption ocellatum* Benoit, 1984; *Gasteruption ocellatum* has the vertex convex and without medio-posterior depression, the antesternal carina narrow and hardly lamelliform, the lateral lobes of the mesoscutum mainly punctulate and with some separate punctures and hind tibia (except subbasal ivory patch) dark brown.

#### Description.

Female, length of body 12.9 mm (of fore wing 5.8 mm).

*Head*. Head slightly convex dorsally, but in front of occipital carina with a rather shallow medio-posterior depression (Figs 419, 422, 427); face, frons anteriorly and temples densely pilose; occipital carina moderately lamelliform (Fig. 413); third and fourth antennal segments 1.6 and 2.4 times as long as second segment; face rather narrow (Fig. 418); frons shiny and finely punctulate; vertex shiny and punctulate; ventrally head not enlarged in anterior view, malar space 0.3 times length of pedicellus.

*Mesosoma*. Length of mesosoma 2.1 times its height; propleuron 0.8 times as long as mesoscutum in front of tegulae, stout; pronotum laterally coarsely crenulate medially and subposteriorly, coriaceous dorsally and ventrally largely smooth and shiny and with coarse punctures, densely pilose except ventrally; side of pronotum with a distinct tooth antero-ventrally; antesternal carina moderately wide lamelliform and upcurved; middle and lateral lobe of mesoscutum mainly coarsely punctate and with shiny smooth interspaces (Fig. 415), medially with some transverse rugae and medio-posteriorly coarsely reticulate-punctate; scutellum largely smooth and with some fine punctures; mesosoma conspicuously white pilose laterally (Fig. 414); middle lobe slightly protuberant (Fig. 415).

*Legs*. Length of hind femur, tibia and basitarsus 4.8, 4.4 and 5.1 times their width, respectively; hind tibia rather swollen and ventrally curved (Fig. 416); fore coxa distinctly removed from mesopleuron (Fig. 414); hind coxa transversely striate dorsally (except basally) and remainder coriaceous; hind basitarsus moderately slender (Fig. 416).

*Metasoma*. Ovipositor sheath as long as body, 1.5 times as long as metasoma, 3.1 times as long as hind tibia and tarsus combined and 4.8 times hind tibia; white apical part of ovipositor sheath 1.9 times as long as hind basitarsus.

*Colour.* Black; mandible (including base), tegulae, trochantelli, apices and bases of femora narrowly yellowish-brown; bases of fore and middle tibiae and a stripe anteriorly, middle basitarsus (except apically). subbasal ring of hind tibia, hind basitarsus (except basal quarter) and apex of ovipositor sheath white; metasoma brown, but base and apex dark brown; remainder of fore and middle legs, and pterostigma dark brown; wing membrane subhyaline.

*Male.* Very similar to female, but mesoscutum more coarsely reticulate-rugose or densely rugulose than in female. Third antennal segment 1.2–1.4 times as long as second segment, fourth segment twice third segment and 1.2 times as long as second and third segments combined, fifth segment as long as fourth segment (Fig. 424); hind tibia dark brown and with subbasal ivory patch; hind basitarsus entirely dark brown or blackish or with small dorsal ivory patch (Figs 425, 426); apex of paramere ivory (Fig. 430).

*Variation.* Length of body of ♀ 6.5–12.3 mm (of ♂ 6.0–10.0 mm) and of fore wing 2.9–5.8 mm (of ♂ 3.1–4.5 mm); occipital carina narrow to moderately lamelliform, especially in Central Asian specimens reduced; vertex sometimes with some small punctures; mesoscutum of ♀ coarsely and densely punctate and medio-posteriorly rugose or rugulose; scutellum rather densely and coarsely punctate; ovipositor sheath 1.0–1.1 times as long as body, 1.5 times as long as metasoma, 3.1–3.5 times as long as hind tibia and tarsus combined and 4.1–5.6 times hind tibia; white apical part of ovipositor sheath 1.8–2.5 times as long as hind basitarsus; fore and middle basitarsi (except apex) white; hind basitarsus of ♀ with distinct ivory band or largely blackish, with only a small dorsal ivory patch, rarely entirely dark brown or blackish as in ♂. Especially males and both sexes of Central Asian specimens may have the medio-posterior depression of the vertex nearly absent. Central Asian specimens have either dark brown or yellowish mandibles. The females from Morocco are excluded from the type series because they have a shallow medio-posterior depression of vertex, the head more narrowed posteriorly, the vertex and frons with small punctures between dense punctulation and the setae of the pronotal side shorter.

#### Distribution.

Iran, Kazakhstan, Kyrgyzstan, Tajikistan, Turkey, Uzbekistan, ?Morocco. In Northwest Iran the most common species in Malaise traps.

#### Biology.

Unknown. Collected in April-September.

#### Notes.

The record of *Gasteruption foveolum* by Tirgari (1975) may concern the new species, because the latter is more common and very similar. The development of the medio-posterior depression of the vertex and the width of the occipital carina is correlated and clinal. Western populations have the medio-posterior depression distinct and the carina rather wide medio-dorsally, eastern populations have the depression shallow or obsolescent and the carina narrow.

#### Etymology.

Named “*pseudolaticeps*”, because of its similarity of this species with the mainly European *Gasteruption laticeps*.

**Figures 423–430.**
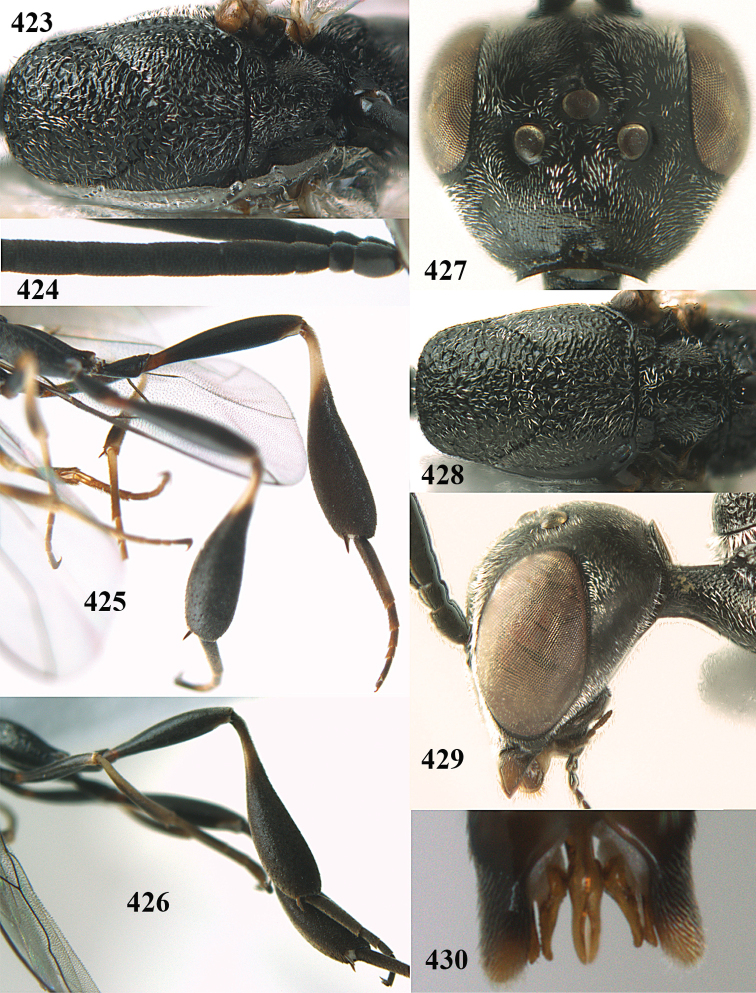
*Gasteruption pseudolaticeps* sp. n., male, paratype, 426 and 428 of small dark male. **423, 428** mesonotum dorsal **424** basal antennal segments **425, 426** hind leg **427** head dorsal **429** head lateral **430** genitalia dorsal.

### Gasteruption punctifrons

Taxon classificationAnimaliaHymenopteraGasteruptiidae

van Achterberg sp. n.

http://zoobank.org/3913777E-A3E6-4ADA-8560-903AB16F3822

Figs 431 –445

#### Type material.

Holotype, ♀ (RMNH), “**Iran**, Tehran-Shahriar, Mal. trap, 15–22.vi.2010, M. Keyrandish, G1, RMNH‘10”. Paratypes (31 ♀ + 44 ♂): 1 ♀ (TMUT), with same label data as holotype; 1 ♀ (RMNH), “N. Iran: Tehran, Shahriar, MT 25, 24–31.viii.2010, A. Nadimi, RMNH’12”; 2 ♂ (RMNH), id., but 25.v.-1.vi.2010; 2 ♂ (RMNH, TMUT), id., but 1–7.ix.2010; 1 ♂ (RMNH), id., but 22–29.vi.2010; 1 ♂ (RMNH), id., but 8–15.vi.2010; 1 ♂ (RMNH), id., but 6–13.vii.2010; 3 ♂ (RMNH, TMUT), id., 1–8.vi.2010, MT 24; 5 ♂ (RMNH, TMUT), id., 15–22.vi.2010; 1 ♀ (RMNH), id., 1–7.ix.2010; 2 ♂ (RMNH), id., but 1–7.ix.2010, G18; 2 ♂ (RMNH, TMUT), id., Karaj, 28.vi.-6.vii.2010, MT 27; 1 ♂ (RMNH), id., but 6–14.vii.2010; 1 ♂ (RMNH), id., but 1–8.vi.2010; 2 ♂ (RMNH), id., but 15–22.vi.2010; 1 ♂ (RMNH), id., but 20–28.vii.2010; 1 ♂ (RMNH), id., but 8–15.vi.2010; 1 ♂ (RMNH), id., but 22–28.vi.2010; 1 ♂ (RMNH), id., but 1–7.ix.2010, MT 26; 24 ♀ + 8 ♂ (BZL, RMNH), “TR [= **Turkey**], Burdur, 20 km SW [of] Burdur, N37°37' E30°9', 940 m, 7.vii.2006, M. Halada”; 5 ♂ (BZL, RMNH), “TR, Burdur, 5 km NE [of] Yesilova, N37°35' E29°55', 1060 m, 6.vii.2006, J. Halada”; 1 ♀ (BZL), “**Syria** west, 50 km S [of] Homs, 24.v.1996, Ma. Halada”; 1 ♀ (MZL), “Syrie, Damas, R[ou]te de Kissoue, 2–18.v.1960, J. de Beaumont”; 3 ♀ + 3 ♂ (CSC), “Türkei, S. Ägäis, Bodrum, Salmakis, [on] *Bupleurum*?, 13, 23 or 25.vii.2001, F. Burger”; 1 ♀ (CSC), “Turkey, 10 km W [of] Alanya, Konakli, 36.58N 31.89E, (shrubland), 1.viii.2009, TR-anti, [C.] Schmid-Egger”; 3 ♂ (RMNH), “Museum Leiden, N.W. **Jordan**, Irbid, 32.33N 35.51E“, “fields near “Eastern Housing”, 23.ix.1981, Ph. Pronk, 81.041”; 1 ♀ + 2 ♂ (CSC), “**Cyprus**, 20 km N of Pafos, Kathikas, 600 m NN, 34.90N 32.42E, 20.vi.2013, Schmid-Egger, cyp-06”; 1 ♂ (CSC), “Cyprus, 20 km N Pafos, Kathikas, 21.vi.2013, C. Saure”.

#### Diagnosis.

**Figures 431–439.**
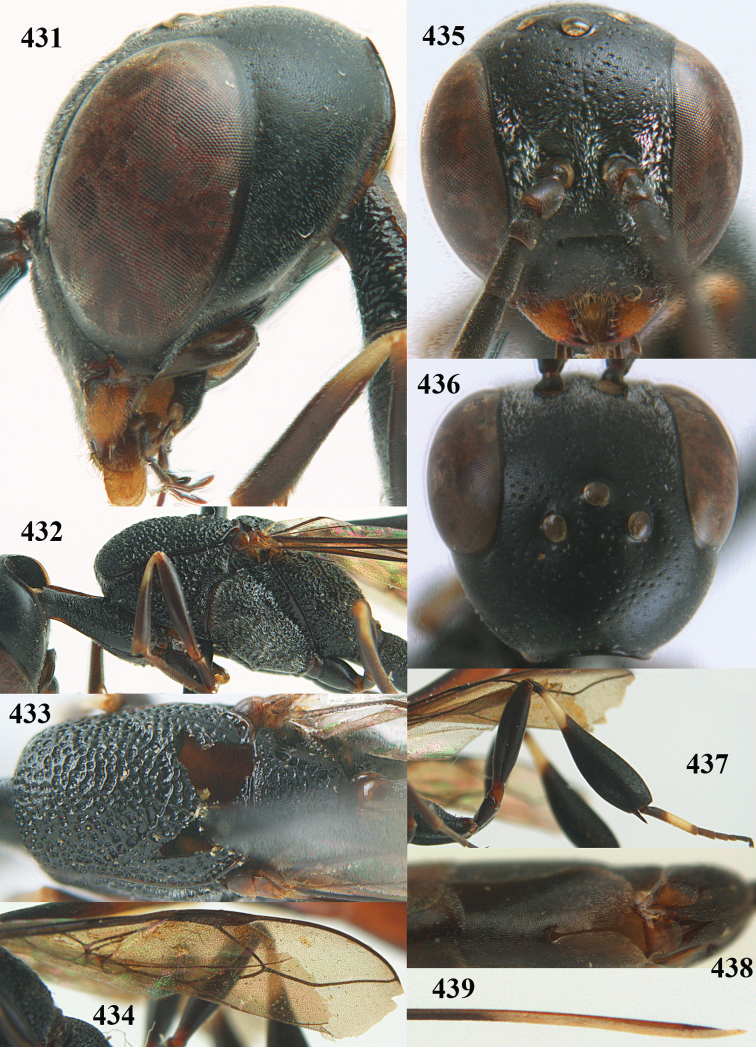
*Gasteruption punctifrons* sp. n., female, holotype. **431** head lateral **432** mesosoma lateral **433** mesonotum dorsal **434** fore wing **435** head anterior **436** head dorsal **437** hind leg **438** hypopygium ventral **439** apex of ovipositor sheath.

Head weakly convex dorsally, in front of occipital carina without medio-posterior depression (Fig. 436); face rather narrow (Fig. 435); frons matt and densely finely punctulate, usually mixed with spaced medium-sized punctures (Figs 435, 443); occipital carina narrowly lamelliform and dark brown (Figs 431, 441); vertex rather matt and densely finely punctulate, often mixed with spaced medium-sized punctures; mandible yellowish brown basally, but partly darkened dorso-basally; propleuron 0.9 times as long as mesoscutum in front of tegulae; antesternal carina narrow and non-lamelliform; middle lobe of mesoscutum coarsely reticulate-punctate, rather matt and with punctulate interspaces, lateral lobe similar but with medial punctulate stripe (Fig. 433); scutellum punctulate anteriorly and remainder transversely rugose; only ventral half of mesopleuron silvery pilose (Fig. 432); middle lobe slightly protuberant (Fig. 433); hind basitarsus dark brown basally, apically brown and remainder white or ivory; hind tibia rather swollen and with subbasal ivory patch (Fig. 437); ovipositor sheath 0.9–1.1 times as long as body, 1.3–1.6 times as long as metasoma, 2.9–3.4 times as long as hind tibia and tarsus combined and 4.8–5.4 times hind tibia; white or ivory apical part of ovipositor sheath 1.8–2.6 times as long as hind basitarsus; length of body 9–14 mm; paramere narrowly pale brown or ivory apically (Fig. 445). Similar to *Gasteruption schlettereri* Magretti, but the new species has the frons punctulate and often with medium-sized punctures (densely and finely rugulose-punctulate and punctures absent in *Gasteruption schlettereri*), the lateral lobe of the mesoscutum partly punctate and coriaceous (reticulate) and the hind basitarsus of the female tricoloured (bicoloured).

#### Description.

Female, length of body 12.5 mm (of fore wing 5.0 mm).

*Head*. Head weakly convex dorsally, without medio-posterior depression; face and frons anteriorly conspicuously silvery pilose; occipital carina narrowly lamelliform, dark brown (Fig. 431); third and fourth antennal segments 1.8 and 2.5 times as long as second segment, apical segment 2.6 times as long as penultimate segment; face moderately narrow (Fig. 435); frons and vertex rather matt and densely finely punctulate, mixed with spaced small punctures; ventrally head not enlarged in anterior view, malar space 0.3 times length of pedicellus.

*Mesosoma*. Length of mesosoma 1.8 times its height; propleuron as long as mesoscutum in front of tegulae, silvery pilose and moderately stout posteriorly; laterally pronotum largely coarsely reticulate, largely without pilosity; side of pronotum with medium-sized acute tooth antero-ventrally; antesternal carina narrow lamelliform; mesoscutum coarsely reticulate-punctate, rather matt and with punctulate interspaces, middle lobe moderately protuberant (Fig. 433), lateral lobe medially coriaceous and with some separate punctures; notauli rather shallow; scutellum punctulate medially and coarsely punctate laterally; mesopleuron and metapleuron silvery pilose (Fig. 432).

*Legs*. Length of hind femur, tibia and basitarsus 4.0, 4.4 and 6.0 times their width, respectively; hind tibia moderately slender and ventrally curved (Fig. 437); fore coxa close to mesopleuron; hind coxa coarsely transversely rugose dorsally; hind basitarsus moderately slender, slightly widened in dorsal view.

*Metasoma*. Ovipositor sheath 0.9 times as long as body, 1.3 times as long as metasoma, 2.9 times as long as hind tibia and tarsus combined and 5.0 times hind tibia; white apical part of ovipositor sheath 2.2 times as long as hind basitarsus.

*Colour.* Black; mesosoma reddish brown; mandible (but dorsally basally darkened), tegulae, coxae, fore femur anteriorly, fore and middle tarsi (except dark brown telotarsi), first tergite, second-fifth tergites laterally, sternites (except dark brown hypopygium) yellowish or orange brown; fore and middle tibiae basally, hind tibia subbasally and hind tarsus medially ivory; base of hind basitarsus dark brown and apically narrowly, as second and third hind tarsal segments, brown; hind tibial spurs blackish, darker than base of hind basitarsus; antenna, palpi, pterostigma, remainder of legs and of metasoma dark brown or black; apex of ovipositor sheath white; wing membrane subhyaline.

*Male.* Very similar to female. Third antennal segment 1.3 times as long as second segment, fourth segment 1.8–1.9 times third segment and 1.0–1.1 times as long as second and third segments combined, fifth segment 0.9–1.0 times as long as fourth segment (Fig. 442); mandible yellowish brown or dark brown; occipital carina non-lamelliform medio-dorsally or very narrowly lamelliform; hind tibia dark brown and with subbasal ivory band; hind tarsus brown, but basitarsus with pale brown or ivory dorsal and lateral patch or complete band, sometimes largely yellowish brown (Cyprus); apex of paramere narrowly pale brown or ivory (Fig. 445).

*Variation.* Length of body of ♀ 9.0–13.7 mm (of ♂ 8.9–13.3 mm); vertex rather matt or with satin sheen; mesosoma and coxae normally black, but sometimes largely reddish brown, or only laterally mainly reddish brown; ovipositor sheath 0.9–1.1 times as long as body, 1.3–1.6 times as long as metasoma, 2.9–3.4 times as long as hind tibia and tarsus combined and 4.8–5.4 times hind tibia; white or ivory apical part of ovipositor sheath 1.8–2.6 times as long as hind basitarsus; female from Syria has mesoscutum and pronotal side partly coriaceous and ivory parts of hind tibia and basitarsus less developed.

#### Distribution.

Cyprus, Iran, Jordan, Syria, Turkey.

#### Biology.

Unknown. Collected in May-September.

#### Etymology.

Named “*punctifrons*”, because of the often distinctly punctate frons.

**Figures 440–445.**
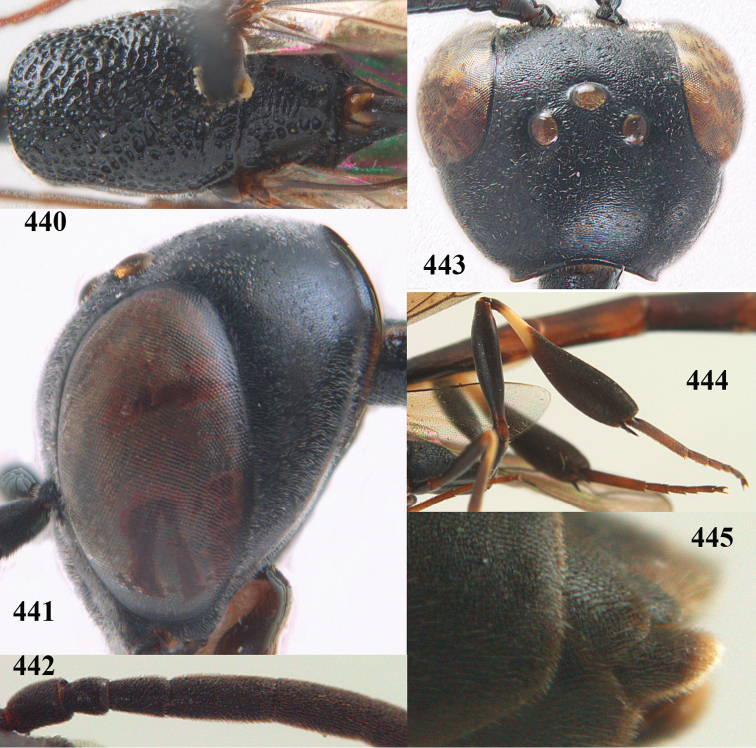
*Gasteruption punctifrons* sp. n., male, paratype. **440** mesonotum dorsal **441** head lateral **442** basal antennal segments **443** head dorsal **444** hind leg **445** genitalia lateral.

### Gasteruption schlettereri

Taxon classificationAnimaliaHymenopteraGasteruptiidae

Magretti, 1890

Figs 446 –461

Gasteruption schlettereri Magretti, 1890: 529; Dalla Torre 1902: 1072; Szépligeti 1903: 369; Kieffer 1904a: 650, 1912: 271; Hedicke 1939: 21; Madl 1990a: 129; Wall 1994: 163.

#### Type material.

Holotype of *Gasteruption schlettereri* ♂ (MCG) “[**Syria**], Dint. Damasco, Febr. Mag. 1889, [legit] Medana”, “Typus”, “*schlettereri* Magr., ♂”, “*Gasteruption schlettereri* Magrt., ♂”, “Holotypus *Gasteruption schlettereri* Magretti, 1890”.

#### Additional material.

*Iran (near Persepolis; near Pasargad; Alborz, Shahrestanak, Chalous Road; id., Shahriar; id., Karaj; Qazvin, Koohin); *Turkey (Hakkari, Habur Deresi Valley, S. Beytishap, 1100 m; id., Akcali, 35 km S of Hakkari, 1700 m; 20 km W of Van; 30 km N of Erdemli Aslanli; 10 km W of Gaziantep; 40 km E of Mut, Cornelek; 60 km E of Mut, Kirobasi; 80 km SW of Malatya Erkenek; 25 km E of Malatya, Kopeksiz; 50 km S of Kars Pasli; Zelve, Mevsehir; near Izmir; 20 km NW of Igdir; Adiyaman, Kahta; Mezikiran Gecidi, 20 km E of Gurun; 40 km N of Muradiye, 2200 m; near Muradiye, 120 km NE of Van, 2000 m; Burdur, 20 km SW of Burdur, 940 m; id., 5 km NE of Yesilova, 1060 m; Muradiye; Van; Göreme; Gevas, Van Gölü; Antalya, Alanya, 50–250 m; Mansisa, 40 km NW of Salihli, 150 m; Anatolia, 10 km S of Kusadasi, W. Davutlar, 0 m; Isparta, 8 km NE of Isparta, 1020 m; id., Egirdir Gölu, 5 km N of Akkecili, 920 m; SW Anatolia, Kusadasi; Hakkari, Mt. Sat, Varegös, SW of Yüksekova, 1700 m; Anatalya, 5 km N of Manavgat, Side, 10 m; Mersin, 30 km N of Silifke, Uzuncaburc, 400 m; Denizli, 20 km NE of Denizli, Pamukkale, 1000 m; Nevsehir, 20 km S of Nevsehir, Kaymakli, 1200 m; Agri, Mt. Ararat, 1800 m; Mardin, Mardin, 1000 m; Van, Mengene Dagi, N of Baskale, 2700–3000 m; Adana, near Feke, 800 m).

#### Diagnosis.

**Figures 446–454.**
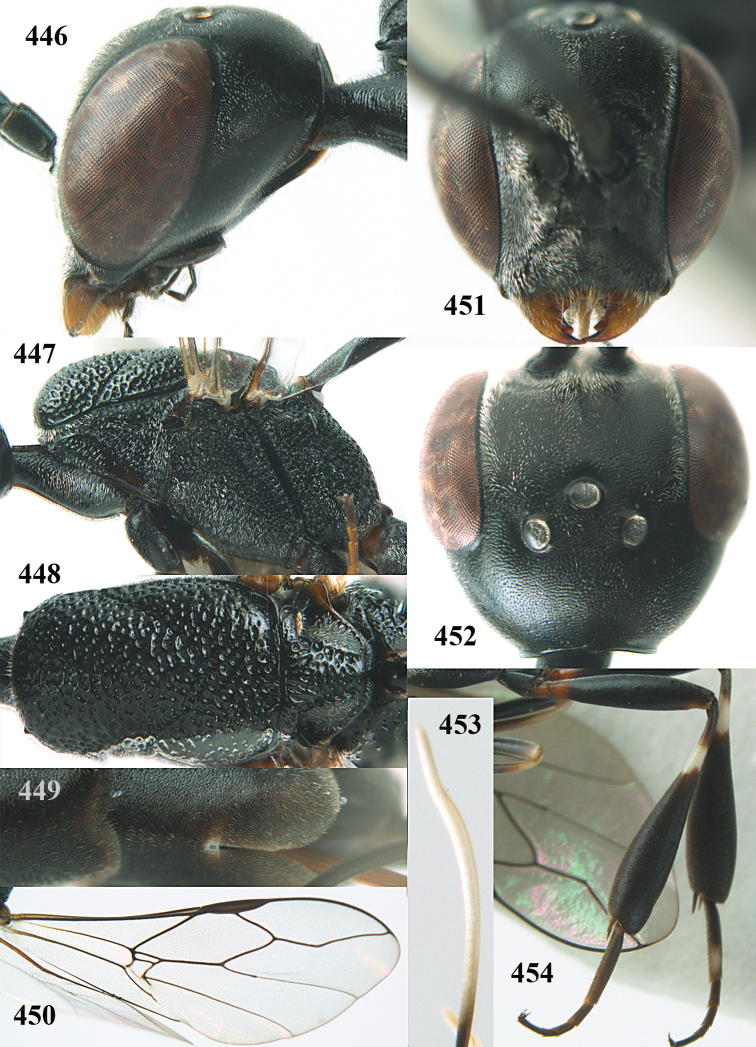
*Gasteruption schlettereri* Magretti, female, Iran. **446** head lateral **447** mesosoma lateral **448** mesonotum dorsal **449** hypopygium ventral **450** fore wing **451** head anterior **452** head dorsal **453** apex of ovipositor sheath **454** hind leg.

**Figures 455–461.**
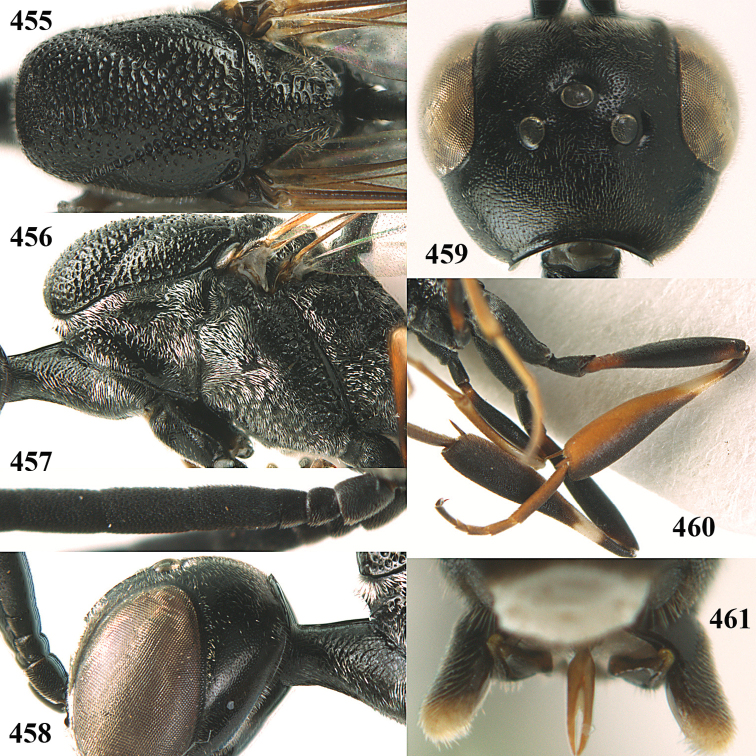
*Gasteruption schlettereri* Magretti, male, Iran. **455** mesonotum dorsal **456** mesosoma lateral **457** basal antennal segments **458** head lateral **459** head dorsal **460** hind leg **461** genitalia dorsal.

Apex of ovipositor sheath with a distinct white or ivory band, 1.7–2.5 times as long as hind basitarsus (Fig. 453); head flat in front of occipital carina, without any depression; antesternal carina of female narrow lamelliform and hardly or not curved up, narrow, somewhat wider than prepectal carina and area between carinae coarsely transversely rugose, but sometimes smooth, of male moderately lamelliform; propleuron 0.8–0.9 times as long as mesoscutum in front of tegulae and rather slender (Fig. 447); occipital carina narrow lamelliform medio-dorsally, distinct (Figs 446, 452, 458); head distinctly narrowed behind eyes in dorsal view (Fig. 452); third antennal segment of female 1.6–1.9 times as long as second segment, fourth segment of female 1.5–1.8 times as long as third segment and subequal or somewhat longer than second and third segments combined; fifth antennal segment of female 1.2–1.5 times as long as third segment and penultimate segments rather short; malar space about 0.2 times as long as second antennal segment (= pedicellus); vertex punctate-coriaceous; antero-lateral teeth of pronotum small; mesoscutum moderately stout (Fig. 448), entirely coarsely reticulate-transversely rugose and shiny, without separate punctures (Figs 448, 455; at most punctate-reticulate); hind coxa finely and densely regularly transversely striate; ovipositor sheath 0.8–0.9 times as long as body, 1.0–1.4 times as long as metasoma and 3.0–4.6 times as long as hind tibia; hind coxa and pronotal side yellowish-brown, dark brown or blackish; mesosoma and coxae usually black but sometimes reddish brown, except for dark brown patch on mesoscutal lobes; hind tibia of female dark brown or black and with ivory subbasal ring; hind basitarsus of female largely ivory (except its dark basal third), but sometimes largely dark brown (Fig. 454); mandible of female orange or yellowish brown basally, in male similar or dark brown; length of body 7–12 mm. Males have fourth antennal segment 3.3 times as long as third segment; shape of third antennal segment similar to second segment, stout (Fig. 457) and about 1.1 times as long as second segment; fourth antennal segment about 1.2 times as long as second and third segments combined; hind tibia of male usually yellowish brown ventrally (Fig. 460); paramere widely ivory apically (Fig. 461).

#### Distribution.

SE Europe, Syria, Iran, Turkey. New for the fauna of Iran and Turkey.

#### Biology.

Unknown. Collected in May-September.

#### Notes.

Females may be confused with *Gasteruption punctifrons*, but *Gasteruption schlettereri* is a more sculptured and shinier species (e.g. mesoscutum and scutellum) with hind tibial spurs and base of hind basitarsus similarly coloured.

### Gasteruption schmideggeri

Taxon classificationAnimaliaHymenopteraGasteruptiidae

van Achterberg & Saure sp. n.

http://zoobank.org/4B44ED42-80B0-4590-85BF-678F2B3FF922

Figs 462 –477

#### Type material.

Holotype, ♀ (RMNH), “TR [= **Turkey**], 54 km W [of] Kayseri, Göreme, 38°39'N, 34°52'E, 17.vii.1998, [C.] Schmid-Egger, TR-nevA”. Paratypes (20 ♀ + 10 ♂): 2 ♀ + 1 ♂ (SEC, RMNH), with same label data as holotype; 2 ♂ (SEC), “TR, Kayseri, Göreme, 1000 m, NN, 9.vii.[19]88, [C.] Schmid-Egger”; 1 ♀ (BZL), “Turkey, 15 km E [of] Refahye, 27.vi.2000, M. Halada”; 7 ♀ + 3 ♂ (BZL, RMNH), “TR, Burdur, 5 km NE [of] Yesilova, N37°35' E29°55', 1060 m, 6.vii.2006, J. Halada”; 2 ♀ (BZL, RMNH), “TR – Isparta, Egirdir Gölu, 5 km N [of] Akkecili, 920 m, N38°06', E30°46', 10.vii.2006, J. Halada”; 1 ♂ (BZL), “Turkey E., 40 km N [of] Muradiye, 2200 m, 5.vii.2000, M. Halada”; 1 ♂ (BZL), “Turkey or., Nemrut Dagi Mt., 50 km NE of Kanta, 2–14.vi.1996, P. Jelinek”; 1 ♂ (BZL), “Turkey mer., Avgadi, 30 km NW of Edemli, 20.vi.1996, P. Jelinek”; 1 ♀ (BZL), “Turkey: Akyaka, 3 m, 37°3'N, 28°20'E, ix.2012, V. Barták”; 2 ♀ (RMNH), “**Greece** – Lesvos, Achladeri, 10 km SE of Kalloni, 29.vi.2001, L. Sijstermans”, “39°9.600'N, 26°17.474'E, altitude 0–25 m”; 4 ♀ (BZL, RMNH), “**Syria** m., Dibbin, 30 km S [of] Suwayda, 15–17.v.1996, Mi. Halada”; 1 ♀ (BZL), “Syria south, Kafr, 10 km SE [of] Suwayda, 19.v.1996, Mi. Halada”; 1 ♀ (BZL), “**Jordan** occ.bor., Aljun env., 32°19'N, 35°43'E, 1.v.2006, F. Kantner”; 1 ♂ (RMNH), “N. **Iran:** Qazvin, Zereshk Road, MT 3, 22.vi.-6.vii.2011, A. Mohammadi, RMNH’12”.

#### Diagnosis.

Head evenly convex dorsally in lateral view, in front of occipital carina without medio-posterior depression; face wide (Fig. 467); frons and vertex with satin sheen and densely very finely punctulate, anteriorly vertex with some additional superficial punctures; occipital carina narrow lamelliform and smooth; propleuron 0.8 times as long as mesoscutum in front of tegulae and large coriaceous and with satin sheen; pronotal side coriaceous dorsally and postero-ventrally, mainly rugulose antero-ventrally and grooves distinctly crenulate; antesternal carina narrow and non-lamelliform; middle lobe of mesoscutum transversely punctate-rugulose and lateral lobe coarsely punctate dorsally and coriaceous with some punctures (Fig. 464); only mesopleuron conspicuously white pilose; hind basitarsus entirely dark brown or partly ivory or pale brown dorsally (as second and sometimes third segment), in dorsal view widened basally; hind tibia moderately slender and with ivory subbasal ring (Fig. 469); fifth sternite of female dark brown; apical half of hypopygium of female incised; ovipositor sheath 0.8–1.0 times as long as body, 1.1–1.4 times as long as metasoma, 3.7–4.6 times as long as hind tibia and 2.3–3.0 times as long as hind tibia and tarsus combined; ovipositor sheath dark brown to pale brown apically, at most pale brown apical part of 0.5 times as long as hind basitarsus; paramere of male black or blackish brown apically (Fig. 477); third antennal segment of male 1.1 times as long as second segment, fourth segment twice as long as third segment and 1.1 times as long as second and third segments combined, fifth segment nearly as long as fourth segment (Fig. 476); mandible brownish or orange yellow basally; hind tibia of male with subbasal ivory ring; paramere dark brown or black apically; length of body 8–13 mm. Close to *Gasteruption smitorum* sp. n., but this species has the hind tibia of the male largely black ventrally and dark brown subbasally (brown or dark brown ventrally and ivory subbasally in *Gasteruption schmideggeri*), the head in dorsal view is more globular (trapezoid), the mesoscutum more shiny (rather matt), the ivory part of the ovipositor sheath longer and the mandible dark brown or black basally (brownish yellow basally).

#### Description.

Female, length of body 9.6 mm (of fore wing 4.4 mm).

**Figures 462–470.**
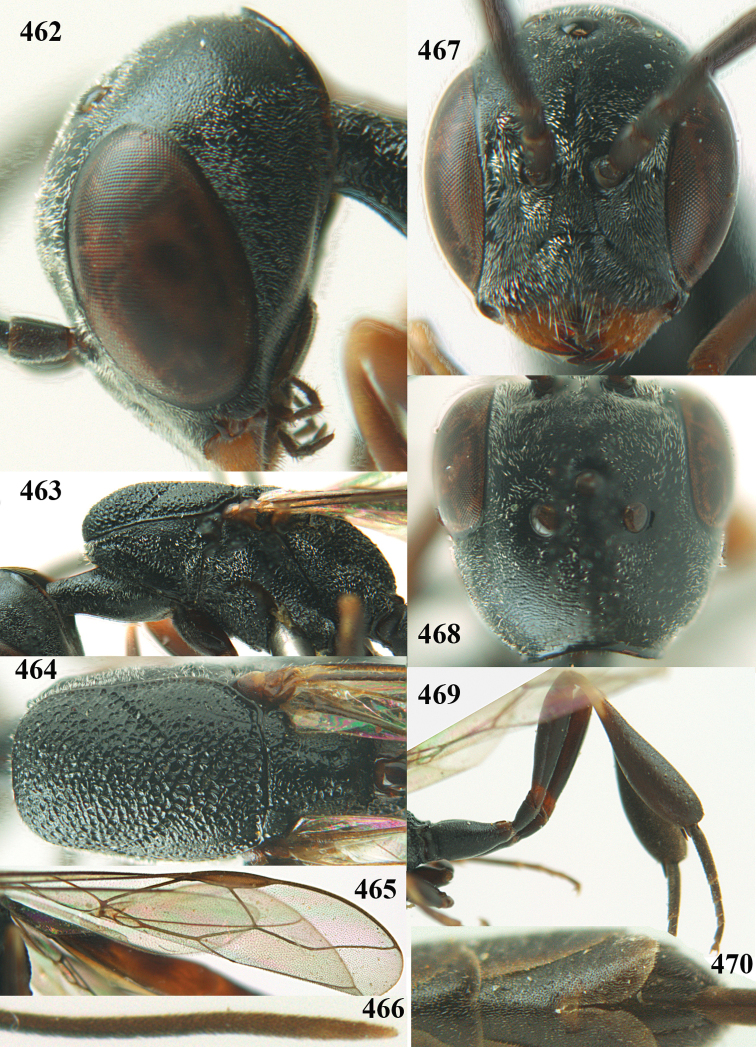
*Gasteruption schmideggeri* sp. n., female, holotype. **462** head lateral **463** mesosoma lateral **464** mesonotum dorsal **465** fore wing **466** apex of ovipositor sheath **467** head anterior **468** head dorsal **469** hind leg **470** hypopygium ventral.

*Head*. Head evenly convex dorsally in lateral view, in front of occipital carina without medio-posterior depression; frons anteriorly and temples inconspicuously pilose; occipital carina narrow lamelliform and smooth (Figs 462, 468); pedicellus rather slender; third and fourth antennal segments 1.4 and 1.9 times as long as second segment; face wide (Fig. 467); frons and vertex with satin sheen and superficially finely punctulate, vertex with some superficial punctures between dense punctulation (Fig. 468); stemmaticum partly finely punctate; temples subparallel-sided behind eyes and head rather trapezoid in dorsal view (Fig. 468); ventrally head not enlarged in anterior view, malar space 0.1 times length of pedicellus; inner tooth of mandible medium-sized.

*Mesosoma*. Length of mesosoma twice its height; propleuron 0.8 times as long as mesoscutum in front of tegulae, coriaceous, stout and with satin sheen; pronotal side coriaceous dorsally and postero-ventrally, mainly rugulose antero-ventrally and grooves distinctly crenulate; pronotum with medium-sized tooth antero-ventrally; antesternal carina narrow and non-lamelliform; middle lobe of mesoscutum transversely punctate-rugulose and lateral lobe coarsely punctate dorsally and coriaceous with some punctures (Fig. 464); scutellum superficially transversely rugulose; only mesopleuron conspicuously white pilose (Fig. 463); propodeum with nearly complete median carina.

*Legs*. Length of hind femur, tibia and basitarsus 4.3, 4.5 and 5.2 times their width, respectively; hind tibia moderately slender (Fig. 469); fore coxa close to mesopleuron; hind coxa mainly transversely rugulose; hind basitarsus moderately slender (Fig. 469), but basally widened in dorsal view.

*Metasoma*. Ovipositor sheath 0.8 times as long as body, 1.1 times as long as metasoma, 3.7 times as long as hind tibia and 2.3 times as long as hind tibia and tarsus combined; brown apical part of ovipositor sheath 0.5 times as long as hind basitarsus.

*Colour.* Black; mandible brownish yellow basally; tegulae brown; bases of fore and middle tibiae and subbasal ring of hind tibia ivory; remainder of fore and middle legs (except dark brown coxae) brown; remainder of hind leg, pterostigma, metasoma basally and most of its apical half dark brown; second and third metasomal segments orange brown; wing membrane subhyaline.

*Male*. Very similar to female, but head shorter in dorsal view and vertex, mesoscutum and scutellum more coarsely sculptured. Third antennal segment 1.4 times as long as second segment, fourth segment twice as long as third segment and 0.8 times as long as second and third segments combined, fifth segment 0.9 times as long as fourth segment (Fig. 476); hind tibia and basitarsus as in female; apex of paramere blackish brown (Fig. 477).

*Variation.* Length of body of ♀ 7.7–13.0 mm (of ♂ 8.4–10.7 mm); antero-lateral tooth of pronotum minute to medium-sized; mesoscutum of ♀ more or less coarsely and densely crater-like punctate and medio-posteriorly rugose or rugulose; scutellum rather densely and coarsely punctate; hind tibial spurs dark brown as base of hind basitarsus, but sometimes paler; hind basitarsus entirely dark brown or partly ivory or pale brown dorsally (as second and sometimes third segment); ovipositor sheath 0.8–1.0 times as long as body, 1.1–1.4 times as long as metasoma, 3.7–4.6 times as long as hind tibia and 2.3–3.0 times as long as hind tibia and tarsus combined; pale brown apical part of ovipositor sheath 0.1–0.5 times as long as hind basitarsus or sheath apically dark brown; pronotal side sometimes mainly coriaceous antero-ventrally; pale setae of hind tibia inconspicuous; wing membrane subhyaline or moderately infuscate.

#### Distribution.

Greece, Jordan, Syria, Turkey.

#### Biology.

Unknown. Collected in May-July, September.

#### Etymology.

Named after the collector of the holotype, Dr Christian Schmid-Egger (Berlin) for his contribution to enlarge and popularise our knowledge of Hymenoptera.

#### Notes.

Two males were identified by M. Madl in 1996 as *Gasteruption hastator*, but males of the new species differs by having the clypeus hardly impressed, the hind tibia ivory subbasally, the hind basitarsus slenderer and dark brown or brown, the hind tibia slenderer and the mesoscutum rugose instead of reticulate.

**Figures 471–477.**
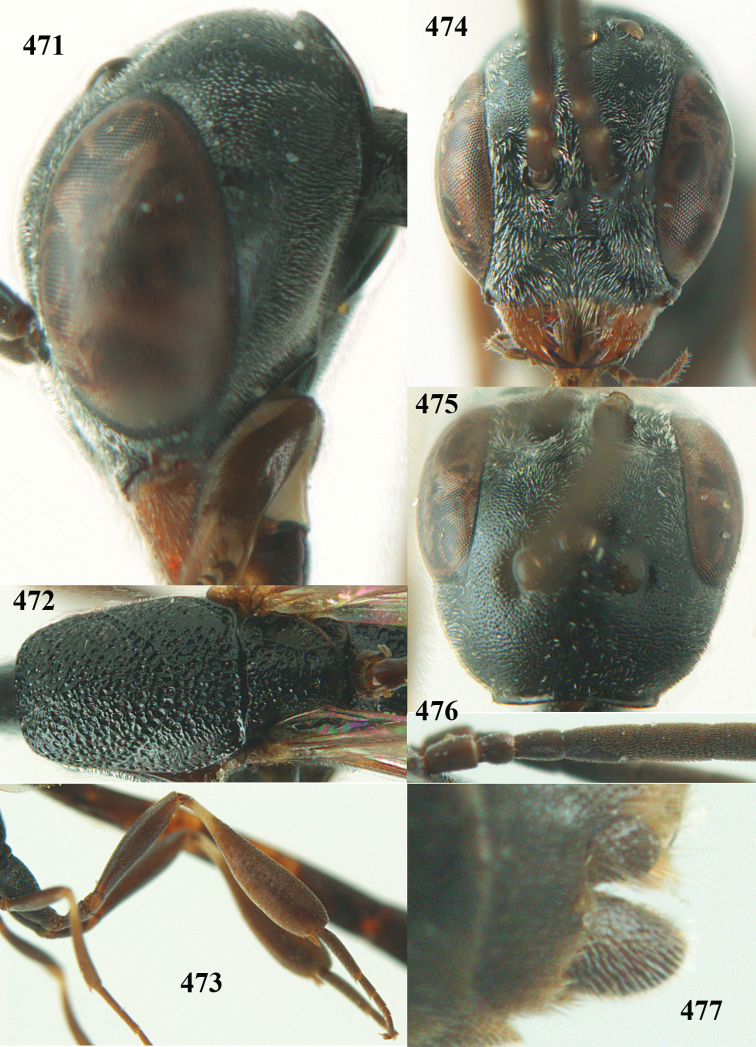
*Gasteruption schmideggeri* sp. n., male, paratype. **471** head lateral **472** mesonotum dorsal **473** hind leg **474** head anterior **475** head dorsal **476** basal antennal segments **477** genitalia lateral.

### Gasteruption scorteum

Taxon classificationAnimaliaHymenopteraGasteruptiidae

van Achterberg sp. n.

http://zoobank.org/C7F35A27-DF24-45FD-A053-C45CE89912E1

Figs 478 –492

#### Type material.

Holotype, ♀ (BZL), “**Turkey**, 15 km W [of] Refahye, W of Erzibcan, 1600 m, 7.vii.2000, M. Halada”. Paratypes (18 ♀ + 6 ♂): 12 ♀ + 5 ♂ (BZL, RMNH), same label data as holotype; 1 ♀ (BZL), “TR., Konya, 30 km S [of] Aksehir, 24.vi.[19]98, J. Halada”; 4 ♀ + 1 ♂ (BZL, RMNH), “Turkey mer., Avgadi, 30 km NW of Erdemli, 1300 m, 20.vi.1996, P. Jelinek”; 1 ♀ (BZL), “NW **Jordan**, Irbid reg., Sarham vill., 25.iv.2003, I. Pljushtch”.

#### Diagnosis.

**Figures 478–486.**
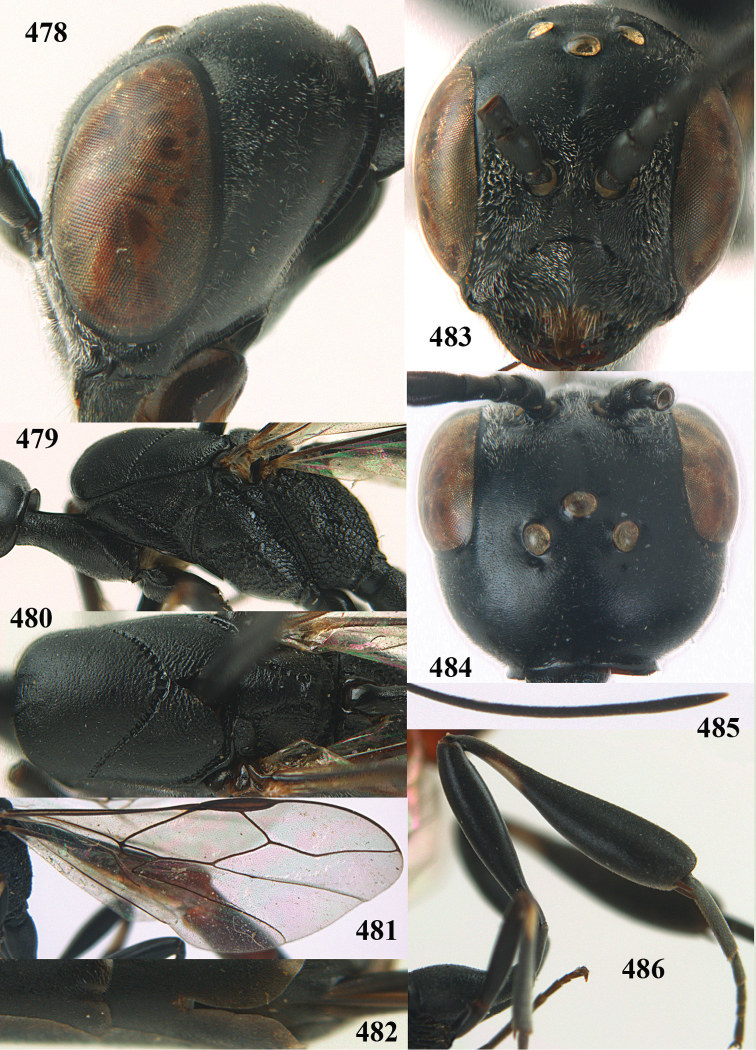
*Gasteruption scorteum* sp. n., female, holotype. **478** head lateral **479** mesosoma lateral **480** mesonotum dorsal **481** fore wing **482** hypopygium ventral **483** head anterior **484** head dorsal **485** apex of ovipositor sheath **486** hind leg.

Head distinctly convex dorsally in lateral view, in front of occipital carina without medio-posterior depression; face rather wide (Fig. 483); frons and vertex with satin sheen and densely very finely punctulate; occipital carina wide, smooth and lamelliform (Figs 478, 484, 492); propleuron 0.8 times as long as mesoscutum in front of tegulae and large coriaceous and with satin sheen; pronotal side very finely coriaceous but groove crenulate-rugose; antesternal carina narrow and non-lamelliform; middle and lateral lobe of mesoscutum very finely coriaceous and with satin sheen (Fig. 480); only mesopleuron ventrally conspicuously white pilose (Fig. 479); hind basitarsus entirely dark brown; hind tibia rather swollen and ventrally with ivory subbasal patch (Fig. 486); fifth sternite of female dark brown; apical 0.6–0.7 of hypopygium of female incised; ovipositor sheath 0.9–1.0 times as long as body, 1.4–1.5 times as long as metasoma, 4.3–5.0 times as long as hind tibia and 2.6–3.6 times as long as hind tibia and tarsus combined; ovipositor sheath dark brown apically, at most pale brown apical part of sheath 0.2 times as long as hind basitarsus; paramere of male black apically; third antennal segment of male 1.1 times as long as second segment, fourth segment twice as long as third segment and 1.1 times as long as second and third segments combined, fifth segment nearly as long as fourth segment (Fig. 490); hind tibia of male with subbasal ivory patch ventrally (Fig. 488); paramere black apically (Fig. 489); length of body 11–14 mm.

#### Description.

Female, length of body 14.2 mm (of fore wing 7.0 mm).

*Head*. Head distinctly convex dorsally in lateral view, in front of occipital carina without medio-posterior depression; face, frons anteriorly and temples inconspicuously pilose; occipital carina wide, smooth and lamelliform (Fig. 478); third and fourth antennal segments 1.4 and 2.3 times as long as second segment; face rather wide (Fig. 483); frons and vertex with satin sheen and superficially very finely punctulate; temples directly roundly narrowed behind eyes and resulting in subglobular head in dorsal view (Fig. 484); ventrally head not enlarged in anterior view, malar space 0.3 times length of pedicellus; inner tooth of mandible medium-sized.

*Mesosoma*. Length of mesosoma 1.9 times its height; propleuron 0.8 times as long as mesoscutum in front of tegulae, coriaceous, stout and with satin sheen; pronotal side mainly coriaceous except for crenulate-rugose grooves, sparsely pilose except posteriorly; side of pronotum with a rather small tooth antero-ventrally; antesternal carina narrow and non-lamelliform; middle and lateral lobe of mesoscutum very finely coriaceous and with satin sheen (Fig. 480); scutellum coriaceous; only mesopleuron ventrally white pilose (Fig. 479); propodeum with nearly complete median carina.

*Legs*. Length of hind femur, tibia and basitarsus 4.5, 4.6 and 6.4 times their width, respectively; hind tibia rather swollen and ventrally curved (Fig. 486); hind coxa mainly coriaceous; hind basitarsus moderately slender (Fig. 486).

*Metasoma*. Ovipositor sheath nearly as long as body, 1.4 times as long as metasoma, 4.7 times as long as hind tibia and 3.6 times as long as hind tibia and tarsus combined; pale brown apical part of ovipositor sheath 0.2 times as long as hind basitarsus.

*Colour.* Black; mandible (including base) and tegulae dark brown; bases of fore and middle tibiae and ventral subbasal patch of hind tibia ivory; apex of second and third tergite largely orange brown; remainder of legs and metasoma dark brown or blackish brown; pterostigma and veins dark brown; wing membrane subhyaline.

*Male.* Very similar to female, but mesoscutum superficially rugulose medio-posteriorly. Third antennal segment 1.1 times as long as second segment, fourth segment twice as long as third segment and 1.1 times as long as second and third segments combined, fifth segment nearly as long as fourth segment (Fig. 490); hind tibia and basitarsus as in female; apex of paramere black (Fig. 492).

*Variation.* Length of body of ♀ 11.4–14.2 mm (of ♂ 12.8 mm); mesoscutum of ♀ coarsely and densely punctate and medio-posteriorly rugose or rugulose; scutellum rather densely and coarsely punctate; ovipositor sheath 0.9–1.0 times as long as body, 1.4–1.5 times as long as metasoma, 4.3–5.0 times as long as hind tibia and 2.6–3.6 times as long as hind tibia and tarsus combined; pale brown apical part of ovipositor sheath 0.1–0.2 times as long as hind basitarsus or dark brown apically.

#### Distribution.

Jordan, Turkey.

#### Biology.

Unknown. Collected in April, June-July.

#### Etymology.

Named after “scorteus”, (Latin for “leathern”) and is used because of the very fine coriaceous mesoscutum.

**Figures 487–492.**
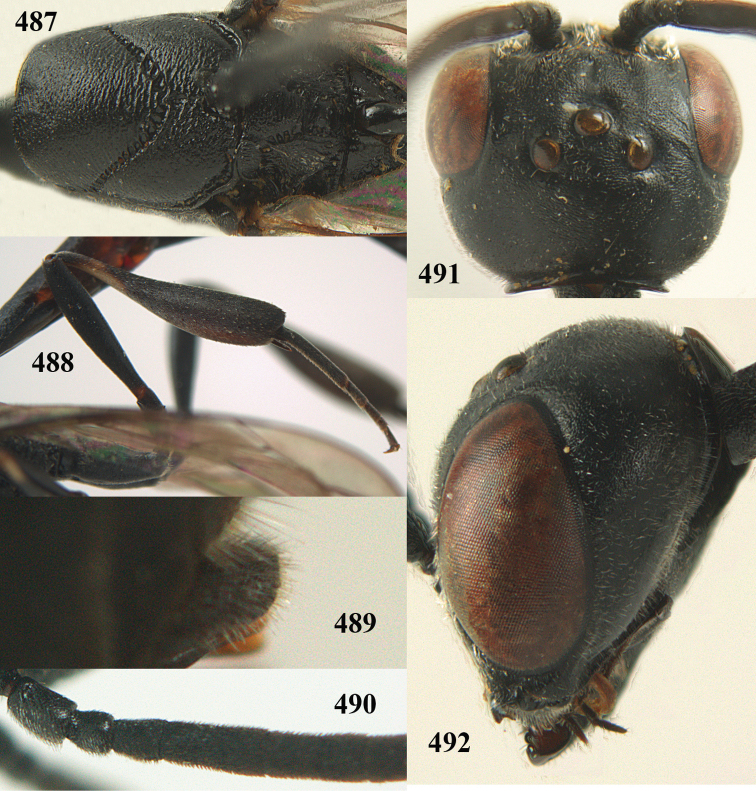
*Gasteruption scorteum* sp. n., male, paratype. **487** mesonotum dorsal **488** hind leg **489** genitalia lateral **490** basal antennal segments **491** head dorsal **492** head lateral.

### Gasteruption smitorum

Taxon classificationAnimaliaHymenopteraGasteruptiidae

van Achterberg sp. n.

http://zoobank.org/3C38DC94-0433-46B3-B59F-DAF8EF645B67

Figs 493 –506

#### Type material.

Holotype, ♀ (RMNH), “**Turkey**; (Van), Van, 1800 m, 13.vii.1987, R. Hensen”. Paratypes (2 ♀ + 5 ♂): 1 ♂ (BZL), “Turkey, 15 km E Malatya, 27.vi.2000, M. Halada”; 2 ♂ (BZL, RMNH), “Turkey E., Muradiye, 3.vii.2000, M. Halada”; 1 ♀ + 1 ♂ (BZL), “Turkey mer. or., Halfeti env., 3–5.v.1994, Mi. Halada”; 1 ♂ (RMNH), “Turkey; (Agri), 30 km W [of] Eliskirt, 2200 m, 14.vii.1987, R. Hensen”; 1 ♀ (CSC), “TR-Kayseri, Göreme, 1000 m NN, 9.vii.[19]88, Schmid-Egger”.

#### Diagnosis.

**Figures 493–501.**
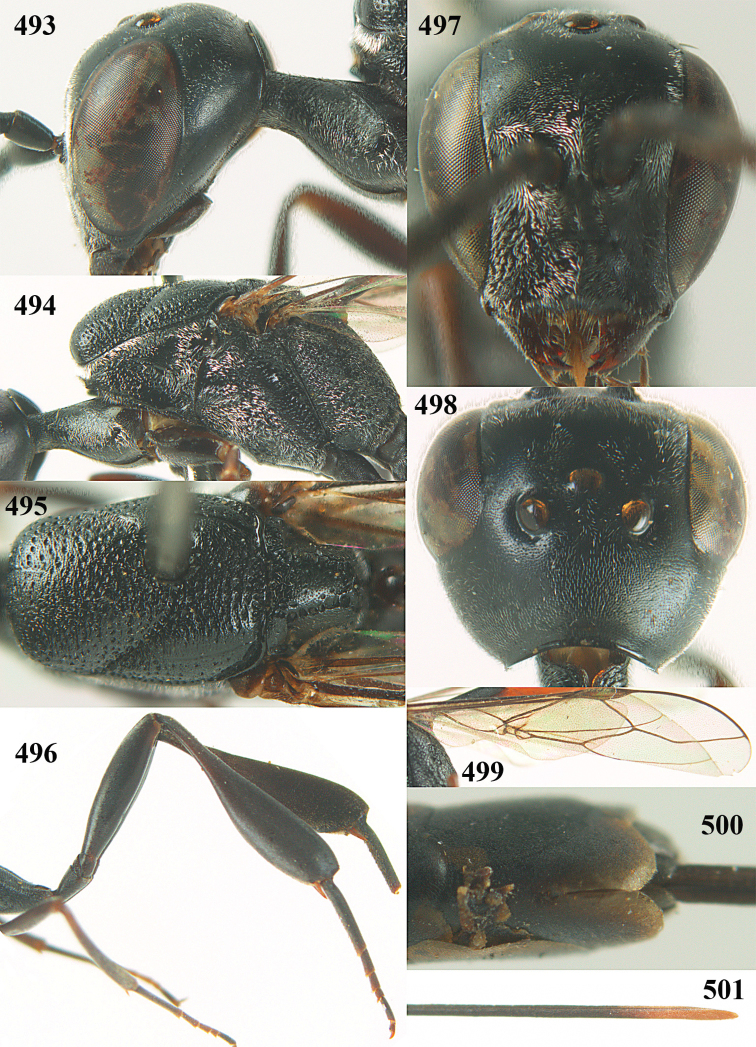
*Gasteruption smitorum* sp. n., female, holotype. **493** head lateral **494** mesosoma lateral **495** mesonotum dorsal **496** hind leg **497** head anterior **498** head dorsal **499** fore wing **500** hypopygium ventral **501** apex of ovipositor sheath.

Head slightly convex dorsally, in front of occipital carina without medio-posterior depression; face moderately wide (Fig. 497); frons and vertex with satin sheen and densely and finely punctulate (Figs 497, 498); occipital carina narrowly lamelliform and dark brown (Fig. 493); vertex with some obsolescent punctures between punctulation (Fig. 498); mandible dark brown basally; propleuron stout, coriaceous and 0.8 times as long as mesoscutum in front of tegulae; antesternal carina narrow and non-lamelliform; mesoscutum coarsely spaced punctate, with punctulate interspaces and with distinct satin sheen, medio-posteriorly densely coarsely punctate (Fig. 495); scutellum punctulate, with some punctures and with satin sheen; mesosoma laterally (except pronotal side medially and ventrally) silvery pilose (Fig. 494); hind basitarsus blackish brown; hind tibia slender and dark brown subbasally (Fig. 496); ovipositor sheath 0.9 times as long as body, 1.5 times as long as metasoma, 2.5 times as long as hind tibia and tarsus combined and 4.2 times hind tibia; ivory apical part of ovipositor sheath 1.0–1.4 times as long as hind basitarsus; length of body 10–13 mm. Very similar to Central European *Gasteruption hungaricum*, but *Gasteruption smitorum* lacks the steep medio-posterior part of the vertex, the mesoscutum is somewhat less shiny and the hind femur and tibia are slenderer. Also similar to the East Palaearctic *Gasteruption sinarum* Kieffer, 1911, but differs as follows: the head less narrowed in dorsal view (more trapezoid in *Gasteruption sinarum*), the mesoscutum with separate punctures medio-posteriorly (reticulate-punctate), the hind femur black (dark brown) and the lateral lobe of the mesoscutum distinctly convex in lateral view (rather flat). Differs from the North and Central European and East Palaearctic *Gasteruption subtile* (Thomson, 1883) by the rather shiny vertex and mesoscutum (matt in *Gasteruption subtile*), the mesoscutum with separate punctures medio-posteriorly (reticulate-punctate), the pronotum largely rugulose antero-ventrally and largely smooth (except some punctures) postero-ventrally (mainly densely coriaceous) and the hind tibia and basitarsus black or dark brown (partly white or ivory).

#### Description.

Female, length of body 11.2 mm (of fore wing 5.5 mm).

*Head*. Head slightly convex dorsally, in front of occipital carina without medio-posterior depression; face and frons anteriorly conspicuously silvery pilose; occipital carina narrowly lamelliform, dark brown (Fig. 493); third and fourth antennal segments 2.0 and 2.4 times as long as second segment, apical segment twice as long as penultimate segment; face moderately wide (Fig. 497); frons and vertex with satin sheen and densely and finely punctulate (Fig. 498); ventrally head not enlarged in anterior view, malar space 0.2 times length of pedicellus.

*Mesosoma*. Length of mesosoma 1.7 times its height; propleuron stout and 0.8 times as long as mesoscutum in front of tegulae, with satin sheen and coriaceous; laterally pronotum largely rugulose antero-ventrally and largely smooth (except some punctures) postero-ventrally; side of pronotum with medium-sized obtuse tooth antero-ventrally; antesternal carina narrow and non-lamelliform; mesoscutum spaced coarsely punctate, with punctulate interspaces and with satin sheen, medio-posteriorly densely coarsely punctate (Fig. 495); notauli narrow and moderately impressed; scutellum punctulate, with some punctures and with satin sheen; mesosoma laterally (except largely pronotal side) silvery pilose (Fig. 494).

*Legs*. Length of hind femur, tibia and basitarsus 4.1, 4.6 and 5.2 times their width, respectively; hind femur and tibia rather slender (Fig. 496); hind coxa mainly transversely rugulose dorsally; hind basitarsus moderately slender and 1.1 times as long as remainder of tarsus and basally widened in dorsal view.

*Metasoma*. Ovipositor sheath 0.9 times as long as body, 1.5 times as long as metasoma, 2.5 times as long as hind tibia and tarsus combined and 4.2 times hind tibia; ivory apical part of ovipositor sheath 1.2 times as long as hind basitarsus.

*Colour.* Black; mandible, antenna mainly, tegulae, palpi, pterostigma, fore and middle legs largely (except blackish coxae) and metasoma (including hypopygium, but second-fourth tergites largely yellowish brown), dark brown; hind tibial spurs yellowish brown and distinctly paler than base of hind basitarsus; hind basitarsus blackish brown; apex of ovipositor sheath yellowish ivory; wing membrane subhyaline.

**Figures 502–506.**
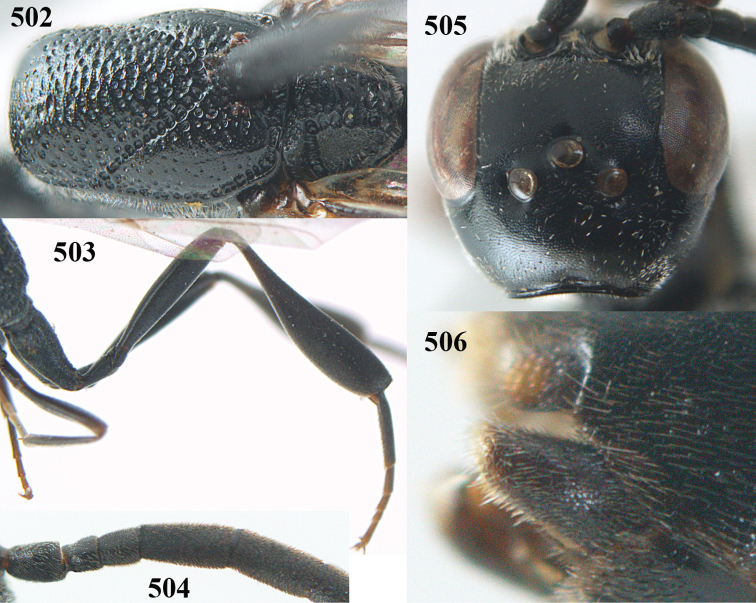
*Gasteruption smitorum* sp. n., male, paratype. **502** mesonotum dorsal **503** hind leg **504** basal antennal segments **505** head dorsal **506** genitalia lateral.

*Male.* Very similar to female, but mesoscutum coarser punctate than in female (Fig. 502). Third antennal segment 1.2–1.3 times as long as second segment, fourth segment 2.1–2.2 times as long as third segment and 1.2 times as long as second and third segments combined, fifth segment slightly shorter than fourth segment (Fig. 504); hind tibia white, ivory or dark brown subbasally; hind basitarsus entirely dark brown; apex of paramere black or narrowly pale brown (Fig. 506).

*Variation.* Length of ovipositor sheath 4.2–4.3 times as long as hind tibia, its ivory apex 1.2–1.3 times as long as hind basitarsus; length of body 10.3–11.9 (female) or 11.0–13.3 (male) mm; hind tibia dark brown or narrowly ivory basally in both sexes.

#### Distribution.

Turkey.

#### Biology.

Unknown. Collected in May-July.

#### Etymology.

Named in honour of the hymenopterist Jan Smit (Duiven) for his contribution to our knowledge of Dutch Hymenoptera and of his son (and dipterist) John Smit (Leiden) for his contribution to our knowledge of European Diptera and for collecting Hymenoptera in South America.

### Gasteruption syriacum

Taxon classificationAnimaliaHymenopteraGasteruptiidae

Szépligeti, 1903

Figs 507 –521

Gasteruption syriacum Szépligeti, 1903: 369, 372; Kieffer 1904a: 648, 1912: 269; Hedicke 1939: 21.
Gasteruption libanense Pic, 1916: 23. **Syn. n.**

#### Type material.

Lectotype of *Gasteruption syriacum* here designated, ♂ (MTMA), “Syria / teste Papp, J., 1986”, “463/4”, “Lectotypus ♂ *Gasteruption syriacum* Szépl., 1903/ desi. J. Papp, 1980”, “preparatum 8”. Holotype of *Gasteruption libanense* ♂ (MNHN) from Lebanon (Mt. Liban), “Museum Paris EY 0000003921”, “3. voumoma [?]”, “Type”, “Museum Paris, coll. M. Pic”, “n. sp. près *dolichoderum*, tête rebordée et mois longue der[ièrre] les yeux”, “*Gasteruption libanense* Pic”, “vu [illegible] special”, “Monotypus, des. Madl, 1987”, “*Gasteruption schlettereri* Magr., ♂, det. Madl 1987”.

#### Additional material.

*Turkey (Baykan, Tuzlagozu; Cornelek, 40 km E of Mut; Pasli, 50 km S of Kars; Eregli; Eskisehir, Sakri ilica near Gumele; Ürgüp, 30 km E of Nevsehir, about 1400 m; Erkenek, 80 km SW of Malatya; 40 km NE of Muradiye, 2200 m; Konya, 30 km S of Aksehir; Adiyaman, Nemrut Dagi Mts., Keradut; SE of Elazig, Hazar Gölü; near Halfeti; Mansisa, 30 km SEE of Salihli, 430 m; id., 15 km SEE of Salihli, 170 m; Govas, Van Gölü; Artvin, Damar, near Murgul; Acigöl, near Cerdak; Denizli, 10 km NE of Denizli, 290 m; 20 km NW of Igdir; Burdur, 20 km SW of Burdur, 940 m; id., 5 km NE of Yesilova, 1060 m; Isparta, 8 km NE of Isparta, 1020 m; id., Karakus Dagi centre, 1460 m; Sultan Daglari, near Yalvac; S. Ägäis, Bodrum, Salmakis, on ?*Bupleurum*; Bursa, Bursa, 300 m; Marmaris; Hakkari, Mt. Sat, SW of Yüksekova, Varegös, 1650 m; id., Hakkari, 1750 m; id., Beytüsebab, 1400 m; Mügla, Köycegiz; Bodrum, Salmakis).

#### Diagnosis.

**Figures 507–514.**
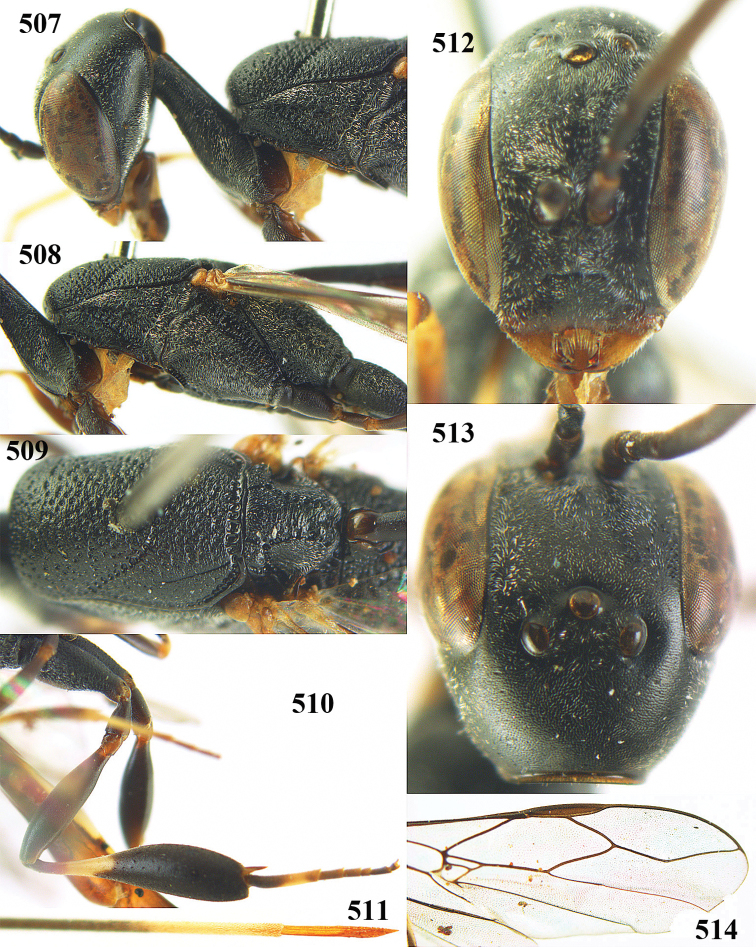
*Gasteruption syriacum* Szépligeti, female, Turkey. **507** head lateral **508** mesosoma lateral **509** mesonotum dorsal **510** hind leg **511** apex of ovipositor and part of sheath **512** head anterior **513** head dorsal **514** fore wing.

Apex of ovipositor sheath with a distinct white or ivory band, 2–3 times as long as hind basitarsus; head flat in front of occipital carina, without any depression; antesternal carina narrow and hardly lamelliform (Fig. 508); temple nearly straight in dorsal view, directly narrowed behind eye (Fig. 513); vertex and frons rather shiny, at least with satin sheen and very finely and densely punctulate (Fig. 513); propleuron elongate, 0.9–1.1 times as long as distance between tegulae and anterior border of mesoscutum and resulting in a long neck (Fig. 507); mesoscutum with separate punctures; medio-posteriorly mesoscutum with distinct punctures between coriaceous sculpture in European specimens, but denser punctate (Fig. 509) or more or less rugulose in Asian specimens; hind basitarsus often with ivory band, but sometimes absent; mandible and ventral part of clypeus orange-brown. Males have third antennal segment 1.3–1.5 times as long as second segment and fourth antennal segment 2–3 times as long as third segment and 1.3 times as long as second and third segments combined (Fig. 520).

#### Distribution.

SE Europe, Lebanon, Syria, Turkey. New for the fauna of Turkey.

#### Biology.

Unknown. Collected in July-September.

#### Notes.

The holotype of *Gasteruption libanense* belongs not to *Gasteruption schlettereri*, because *Gasteruption libanense* has a more slender and longer propleuron (about as long as mesoscutum up to tegulum; rather stout and 0.9 times in *Gasteruption schlettereri*), hind tibia dark brown ventrally (yellowish), lateral lobe of mesoscutum mainly coriaceous and laterally with rugulae (coarsely rugose) and middle lobe of mesoscutum with some punctures among rugulosity (coarsely rugose).

**Figures 515–521.**
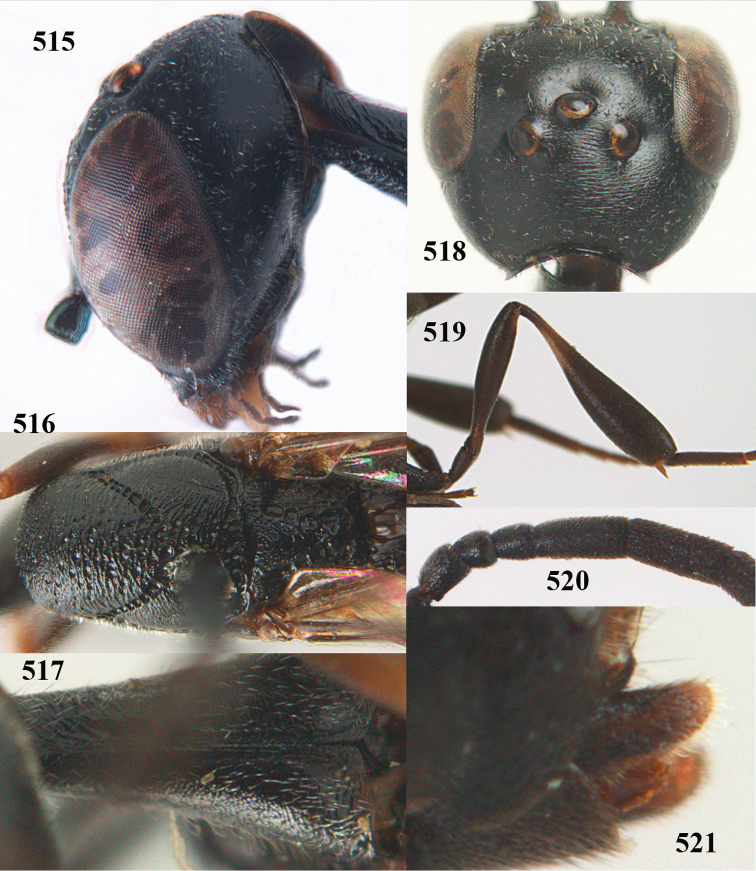
*Gasteruption syriacum* Szépligeti, male, Syria. **515** head lateral **516** mesonotum dorsal **517** propleuron ventral **518** head dorsal **519** hind leg **520** basal antennal segments **521** genitalia lateral.

### Gasteruption tournieri

Taxon classificationAnimaliaHymenopteraGasteruptiidae

Schletterer, 1885

Figs 522 –534

Foenus jaculator ; Tournier 1877: viii.
Faenus jaculator ; Abeille de Perrin 1879: 263, 265, 270.
Gasteruption tournieri Schletterer, 1885: 287, 1889: 382, 388, 394, 395, 415; Dalla Torre 1902: 1075; Szépligeti 1903: 368; Kieffer 1912: 247; Schmiedeknecht 1930: 376, 380; Hedicke 1939: 25; Ferrière 1946: 237, 238, 246; Crosskey 1951: 292; Šedivý 1958: 35, 36, 42; Györfi and Bajári 1962: 45, 49; Schmidt 1969: 295; Dolfuss 1982: 24; Oehlke 1984: 168, 171, 181; Madl 1987a: 404, 1987b: 24, 1988c 39, 1989a 161, 1989b 45, 1990a 129, 1990b 480, 483; Kozlov 1988: 246, 247; Kofler and Madl 1990: 322; Wall 1994: 164; Scaramozzino 1995: 3; Neumayer et al. 1999: 220; Pagliano and Scaramozzino 2000: 15, 17, 33; Saure 2001: 29; Yildirim et al. 2004: 1351; Wisniowski 2004: 118; Turrisi 2004: 84; van der Smissen 2010: 373; Zhao et al. 2012: 103–108; Samin and Bagriacik 2012: 387; van Achterberg 2013: 83.
Gasteryption tournieri ; Semenov 1892: 206.
Gasteruption austriacum Schletterer, 1885: 277; Hedicke 1939: 26; Wall 1994: 148. Synonymized with *Gasteruption tournieri* Schletterer by Schletterer, 1889.
Gasteruption nitidum Schletterer, 1885: 281; Hedicke 1939: 26; Wall 1994: 149. Synonymized with *Gasteruption tournieri* Schletterer by Schletterer, 1889.

#### Type material.

On p. 324 Schletterer refers under “*Gasteruption Tournieri* Schlett. ♀", to both sexes of “*Foenus jaculator*”, of Tournier (1877) but also indicated that the specimens are in the Vienna museum (“Mus. caes. Vindob.”). The Tournier specimens from Switzerland, France, and Italy mentioned in the original description may be included in the type series (as was done in Zhao et al. (2012)), but after reconsideration the indication by Schletterer (1885) should be accepted. The ♀ (NMW) labeled “*Gasteruption tournieri* Schlett., type”, “*tournieri* det. Schlett.”, “Typus”, belongs to *Gasteruption jaculator* and is disagreeing with the original description. There are two females from 1885 (collected by Handlirsch on 12.viii.1885 in “Tyrol Bozen”, one female collected by Schletterer “b. Bozen”, 1885). The paper with the original publication of G. *tournieri* is submitted (“Vorgelegt”) on 4.ii.1885 and was published end of August 1885. Both specimens collected in summer 1885 cannot be syntypes and, therefore, the female syntypes of *Gasteruption tournieri* (at least two females without locality mentioned in the original description) are not present in NMW and are probably lost. The following specimen (identified by the author after the original publication) is here designated as neotype: ♀ (NMW), “[Italy:] St. Pauls (Tirol), Schlett., 1887”, “*Tournieri*, Typ., det. Schletter.”, “Typus”. The type series of *Gasteruption austriacum* (male holotype in NMW from Austria, Frankenfeld and collected by Erber) and of *Gasteruption nitidum* (syntype males in NMW from Italy, Calabria, collected by Erber) could not be traced and are probably lost.

#### Additional material.

**Turkey** (Biga, Cinardere; Cornelek, 40 km E of Mut; Konya, 30 km S of Aksehir; Bursa, near Caglian; Bursa, Ulu Dagi Mts., near Barakli; 15 km W of Refahye, W of Erzincan, 1600 m; Eskisehir, Sakri ilica near Gümele; Sultan Daglari, near Yalvac; Hakkari, Akcali, 35 km S of Hakkari; id., 25 km E of Gözeldere, 930 m; Antalya, east of dunes; Acigöl, near Sardak; Mersin, Kuzucubelen; Seydisehir, Teke Gec.; Burdur, 20 km SW of Burdur, 940 m; id., 5 km NE of Yesilova, 1060 m; Mansisa, 35 km SEE of Salihli, 900 m; id., 30 km SEE of Salihli, 430 m; id., 40 km NW of Salihli, 150 m; Denizli, 10 km NE of Denizli, 290 m; Maras, Göksun, 1400 m; Hakkari, Mt. Sat, SW of Yüksekova, Varegös, 1650 m; Bodrum, Salmakis, on ? *Bupleurum*; Burdur, 20 km N of Aglasun, Koruglubeli, 950 m; Adana, 8–15 km N of Adana, 50 m; Gaziantep, 43 km WNW of Kilis, Gözkaya, 600 m).

#### Diagnosis.

**Figures 522–529.**
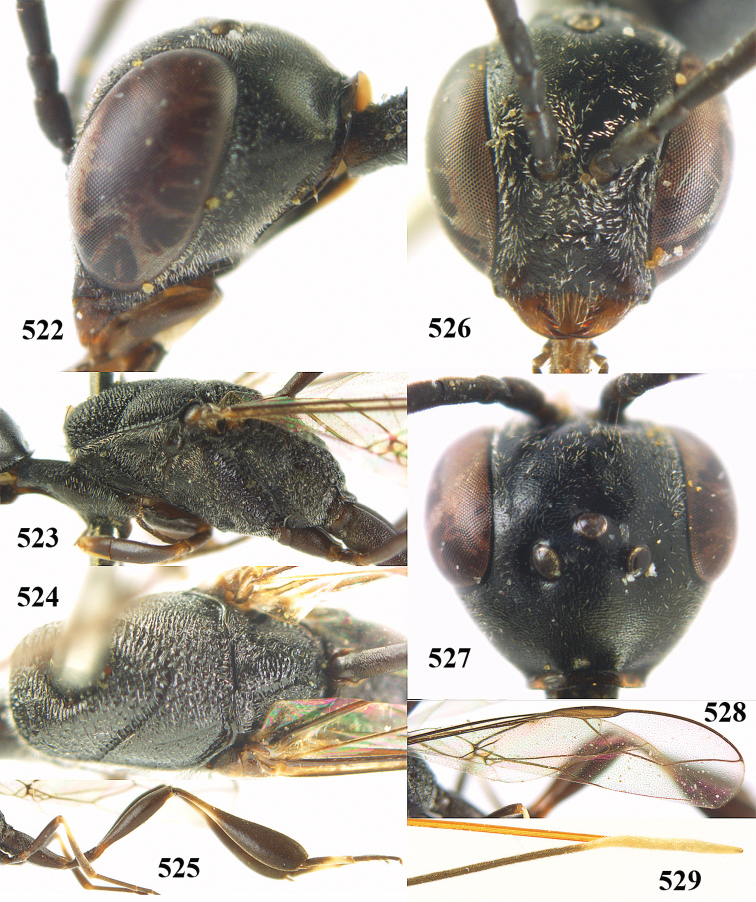
*Gasteruption tournieri* Schletterer, female, Netherlands. **522** head lateral **523** mesosoma lateral **524** mesonotum dorsal **525** hind leg **526** head anterior **527** head dorsal **528** fore wing **529** apex of ovipositor sheath.

Apex of ovipositor sheath with a distinct white or ivory band, 1.7–3.0 times as long as hind basitarsus; head with shallow middle depression in front of occipital carina (Fig. 527), but rather frequently reduced and rarely absent; occipital carina distinctly lamelliform and medium-sized to wide, more or less concave medio-dorsally (Figs 522, 527, 533); antesternal carina narrow and non-lamelliform or nearly so, not or slightly elevated above mesosternum (Fig. 523); fourth and fifth antennal segments of female 1.5–2.1 and 1.3–2.0 times as long as third segment, respectively; frons sparsely punctulate and with distinct interspaces or very finely and densely punctulate; vertex more or less finely punctulate and distinctly shiny (Fig. 527); face moderately wide (Fig. 526); temples linearly narrowed behind eyes (Fig. 527); propleuron slightly longer than mesoscutum up to tegulae (Fig. 523); lateral lobes of mesoscutum rugulose-coriaceous; anterior half of mesoscutum moderately punctate-rugose (Fig. 524); subbasally outer side of hind tibia and apical half of hind basitarsus ivory (Fig. 525); fore and middle legs to variable extend white, ivory and brown; body slender (Fig. 523). Males have third antennal segment about as long as second segment, fourth and fifth segments 3 and 2.5 times as long as third segment, respectively; fifth antennal segment distinctly wider than third segment (Fig. 532).

#### Distribution.

South and Central Europe, Turkey, Iran. *Gasteruption tournieri* was recently reported from north-western Iran (East Azerbaijan, Arasbaran, 746 m) by Samin and Bagriacik (2012).

#### Biology.

Unknown. Collected from early June till late September.

#### Notes.

One female in NMW identified as *Gasteruption tournieri* by Schletterer and labelled as “Typus”, belongs to *Gasteruption jaculator* (Linnaeus). Males have rather frequently no depression in front of the occipital carina and the head is more or less densely finely aciculate.

**Figures 530–535.**
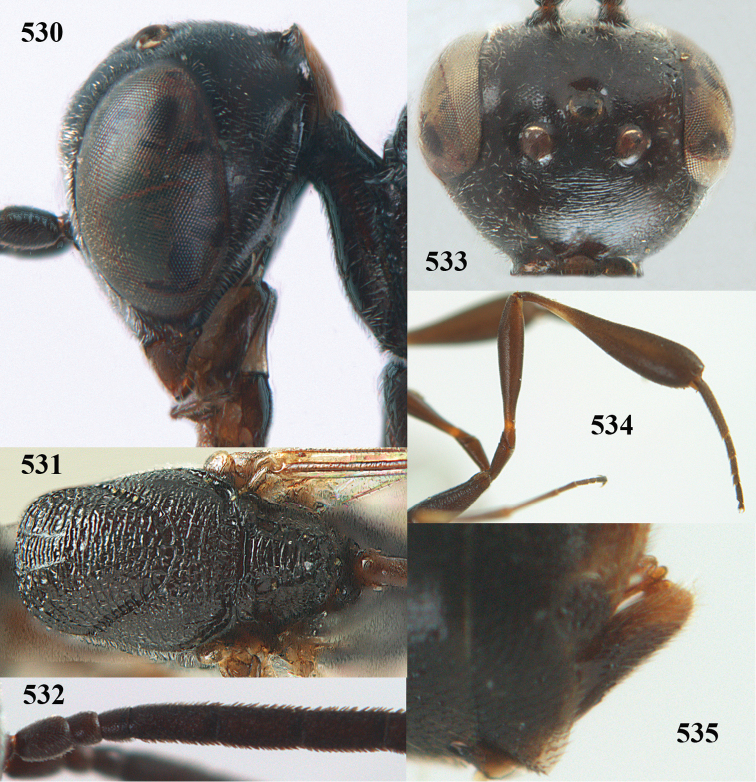
*Gasteruption tournieri* Schletterer, male, France. **530** head lateral **531** mesonotum dorsal **532** basal antennal segments **533** head dorsal **534** hind leg **535** genitalia lateral.

### Gasteruption undulatum

Taxon classificationAnimaliaHymenopteraGasteruptiidae

(Abeille de Perrin, 1879)

Figs 536 –547

Faenus undulatus Abeille de Perrin, 1879: 276.
Gasteruption undulatum ; Schletterer 1885: 315, 1889: 407; Dalla Torre 1902: 1075; Kieffer 1912: 269; Hedicke 1939: 26; Madl 1987a: 404, 1988c 39, 1989b 45, 1990a 130, 1990b 480, 484; Wall 1994: 165; Scaramozzino 1995: 3; Pagliano and Scaramozzino 2000: 11, 19, 34; Saure 2001: 30; Yildirim et al. 2004: 1351; van Achterberg 2013: 83.
Foenus bidentulus Thomson, 1883: 848; Hellén 1950: 4; Hedqvist 1973: 181, 182 (as synonym of *Gasteruption assectator* (Linnaeus)); Oehlke 1984: 175 (id.); Wall 1994: 148. Synonymized with *Gasteruption undulatum* (Abeille de Perrin) by Madl, 1987b.
Gasteruption bidentulum ; Schletterer 1885: 303, 1889: 405; Dalla Torre 1902: 1065; Kieffer 1912: 257; Schmiedeknecht 1930: 379, 381; Hedicke 1939: 8; Hellén 1950: 4; Šedivý 1958: 36, 37, 38; Györfi and Bajári 1962: 47, 51; Dolfuss 1982: 23; Madl 1987b: 24, 1988a: 13, 14.
Gasteruption tibiale ; Schletterer 1889: 402–403 (p.p.); Ferrière 1946: 234, 238, 241 (p.p.).

#### Type material.

Lectotype of *Gasteruption undulatum* here designated, ♀ (MNHN) from S France, “Museum Paris EY 0000003924”, “1u”, “Museum Paris, coll. Abeille de Perrin, 1919”, “*Gasteruption undulatum* Ab., ♀, des. Madl 1987 % Lectotypus”, “Lectotypus, des. Madl, 1987”, metasoma largely missing; according to the original description 4 additional females from Marseille, Bordeaux and Landes, of which one female is in MNHN and labelled as paralectotype by Madl in 1987; he misidentified it as *Gasteruption hastator*, but it is a normal female of *Gasteruption undulatum*. Holotype of *Gasteruption bidentulum* examined, ♀ (ZIL) from Gotland, “G.”, “*bidentulus*”, “Lectotypus *Foenus bidentulus* Thoms., ♀, K.-J. Hedqvist, det. 1972”.

#### Additional material.

**Turkey** (Bilecik; 20 km NW of Igdir; Nigde, Camardi; Mezikiran Gecidi, 20 km E of Gurun; 30 km S of Aksehir; Burdur, 20 km SW Burdur, 940 m; 20 km SW of Bitlis; Bolu, near lake; Sultan Daglari, near Yalvac; Sivas, 45 km E of Yarhisar; Denizli, 35 km SSE of Denizli, 970 m; Mansisa, 40 km NW of Salihli, 150 m; Nevsehir, 20 km S of Nevsehir, Kaymakli, 1200 m).

#### Diagnosis.

**Figures 536–544.**
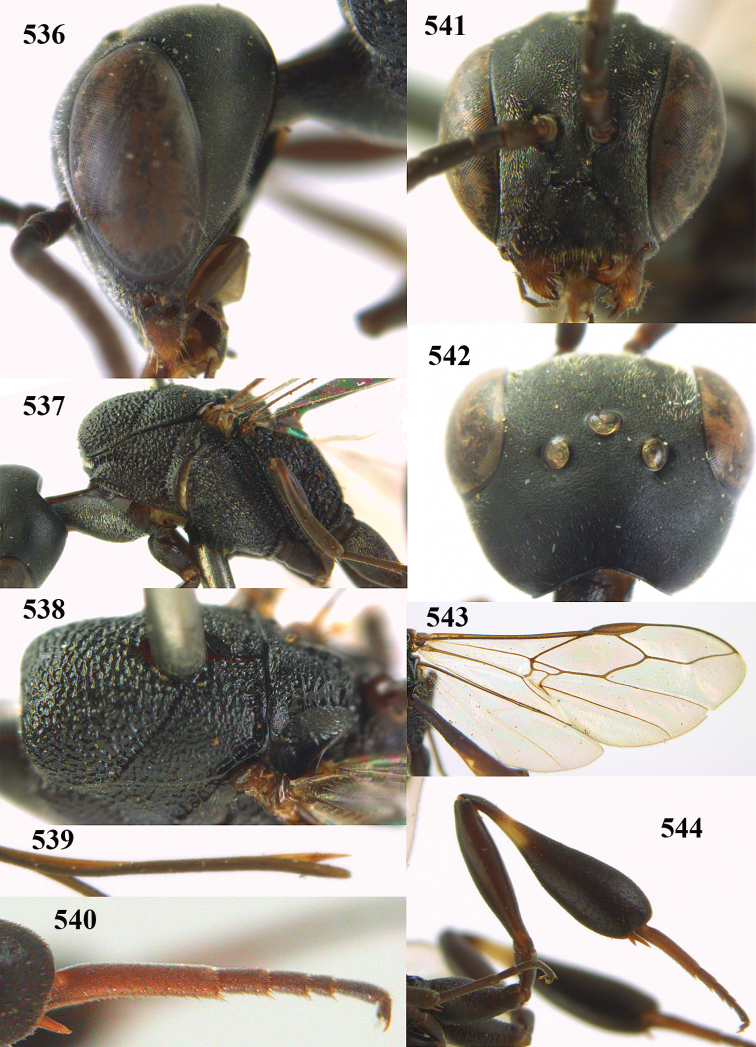
*Gasteruption undulatum* (Abeille de Perrin), female, France. **536** head lateral **537** mesosoma lateral **538** mesonotum dorsal **539** apex of ovipositor sheath **540** hind tarsus lateral **541** head anterior **542** head dorsal **543** fore wing **544** hind leg.

Apex of ovipositor sheath blackish or slightly brownish, if rather pale apically then pale part distinctly shorter than hind basitarsus; ovipositor sheath 1.3–1.5 times as long as hind tibia and 0.7–0.9 times as long as hind tibia and tarsus combined; occipital carina obsolescent medio-dorsally (Figs 536, 542); head, laterally mesosoma and scapus black; head not protruding below eyes; clypeus with small depression or depression obsolescent; apical antennal segment at most 1.2 times as long as third antennal segment and its colour similar to colour of medial segments; antesternal carina narrow; mesoscutum distinctly densely rugose, distinctly different from very finely aciculate vertex; hind tibia stout, with a distinct subbasal ivory ring and distinctly swollen, resulting in a distinctly convex ventral border (Fig. 544); hind basitarsus rather long (Fig. 544); hind tibial spurs yellowish-brown or brown; hind tibial spurs yellowish-brown or brown; hind tarsus (except telotarsus) brownish-yellow to yellowish-brown; incision of hypopygium shallow. Males have third antennal segment about 1.5 times longer than second segment and as long as fifth segment (Fig. 546) and fourth antennal segment about 0.7 times as long as second and third segments combined.

#### Distribution.

Central and South Europe, Turkey.

#### Biology.

Unknown. Collected from June till September, in South Europe present from May onwards.

**Figures 545–547.**
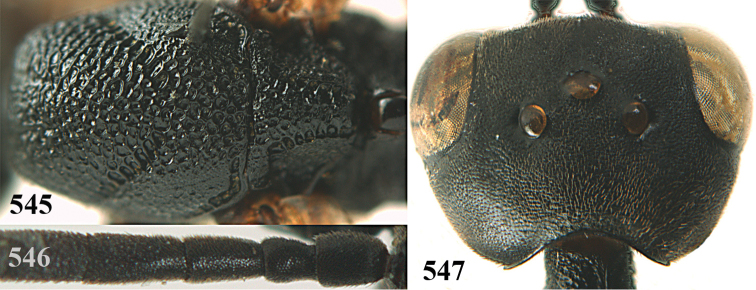
*Gasteruption undulatum* (Abeille de Perrin), male, Hungary. **545** mesonotum dorsal **546** basal antennal segments **547** head dorsal.

### Gasteruption variolosum

Taxon classificationAnimaliaHymenopteraGasteruptiidae

(Abeille de Perrin, 1879)

Figs 548 –562

Faenus variolosus Abeille de Perrin, 1879: 264, 267, 275.
Gasteruption variolosum ; Schletterer 1885: 316, 1889: 408; Dalla Torre 1902: 1075; Kieffer 1912: 250; Hedicke 1939: 26; Ferrière 1946: 236, 238, 247; Madl 1988a: 13, 17, 1990a 130; Wall 1994: 165; Scaramozzino 1995: 3; Pagliano and Scaramozzino 2000: 13, 19, 34.
Gasteruption laeviceps Schletterer, 1885: 281, 1889: 403; Szépligeti 1903: 369; Kieffer 1912: 258; Schmiedeknecht 1930: 379, 381; Ferrière 1946: 247; Hedicke 1939: 15; Wall 1994: 149. Synonymized with *Gasteruption variolosum* (Abielle de Perrin) by Ferrière (1946).
Gasteruption leviceps Schletterer, 1889: 382, 392, 394, 397. Invalid emendation.

#### Type material.

Lectotype of *Gasteruption variolosum* here designated, ♀ (MNHN) from Marseille, “Museum Paris EY 0000003923”, “*variolosus* ab.”, “Museum Paris, coll. Abeille de Perrin, 1919”, “Monotypus, des. Madl, 1987”. Lectotype of *Gasteruption laeviceps* here designated, ♂ (NMW) “[Greece], Rhodus, Erber”.

#### Additional material.

*Iran (Tehran, Shahriar; near Kasan); *Turkey (Agri, Patnos, 1650 m; Capadocia, Ürgüp; 10 km W of Ürgüp; Burdur, 20 km SW of Burdur, 940 m; 20 km E of Alanya; Kopeksiz, 25 km E of Malatya; Nidge, Camardi; Uzuncaburc, 30 km N of Silifke; Kafr. 10 km SE of Suwayda; 20 km NW of Igdir; Cornelek, 40 km E of Mut; near Agri; Hakkari, Akcali, 35 km S of Hakkari, 1700 m; Gevas, Van Gölü; Sivas, 45 km E of Yarhisar; Burdur, 5 km NE of Yesilova, 1060 m; N of Sivas; Isparta, 8 km NE of Isparta, 1020 m; id., Egirdir Gölu, 5 km N of Akkecili, 920 m; Beysehir; Manisa, 15 km SEE of Salihi, 170 m; Nevsehir, 20 km S of Nevsehir, Kaymakli, 1200 m; Nevsehir, 5 km S of Avanos, Zelve, 1000 m; id., 10 km S of Avanos, Göreme, 1000 m; id., 10 km E of Ürgüp, W of Aksalur, 1350 m; Gümüshane, Gümüshane, 1200 m; Hakkari, Mt. Sat, SW of Yüksekova, Varegös, 1650 m; Inner Anatolia, 5 km W of Koyulhisar).

#### Diagnosis.

**Figures 548–556.**
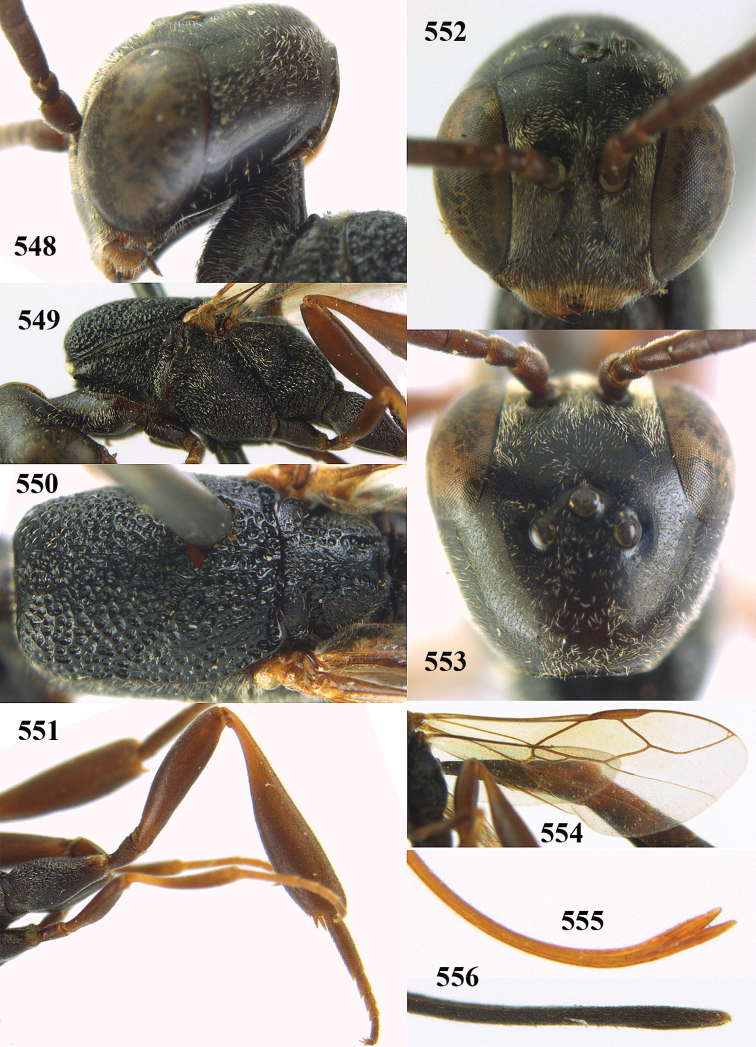
*Gasteruption variolosum* (Abeille de Perrin), female, France. **548** head lateral **549** mesosoma lateral **550** mesonotum dorsal **551** hind leg **552** head anterior **553** head dorsal **554** fore wing **555, 556** apex of ovipositor sheath.

Apex of ovipositor sheath blackish or slightly brownish, if rather pale apically then pale part distinctly shorter than hind basitarsus; ovipositor sheath 1.6–2.0 times as long as hind tibia and at least as long as hind tibia and tarsus combined; occipital carina non-lamelliform, narrow (Figs 548, 553); temple distinctly elongate (Fig. 553), about as long as eye in dorsal view, but sometimes 0.7–0.8 times; apical antennal segment short, about 1.5 times as long as wide and its tip distinctly shiny; eyes at most sparsely setose; face rather wide (Fig. 552); temples strongly narrowed behind eyes in dorsal view (Fig. 553), but less so in male (Fig. 560); head in anterior view less protruding dorsally than in *Gasteruption dolichoderum* (Fig. 552); vertex distinctly bulging near occipital carina and extremely finely coriaceous (Fig. 548); propleuron 0.8–1.1 times distance from tegulae to anterior border of mesoscutum; genal bridge at most half as long as third antennal segment; pronotum with pair of small and more or less blunt protuberances antero-laterally; antesternal carina narrowly lamelliform (Fig. 549); anterior half of mesoscutum more or less coarsely reticulate-rugose (Fig. 550); incision of hypopygium shallow. Males have fourth antennal segment 1.3–1.5 times as long as third segment and apical antennal segment about 1.5 times as long as wide (Fig. 559).

#### Distribution.

South Europe, Iran, Turkey. New for the fauna of Iran and Turkey.

#### Biology.

Unknown. Collected in May-July.

**Figures 557–562.**
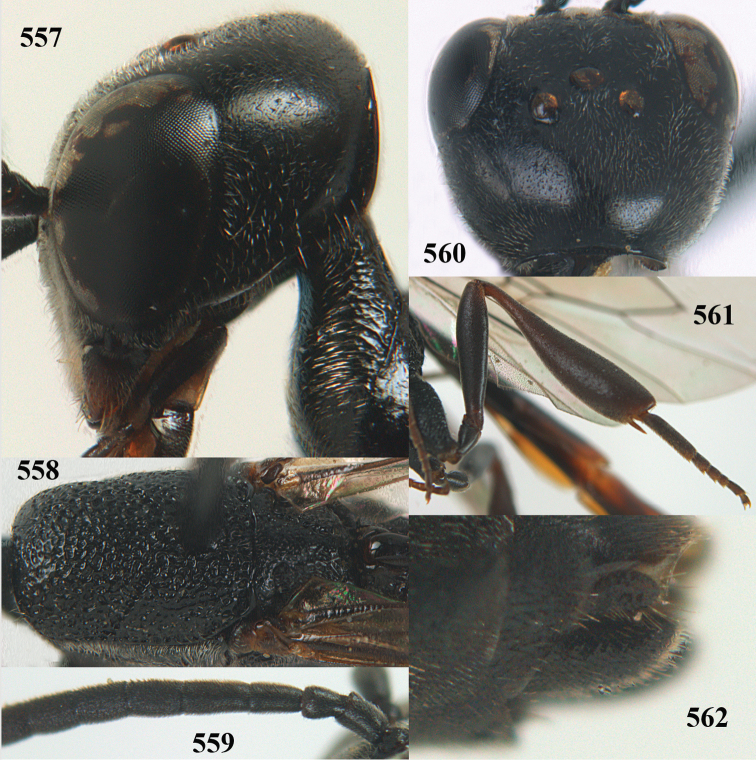
*Gasteruption variolosum* (Abeille de Perrin), male, Turkey. **557** head lateral **558** mesonotum dorsal **559** basal antennal segments **560** head dorsal **561** hind leg **562** genitalia lateral.

## Acknowledgements

Thanks are due to Sergey Belokobylskij (St. Petersburg), Hege Vårdal, Julia Stigenberg and Seraina Klopfstein (Stockholm), Claire Villemant and Agnièle Touret-Alby (Paris), Carmen Rey del Castillo, Isabel Izquierdo and Mercedis París (Madrid), Bernhard Merz (Geneva), Andreas Müller (Zürich), David Notton and Gavin Broad (London), Gloria Ortega Muñoz (Tenerife), Roberto Poggi (Genoa), Sándor Csösz and Zoltán Vas (Budapest), Dominique Zimmermann and Michael Madl (Vienna) for the loan of types and information about available specimens. Chris Saure (Berlin), Christian Schmid-Egger (Berlin) and Fritz Gusenleitner (Linz) for the loan and partly gift of specimens, Chris Saure for his very usueful comments, Anne Freitag (Lausanne) and Ehsan Rakhshani (Zabol) for their hospitality during the visit of the first author and for gift of specimens.

## Supplementary Material

XML Treatment for Gasteruption aciculatum

XML Treatment for Gasteruption agrenum

XML Treatment for Gasteruption assectator

XML Treatment for Gasteruption brevibasale

XML Treatment for Gasteruption caucasicum

XML Treatment for Gasteruption coriacoxale

XML Treatment for Gasteruption diversipes

XML Treatment for Gasteruption dolichoderum

XML Treatment for Gasteruption flavimarginatum

XML Treatment for Gasteruption freyi

XML Treatment for Gasteruption goberti

XML Treatment for Gasteruption hastator

XML Treatment for Gasteruption heminitidum

XML Treatment for Gasteruption henseni

XML Treatment for Gasteruption insidiosum

XML Treatment for Gasteruption ischnolaimum

XML Treatment for Gasteruption jaculator

XML Treatment for Gasteruption laticeps

XML Treatment for Gasteruption lugubre

XML Treatment for Gasteruption merceti

XML Treatment for Gasteruption minutum

XML Treatment for Gasteruption nigrapiculatum

XML Treatment for Gasteruption nigrescens

XML Treatment for Gasteruption opacum

XML Treatment for Gasteruption paglianoi

XML Treatment for Gasteruption phragmiticola

XML Treatment for Gasteruption pseudolaticeps

XML Treatment for Gasteruption punctifrons

XML Treatment for Gasteruption schlettereri

XML Treatment for Gasteruption schmideggeri

XML Treatment for Gasteruption scorteum

XML Treatment for Gasteruption smitorum

XML Treatment for Gasteruption syriacum

XML Treatment for Gasteruption tournieri

XML Treatment for Gasteruption undulatum

XML Treatment for Gasteruption variolosum
